# Phosphoproteomics analysis of a clinical *Mycobacterium tuberculosis* Beijing isolate: expanding the mycobacterial phosphoproteome catalog

**DOI:** 10.3389/fmicb.2015.00006

**Published:** 2015-02-10

**Authors:** Suereta Fortuin, Gisele G. Tomazella, Nagarjuna Nagaraj, Samantha L. Sampson, Nicolaas C. Gey van Pittius, Nelson C. Soares, Harald G. Wiker, Gustavo A. de Souza, Robin M. Warren

**Affiliations:** ^1^Division of Molecular Biology and Human Genetics, Faculty Medicine and Health Sciences, DST/NRF Centre of Excellence for Biomedical Tuberculosis Research, SAMRC Centre for Tuberculosis Research, Stellenbosch UniversityCape Town, South Africa; ^2^The Gade Research Group for Infection and Immunity, Department of Clinical Science, University of BergenBergen, Norway; ^3^Max Planck Institute for BiochemistryMunich, Germany; ^4^Faculty of Health Sciences, Institute of Infectious Disease and Molecular Medicine, University of Cape TownCape Town, South Africa; ^5^Norway Proteomics Core Facility, Department of Immunology, Oslo UniversityOslo, Norway

**Keywords:** *M. tuberculosis*, phosphoproteomics, tyrosine phosphorylation, serine phosphorylation, threonine phosphorylation

## Abstract

Reversible protein phosphorylation, regulated by protein kinases and phosphatases, mediates a switch between protein activity and cellular pathways that contribute to a large number of cellular processes. The *Mycobacterium tuberculosis* genome encodes 11 Serine/Threonine kinases (STPKs) which show close homology to eukaryotic kinases. This study aimed to elucidate the phosphoproteomic landscape of a clinical isolate of *M. tuberculosis*. We performed a high throughput mass spectrometric analysis of proteins extracted from an early-logarithmic phase culture. Whole cell lysate proteins were processed using the filter-aided sample preparation method, followed by phosphopeptide enrichment of tryptic peptides by strong cation exchange (SCX) and Titanium dioxide (TiO_2_) chromatography. The MaxQuant quantitative proteomics software package was used for protein identification. Our analysis identified 414 serine/threonine/tyrosine phosphorylated sites, with a distribution of S/T/Y sites; 38% on serine, 59% on threonine and 3% on tyrosine; present on 303 unique peptides mapping to 214 *M. tuberculosis* proteins. Only 45 of the S/T/Y phosphorylated proteins identified in our study had been previously described in the laboratory strain H_37_Rv, confirming previous reports. The remaining 169 phosphorylated proteins were newly identified in this clinical *M. tuberculosis* Beijing strain. We identified 5 novel tyrosine phosphorylated proteins. These findings not only expand upon our current understanding of the protein phosphorylation network in clinical *M. tuberculosis* but the data set also further extends and complements previous knowledge regarding phosphorylated peptides and phosphorylation sites in *M. tuberculosis.*

## Introduction

According to the World Health Organization (WHO), tuberculosis (TB) ranks as the second leading cause of death from an infectious disease worldwide, after HIV (WHO|Global tuberculosis report 2014, 2014). It is estimated that one third of the world's population is infected with *Mycobacterium tuberculosis*, the causative agent of TB and 8.6 million new TB cases were reported in 2012 alone (WHO|Global tuberculosis report 2013, 2013). In order to control this epidemic there is a critical need for the development of effective and affordable anti-TB therapy and diagnostic tools.

Harnessing the power of the field of proteomics provides a unique opportunity to identify novel protein candidates for diagnosis and drug targets of pathogenic bacteria. Of particular interest is the identification of proteins with post-translational modifications (PTMs) as these modifications are often critical to protein functions, such as regulating protein-protein interactions, subcellular localization or modification of catalytic sites (Seo and Lee, 2004; Gupta et al., 2007). Protein phosphorylation is an important reversible PTM that directly or indirectly regulates signal transduction cascades linking the intracellular and extracellular environments. In bacteria, protein phosphorylation plays a fundamental role in the regulation of key processes ranging from metabolism and cellular homeostasis to cellular signaling which can be mediated by two classes of phosphorylation events (Cozzone, 1998). The underlying molecular mechanisms regulating protein phosphorylation and dephosphorylation is of great physiological importance due to its ability to ultimately affect protein activity, function, half-life or subcellular localization (McConnell and Wadzinski, 2009). Until recently it was thought that histidine/aspartate phosphorylation was the main mediator of signal transduction in bacteria (Frasch and Dworkin, 1996). However, with the advancement of mass spectrometry-based analyses serine/threonine and tyrosine kinases have been identified in a number of different bacteria (Macek et al., 2007; Macek and Mijakovic, 2011; Mijakovic and Macek, 2012).

The *M. tuberculosis* genome encodes 11 Serine/Threonine kinases (STPK's) (PknA, PknB, PknD, PknE, PknF, PknG, PknH, PknI, PknJ, PknK, PknL), two tyrosine phosphatases (PtpA, PtpB) and 11 two-component systems, highlighting the complexity of signaling network mediated by protein phosphorylation and thereby their potential as drug targets (Chopra et al., 2003; Koul et al., 2004; Sharma et al., 2004; Sala and Hartkoorn, 2011). Prisic et al. described the Serine/Threonine (S/T) phosphorylation profiles of the laboratory strain *M. tuberculosis* H_37_Rv under 6 different culture conditions (Prisic et al., 2010). This study identified 301 phosphorylated proteins after combining data from six different culture conditions (Prisic et al., 2010) and identified four phosphorylated STPKs, ribosomal and ribosome-associated proteins as well as phosphorylated substrates which suggest that protein phosphorylation provides a mechanism for regulating key physiological process during infection. A more recent study of H_37_Rv further expanded the knowledge of the phosphoproteome by identifying novel tyrosine (Y) phosphorylated proteins in *M. tuberculosis* further supporting the broad regulation of its physiology by phosphorylation (Kusebauch et al., 2014).

In this study we report the phosphoproteome of a previously described clinical Beijing genotype *M. tuberculosis* isolate at early-logarithmic growth phase in liquid culture to provide further insight the influence of S/T/Y phosphorylation events on bacterial growth and virulence (de Souza et al., 2010). We used a combination of strong cation exchange (SCX) with Titanium dioxide (TiO_2_) enrichment in a mass spectrometry-based phosphoproteomic analysis of a hyper-virulent clinical *M. tuberculosis* isolate (de Souza et al., 2010). We confirmed the presence of previously described *M. tuberculosis* phosphorylated proteins and also identified novel phosphorylated proteins and sites. In addition, this dataset identified novel tyrosine phosphorylation events, and thereby confirmed that there are multiple tyrosine kinase targets in this clinically relevant *M. tuberculosis* strain.

## Materials and methods

### Cell culture and lysate preparation

A previously described hyper-virulent clinical Beijing genotype *Mycobacterium tuberculosis* isolate, SAW5527, isolated from a TB patient attending a primary health care clinic in the Western Cape province, South Africa was used for this phosphoproteomics analysis (de Souza et al., 2010). Secondary cultures were inoculated into 50 ml 7H9 Middlebrooks medium supplemented with Dextrose and Catalase and incubated at 37°C until early-logarithmic phase (OD_600_ between 0.6 and 0.7). Mycobacterial cells were collected by centrifugation (2000 × g for 10 min at 4°C) and washed two times with cold lysis buffer containing 10 mM Tris-HCl (pH 7.4), 0.1% Tween-80, Complete Protease inhibitor cocktail (Roche, Mannheim Germany) and Phosphatase inhibitor cocktail (Roche, Mannheim Germany). An equal amount of 0.1 mm glass beads (Biospec Products Inc., Bartlesville, OK) was added to the cell pellet after centrifugation together with cold 300 μl lysis buffer and 10 μl DNaseI (2U/ml) (NEB, New England Laboratories). Lysis was achieved by mechanical bead-beating in a Rybolyser (Bio101 SAVANT, Vista, CA) for 6 cycles of 20 s at a speed of 4.0 m.s^−1^, with 1 min cooling periods on ice. The whole cell lysates were filter-sterilized with a sterile 0.22 μm pore acrodisc 25 mm PF syringe filter (Pall Life Sciences, Pall Corporation, Ann Arbour, MI) and stored at −80°C. The protein concentration of the whole cell lysate was determined using the RC DC Protein assay according to manufacturer's instructions (BioRad). A single biological replicate was analyzed in triplicate for downstream phosphoproteomic analysis.

### Filter aided sample preparation and trypsin digestion

Four milligrams of concentrated whole cell lysate proteins was heated in 4% SDS buffer and 0.1 M dithiothreitol (DTT) in 100 mM Tris/HCl pH 7.5. The samples were processed using Filter Aided Sample Preparation (FASP) (Wiśniewski et al., 2009). In brief, 4 mg dried whole cell lysate protein was resuspended in 250 μl of urea (UA) and loaded onto a 15 ml Amicon filtration device (30 kDa MWCO) and centrifuged at 2000 × g for 40 min at 25°C. After centrifugation, the flow-through was collected in a clean falcon tube and discarded. The concentrated whole cell lysate proteins in the filter unit were diluted in 2 ml 8 M Urea in 0.1 M Tris/HCl (pH 8.5) and re-centrifuged to remove the SDS. The flow-through was discarded and the remaining proteins in the filter unit were alkylated by mixing with 1.5 ml 50 mM iodacetamide (IAA) and incubated in the dark for 20 min to irreversibly modify cysteine. The alkylated proteins were equilibrated with 2 ml 50 mM ammonium bicarbonate (ABC) and digested with trypsin (Promega) in a protein to enzyme ratio of 100:1 at 37°C overnight. After trypsin digestion the filter unit is transferred to a clean collection tube and the peptides were eluted by centrifuged at 14 000 × g for 10 min. The eluted peptides were diluted in 50 μl water to avoid desalting for further processing of the peptide and acidified with trifluoroacetic acid (TFA).

### Fractionation of peptides by strong cation exchange (SCX)

Extracted trypsin digested peptides were diluted to a volume of 7 ml in Solvent A (5 mM monopotassium phosphate (KH_2_PO_4_) 30% acetonitrile (ACN), pH 2.7). The pH of the diluted peptide samples was adjusted to 2.7 and made up to a volume of 10 ml with 100% ACN. The respective peptide samples were then separated at pH 2.7 by strong cation exchange (SCX ) by loading each peptide mixture onto a cation exchanger column (3.0 mm × 20 cm) (Poly LC, Columbia, MD) containing 5 μm polysulfoethyl aspartamine beads with a 200 Å pore size and a flow rate of 350 μl/min^−1^ equilibrated with SCX solvent A. The flow-through was collected and the bound peptides were eluted from the columns using an increasing salt gradient (0–30%) containing 5 mM KH_2_PO_4_ pH and 150 mM KCl. A total of 9 fractions were collected; 5 fractions generated by SCX based on UV absorbance (220 and 280), 3 from the flow-through and 1 salvage fraction (containing washes from the cation exchanger column) from the SCX column as an additional fraction.

### Enrichment of phosphopeptides with TiO_2_ beads

All nine fractions (5 SCX, 3 SCX flow-through, 1 salvage fraction) were subjected to Titanium dioxide (TiO_2_) phosphopeptide enrichment. The TiO_2_-beads,10 μm in size, (GL Sciences, Inc., Japan) was resuspended 30 mg/ml dihydrobenzoic acid (DHB) (Sigma) to prevent non-specific binding. Each of the 9 fractions was incubated 4 times with TiO_2_ at a peptide to bead ratio of 1:2–1:8. Each fraction was rotated for 30 min, and then briefly centrifuged (14,000 g × 30 s). The supernatants were aspirated and transferred to a new labeled tube and the phosphopeptides bound to the TiO_2_beads were washed twice with 30% ACN and 3% TFA followed by washing twice with 75% ACN and 0.3% TFA. The enriched phosphorylated peptides were eluted with elution buffer containing 25% ammonia and ACN pH10. The eluted phosphopeptides were desalted using in house prepared C_18_ Stage tips.

### LC-MS/MS analysis

The peptides were separated on a column packed in-house with C18 beads (reprosil-AQ Pur, Rd Maisch) on an Proxeon Easy-nLC system (Proxeon Biosystems, Odense, Denmark) using a binary gradient with buffer A (0.5% acetic acid in water) and buffer B (0.5% acetic acid and 80% ACN). The 4 μl of the enriched phosphopeptides from each of the 9 fractions were injected 3 times and were loaded directly without any trapping column with buffer A at a flow rate of 500 nl/min. Elution was carried out at a flow rate of 250 nl/min, with a linear gradient from 10 to 35% buffer B over a period of 95 min followed by 50% buffer B for 15 min. At the end of the gradient the column was washed with 90% buffer B and equilibrated with 5% buffer B for 10 min. The LC system was directly coupled in-line with the LTQ-Orbitrap Velos instrument (Thermo Fisher Scientific) via the Proxeon Biosystems nanoelectrospray source. The mass spectrometer was programmed to acquire in a data-dependant mode with a resolution of 30,000 at 400 m/z with lock mass option enabled for the 445.120025. However, the target lock mass abundance was set to 0% in order to save the injection time for lock mass. For full scans 1E6 ions were accumulated within a maximum injection time of 250 ms in the C trap and detected in the Orbitrap mass analyser. The 10 most intense ions with charge states ≥2 were sequentially isolated and fragmented by high-energy collision dissociation (HCD) mode in the collision cell with normalized collision energy of 40% and detected in the Orbitrap analyser at 75,000 resolution. For HCD based method, the activation time option in the Xcalibur file was set to 0.1 ms. For the high-low strategy, full scans were acquired in the Orbitrap analyser at 60,000 resolution as parallel acquisition is enabled in the high-low mode. Up to the 10 most intense peaks with charge states ≥2 were selected for sequencing to a target value of 5000 with a maximum injection time of 25 ms and fragmented in the ion trap by HCD with normalized collision energy of 35%, activation of 0.25 and activation time of 10 ms.

### Database search

The raw data acquired were processed using MaxQuant software version (1.4.1.2) and processed as per default workflow. Since HCD spectra were acquired in profile mode, deisotoping was performed similar to survey MS scans to obtain singly charged peak lists and searched against the *M. tuberculosis* H_37_Rv protein database (version R11 tuberculist.epfl.ch). The search included cysteine carbamidomethylation as a fixed modification while N-acetylation, oxidation of methionine and phosphorylation at serine, threonine and tyrosine were set as variable modifications. Up to two missed cleavages were allowed for protease digestion and a peptide had to be fully tryptic. Identifications were filtered at 1% FDR at three levels namely; site, peptide and protein using reversed sequences. As such there is no fixed cut-off score threshold but instead spectra were accepted until the 1% false discovery rate (FDR) is reached. Only peptides with a minimum length of 7 amino acids were considered for identification and detected in at least one or more of the replicates. All phosphopeptide spectra were manually validated by applying stringent acceptance criteria: only phosphorylation events on S/T/Y with a localization probability of ≥0.75 and PEP ≤ 0.01 were used for further analysis and reported as high confidence localized phosphosites.

### Gene ontology analysis

The categorization of identified phosphorylated proteins in terms of functional categorization, molecular function, biological processes and cellular components was carried out using TubercuList-*Mycobacterium tuberculosis* Database.

## Results

**Figure 1:**
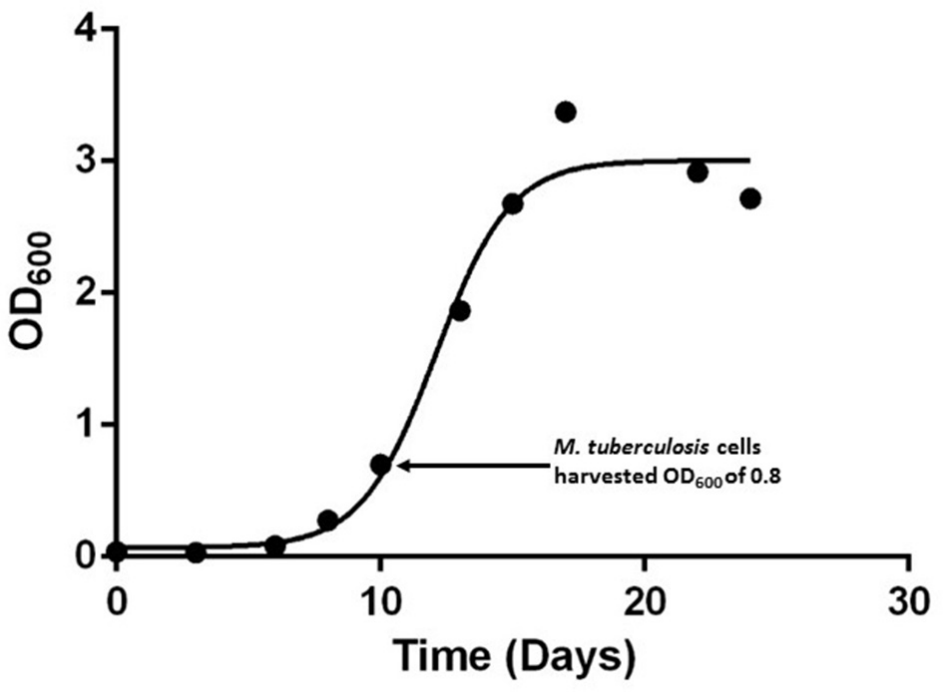
**Growth curves of hyper-virulent *M. tuberculosis* Beijing strain**. Growth monitored over a 24 day period using OD_600_ measurements in 7H9 Middlebrooks liquid media supplemented with dextrose and catalase.

In this study we set out to analyse the phosphoproteome of a hyper-virulent Beijing genotype *M. tuberculosis* isolate by extracting whole cell lysate proteins at early-logarithmic growth (OD_600_ of 0.8) (Figure 1) which resulted in the identification of 619 MS/MS spectra. The 274 LC-MS/MS spectra that fulfilled the criteria for high confidence phosphosites identified a total of 414 (38:59:3%) S/T/Y phosphorylation sites present in 214 *M. tuberculosis* H_37_Rv proteins (Supplementary Table S2; Supplementary Figure S1).

**Figure 2:**
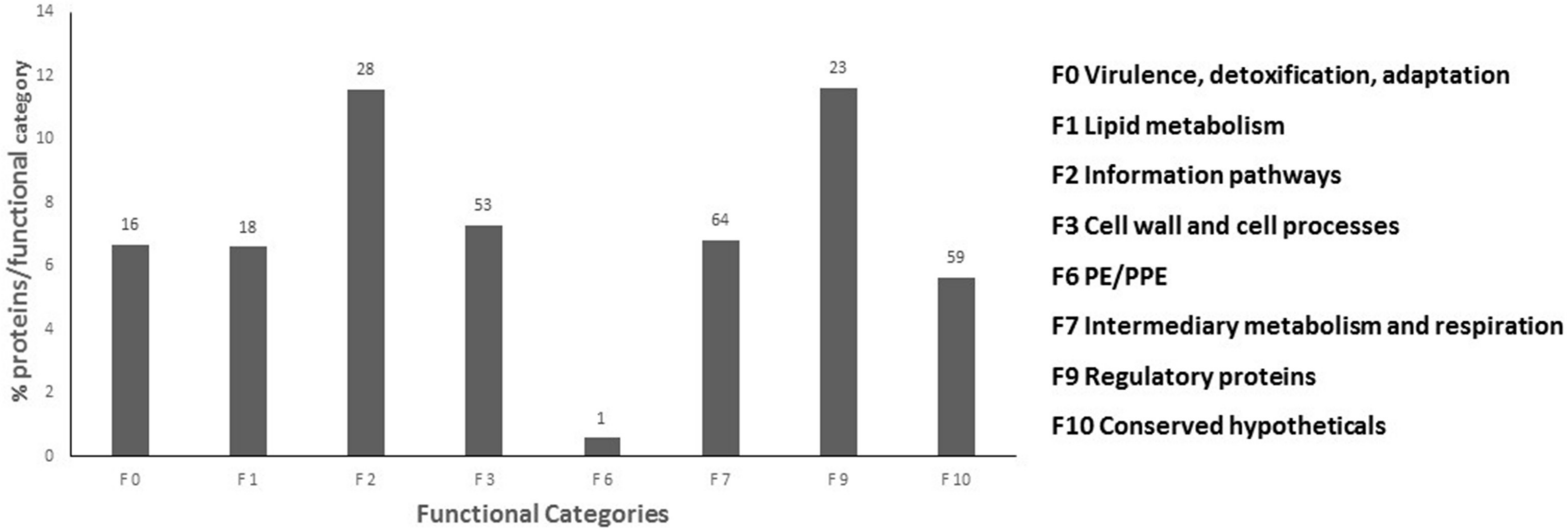
**Functional classification of phosphorylated proteins**. Percentage phosphorylated proteins per functional category were classified according to Tuberculist. Number of phosphorylated proteins identified in each functional category depicted above the black bars.

Of the 401 serine/threonine phosphorylation sites (pS/T), only 156 had been previously described for *M. tuberculosis* (Table 1). Only 6 of 13 tyrosine phosphorylation sites (pY) has been previously described for *M. tuberculosis* (Kusebauch et al., 2014). The remaining 245 pS/T and 7 pY were uniquely identified in this study (Supplemental Table S2). To determine whether phosphorylated proteins were differentially represented in any particular cellular process, we grouped the phosphorylated proteins based on their functional category according to Tuberculist (Lew et al., 2011) (Figure 2). One hundred and seventy (79.4%) of the phosphorylated proteins had an annotated function, while the remaining 59 phosphorylated proteins (20.5%) were assigned as hypothetical. The biological function of the annotated proteins varied from transcription, translation, protein biosynthesis, fatty acid metabolism to phosphorylation. Our analysis identified phosphorylated forms of the 9 of the 11 *M. tuberculosis* STPK's: PknA, PknB, PknD, PknF, PknG PknE, PknH, PknJ, and PknL (Table 2). Of these, phosphorylated forms of PknE, PknH, PknJ, and PknL had not been previously described in *M. tuberculosis*.

**Table 1:** **List of phosphopeptides identified in this and previous studies of *M. tuberculosis***.

**Rv numbers**	**Protein name**	**Phosphopeptides**	**Phospho-residue**	**References**
Rv0007	Rv0007	FISGASAPVTGPAAAVR	Not known	Prisic et al., 2010
		FIS^*^GAS^*^APVT^*^GPAAAVR	S; T	This study
		FIS^*^GASAPVT^*^GPAAAVR	S; T	This study
Rv0014c	PknB	AIADSGNSVTQTAAVIGTAQYLSPEQAR	Not known	Prisic et al., 2010
		AIADSGNSVT^*^QT^*^AAVIGTAQYLSPEQAR	T	This study
		AIADSGNS^*^VT^*^QTAAVIGTAQYLSPEQAR	S; T	This study
		TSLLSSAAGNLSGPRTDPLPR	Not known	Prisic et al., 2010
		TSLLSSAAGNLS^*^GPRTDPLPR	S	This study
		TSLLSSAAGNLSGPRT^*^DPLPR	T	This study
Rv0015c	PknA	RPFAGDGALT^$^VAMK	T	Prisic et al., 2010; This study
Rv0020c	FhaA	FEQSSNLHTGQFR	Not known	Prisic et al., 2010
		FEQS^*^SNLHT^*^GQFR	S; T	This study
		HPDQGDY^$^PEQIGYPDQGGYPEQR	Y	Kusebauch et al., 2014; This study
		QDYGGGADY^$^TR	Y	Kusebauch et al., 2014; This study
		VPGY^$^APQGGGYAEPAGR	Y	Kusebauch et al., 2014; This study
Rv0175	Rv0175	AADSAESDAGADQTGPQVK	Not known	Prisic et al., 2010
		AADSAESDAGADQT^*^GPQVK	T	This study
Rv0204c	Rv0204c	DPPT^$^DPNLR		Prisic et al., 2010; This study
Rv0227c	Rv0227c	GGFEEPVPGAEAETEKLPTQRPDFPR	Not known	Prisic et al., 2010
		GGFEEPVPGAEAET^$^EK	T	Prisic et al., 2010; This study
Rv0351	GrpE	RIDPET^#^GEVR	T	Prisic et al., 2010
		IDPET^*^GEVR	T	This study
Rv0389	PurT	AAGHQVQPQT^$^GGVSPR	T	Prisic et al., 2010; This study
Rv0410c	PknG	SGPGTQPADAQTAT^#^SATVRPL	T	O'Hare et al., 2008
		S^*^GPGT^*^QPADAQTATSATVR	S; T	This study
		SGPGTQPADAQTAT^*^S^*^ATVRPLSTQAVFR	S; T	This study
		SGPGTQPADAQTAT^*^SAT^*^VR	T	This study
		PLST^#^QAVFRPDFGDEDNFPHPTLGPDTEPQDR	T	O'Hare et al., 2008
		PLS^*^T^*^QAVFR	S; T	This study
Rv0421c	Rv0421c	GLAEGPLIAGGHS^#^YGGR	S	Prisic et al., 2010
		GLAEGPLIAGGHS^*^Y^*^GGR	S; Y	This study
Rv0440	GroEL2	KWGAPTIT^$^NDGVSIAK	T	Molle et al., 2006
		WGAPTIT^*^NDGVSIAK	T	This study
		AVEKVT^#^ETLLK	T	Molle et al., 2010
		VTET^*^LLK	T	This study
Rv0497	Rv0497	RGDSDAITVAELT^$^GEIPIIR	T	Prisic et al., 2010; This study
		T^*^GPHPETESSGNR	T	This study
Rv0685	Tuf	PDLNET^$^KAFDQ	T	Sajid et al., 2011
		VLHDKFPDLNET^*^K	T	This study
Rv0733	Adk	LGIPQISTGELFR	Not known	Prisic et al., 2010
		LGIPQIS^*^TGELFR	S	This study
Rv0822c	Rv0822c	VHDDADDQQDTEAIAIPAHSLEFLSELPDLR	Not Known	Prisic et al., 2010
		VHDDADDQQDT^*^EAIAIPAHSLEFLSELPDLR	T	This study
Rv0896	GltA2	ADTDDT^$^ATLR	T	Prisic et al., 2010; This study
Rv0931c	PknD	PGLTQT^$^GTAVG	T	Durán et al., 2005; This study
		AASDPGLT^*^QT^*^GTAVGTYNYMAPER	T	This study
		WSPGDS^*^AT^*^VAGPLAADSR	S; T	This study
		WSPGDSATVAGPLAADSR	Not known	Prisic et al., 2010
		WSPGDS^*^AT^*^VAGPLAADSR	S; T	This study
		WSPGDS^*^ATVAGPLAADS^*^R	S	This study
Rv1388	MihF	AQEIMTELEIAPT^$^RR	T	Prisic et al., 2010; This study
Rv1719	Rv1719	S^$^GGIQVIAR	S	Prisic et al., 2010This study
Rv1746	PknF	DDTRVS^$^QPVAV	S	Durán et al., 2005; This study
Rv1747	Rv1747	YPTGGQQLWPPSGPQR	T	Prisic et al., 2010
		YPT^*^GGQQLWPPS^*^GPQR	S; T	This study
		IPAAPPSGPQPR	Not known	Prisic et al., 2010
		IPAAPPS^*^GPQPR	S	This study
Rv1820	IlvG	STDTAPAQTMHAGR	Not Known	Prisic et al., 2010
		STDT^*^APAQTMHAGR	T	This study
Rv1827	GarA	DQTSDEVTVETTSVFR	Not known	Prisic et al., 2010
		DQTSDEVT^*^VET^*^T^*^SVFR	T	This study
		VTVETT^$^SVFRA	T	Prisic et al., 2010; This study
		DQTSDEVTVET^$^TSVFR	T	Villarino et al., 2005; This study
		DQT^*^SDEVTVET^*^TSVFR	T	This study
		DQTSDEVT^*^VET^*^TSVFR	T	This study
		DQTSDEVTVET^*^T^*^SVFR	T	This study
		DQTSDEVT^*^VET^*^T^*^SVFR	T	This study
Rv2094c	TatA	VDPSAASGQDS^*^T^*^EARPA	S; T	This study
		AEASIETPTPVQSQR	Not known	Prisic et al., 2010
		AEAS^*^IETPTPVQSQR	S	This study
		AEASIETPT^*^PVQSQR	T	Prisic et al., 2010; This study
		VDPSAASGQDSTEARPA	Not known	Chou et al., 2012
		VDPSAASGQDST^*^EARPA	T	This study
Rv2127	AnsP1	ERLGHT^$^GPFPAVANPPVR	T	Prisic et al., 2010; This study
Rv2151c	FtsQ	VADDAADEEAVT^#^EPLATESK	T	Prisic et al., 2010; This study
Rv2197c	Rv2197c	MAEAEPATRPT^#^GASVR	T	Prisic et al., 2010; This study
		MAEAEPAT^#^RPTGASVR	T	Prisic et al., 2010
		MAEAEPAT^*^RPT^*^GASVR	T	This study
Rv2198c	MmpS3	ASGNHLPPVAGGGDKLPSDQTGETDAYSR	Not known	Prisic et al., 2010
		ASGNHLPPVAGGGDKLPSDQT^*^GETDAY^$^SR	T; Y	Kusebauch et al., 2014; This study
Rv2536	Rv2536	ADDSPTGEMQVAQPEAQTAAVATVER	Not known	Prisic et al., 2010
		AADTDVFSAVR	Not known	Prisic et al., 2010
		AADT^*^DVFS^*^AVR	T	This study
Rv2536	Rv2536	EAPT^$^EVIR	T	Prisic et al., 2010; This study
		ADDSPT^*^GEMQVAQPEAQTAAVAT^*^VER	T	This study
		ADDS^*^PTGEMQVAQPEAQTAAVATVER	S	This study
Rv2606c	SnzP	MDPAGNPATGT^$^AR	T	Prisic et al., 2010; This study
		MDPAGNPAT^*^GTAR	T	This study
Rv2694c	Rv2694c	RIPGIDT^$^GR	T	Prisic et al., 2010; This study
Rv2696c	Rv2696c	EAAAAQADT^#^QRQAAAGVAR	T	Prisic et al., 2010
		EAAAAQADT^*^QR	T	This study
Rv2921	FtsY	IDTSGLPAVGDDATVPR	Not known	Prisic et al., 2010
		IDTS^*^GLPAVGDDATVPR	S	This study
		IDT^*^SGLPAVGDDAT^*^VPR	T	This study
		IDT^*^SGLPAVGDDAT^*^VPR	T	This study
Rv2940c	Mas	ALAQYLADTLAEEQAAAPAAS^$^	S	Prisic et al., 2010; This study
Rv2996c	SerA1	SATTVDAEVLAAAPK	Not known	Prisic et al., 2010
		SAT^*^TVDAEVLAAAPK	T	This study
		S^*^ATTVDAEVLAAAPK	S	This study
Rv3181c	Rv3181c	LAALDST^#^DTLER	T	Prisic et al., 2010
		LAALDST^*^DT^*^LER	T	This study
Rv3197	Rv3197	S^#^KDEVTAELMEK	S	Prisic et al., 2010
Rv3200c	Rv3200c	QSGADTVVVSS^$^ETAGR	S	Prisic et al., 2010; This study
		QSGADTVVVS^*^SETAGR	S	This study
Rv3248c	SahH	GVTEETTTGVLR	Not known	Prisic et al., 2010
		GVTEETT^*^T^*^GVLR	T	This study
		GVTEET^*^TTGVLR	T	This study
Rv3604c	Rv3604c	TADTPPDDSGGLHAR	Not known	Prisic et al., 2010
		TADTPPDDS^*^GGLHAR	S	This study
		DPLTGGQSVADLMAR	Not known	Prisic et al., 2010
		DPLT^$^GGQSVADLMAR	T	Prisic et al., 2010; This study
Rv3801c	FadD32	FDPEDTSEQLVIVGER	Not known	Prisic et al., 2010
		FDPEDT^*^SEQLVIVGER	T	This study
Rv3817	Rv3817	LWQAEDDS^*^S^*^R	S	This study
		RLWQAEDDSSR	Not known	Prisic et al., 2010
Rv3868	EccA1	LAQVLDIDT^$^LDEDRLR	T	Prisic et al., 2010; This study

* Novel phosphorylated amino acid identified in this study.

# Phosphorylated residue identified in previous studies.

$ Phosphorylated residue identified in current and previous studies.

**Table 2:** **Serine/threonine kinases and their phosphorylation sites identified in this study**.

**STPK**	**Phosphorylated residue (position in protein)**	**Phosphopeptides**	**References**
PknA	T^8^, S^10^, T^224^, S^299^, T^301^, T^302^	AAPAAIPS^*^GT^*^T^*^AR	This study
		VGVT^*^LS^*^GR	This study
		RPFAGDGALT^#^VAMK	Prisic et al., 2010
PknB	S^169^, T^171^, T^173^, S^305^, T^309^	AIADSGNS^*^VT^*^QTAAVIGTAQYLSPEQAR	This study
		TSLLSSAAGNLS^*^GPRTDPLPR	This study
		AIADSGNSVT^*^QT^*^AAVIGTAQYLSPEQAR	This study
		TSLLSSAAGNLSGPRT^*^DPLPR	This study
PknD	T^169^, T^171^, S^332^,T^334^,S^343^,S^350^	GGNWPS^*^QTGHSPAVPNALQASLGHAVPPAGNK	This study
		WSPGDS^*^AT^*^VAGPLAADSR	This study
		WSPGDS^*^ATVAGPLAADS^*^R	This study
		AASDPGLT^*^QT^*^GTAVGTYNYMAPER	This study
PknE	S^304^	LPVPSTHPVS^*^PGTR	This study
PknF	T^289^, S^290^	LGGAGDPDDT^*^RVS^*^QPVAVAAPAK	This study
PknG	S^10^, T^14^, T^23^, S^24^, T^26^, S^31^, T^32^, T^55^	PLS^*^T^*^QAVFR	This study
		S^*^GPGT^*^QPADAQTATSATVR	This study
		SGPGTQPADAQTAT^*^S^*^ATVRPLSTQAVFR	This study
		PDFGDEDNFPHPTLGPDT^*^EPQDR	This study
		SGPGTQPADAQTAT^*^SAT^*^VR	This study
PknH	T^174^	LTQLGT^*^AVGTWK	This study
PknJ	S^498^	HLADLAS^*^IWRR	This study
PknL	S^306^, T^309^	S^*^RIT^*^QQGQLGAK	This study

* Novel phosphorylation sites identified in this study.

# Phosphorylated residue identified in previous studies.

We detected 13 Y phosphorylation sites located on 10 proteins in *M. tuberculosis* during early-logarithmic growth. Six of the 13 Y phosphorylation sites (Table 3) were located on two proteins, FhaA and MmpS3 (Kusebauch et al., 2014) and have recently been described in a *M. tuberculosis* H_37_Rv at stationary growth phase. The remaining 7 Y phosphorylated sites were uniquely identified in this study. Amongst these with known annotations were 2 virulence factors (GroS and GroEL2) and Ppa involved in macromolecule biosynthesis.

**Table 3:** **Tyrosine phosphorylation sites identified**.

**Rv number**	**Protein name**	**Tyrosine phosphopeptides**	**Biological Function**	**References**
Rv0020c	FhaA	GGQGQGRPDEY^*^YDDR	Signal transduction	This study
		GGYPPETGGYPPQPGY^*^PRPR		This study
		HEEGSY^*^VPSGPPGPPEQR		This study
		HPDQGDY^$^PEQIGYPDQGGYPEQR		Kusebauch et al., 2014; This study
		QDYGGGADY^$^TR		Kusebauch et al., 2014; This study
		VPGY^$^APQGGGYAEPAGR		Kusebauch et al., 2014; This study
Rv0421c	Rv0421c	GLAEGPLIAGGHS^*^Y^*^GGR	Hypothetical	This study
Rv0440	GroEL2	QEIENSDSDY^$^DREK	Protein refolding	Kusebauch et al., 2014; This study
Rv0613c	RecC	IVLAGY^*^DEELLER	Exonuclease V gamma chain	This study
Rv1513	Rv1513	HQDAFPPANY^*^VGAQR	Hypothetical	This study
Rv2198c	MmpS3	ASGNHLPPVAGGGDKLPSDQT^*^GETDAY^*^SR	Integral Membrane protein	This study
		AYS^*^APESEHVTGGPY^$^VPADLR		Kusebauch et al., 2014
		AYS^*^APESEHVT^*^GGPY^*^VPADLR		This study
Rv3418c	GroS	Y^*^GGTEIK	Response to stress	This study
Rv3628	Ppa	HFFVHY^*^K	Phosphate metabolic process	This study

^#^Previously identified pY site.

* Novel pY site identified in this study.

$ pY site identified in current and previous studies.

A large number of proteins involved in intermediary metabolism and respiration processes such as lipid and fatty acid metabolism (KasB, FadD32, AccD4, and MmaA3) were found to be phosphorylated in this study (Supplemental Table S2). In addition, several proteins from the ESX-1 type seven secretion system (T7SS) in *M. tuberculosis* including EspR, EccA, CFP10, EspI, EspL, EspB were amongst the identified phosphorylated proteins (Supplemental Table S1). We also identified virulence factors such Pks15, AceA5, FadD5, EsxB, KatG, GlpX, Rv2032, and PtbB that were phosphorylated in this hyper-virulent *M. tuberculosis* strain (Supplemental Table S2).

Of the 21 phosphorylated proteins involved in information pathways, we identified 6 phosphorylated ribosomal proteins; two phosphorylated small subunit (30S) ribosomal proteins (S3, S19), and four large subunit (50S) ribosomal proteins (L3, L24, L29, L31) with a total of 8 S/T phosphorylation sites. In addition, phosphorylated sites on the ribosomal proteins RpsS and RplX were also identified (Table 4).

**Table 4:** **List of phosphorylated ribosomal proteins identified in this study and other bacteria**.

**Protein name**	**Protein name**	**Phosphopeptides**	**Phospho-residue**	**References**
*rpsC*	*Streptomyces coelicolor*	Not known	Not known	Mikulík et al., 2011
	*M. tuberculosis*	NPES^*^QAQLVAQGVAEQLSNR	S	This study
		AAGGEEAAPDAAAPVEAQSTES^*^	S	This study
*rpsS*	*M. tuberculosis*	HVPVFVTES^*^MVGHK	S	This study
*rplC*	*Streptomyces coelicolor*	Not known	Not known	Mikulík et al., 2011
	*Streptococcus pneumonia*	Not known	Not known	Zhang et al., 2000
	*Halobacterium salinarum*	Not known	Not known	Aivaliotis et al., 2009
	*M. tuberculosis*	IVVEVCSQCHPFYT^*^GK	T	This study
*rplX*	*M. tuberculosis*	S^*^GGIVTQEAPIHVSNVMVVDSDGKPTR	S	This study
*rpmC*	*Streptococcus agalatiae*	FQAAAGQLEKT^*^AR	T	Burnside et al., 2011
	*Streptomyces coelicolor*	RERELGIET^*^VESA	T	Manteca et al., 2011
	*Listeria monocytogenes*	FQLATGQLENT^*^AR	T	Misra et al., 2011
		DLSTTEIQDQEK	Not known	Misra et al., 2011
	*Lactococcus lactis*	MKLSETK	Not known	Soufi et al., 2008
	*M. tuberculosis*	ELGLATGPDGKES^*^	S	This study
*rpmE*	*Klebsiella pneumonia*	S^*^TVGHDLNLDVCGK	S	Lin et al., 2009
	*Halobacterium salinarum*	ASSEFDDRFVTVPLRDVTK	Not known	Aivaliotis et al., 2009
	*M. tuberculosis*	T^*^GGLVMVR	T	This study

* Novel pY site identified in this study.

## Discussion

Here we report 214 phosphorylated proteins extracted during early-logarithmic growth phase from a hyper-virulent clinical Beijing genotype *Mycobacterium tuberculosis* isolate. These proteins can be categorized into different biological functions according to Tuberculist (Lew et al., 2011). The impact of phosphorylation on these Hank's type Ser/Thr kinases (STPKs), phosphatases and their substrates, and the functional role of phosphorylated residues still remains to be elucidated. However, as in previous studies, the identification of phosphorylated residues clearly suggests functional importance.

### Regulatory proteins

In recent years, bacterial S/T/Y kinases and phosphatases have been extensively investigated, with indications that they might play a crucial role in pathogenicity. These Hank's type kinases have the ability to short-circuit the host defense mechanism since they are mostly involved in key biological processes. We identified phosphopeptides from 9 of the 11 STPKs encoded by the *M. tuberculosis* genome. This included both previously described S/T phosphorylation sites (Boitel et al., 2003; Young et al., 2003; Durán et al., 2005; Villarino et al., 2005; Molle et al., 2006; O'Hare et al., 2008; Prisic et al., 2010; Sajid et al., 2011; Chou et al., 2012; Kusebauch et al., 2014) and novel sites on these STPKs thereby highlighting the complexity of the signal transduction mechanism of this pathogen. Phosphorylated peptides were not detected for PknI and PknK. However MS/MS spectra for these peptides for these proteins were detected with mass spectrometry thereby confirming the presence of these proteins (data not shown).

Of the phosphorylated STPKs, PknA, PknB, and PknG have been shown to be essential for *in vitro* growth (Sassetti et al., 2003) and to regulate cell growth and cell division and interfere with host signaling pathways (Fernandez et al., 2006). PknE, PknH, PknJ, and PknL have been implicated in the adaptation to the extracellular environment or intracellular survival of *M. tuberculosis* (Sharma et al., 2006; Lakshminarayan, 2009; Arora et al., 2010; Parandhaman et al., 2014) which is in agreement with reports that during early growth the bacilli undergo a period of adaptation to their external environment (Stock et al., 1989; Soares et al., 2013). PknE is involved in the suppression of apoptosis during nitrate stress (Kumar and Narayanan, 2012) and intracellular survival and adaptation to hostile environments (Parandhaman et al., 2014). In *M. tuberculosis*, PknH controls the expression of a variety of cell wall related enzymes and regulates *in vivo* growth in mice (Zheng et al., 2007). PknJ undergoes autophosphorylation and phosphorylates the Thr^168^, Thr^171^, and Thr^173^ residues of Embr (a transcriptional regulator), MmaA4/Hma (a methyltransferase involved in mycolic acid biosynthesis) and PepE (a peptidase located adjacent to the *pknJ* gene in the *M. tuberculosis* genome), respectively (Jang et al., 2010). Lastly, PknL is involved in an adaptive response to nutrient starvation. This kinase regulates transcription which allows the bacilli to maintain metabolic activity without sourcing energy from elsewhere (Lakshminarayan, 2009). Furthermore, we identified a number of STPK substrates that were phosphorylated in clinical hyper-virulent *M. tuberculosis* strain (list not shown) thereby highlighting the complexity of the phosphorylation regulatory network in *M. tuberculosis*. Even though the role of STPKs in bacterial physiology is not yet fully understood the data presented here could underpin a targeted approach to improving our understanding of STPK-mediated signal transduction mechanisms in *M. tuberculosis*.

### Tyrosine phosphorylation

The *M. tuberculosis* genome encodes for two putative tyrosine phosphatases (PtpA and PtpB) but is not predicted to encode tyrosine kinases (Cole et al., 1998; Bach et al., 2009). Most bacterial phosphorylation sites are on serine and threonine; a survey of 11 bacterial phosphoproteomes revealed that S/T phosphorylation accounted for an average of 48 and 40% of phosphorylated sites, respectively, while tyrosine phosphorylation events account for less than 10% of the overall phosphoproteome (Ge and Shan, 2011). Tyrosine phosphorylated proteins have been previously shown to play important regulatory roles through their involvement in biological functions such as exopolysaccharide production, DNA metabolism, stress responses (Ge et al., 2011; Whitmore and Lamont, 2012). Recently, Kusebauch et al. identified tyrosine phosphorylated proteins in *M. tuberculosis* and demonstrated that a number of STPKs can phosphorylate tyrosine in either *cis* or *trans* (Kusebauch et al., 2014). This suggests that STPKs have the ability to phosphorylate S/T/Y. In this study we identified 13 tyrosine phosphorylation sites in 8 proteins (Table 3). An overlap of three proteins (FhaA, MmpS3, and GroES) and 6 tyrosine phosphorylated sites we similar between this and the previous study (Kusebauch et al., 2014). Five of the tyrosine phosphorylated proteins (FhaA, GroEL2, MmpS3, GroES, and Ppa) identified in this study are essential for *in vitro* growth (Sassetti et al., 2003; Griffin et al., 2011) and involved in a variety of functions.

This study confirmed and expanded work by Kusebauch et al., where multiple tyrosine phosphopeptides were identified for FhaA. FhaA is a regulatory protein which has been implicated in cell wall biosynthesis (Fernandez et al., 2006) and has a strong association with PknA and PknB (Pallen et al., 2002; Roumestand et al., 2011). We found that the highly S/T/Y phosphorylated FHA-domain contained 6 tyrosine phosphopeptides of which 4 were previously been identified in H_37_Rv (Kusebauch et al., 2014). FhaA is a major substrate of PknB and has been implicated in the formation of a regulatory complex with MviN required for peptidoglycan biosynthesis (Warner and Mizrahi, 2012). In our dataset, we found that all three of the proteins (PknB, FhaA, and MviN) in the regulator complex were phosphorylated.

We also confirm the presence of a previously reported Y phosphopeptide of MmpS3 and identified a second phosphopeptide. MmpS3 forms part of the mycobacterial membrane protein small family and is an essential protein for mycobacterial growth and cholesterol metabolism (Griffin et al., 2012). The role of phosphorylation of this protein has yet to be determined.

The two proteins GroEL2 and GroES have been identified as potential candidates for antituberculosis treatment (Al-Attiyah et al., 2006). We confirmed the presence of the Y phosphopeptide in GroEL2 identified in H_37_Rv (Kusebauch et al., 2014). Recently it has been shown that the antigen GroES is sufficient to protect BALB/c mice against challenge infection (Lima et al., 2003) and up-regulated in kanamycin and amikacin resistant isolates (Kumar et al., 2013). Ppa is an inorganic pyrophosphate and is involved in macromolecule biosynthesis. The *M. tuberculosis* Ppa is highly similar to a well conserved homolog of *Legionella. pneumophila* PPase which is induced in macrophages, although the *M. tuberculosis* PPa promotor is not responsive to any specific intracellular triggers (Triccas and Gicquel, 2001).

### Virulence factors

The identification of virulence factors is crucial in order to improve our understanding of the mechanisms involved in pathogenesis of *M. tuberculosis*. Several of the phosphorylated virulence factors identified in this study were found to be involved in basic metabolic pathways such a lipid and fatty acid metabolism, secretion systems and response and adaptation to environmental changes. The virulence factor KasB and key enzymes (FadD32, AccD4, and MmaA3) in the mycolic acids biosynthesis pathway were phosphorylated in this hyper-virulent *M. tuberculosis* strain. The *kasB* gene is not essential for growth, however, the deletion mutant, Δ*kasB*, resulted in an alteration in growth morphology and loss of acid-fast staining (Bhatt et al., 2007). This suggests that modification of this protein could influence the synthesis of mycolic acids and thereby the pathogenicity of the bacilli.

The specialized ESX-1 Type VII secretion system (T7SS), unique to pathogenic mycobacteria is responsible for the secretion of two culture filtrate proteins EsxA and EsxB (ESAT-6 and CFP-10). These secretion systems have been shown to be involved in virulence and are critical for intracellular survival (Bitter and Kuijl, 2014) due to their ability to secrete proteins that lack classical signal peptides across the complex cell envelope to host cells during infection (Houben et al., 2014, p. 5). *M. tuberculosis* have several different ESX regions (ESX-1 to ESX-5) (Daleke et al., 2012) with varying gene numbers and size for each of these secretion machinery. In this study we found 6 T7SS proteins to be phosphorylated in the hyper-virulent strain. In a previous study, proteomics of whole cell extracts of this hyper-virulent *M. tuberculosis* strain revealed an under-representation of virulence factors such as ESAT-6 and Esx-like proteins (de Souza et al., 2010). The authors showed the abundance of ESAT-6 gene expression was reduced in the hyper-virulent *M. tuberculosis* suggesting that the low levels of this protein might be as a result of its ability to export these proteins more efficiently into the extracellular environment (de Souza et al., 2010).

### Protein synthesis and interactions

The impact of phosphorylation on the functionality of ribosomal proteins is not fully understood. Mikulik et al. hypothesized that phosphorylation of ribosomal proteins induces or stabilizes conformational changes during proteins synthesis which could allow modification of subunit association or changes in interactions with proteins and RNAs (Mikulík et al., 2011). According to the protein phosphorylation database, phosphopeptides of RpmC have been identified in four different bacteria (Soufi et al., 2008; Burnside et al., 2011; Manteca et al., 2011; Misra et al., 2011). The implication of phosphorylation on RpmC has not been investigated. However, in *E.coli*, RpmC, RplW and Trigger factor are located at the exit tunnel in the ribosome, suggesting that phosphorylation may impact on multiple stages of transcription (Kramer et al., 2002). In our study we identified phosphopeptides for 7 ribosomal proteins. We also identified unique phosphopeptides on ribosomal proteins RpsS and RplX (Table 3).

### Overlap of phosphorylated proteins with other bacteria

Twenty-five of phosphorylated proteins identified in our study were also identified in phosphoproteomics studies of other bacteria such as *Klebsiella pneumonia* (Lin et al., 2009), *Helicobacter pylori* (Ge et al., 2011), *Steptococcus pneumonia* (Sun et al., 2010), *Bacillus subtillis* (Macek et al., 2007), *Halobacterium salinarum* (Aivaliotis et al., 2009), etc. (Table 4). In our dataset the distribution of S/T/Y seem to be bias toward pT and is in accordance with previously described phosphoproteomes of *M. tuberculosis* (Prisic et al., 2010; Kusebauch et al., 2014). Manual evaluation of the genome found an over-representation of Threonine relative to Serine (52:48%). This compared to other bacteria such as *Acinetobacter baumannii* (Soares et al., 2014), *Bacillus subtilis* (Macek et al., 2007), *Escherichia coli* (Macek et al., 2008; Soares et al., 2013) and *Halobacterium salinarum* (Aivaliotis et al., 2009), *Pseudomonas aeruginosa* (Ravichandran et al., 2009), and *Streptomyces coelicolor* (Parker et al., 2010) which demonstrate a bias toward pS.

Forty-five of the phosphorylated proteins identified in our study were previously described for *M. tuberculosis* H_37_Rv (Boitel et al., 2003; Young et al., 2003; Molle et al., 2004, 2006; Durán et al., 2005; Kang et al., 2005; Villarino et al., 2005; O'Hare et al., 2008; Thakur et al., 2008; Prisic et al., 2010; Sajid et al., 2011; Gee et al., 2012). The reason for not identifying all of the previously identified phosphorylated proteins in the protein phosphorylation database could be ascribed to different genetic backgrounds of the analyzed *M. tuberculosis* strains, culture conditions, sample preparation and different MS-based proteomics approaches used in each of the studies. Our analysis was performed on a hyper-virulent clinical isolate of *M. tuberculosis* and a member of the Beijing genotype which is genetically distinct from the laboratory strain *M. tuberculosis* H_37_Rv analyzed by Prisic et al. and Kusebauch et al. In addition, the Prisic et al. study reported on the combined phosphoproteome from 6 different conditions (5 different culture conditions and 2 different growth phases) (Prisic et al., 2010) while Kusebauch et al. reported on the phosphoproteome of late-logarithmic phase cultures (Kusebauch et al., 2014), whereas our study analyzed early-logarithmic phase cultures. Even though the overlap between our study of clinical *M. tuberculosis* and that of the previously described laboratory *M. tuberculosis* H_37_Rv is low this work substantially extends our knowledge of the *M. tuberculosis* phosphoproteome. During logarithmic growth phase of bacterial growth the cells are adapting to the environment of the growth media and biological process such as RNA synthesis, DNA replication and synthesis of micro- and macromolecules are up-regulated. It is important to note that in this study the whole cell lysate proteins were enriched for phosphopeptides and we detected a number of phosphorylated proteins involved in these biological processes such as fatty acid- and lipid biosynthetic metabolism; RNA modification and translation; DNA repair, replication and modification. It is believed that environmental conditions, cell density and growth phase influence the expression of virulence factors by a pathogen (McIver et al., 1995). This is consistent other bacterial phosphoproteomes, thereby emphasizing that S/T/Y phosphorylation is an important process required for the regulation of numerous cellular processes.

## Conclusion

Recent developments in the methodology and mass spectrometry technology for phosphoproteomics have highlighted the need to explore the involvement of phosphorylation in disease development and progression. However, the impact of the protein phosphorylation cascade on the physiology of pathogenic bacteria such as *M. tuberculosis* has yet to be fully elucidated. Improved preparative techniques and more sensitive instrumentation are required to fully appreciate the complexity of protein modification. This can only be achieved if concomitant methods are developed to elucidate the impact of phosphorylation on protein function. Although this qualitative study was done in clinical hyper-virulent *M. tuberculosis*, without any follow-up validation studies it still provides a valuable resource for further investigating and understanding the impact of protein phosphorylation regulation in *M. tuberculosis*.

### Conflict of interest statement

The authors declare that the research was conducted in the absence of any commercial or financial relationships that could be construed as a potential conflict of interest.
