# Supplementary material for: Phosphoproteomics analysis of a clinical Mycobacterium tuberculosis Beijing isolate: expanding the mycobacterial phosphoproteome catalog
Source: Front Microbiol. 2015 Feb 10;6:6. doi: 10.3389/fmicb.2015.00006 (PMC4322841; doi:10.3389/fmicb.2015.00006)
Supplement: Supplementary file 2 [file DataSheet1.DOCX]

***Supplementary Material***

**Phosphoproteomics analysis of a clinical *Mycobacterium***

***tuberculosis* Beijing isolate: expanding the mycobacterial**

**phosphoproteome catalog**

Suereta Fortuin^1^, Gisele Tomazella^2^, Nagarjuna Nagaraj*^3^*, Samantha Leigh Sampson^1^, Nicolaas Gey van Pittius^1^, Nelson Cruz Soares^4^, Harald Wiker^2#^, Gustavo Antonio de Souza^5#^, Robin Mark Warren^1#*^

*^1^Division of Molecular Biology and Human Genetics, Faculty Medicine and Health Sciences, Stellenbosch University, Cape Town, South Africa, DST/NRF Centre of Excellence for Biomedical Tuberculosis Research, SAMRC Centre for Tuberculosis Research.*

*^2^The Gade Research Group for Infection and Immunity, Department of Clinical Science,* *University of Bergen, Bergen,*

*^3^Max Planck Institute of Biochemistry, Munich, Germany*

*^4^Institute of Infectious Disease and Molecular Medicine (IIDMM), University of Cape Town, Faculty of Health Sciences, Anzio, Observatory, Cape Town, South Africa*

*^5^Norway* *Proteomics Core Facility, Department of Immunology, Oslo University, Norway*

**^#^ contributed equally**

1. **Supplementary Data**

**Supplementary data Table S1**. Excel Sheet containing phosphoproteomics data for hyper-virulent *M. tuberculosis* Beijing strain.

**Supplementary data Table S1**. Excel Sheet containing high confidence phosphosites for hyper-virulent *M. tuberculosis* Beijing strain

1. **Supplementary Figures and Tables**

**Supplementary Figures S1**. Identification of phosphorylated residues by mass spectrometry. Fragmentation spectra for the all high confidence Ser/Thr/Tyr phosphosites identified for the hyper-virulent M. tuberculosis Beijing strain.

**Supplementary Figures S2** (Continue from **Supplementary Figures S1**. Identification of phosphorylated residues by mass spectrometry. Fragmentation spectra for the all high confidence Ser/Thr/Tyr phosphosites identified for the hyper-virulent M. tuberculosis Beijing strain.)
