# Supplementary material for: Phosphoproteomics analysis of a clinical Mycobacterium tuberculosis Beijing isolate: expanding the mycobacterial phosphoproteome catalog
Source: Front Microbiol. 2015 Feb 10;6:6. doi: 10.3389/fmicb.2015.00006 (PMC4322841; doi:10.3389/fmicb.2015.00006)

Raw file	Scan	Method	Score	m/z
20101013_Velos3_NaNa_COLLAB_5527_rep_01_flowthru_01	7601	FTMS; HCD	71.65	866.4

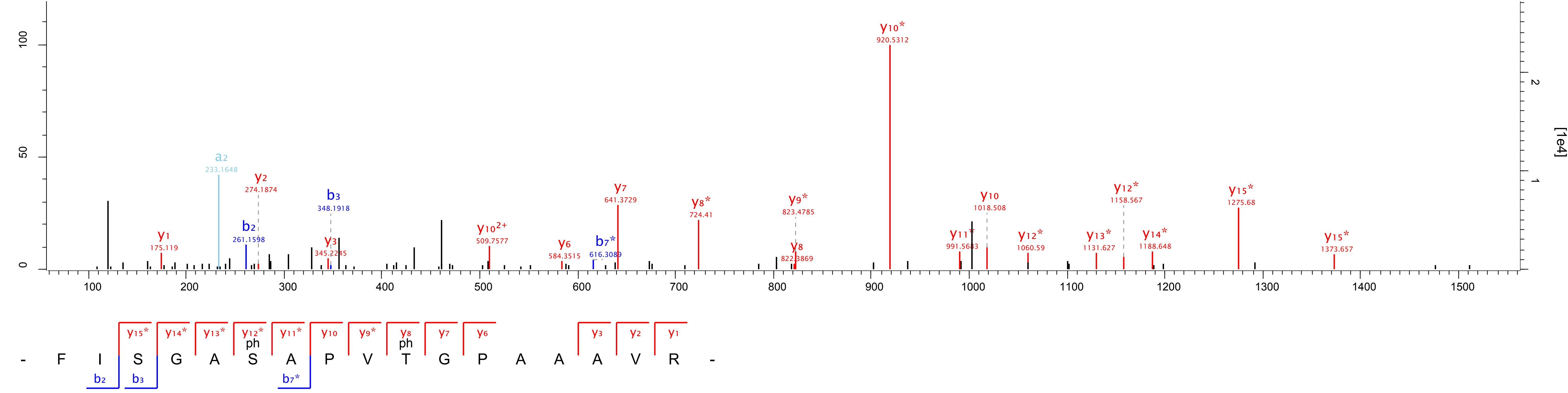

Raw file	Scan	Method	Score	m/z
20101013_Velos3_NaNa_COLLAB_5527_rep_01_fraction02	13894	FTMS; HCD	122.18	670.36

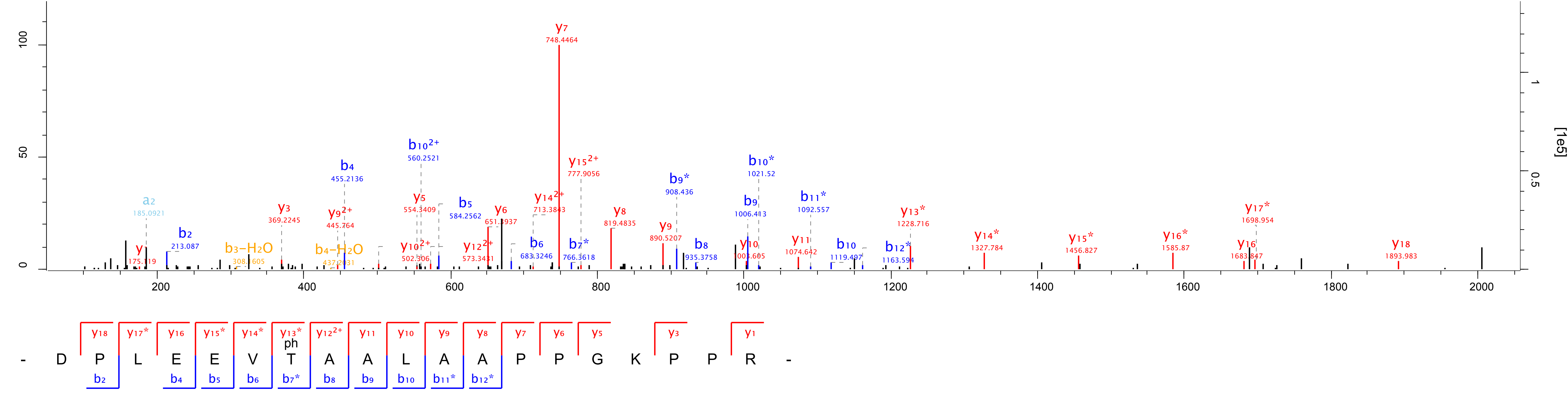

Raw file	Scan	Method	Score	m/z
20101013_Velos3_NaNa_COLLAB_5527_rep_03_fraction01	5334	FTMS; HCD	98.54	940.39

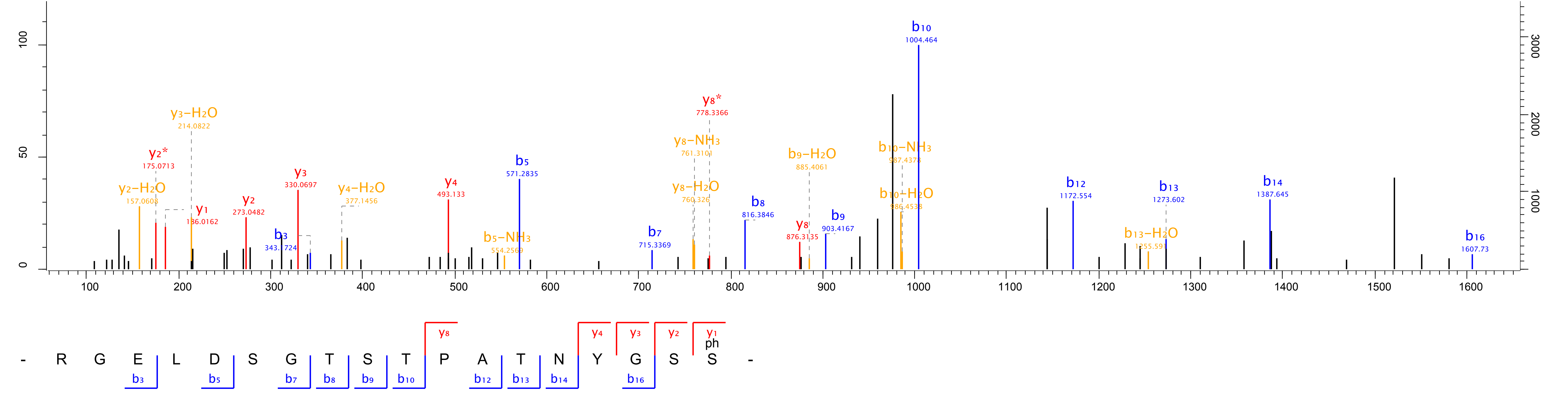

Raw file	Scan	Method	Score	m/z
20101013_Velos3_NaNa_COLLAB_5527_rep_03_fraction04	4024	FTMS; HCD	69.98	554.29

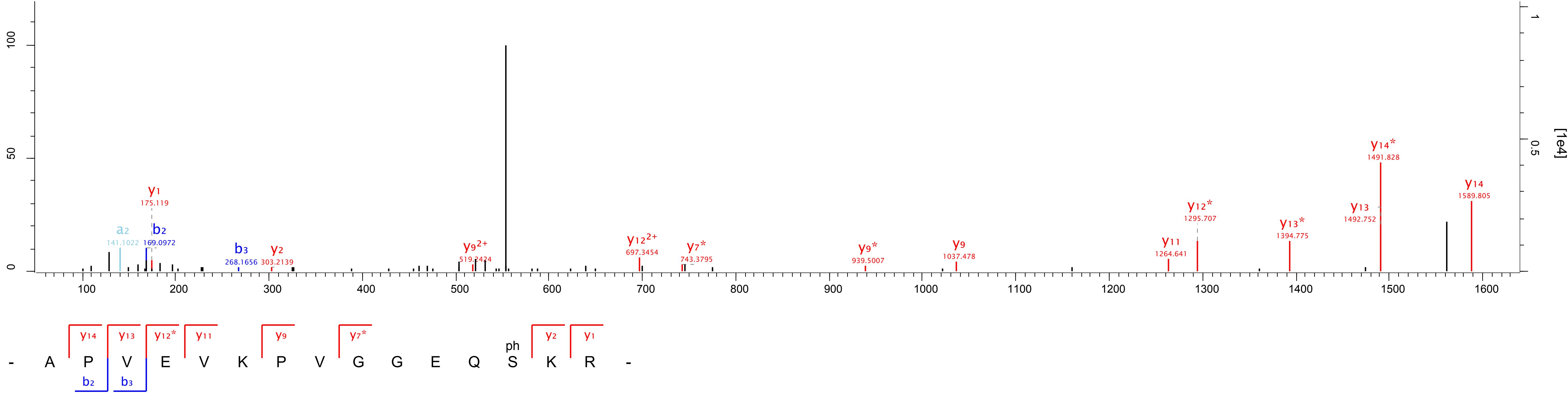

Raw file	Scan	Method	Score	m/z
20101013_Velos3_NaNa_COLLAB_salvage_5527_02	2557	FTMS; HCD	51.09	817.32

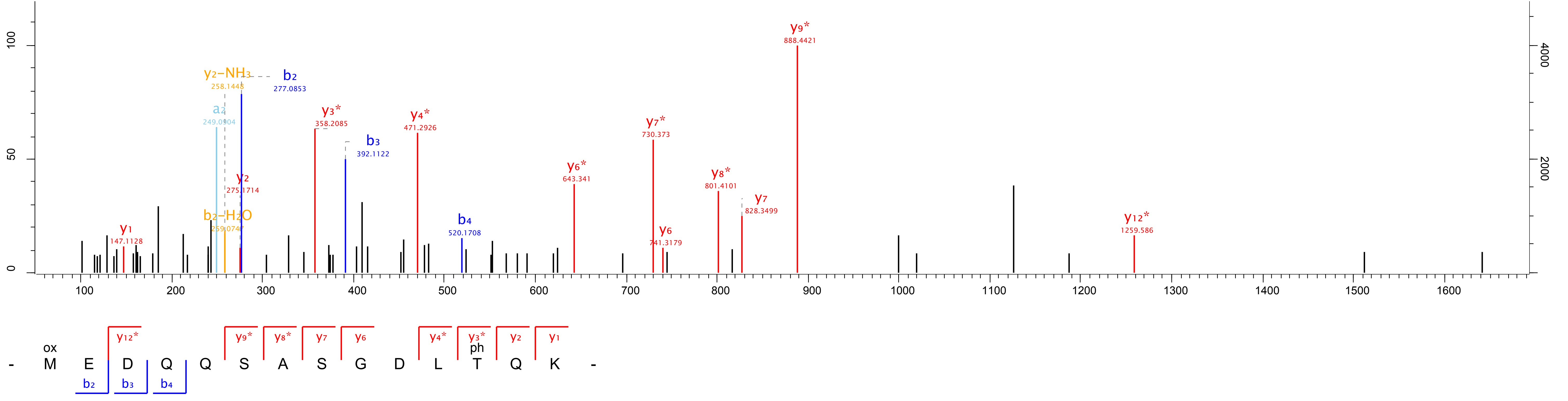

Raw file	Scan	Method	Score	m/z
20101013_Velos3_NaNa_COLLAB_salvage_5527_02	2640	FTMS; HCD	74.14	817.32

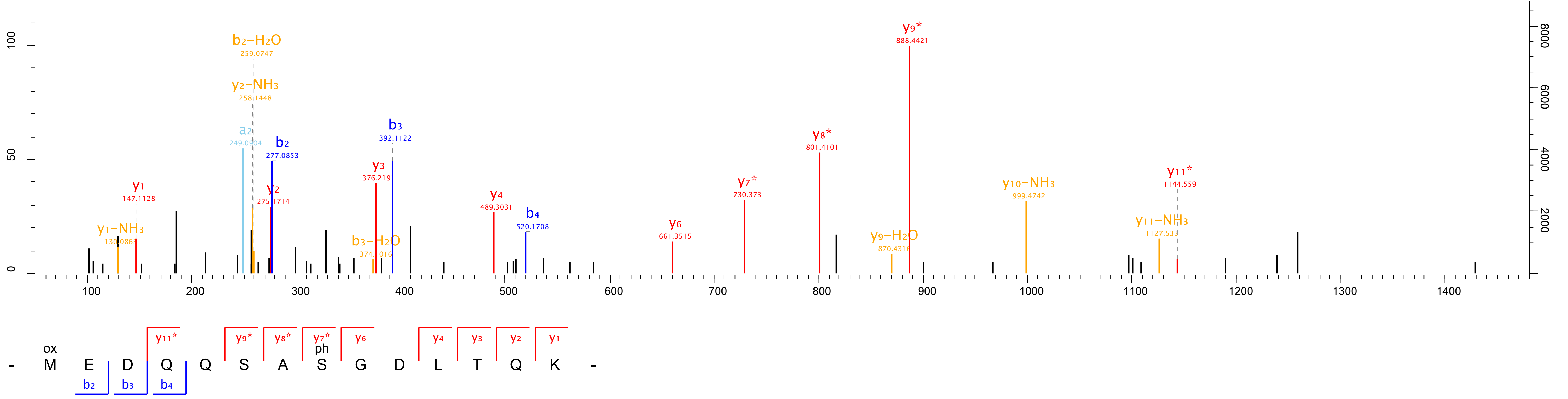

Raw file	Scan	Method	Score	m/z
20101013_Velos3_NaNa_COLLAB_5527_rep_03_fraction05	3803	FTMS; HCD	41.7	355.18

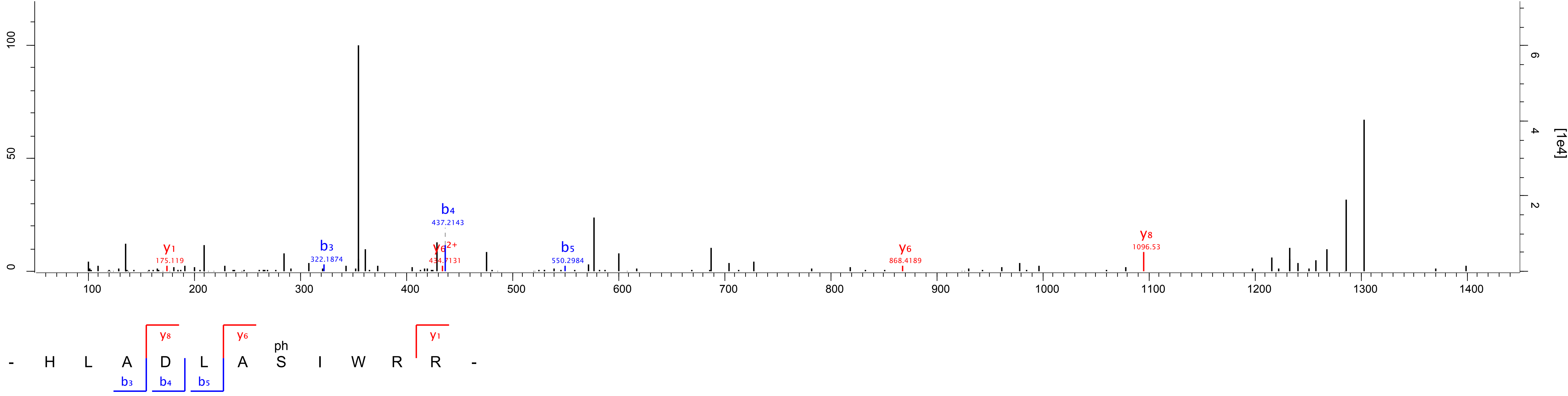

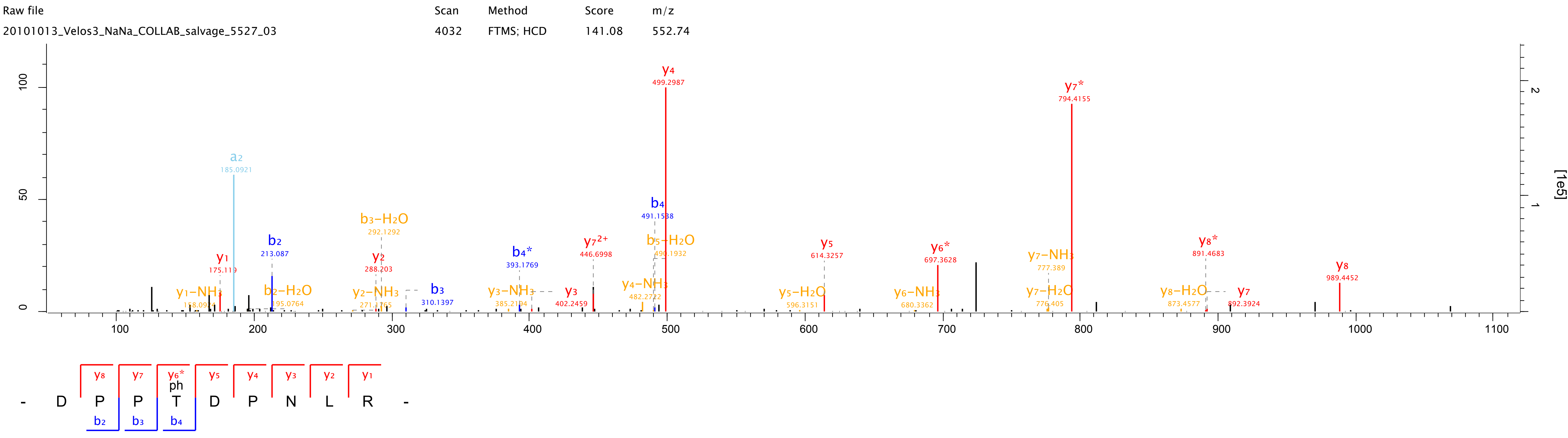
Raw file
20101013_Velos3_NaNa_COLLAB_salvage_5527_03
Scan
4032
Method
FTMS; HCD
Score
141.08
m/z
552.74
[1e5]
y4 499.2987
y7* 794.4155
a2 185.0921
b4 491.1538
b3−H2O 292.1292
b5−H2O 490.1932
b2 213.087
b4* 393.1769
y7 2+ 446.6998
y5 614.3257
y6* 697.3628
y7−NH3 777.389
y8* 891.4683
y1 175.119
y2 288.203
y8 989.4452
y1−NH3 158.0924
b2−H2O 195.0764
y2−NH3 271.1765
b3 310.1397
y3−NH3 385.2194
y3 402.2459
y4−NH3 482.2722
y5−H2O 596.3151
y6−NH3 680.3362
y7−H2O 776.405
y8−H2O 873.4577
y7 892.3924
- D P P T D P N L R -
ph
y8 y7 y6* y5 y4 y3 y2 y1
b2 b3 b4

Raw file	Scan	Method	Score	m/z
20101013_Velos3_NaNa_COLLAB_salvage_5527_03	6706	FTMS; HCD	78.08	485.93

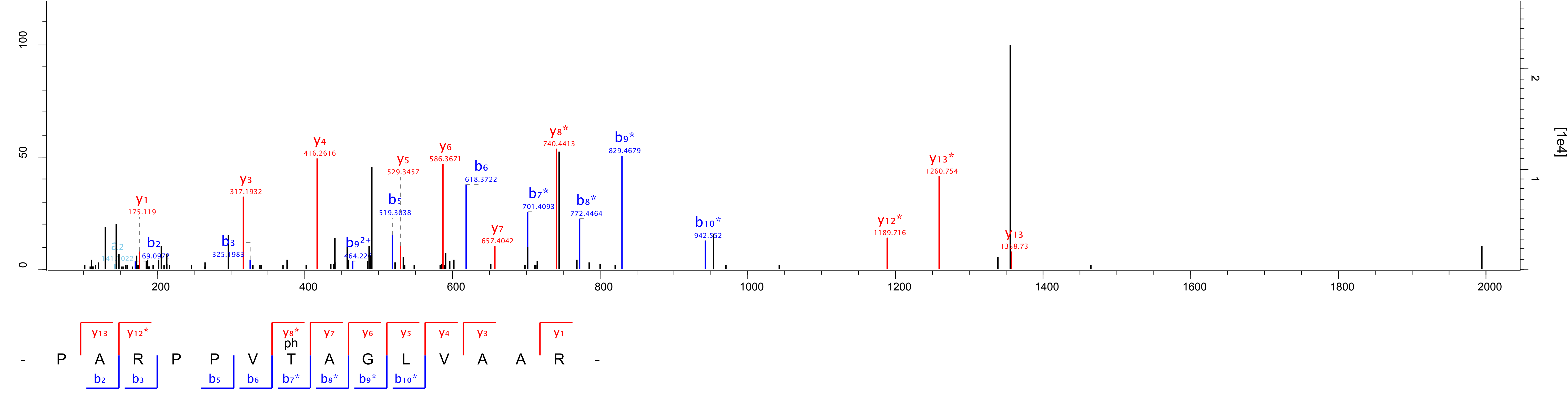

Raw file	Scan	Method	Score	m/z
20101013_Velos3_NaNa_COLLAB_salvage_5527_02	3533	FTMS; HCD	226	586.9

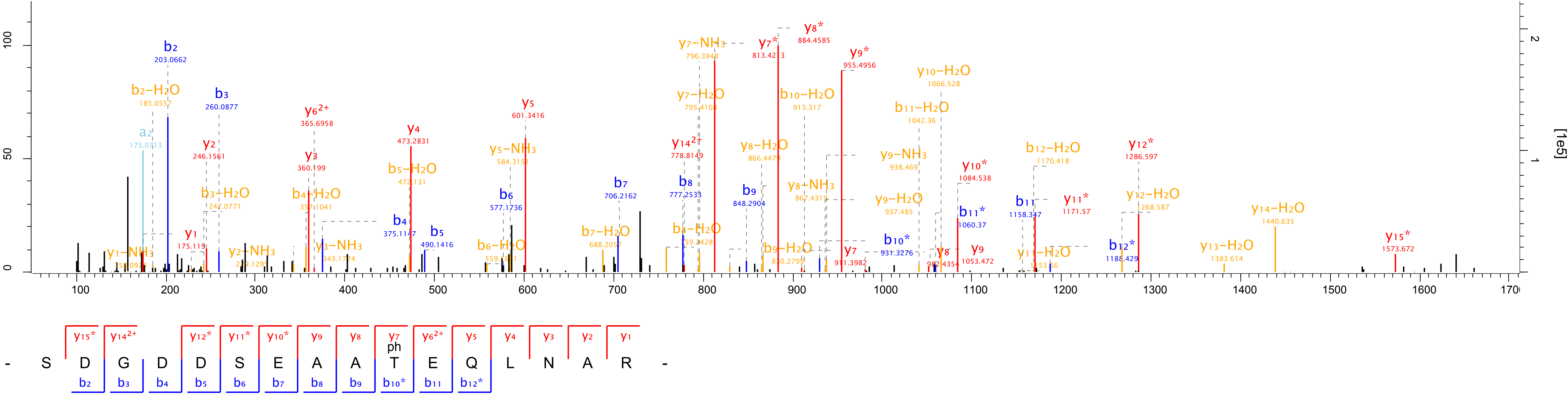

Raw file	Scan	Method	Score	m/z
20101013_Velos3_NaNa_COLLAB_5527_rep_03_flowthru_02	7516	FTMS; HCD	133.84	973.47

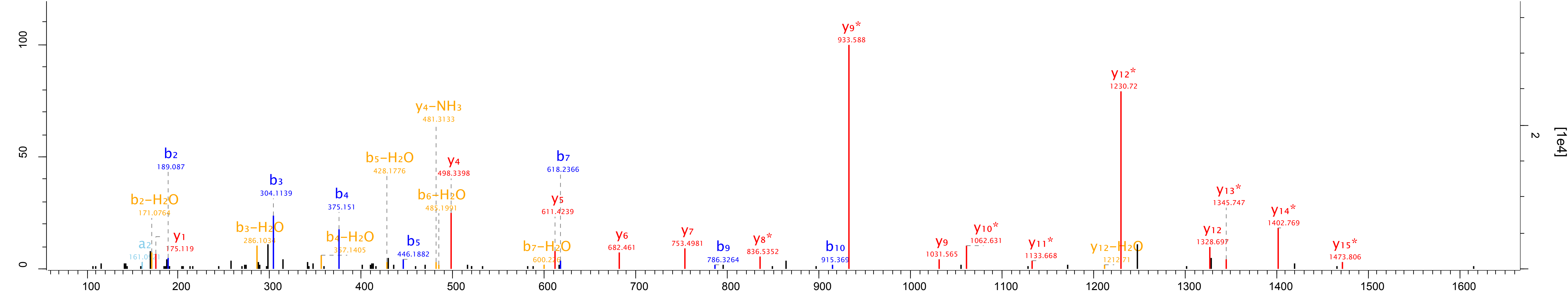

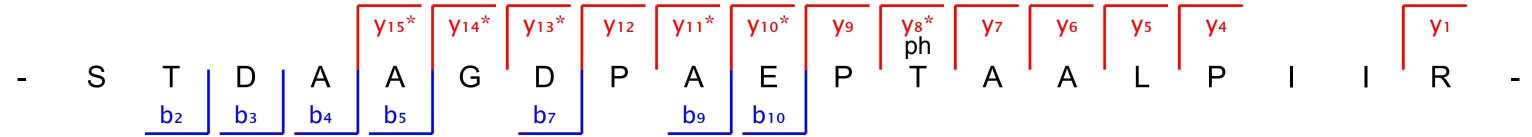

Raw file	Scan	Method	Score	m/z
20101013_Velos3_NaNa_COLLAB_salvage_5527_02	5785	FTMS; HCD	61.96	576.25

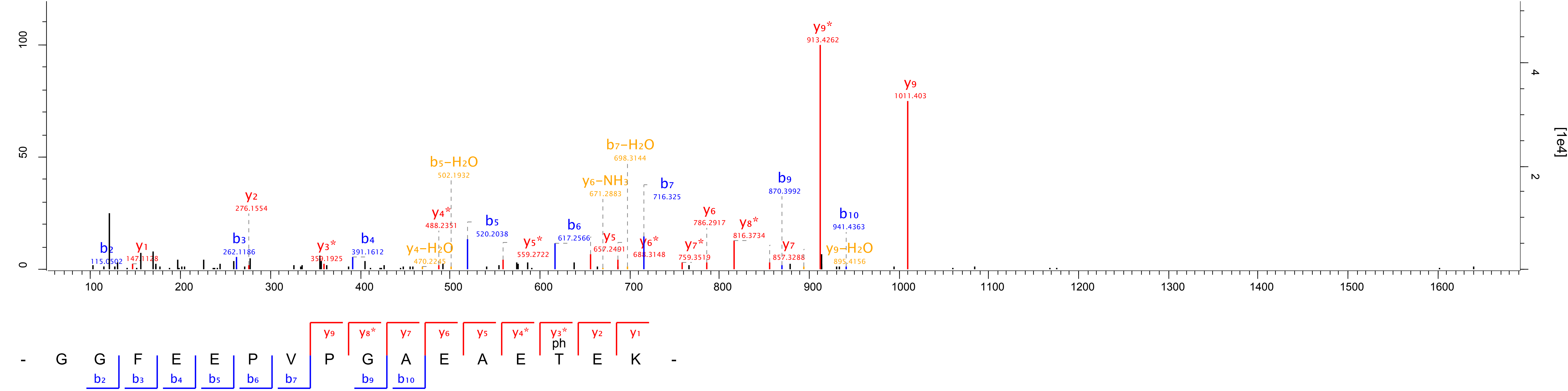

Raw file	Scan	Method	Score	m/z
20101013_Velos3_NaNa_COLLAB_salvage_5527_02	4080	FTMS; HCD	96.23	543.75

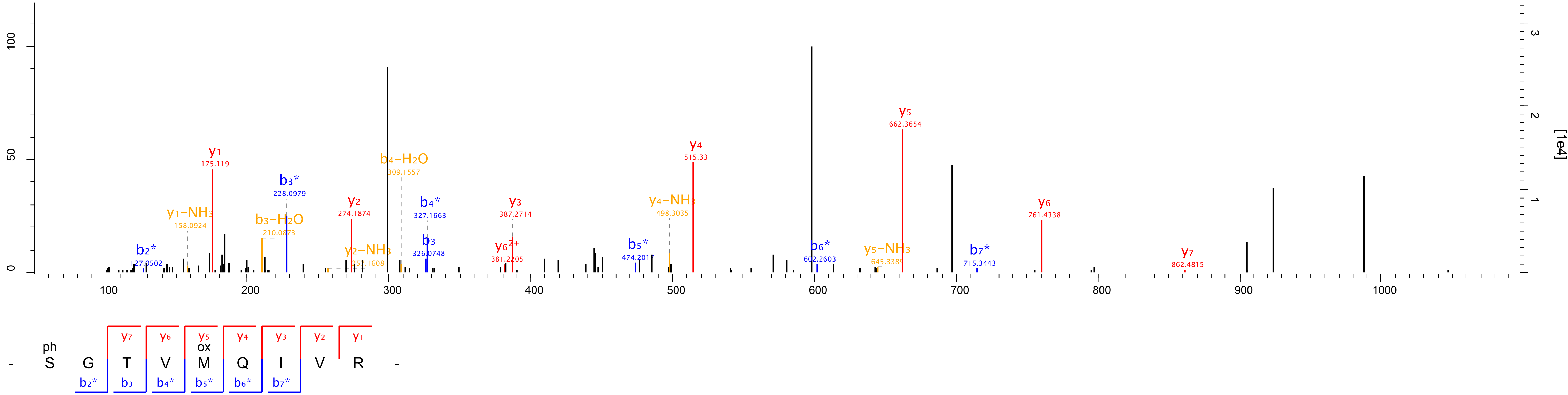

Raw file	Scan	Method	Score	m/z
20101013_Velos3_NaNa_COLLAB_salvage_5527_02	3051	FTMS; HCD	54.28	729.81

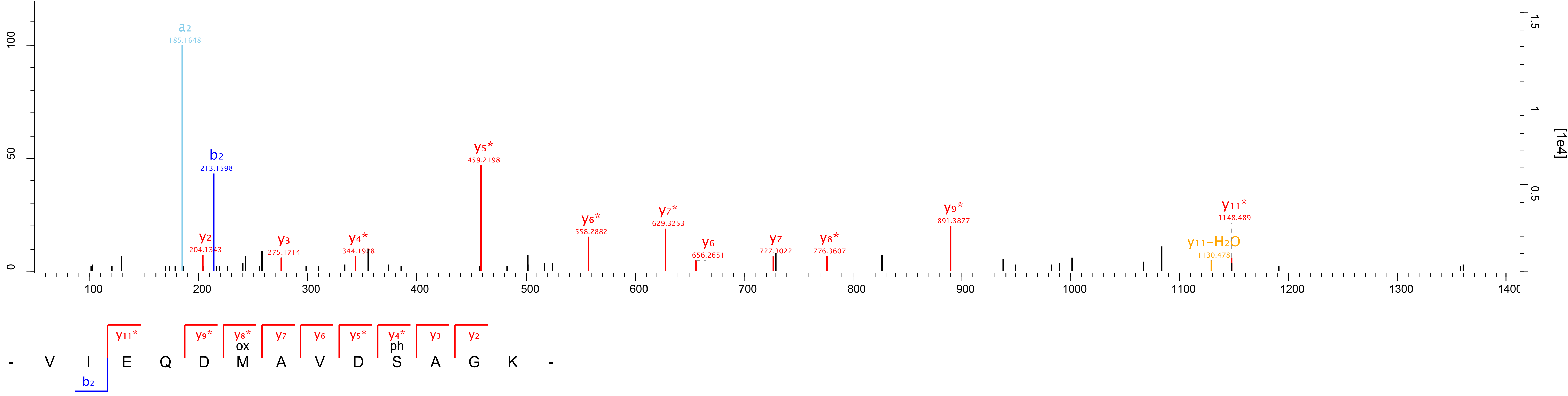

Raw file	Scan	Method	Score	m/z
20101013_Velos3_NaNa_COLLAB_5527_rep_01_flowthru_03	6891	FTMS; HCD	54.1	814.89

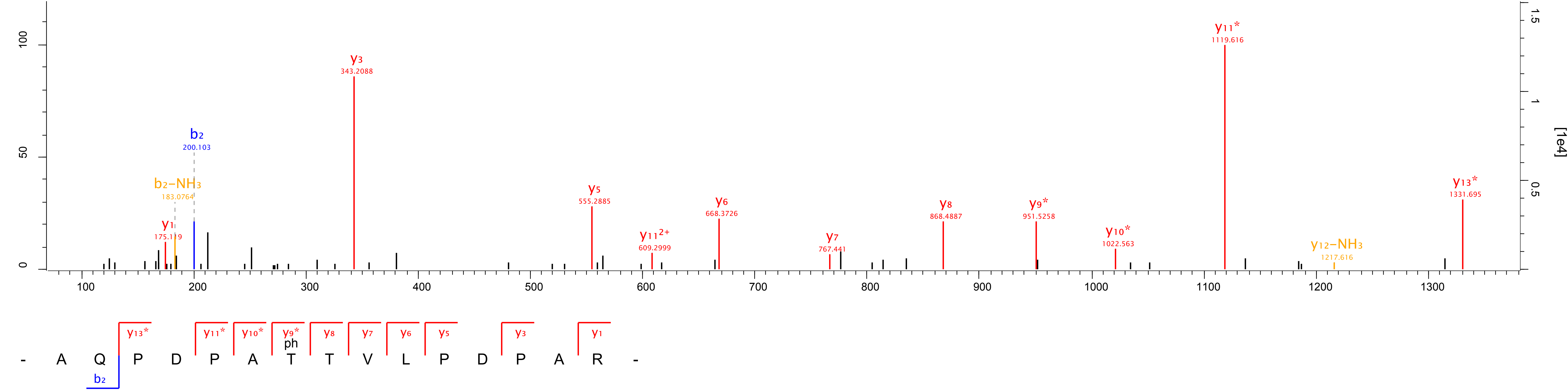

Raw file	Scan	Method	Score	m/z
20101013_Velos3_NaNa_COLLAB_5527_rep_03_flowthru_01	5905	FTMS; HCD	65.04	680.31

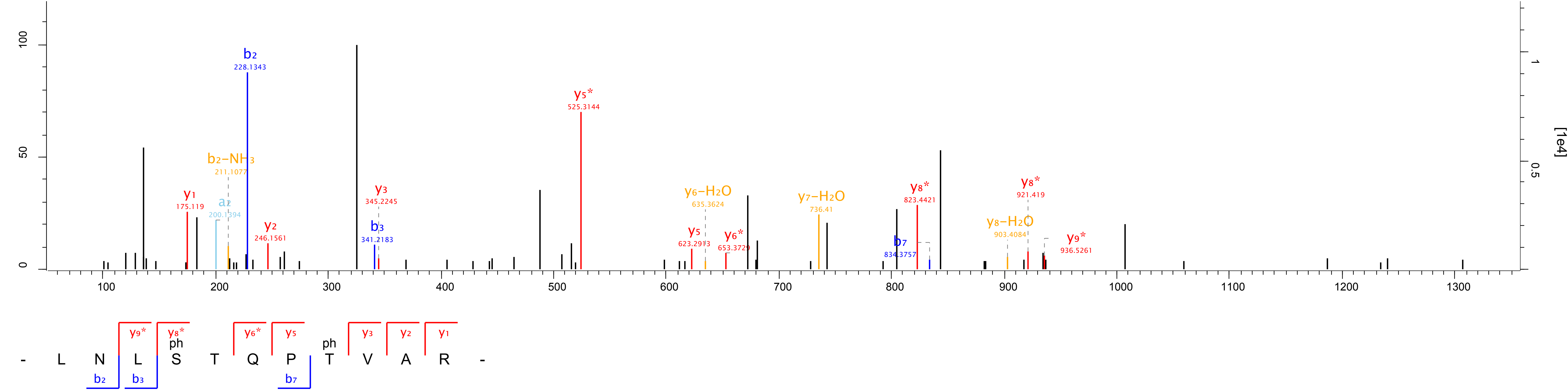

Raw file	Scan	Method	Score	m/z
20101013_Velos3_NaNa_COLLAB_salvage_5527_02	2274	FTMS; HCD	117	745.8

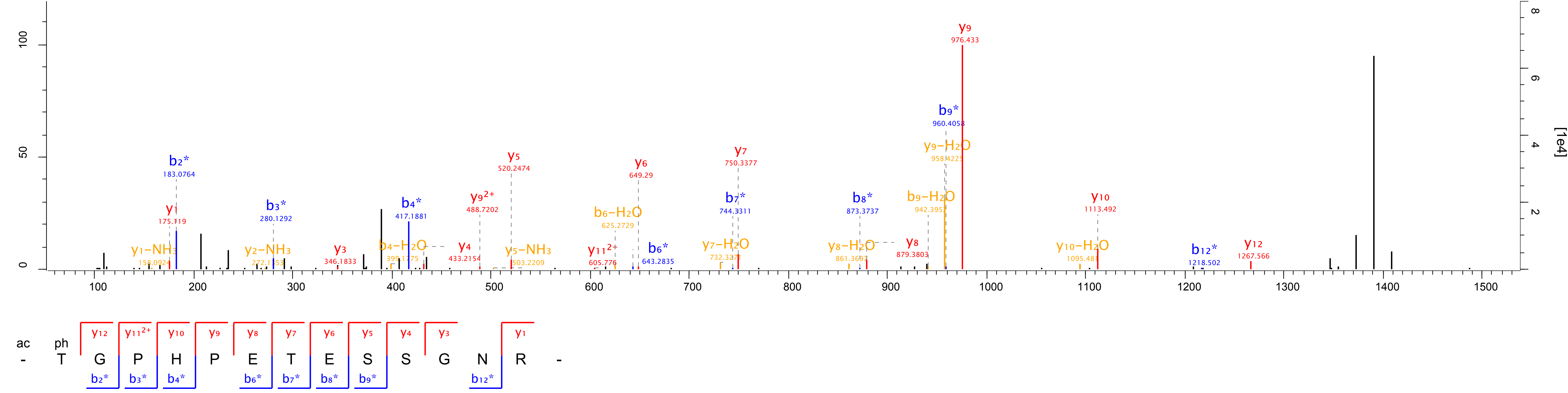

Raw file	Scan	Method	Score	m/z
20101013_Velos3_NaNa_COLLAB_salvage_5527_01	12882	FTMS; HCD	75.54	736.38

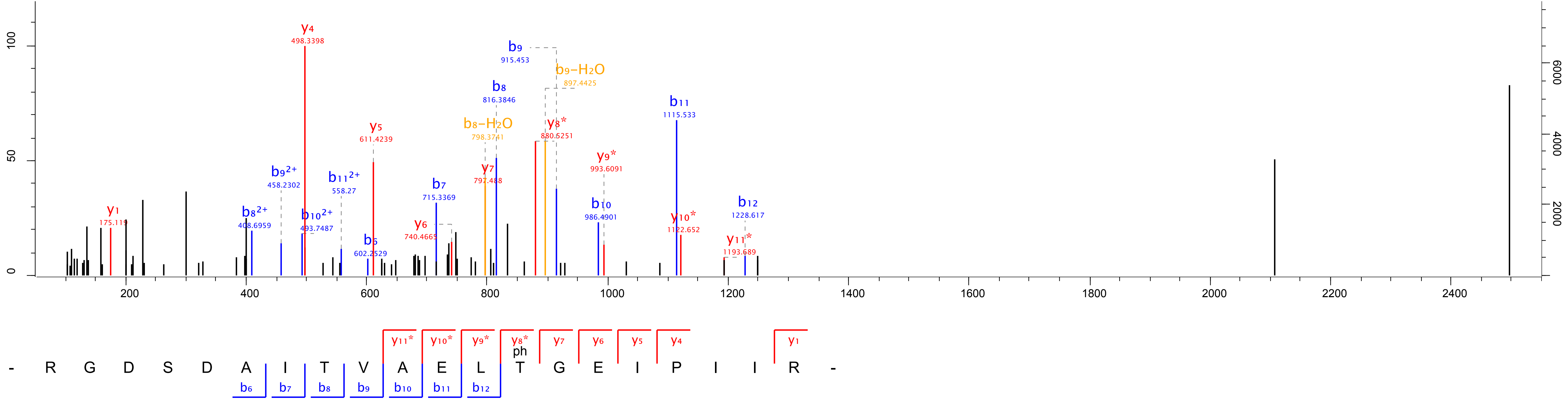

Raw file	Scan	Method	Score	m/z
20101013_Velos3_NaNa_COLLAB_salvage_5527_02	6351	FTMS; HCD	66.39	671.81

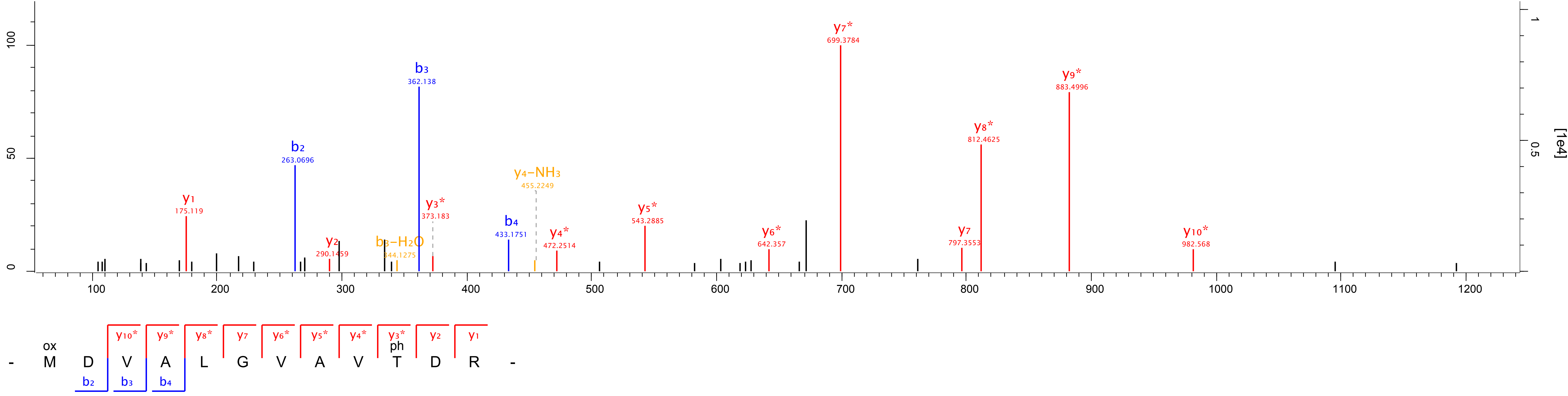

Raw file	Scan	Method	Score	m/z
20101013_Velos3_NaNa_COLLAB_5527_rep_01_flowthru_03	4844	FTMS; HCD	84.3	676.32

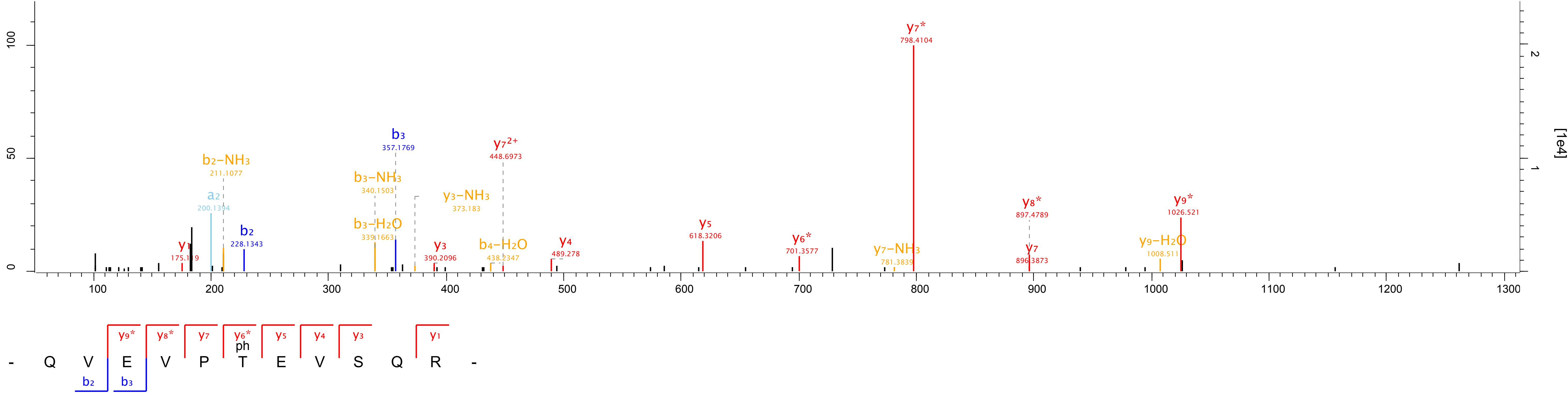

Raw file	Scan	Method	Score	m/z
20101013_Velos3_NaNa_COLLAB_salvage_5527_02	5160	FTMS; HCD	64.04	725.28

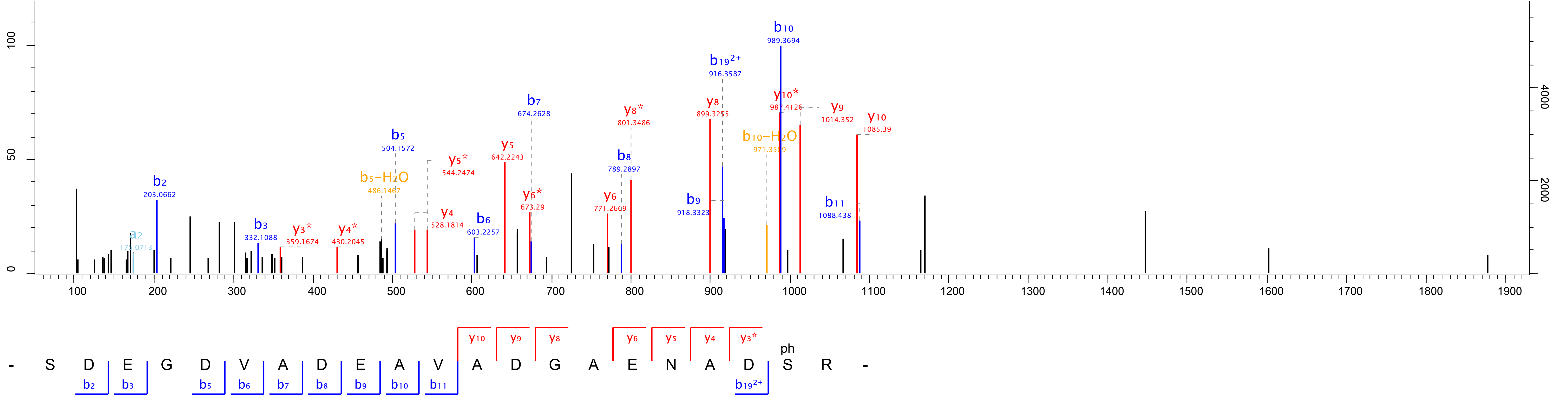

Raw file	Scan	Method	Score	m/z
20101013_Velos3_NaNa_COLLAB_salvage_5527_03	7443	FTMS; HCD	102.07	563.94

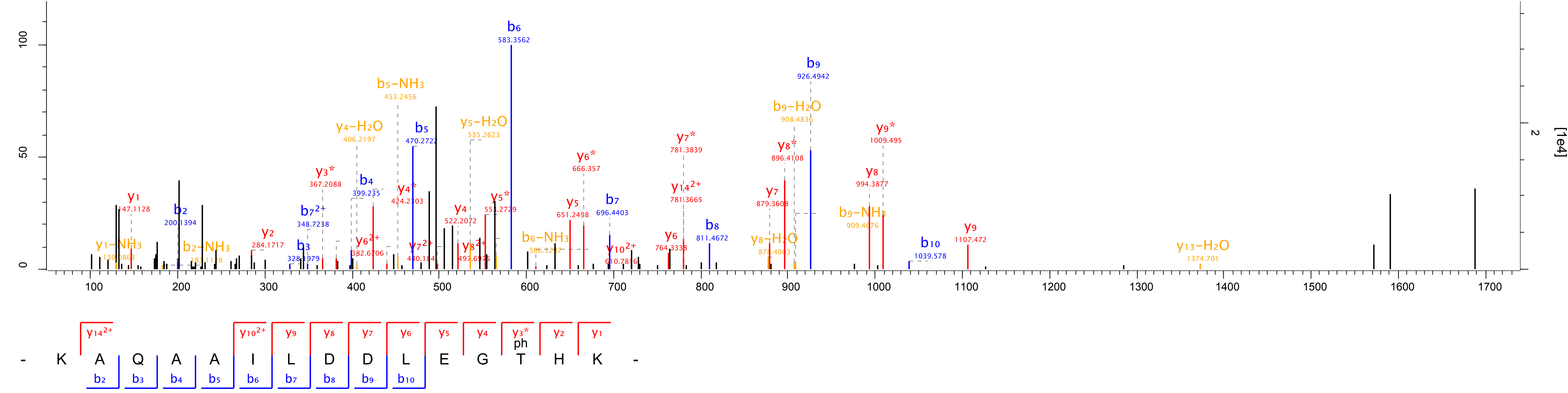

Raw file	Scan	Method	Score	m/z
20101013_Velos3_NaNa_COLLAB_5527_rep_01_flowthru_03	4596	FTMS; HCD	82.45	797.83

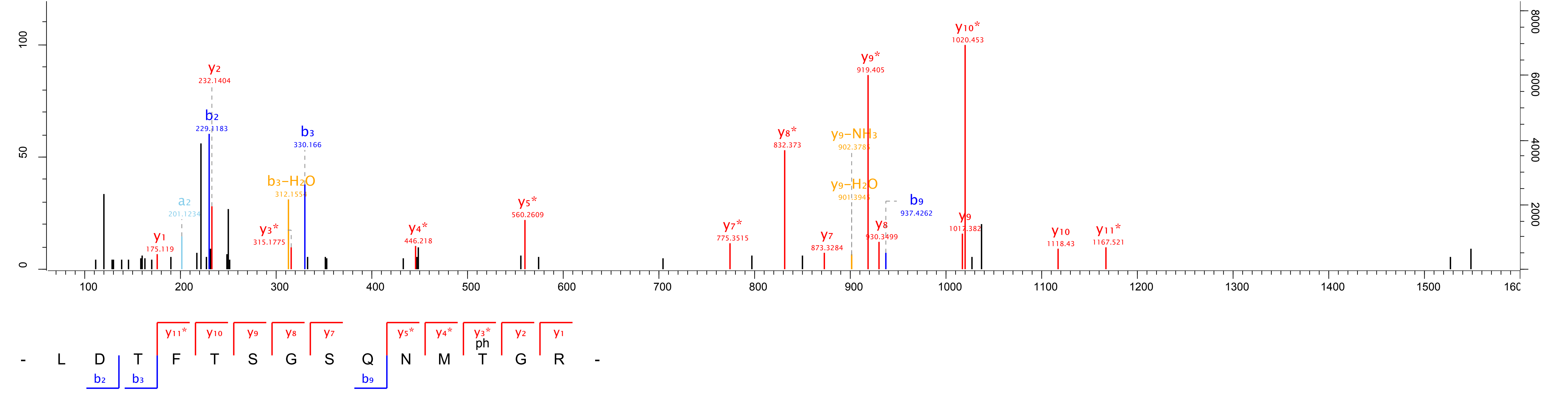

Raw file	Scan	Method	Score	m/z
20101013_Velos3_NaNa_COLLAB_5527_rep_03_flowthru_01	11695	FTMS; HCD	45.44	647.98

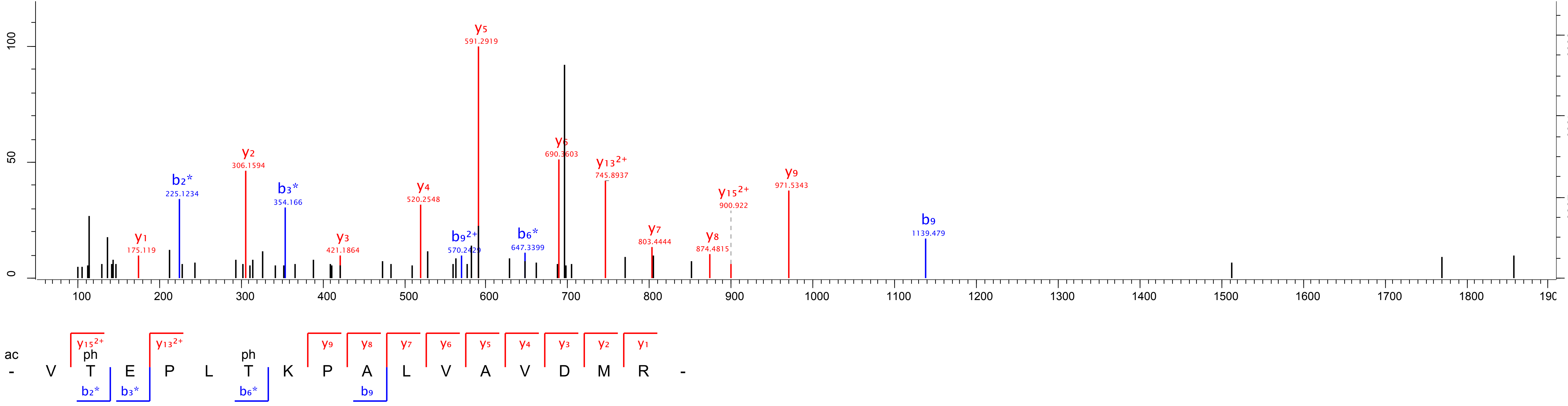

Raw file	Scan	Method	Score	m/z
20101013_Velos3_NaNa_COLLAB_salvage_5527_02	5083	FTMS; HCD	62.09	544.75

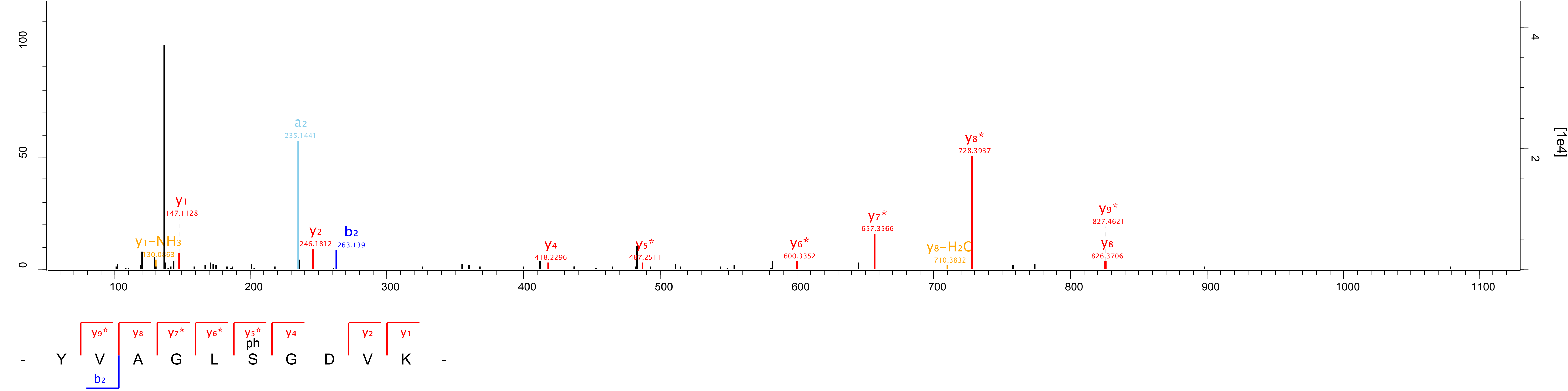

Raw file	Scan	Method	Score	m/z
20101013_Velos3_NaNa_COLLAB_5527_rep_02_flowthru_01	5879	FTMS; HCD	104.18	711.96

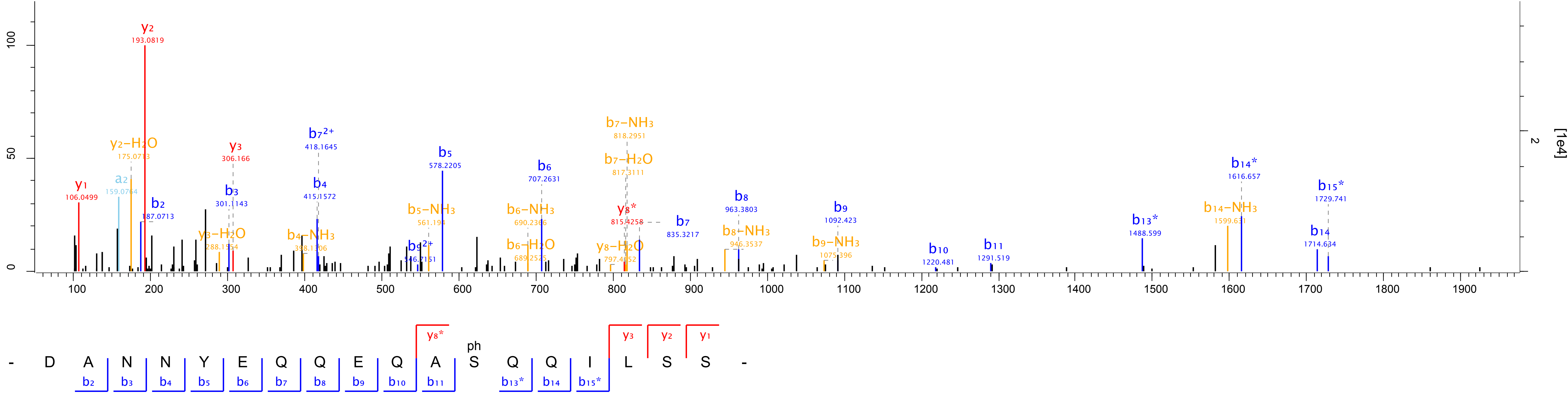

Raw file	Scan	Method	Score	m/z
20101013_Velos3_NaNa_COLLAB_5527_rep_02_flowthru_01	6267	FTMS; HCD	147.09	711.96

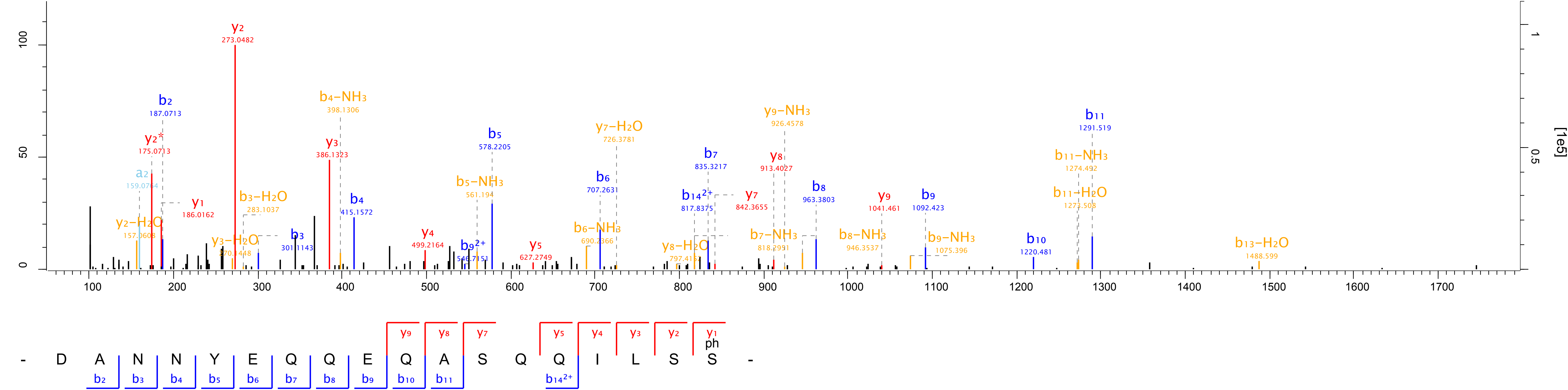

Raw file	Scan	Method	Score	m/z
20101013_Velos3_NaNa_COLLAB_5527_rep_03_fraction01	3831	FTMS; HCD	62.69	559.74

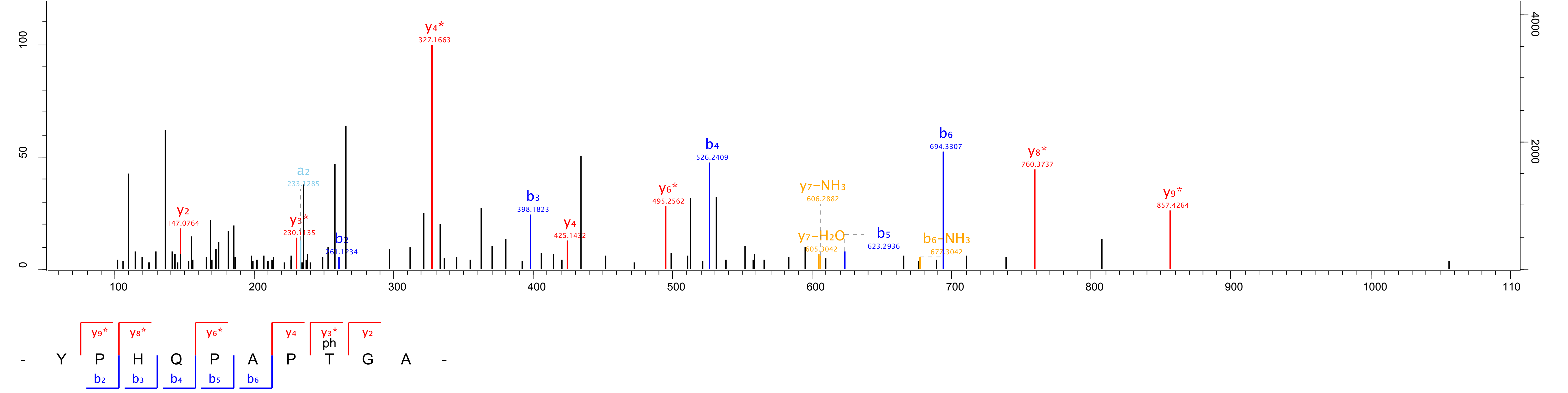

Raw file	Scan	Method	Score	m/z
20101013_Velos3_NaNa_COLLAB_5527_rep_01_flowthru_03	4802	FTMS; HCD	104.18	1007.91

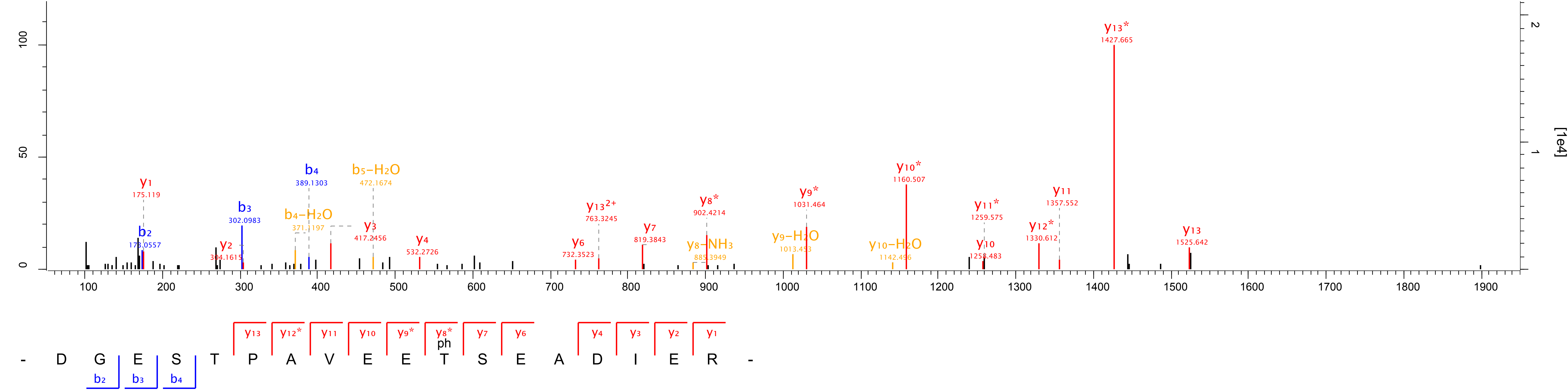

Raw file	Scan	Method	Score	m/z
20101013_Velos3_NaNa_COLLAB_5527_rep_03_flowthru_01	6800	FTMS; HCD	97.38	764.28

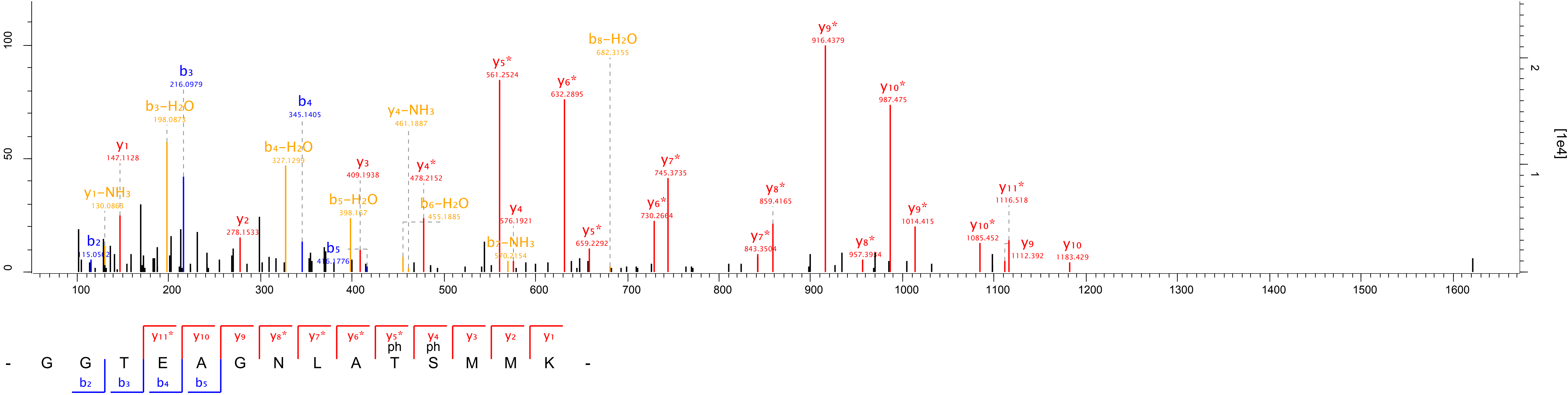

Raw file	Scan	Method	Score	m/z
20101013_Velos3_NaNa_COLLAB_5527_rep_02_fraction02	2831	FTMS; HCD	87.77	744.32

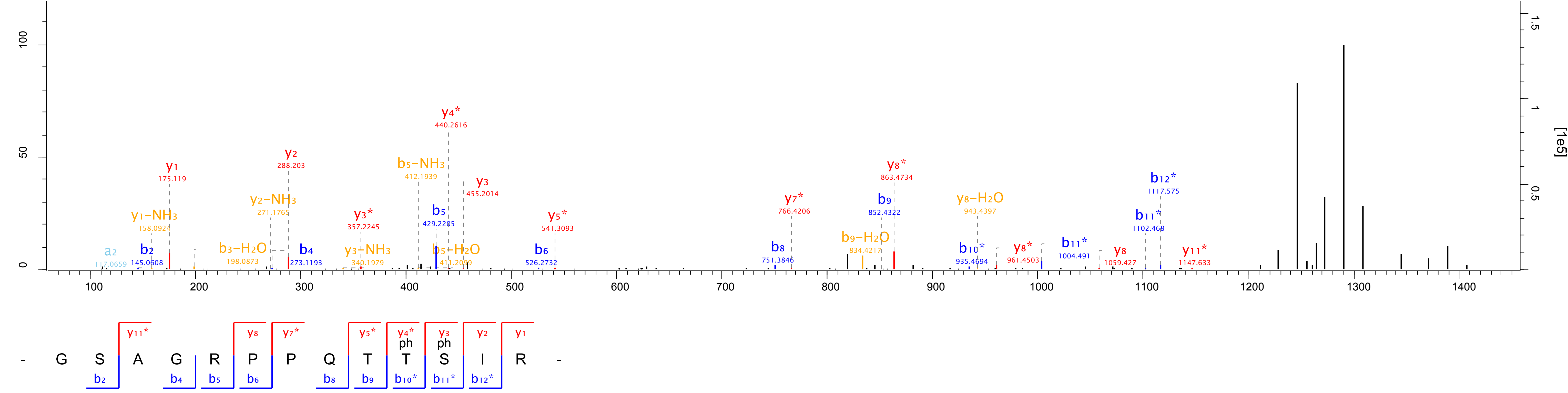

Raw file	Scan	Method	Score	m/z
20101013_Velos3_NaNa_COLLAB_5527_rep_03_flowthru_01	7580	FTMS; HCD	56.72	764.28

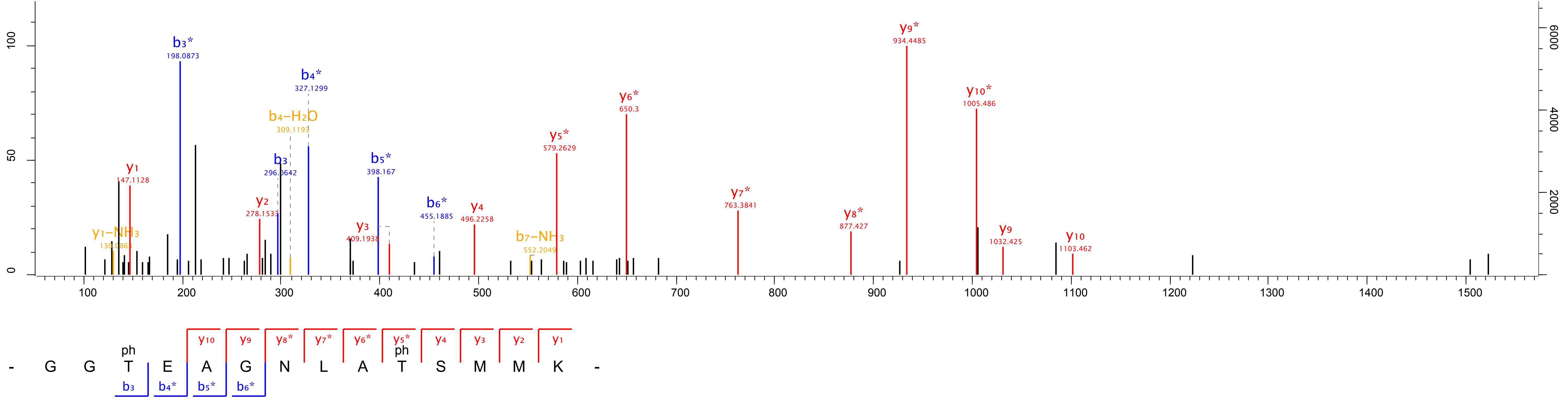

Raw file	Scan	Method	Score	m/z
20101013_Velos3_NaNa_COLLAB_salvage_5527_02	4185	FTMS; HCD	105.99	634.31

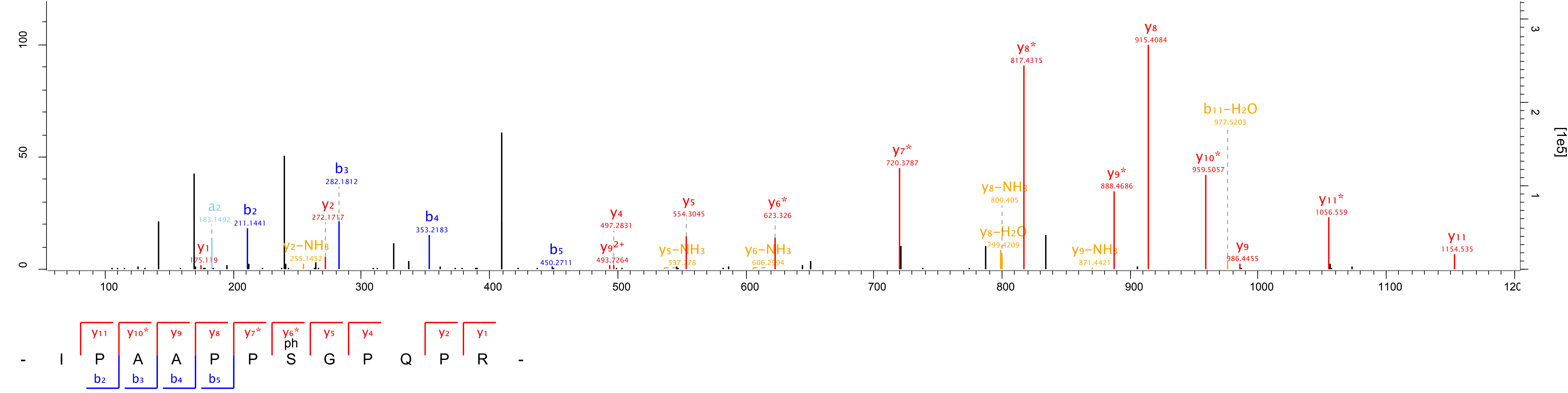

Raw file	Scan	Method	Score	m/z
20101013_Velos3_NaNa_COLLAB_5527_rep_01_fraction01	7631	FTMS; HCD	98	930.45

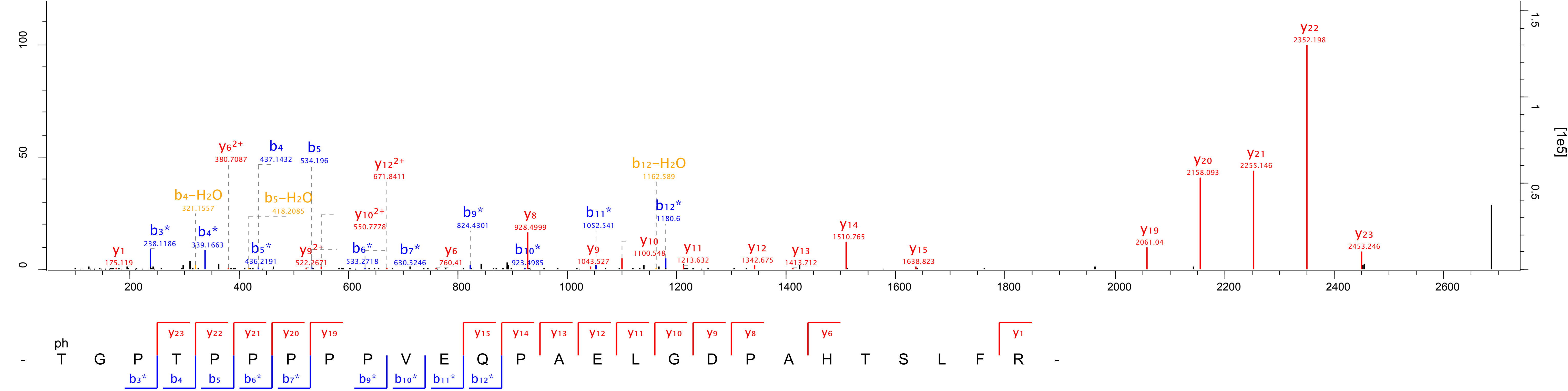

Raw file	Scan	Method	Score	m/z
20101013_Velos3_NaNa_COLLAB_salvage_5527_02	4808	FTMS; HCD	98.57	847.39

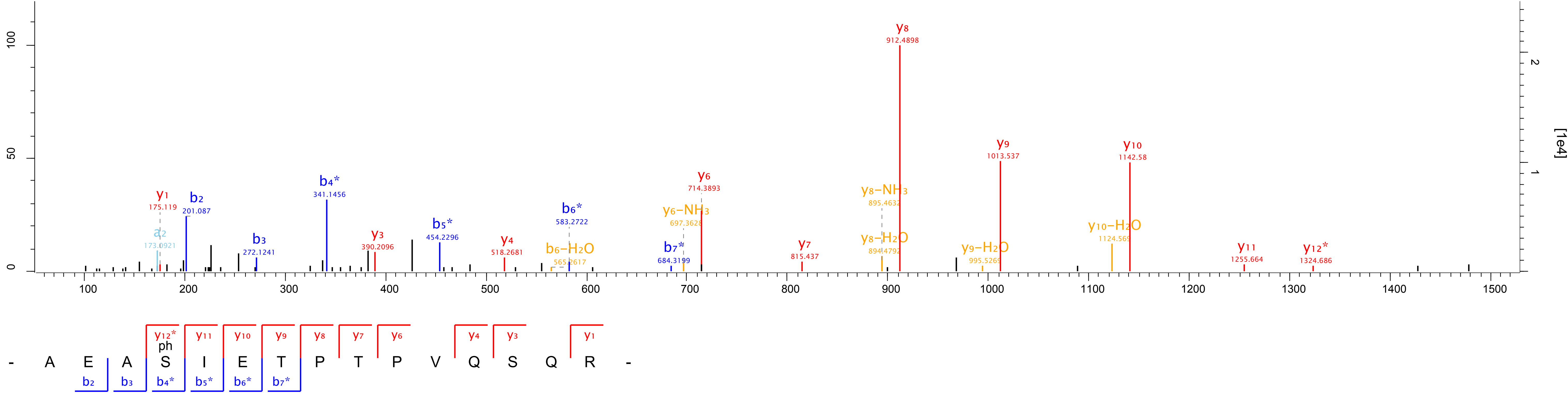

Raw file	Scan	Method	Score	m/z
20101013_Velos3_NaNa_COLLAB_salvage_5527_02	3113	FTMS; HCD	181.87	869.87

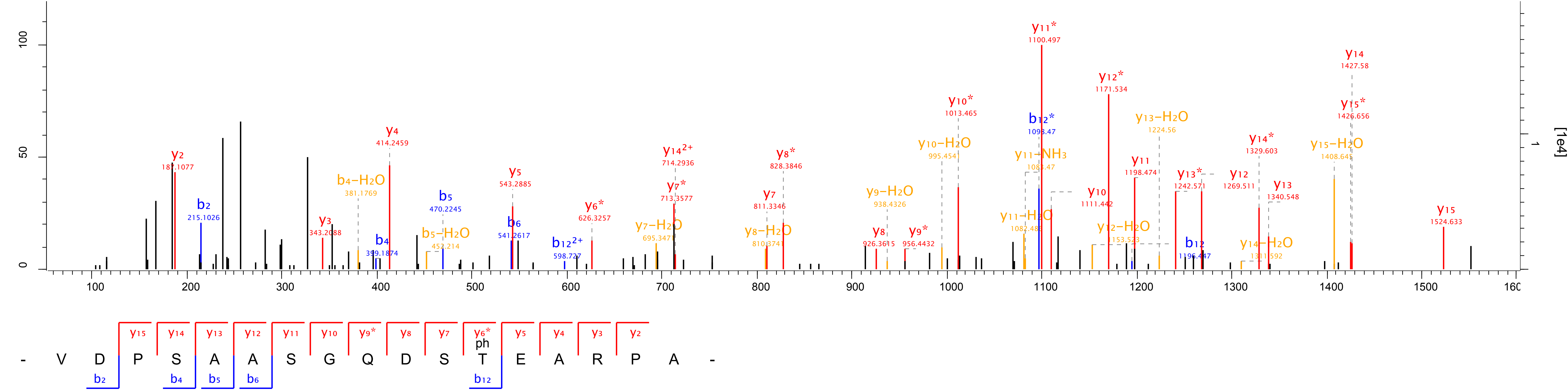

Raw file	Scan	Method	Score	m/z
20101013_Velos3_NaNa_COLLAB_salvage_5527_03	8190	FTMS; HCD	46.41	570.62

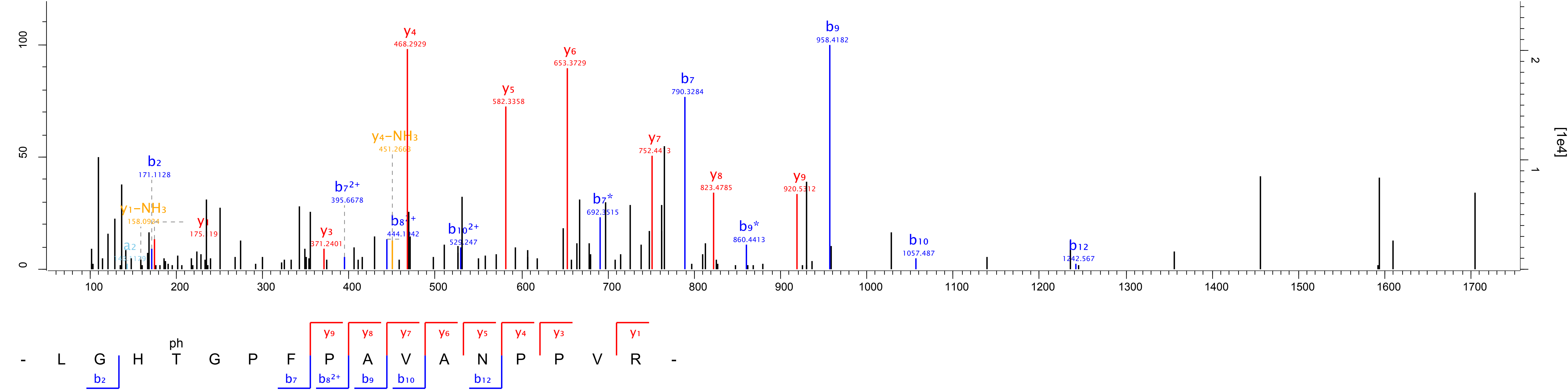

Raw file	Scan	Method	Score	m/z
20101013_Velos3_NaNa_COLLAB_5527_rep_01_flowthru_03	6583	FTMS; HCD	89.3	692.95

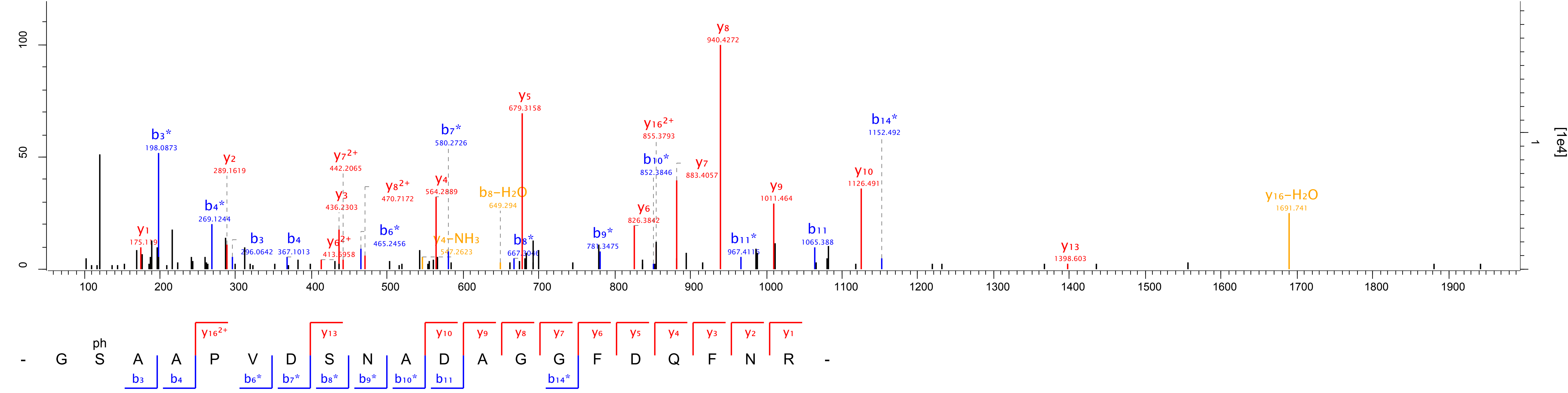

Raw file	Scan	Method	Score	m/z
20101013_Velos3_NaNa_COLLAB_5527_rep_01_flowthru_01	6221	FTMS; HCD	194.68	714.64

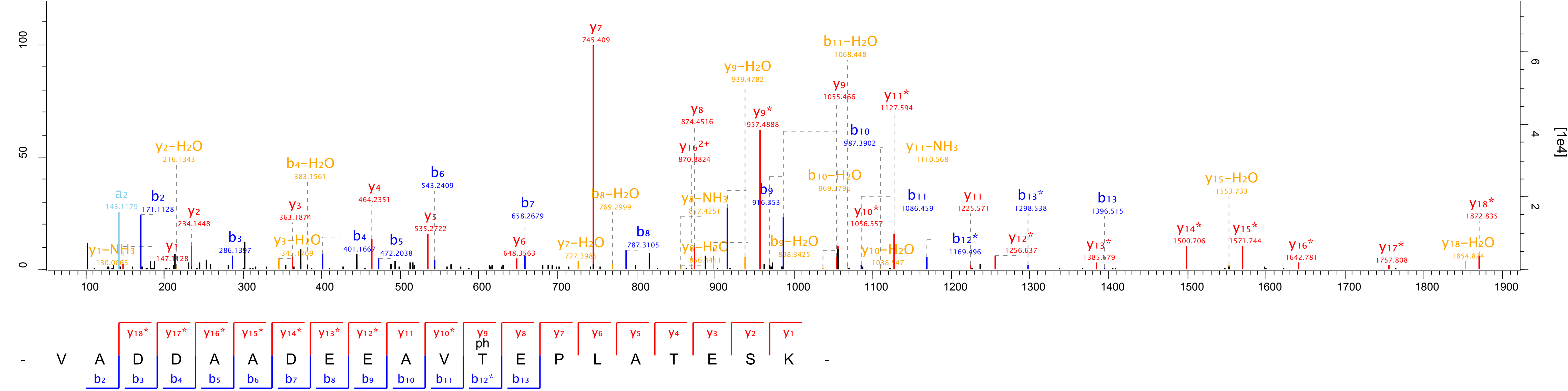

Raw file	Scan	Method	Score	m/z
20101013_Velos3_NaNa_COLLAB_salvage_5527_02	5714	FTMS; HCD	42.84	684.31

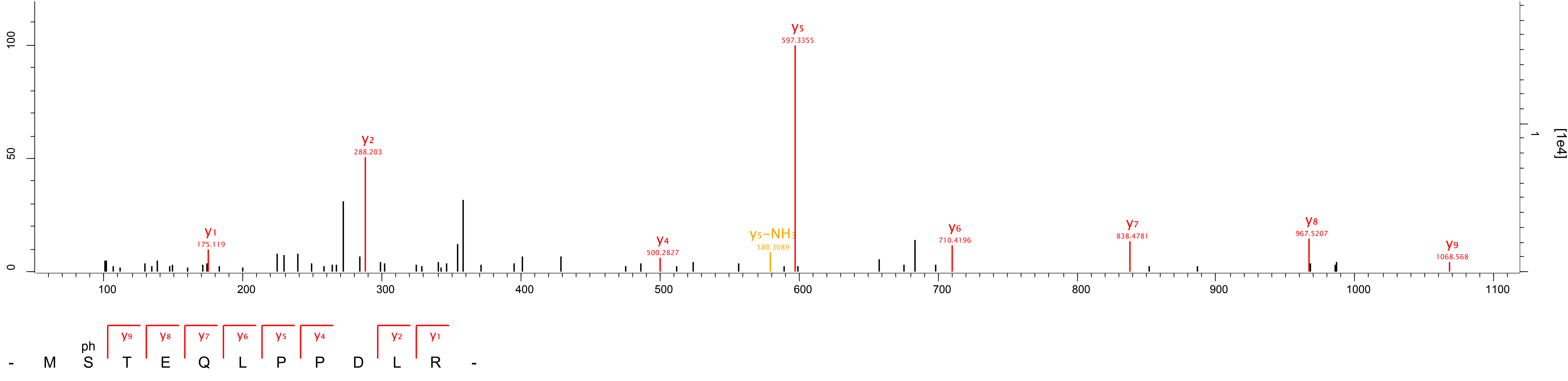

Raw file	Scan	Method	Score	m/z
20101013_Velos3_NaNa_COLLAB_5527_rep_03_fraction02	4355	FTMS; HCD	49.77	486.22

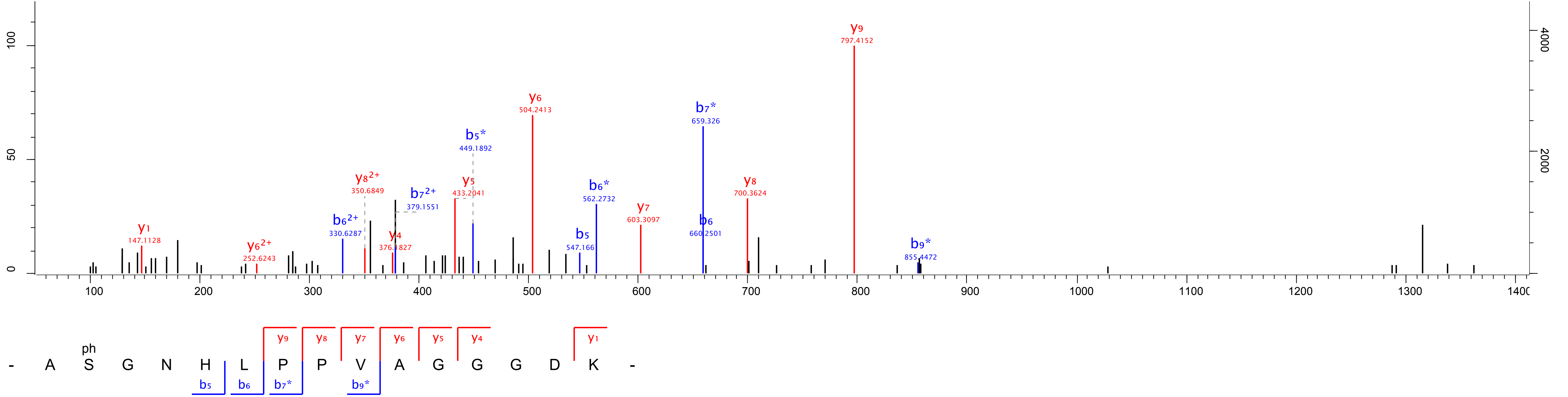

Raw file	Scan	Method	Score	m/z
20101013_Velos3_NaNa_COLLAB_5527_rep_03_flowthru_02	2898	FTMS; HCD	53.24	472.7

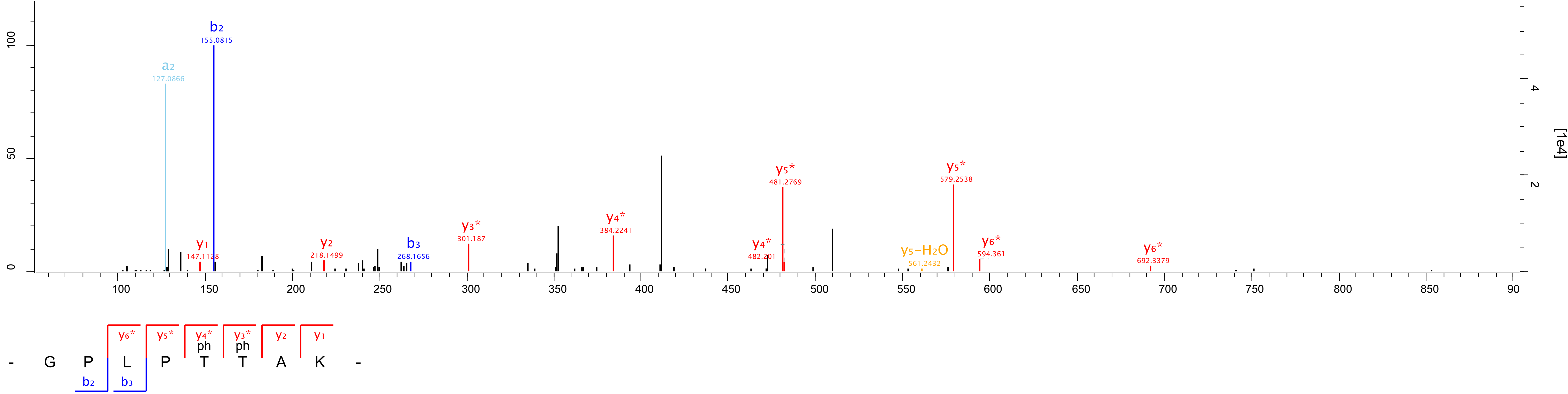

Raw file	Scan	Method	Score	m/z
20101013_Velos3_NaNa_COLLAB_5527_rep_03_flowthru_02	6135	FTMS; HCD	64.52	656.25

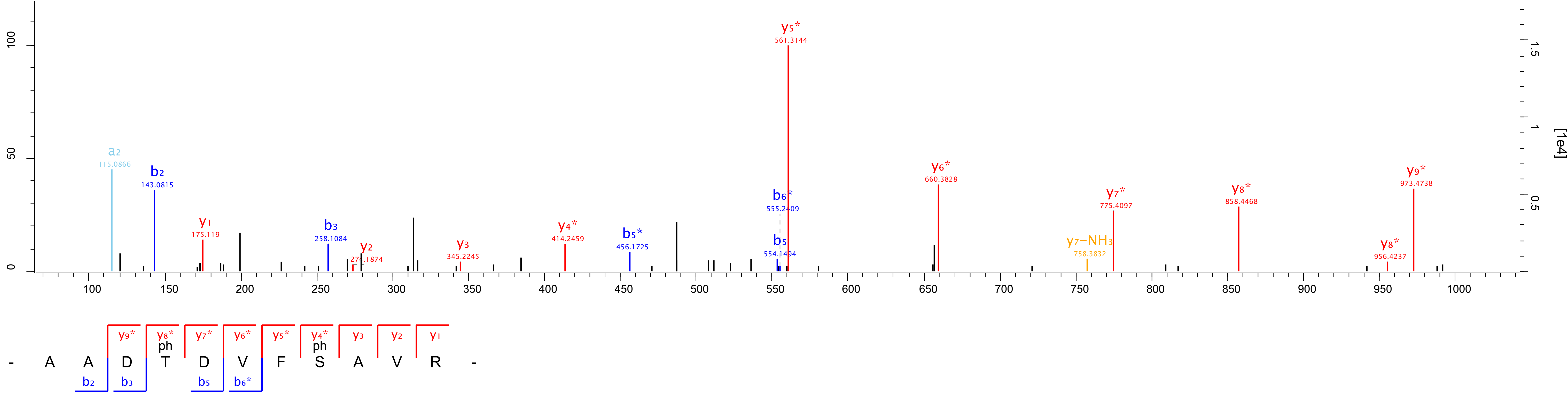

Raw file	Scan	Method	Score	m/z
20101013_Velos3_NaNa_COLLAB_5527_rep_03_fraction01	4917	FTMS; HCD	58.7	488.73

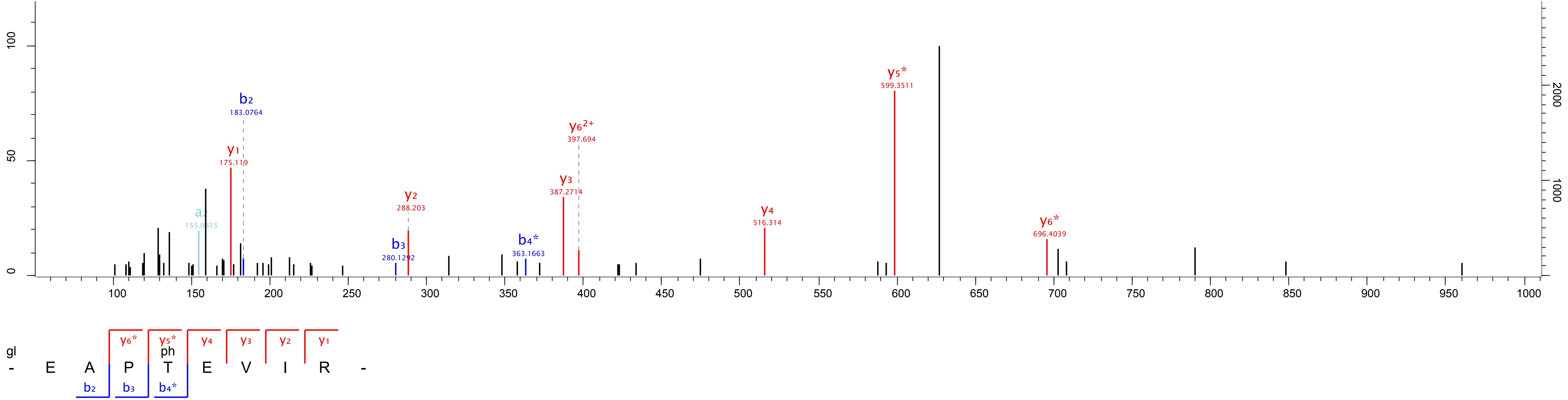

Raw file	Scan	Method	Score	m/z
20101013_Velos3_NaNa_COLLAB_5527_rep_03_flowthru_01	7878	FTMS; HCD	52.47	742.64

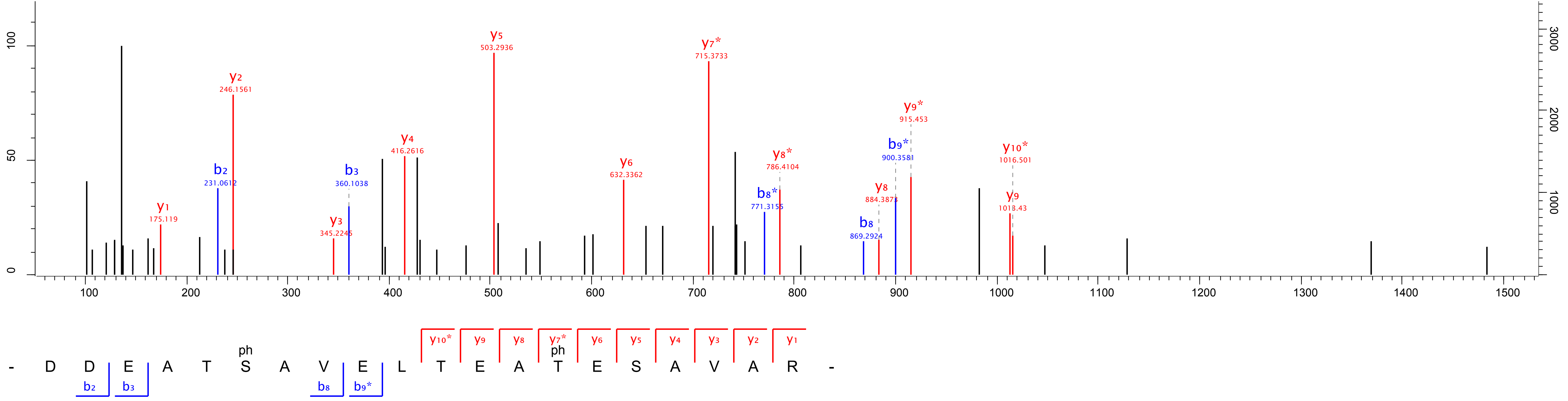

Raw file	Scan	Method	Score	m/z
20101013_Velos3_NaNa_COLLAB_5527_rep_01_flowthru_02	9327	FTMS; HCD	149.71	715.98

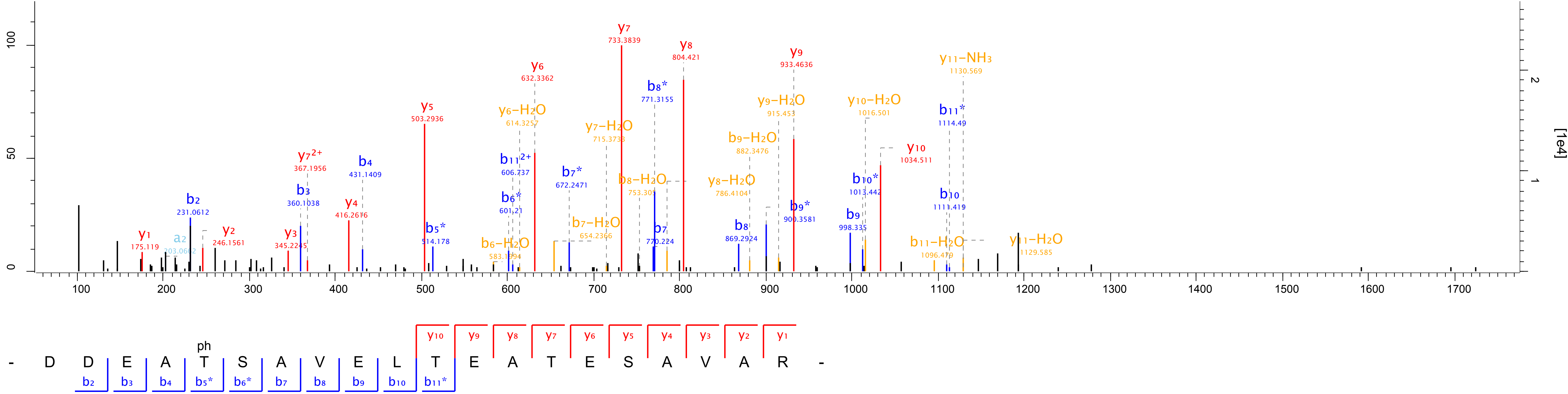

Raw file	Scan	Method	Score	m/z
20101013_Velos3_NaNa_COLLAB_5527_rep_03_fraction01	5866	FTMS; HCD	76.01	619.3

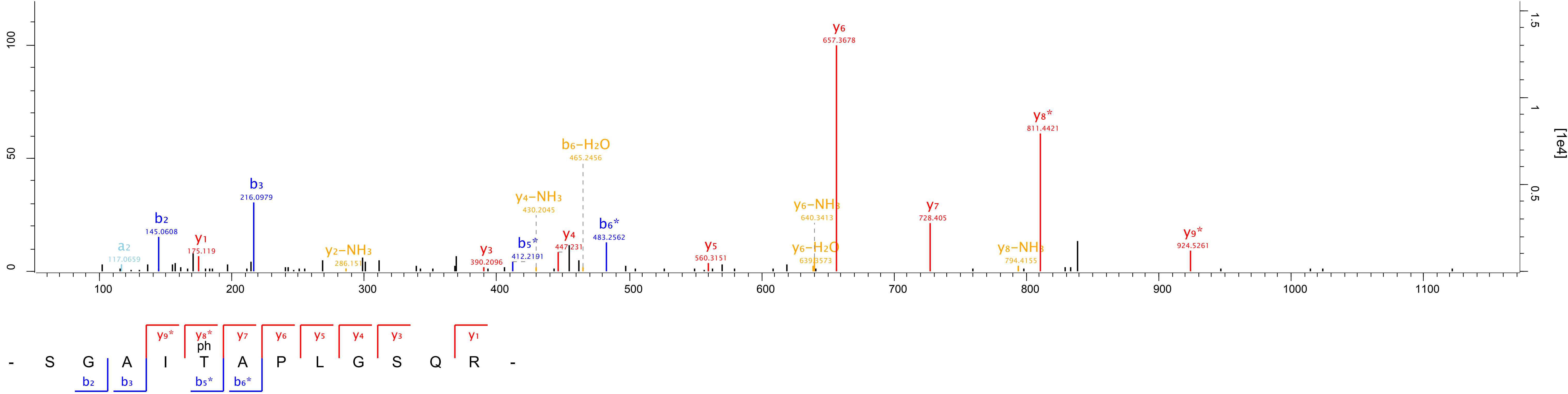

Raw file	Scan	Method	Score	m/z
20101013_Velos3_NaNa_COLLAB_5527_rep_03_fraction02	10993	FTMS; HCD	89.46	691.66

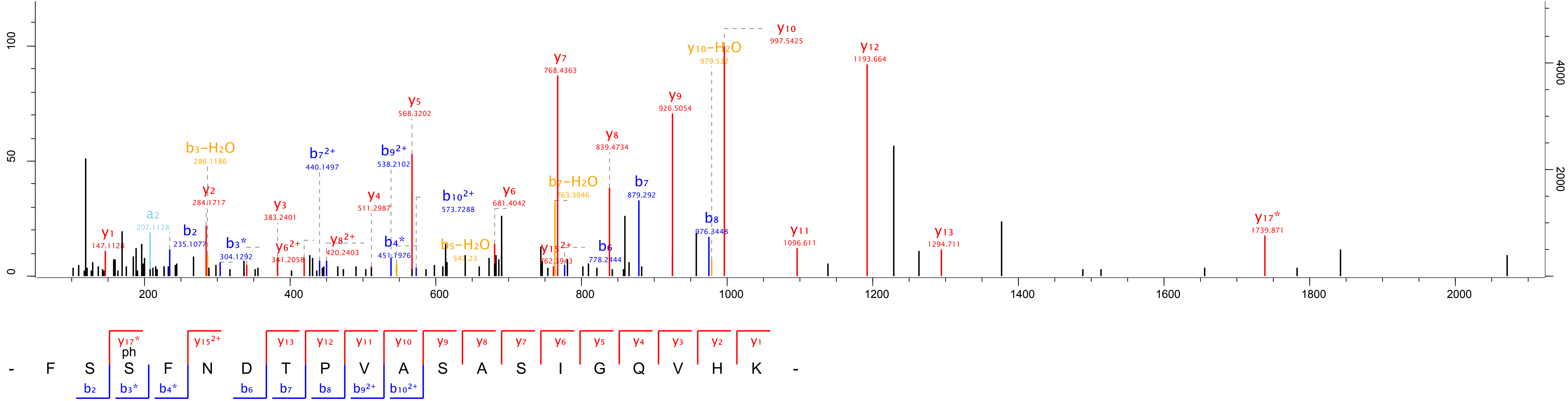

Raw file	Scan	Method	Score	m/z
20101013_Velos3_NaNa_COLLAB_salvage_5527_02	6400	FTMS; HCD	62.2	761.84

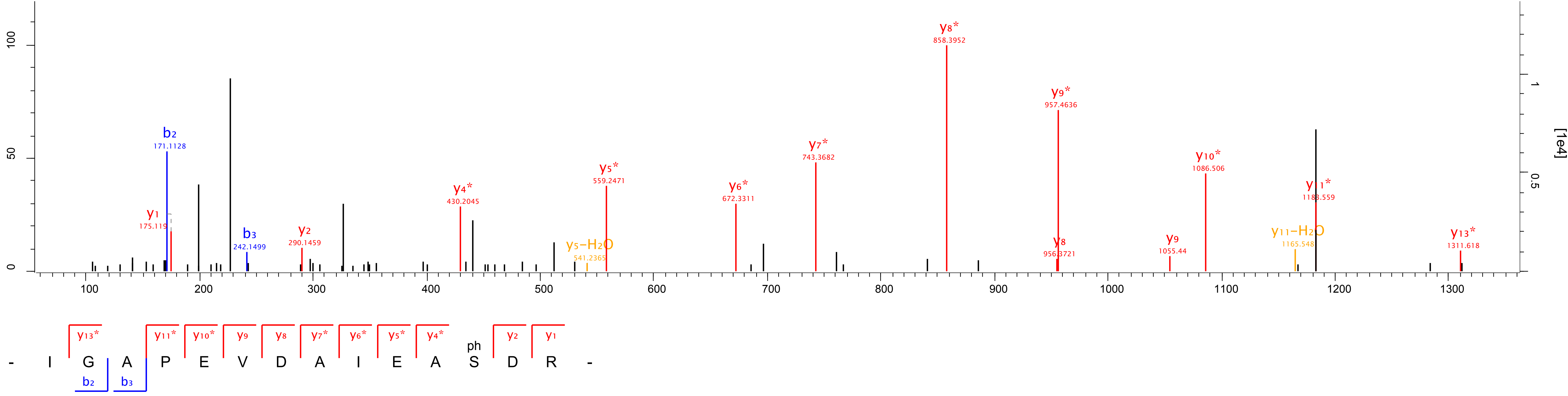

Raw file	Scan	Method	Score	m/z
20101013_Velos3_NaNa_COLLAB_5527_rep_01_flowthru_03	3712	FTMS; HCD	129.77	822.37

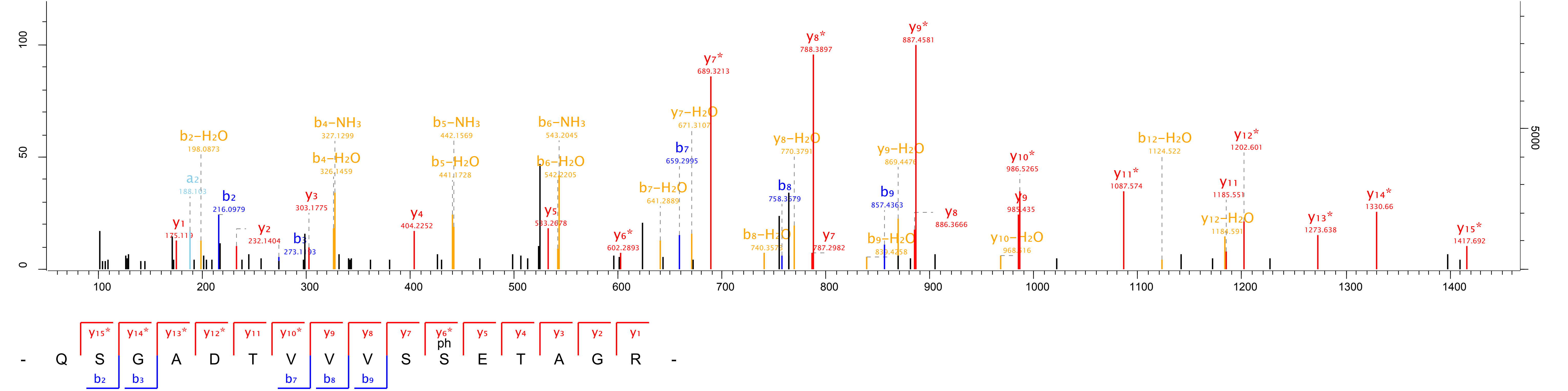

Raw file	Scan	Method	Score	m/z
20101013_Velos3_NaNa_COLLAB_5527_rep_02_fraction05	4665	FTMS; HCD	81.59	516.59

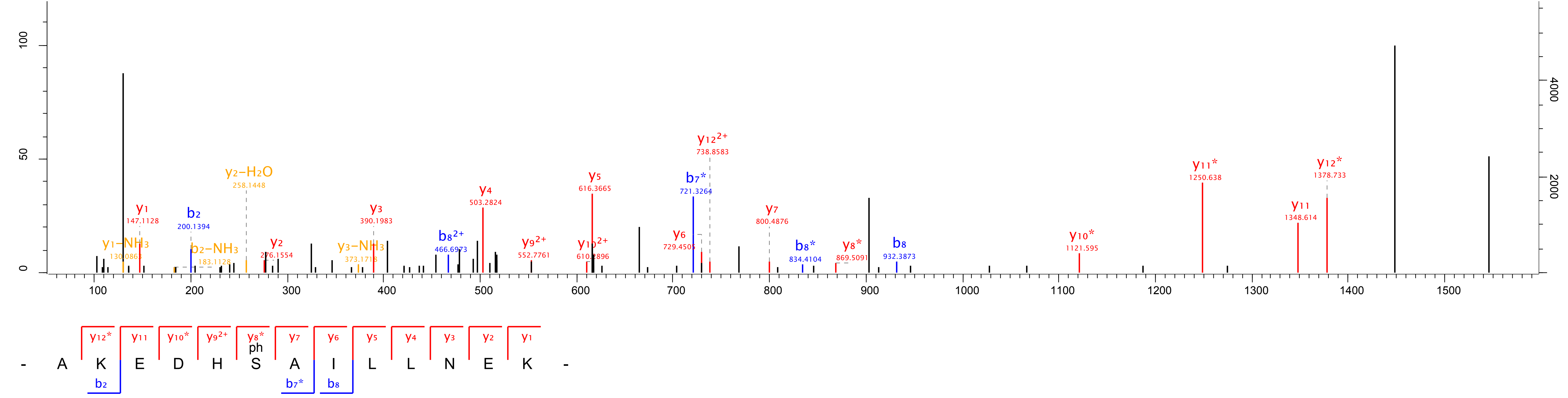

Raw file	Scan	Method	Score	m/z
20101013_Velos3_NaNa_COLLAB_salvage_5527_02	5591	FTMS; HCD	68.54	455.74

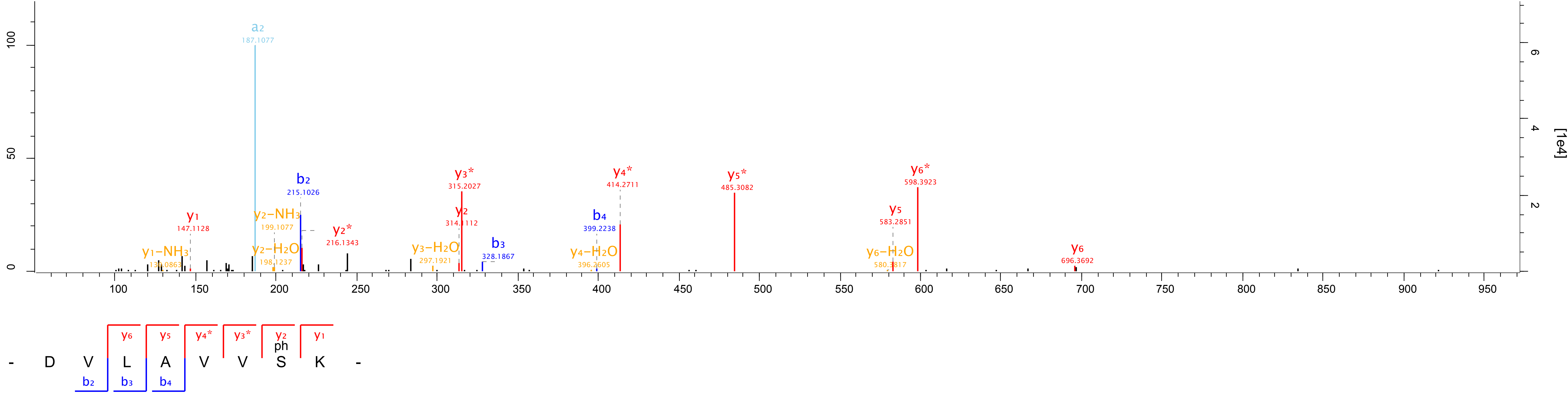

Raw file	Scan	Method	Score	m/z
20101013_Velos3_NaNa_COLLAB_5527_rep_03_fraction03	7327	FTMS; HCD	89.62	702.82

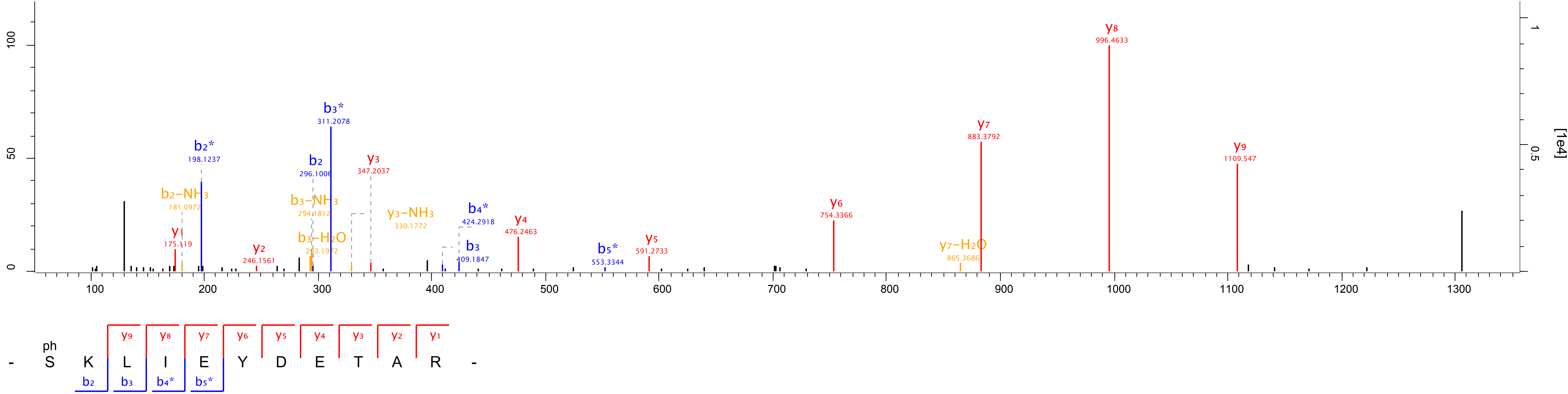

Raw file	Scan	Method	Score	m/z
20101013_Velos3_NaNa_COLLAB_5527_rep_01_flowthru_03	4294	FTMS; HCD	96.46	577.78

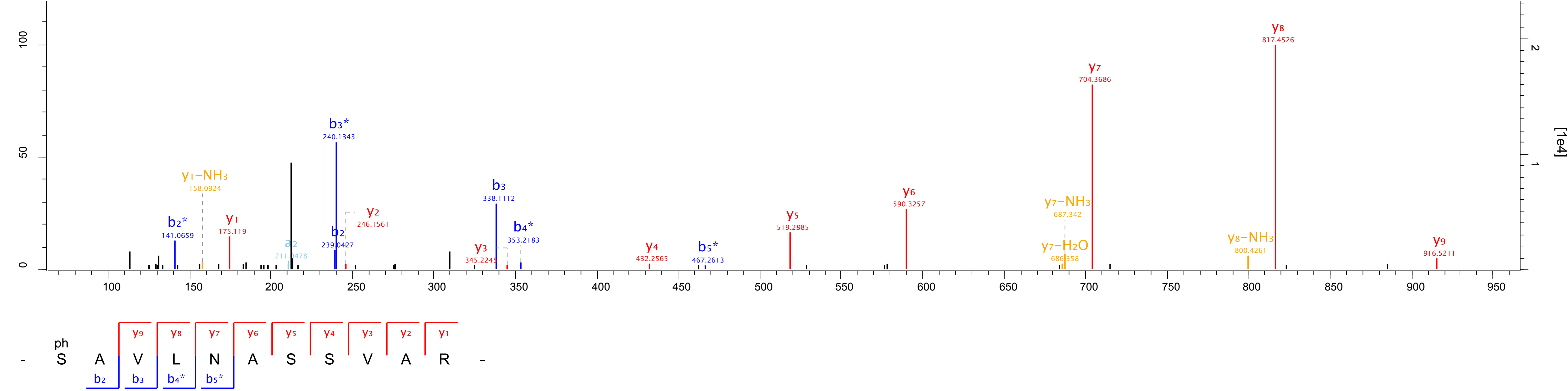

Raw file	Scan	Method	Score	m/z
20101013_Velos3_NaNa_COLLAB_5527_rep_01_fraction04	8086	FTMS; HCD	122.49	684.29

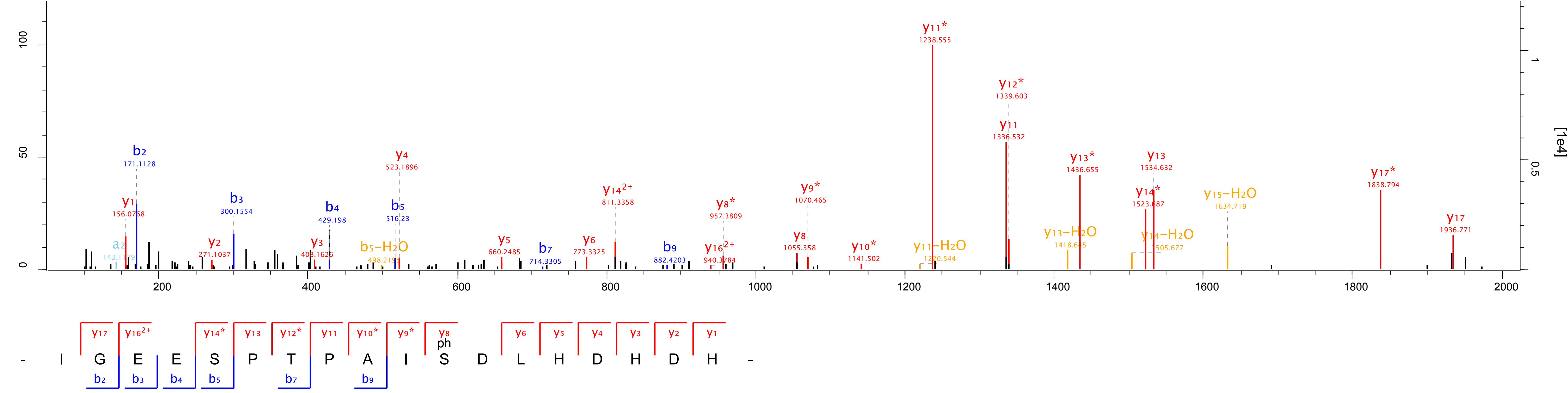

Raw file	Scan	Method	Score	m/z
20101013_Velos3_NaNa_COLLAB_salvage_5527_01	4191	FTMS; HCD	44.54	338.18

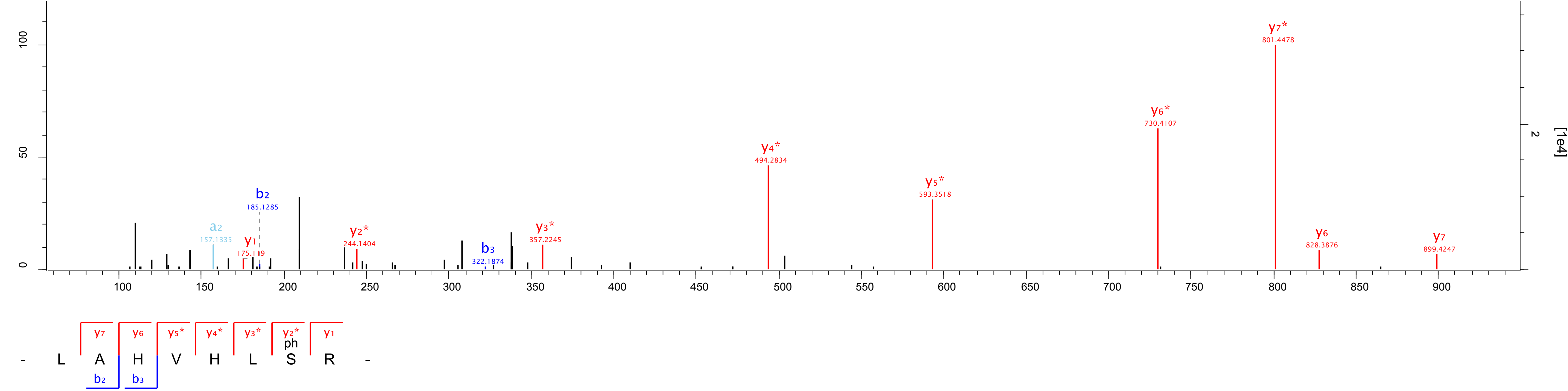

Raw file	Scan	Method	Score	m/z
20101013_Velos3_NaNa_COLLAB_5527_rep_03_flowthru_02	10173	FTMS; HCD	88.18	956.46

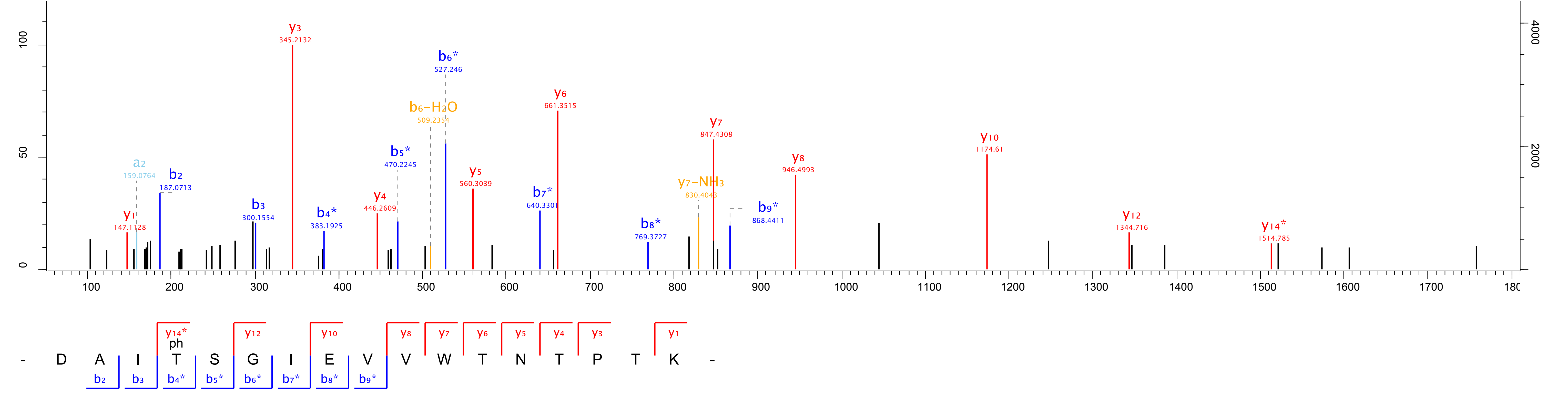

Raw file	Scan	Method	Score	m/z
20101013_Velos3_NaNa_COLLAB_salvage_5527_02	3169	FTMS; HCD	78.1	463.21

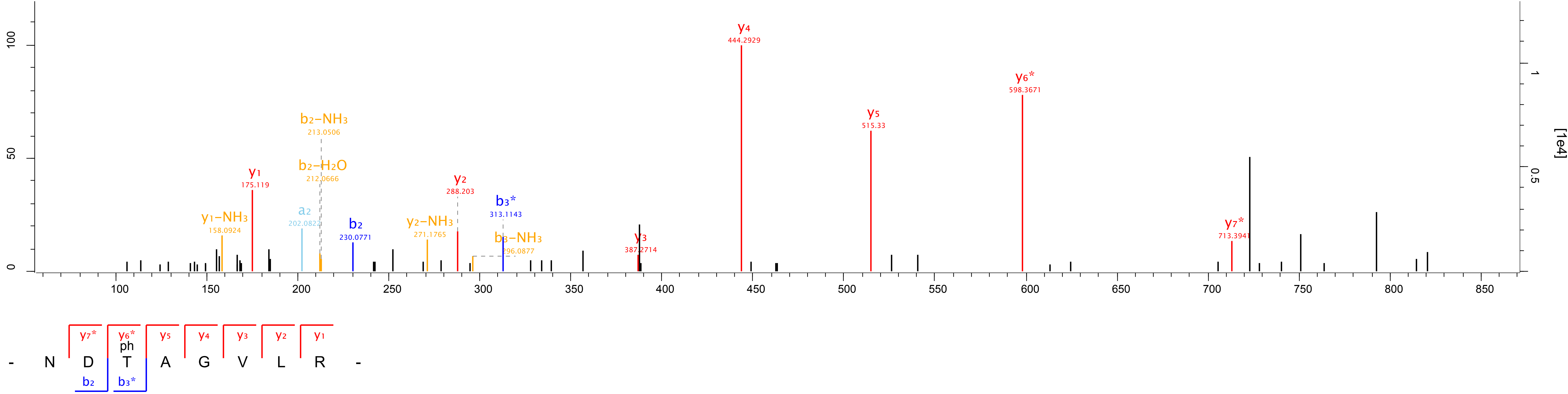

Raw file	Scan	Method	Score	m/z
20101013_Velos3_NaNa_COLLAB_salvage_5527_02	3169	FTMS; HCD	78.1	463.21

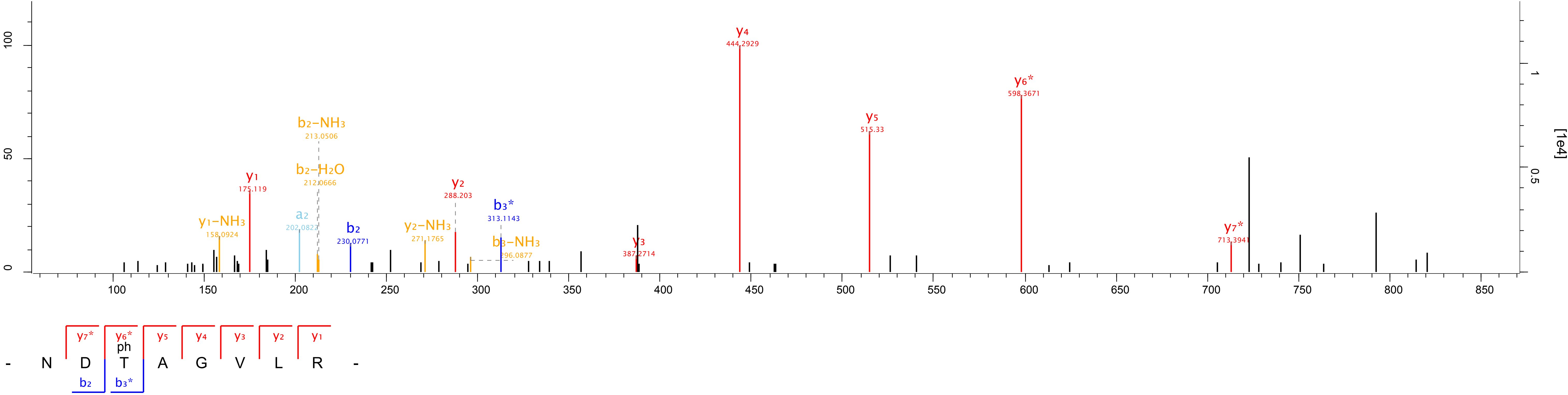

Raw file	Scan	Method	Score	m/z
20101013_Velos3_NaNa_COLLAB_5527_rep_03_fraction01	3315	FTMS; HCD	64.1	721.78

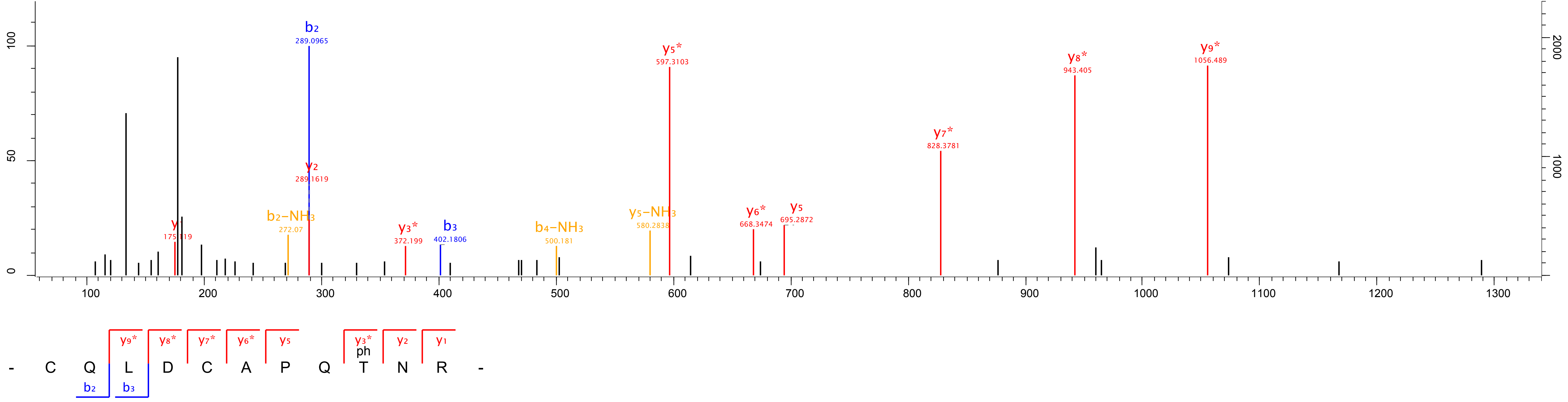

Raw file	Scan	Method	Score	m/z
20101013_Velos3_NaNa_COLLAB_5527_rep_01_flowthru_01	9020	FTMS; HCD	60.76	805.38

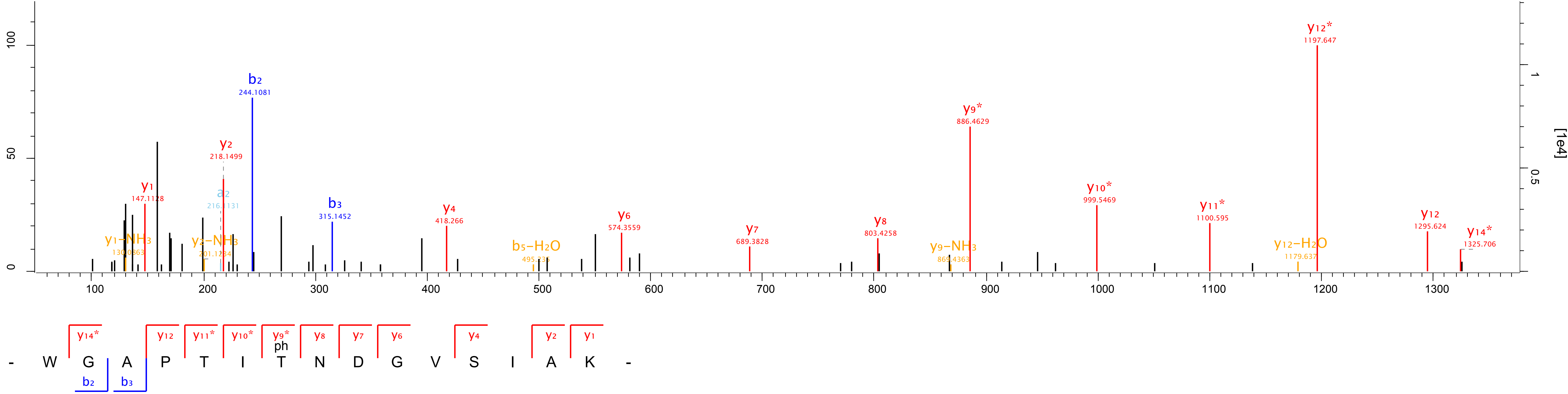

Raw file	Scan	Method	Score	m/z
20101013_Velos3_NaNa_COLLAB_5527_rep_03_flowthru_01	3658	FTMS; HCD	130.72	775.79

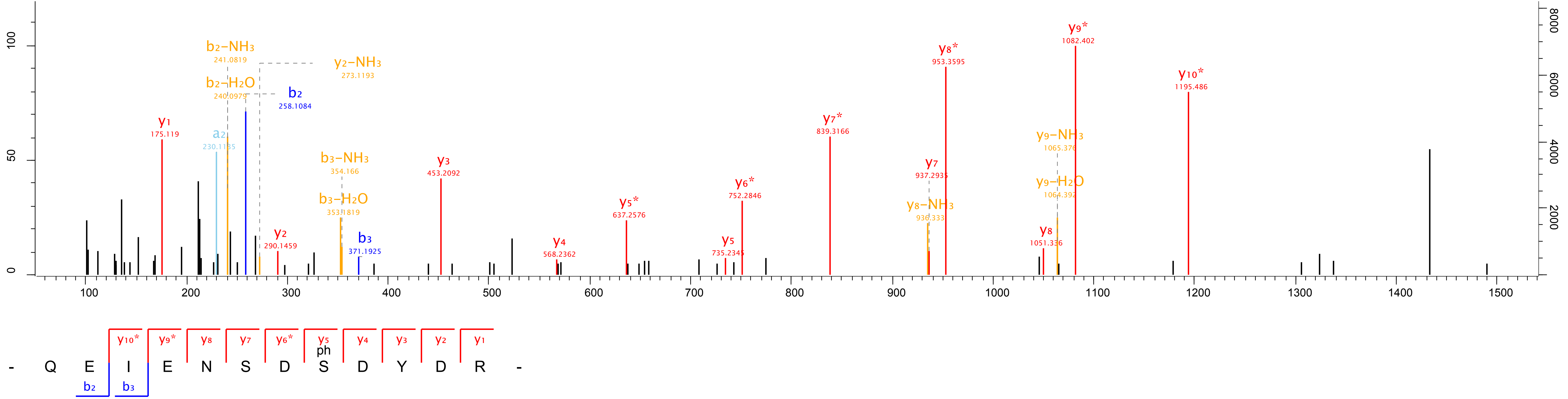

Raw file	Scan	Method	Score	m/z
20101013_Velos3_NaNa_COLLAB_5527_rep_03_fraction03	4123	FTMS; HCD	178.23	603.24

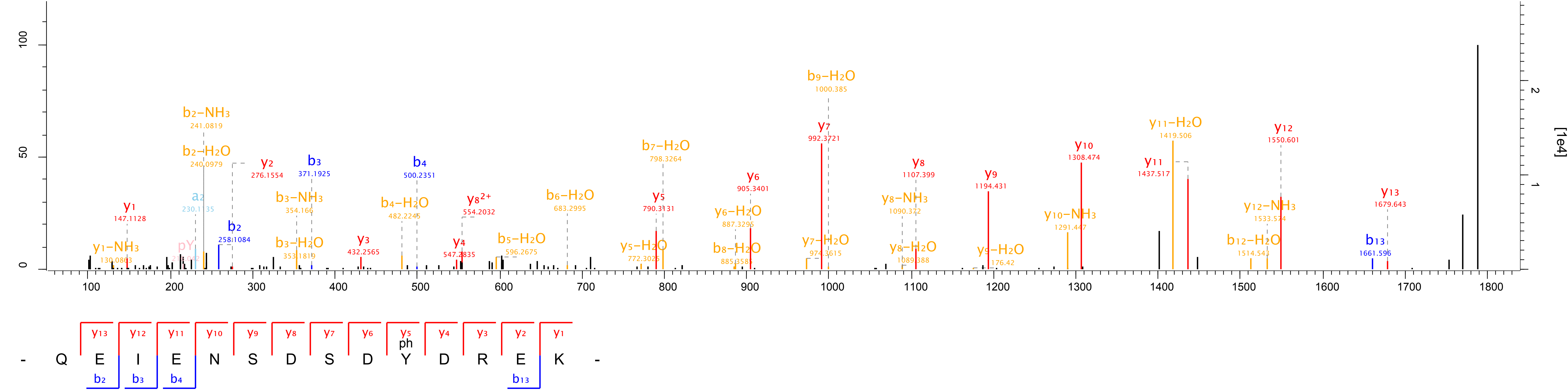

Raw file	Scan	Method	Score	m/z
20101013_Velos3_NaNa_COLLAB_5527_rep_01_flowthru_03	4270	FTMS; HCD	81.85	545.6

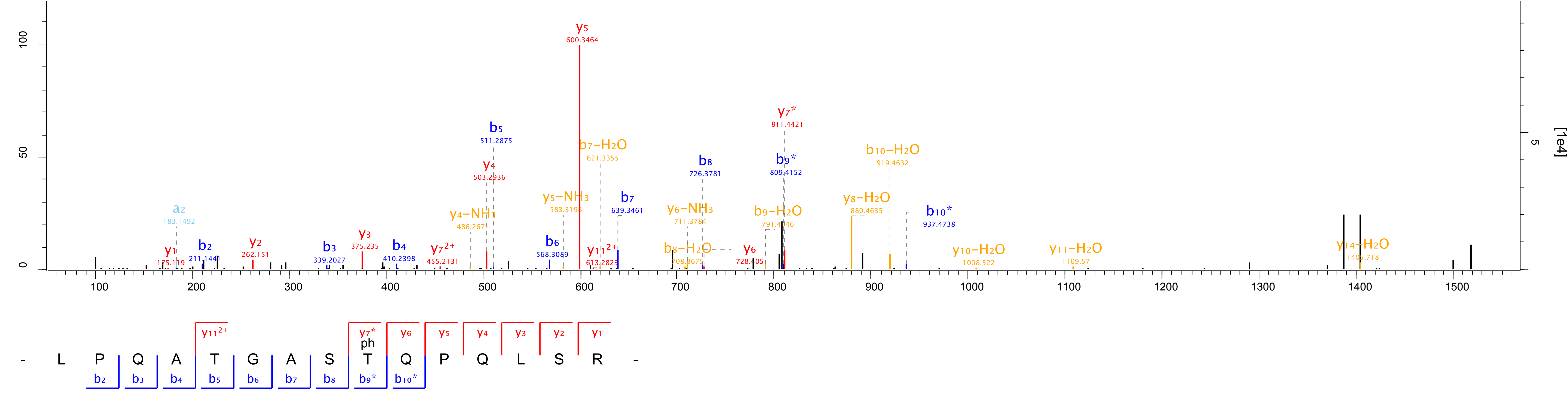

Raw file	Scan	Method	Score	m/z
20101013_Velos3_NaNa_COLLAB_5527_rep_02_fraction02	3145	FTMS; HCD	131.26	724.32

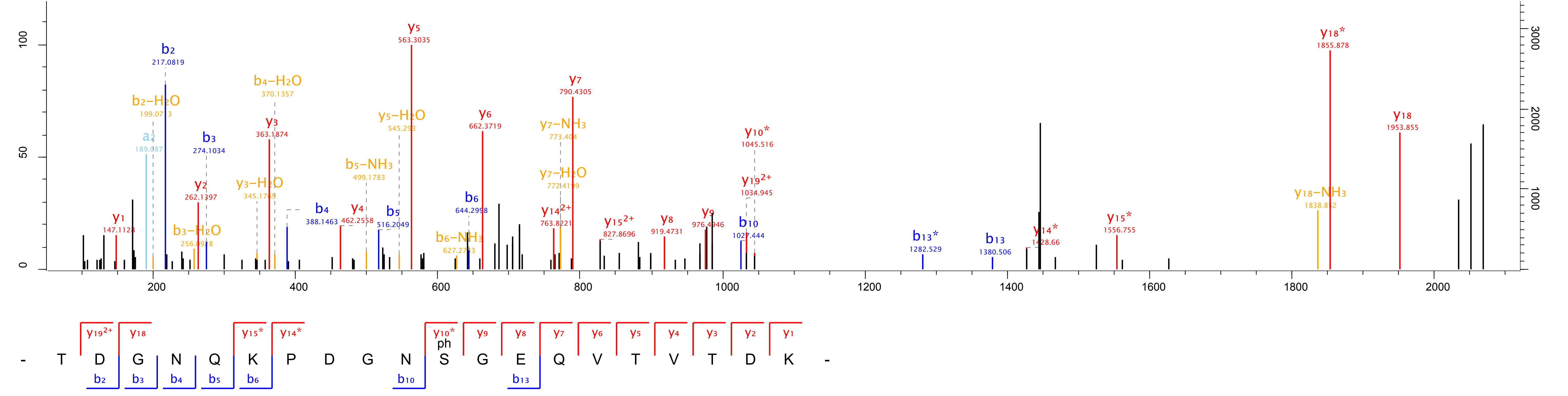

Raw file	Scan	Method	Score	m/z
20101013_Velos3_NaNa_COLLAB_5527_rep_02_fraction03	3973	FTMS; HCD	282.91	724.32

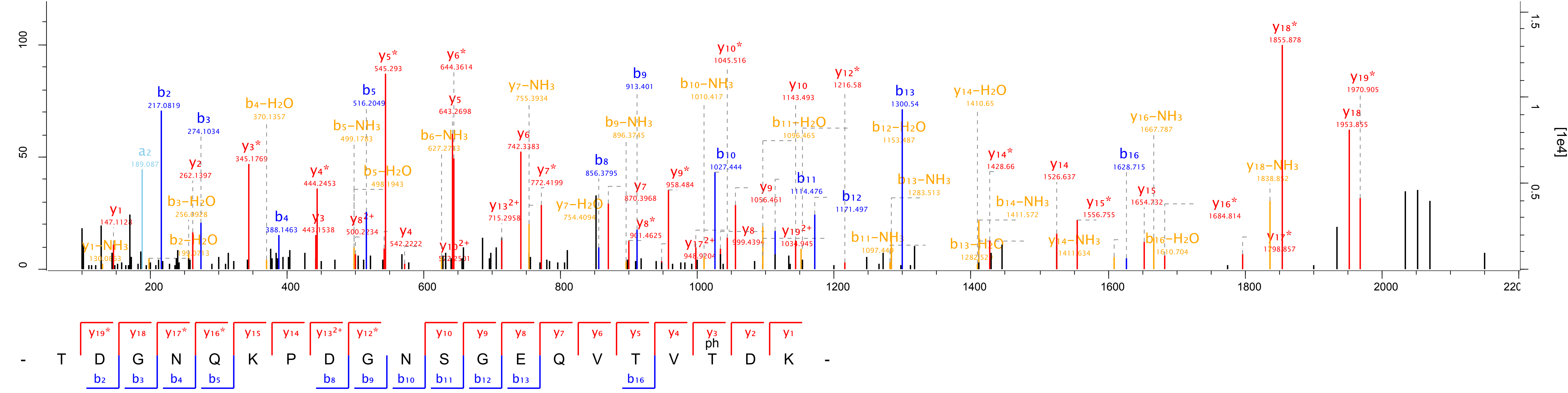

Raw file	Scan	Method	Score	m/z
20101013_Velos3_NaNa_COLLAB_5527_rep_01_fraction04	3741	FTMS; HCD	172.32	776.35

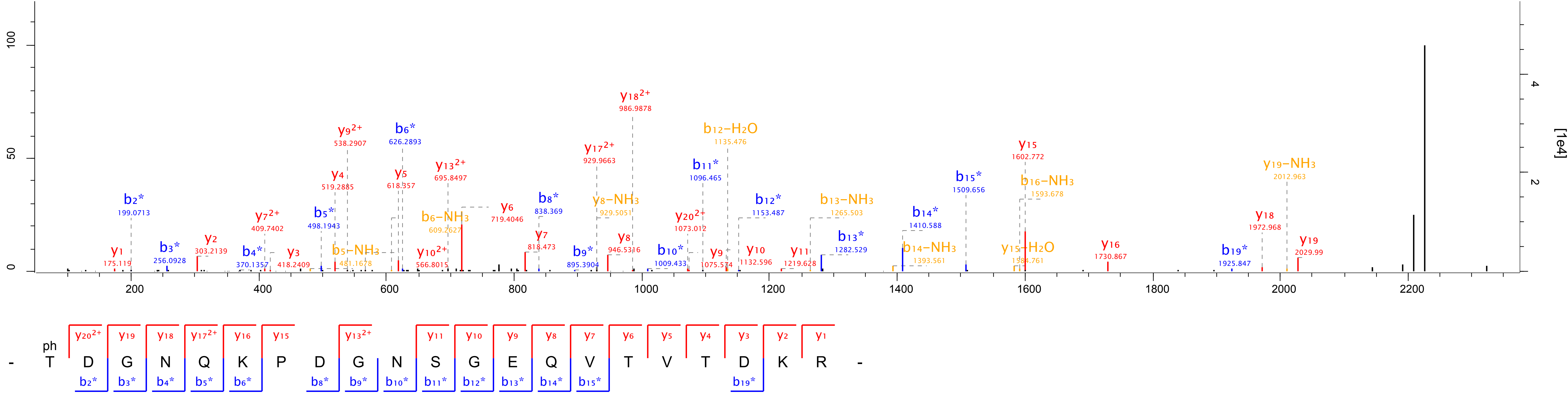

Raw file	Scan	Method	Score	m/z
20101013_Velos3_NaNa_COLLAB_salvage_5527_02	3283	FTMS; HCD	54.26	548.24

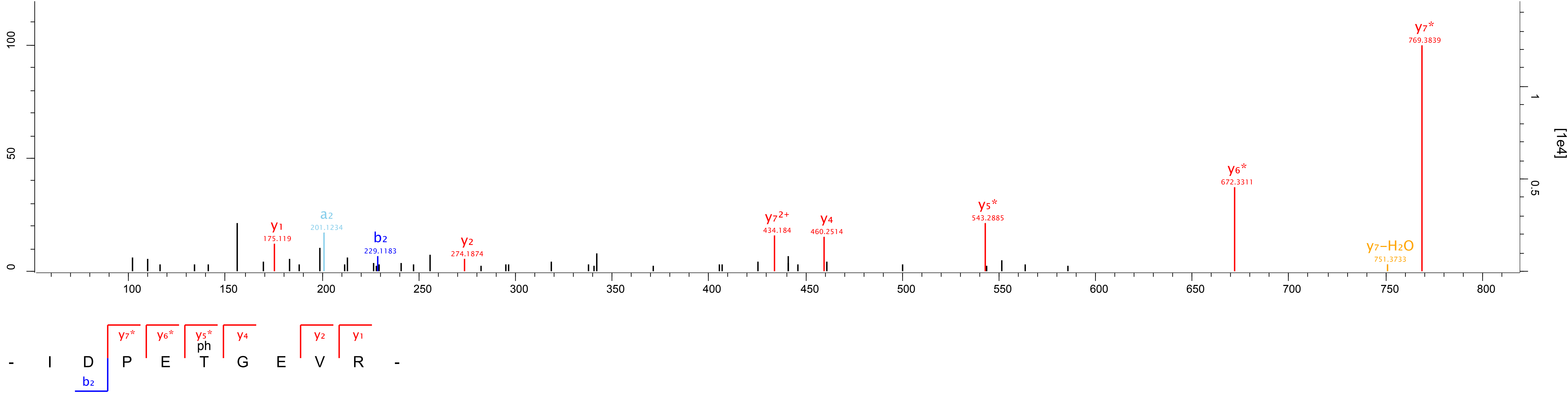

Raw file	Scan	Method	Score	m/z
20101013_Velos3_NaNa_COLLAB_salvage_5527_01	2670	FTMS; HCD	85.82	505.76

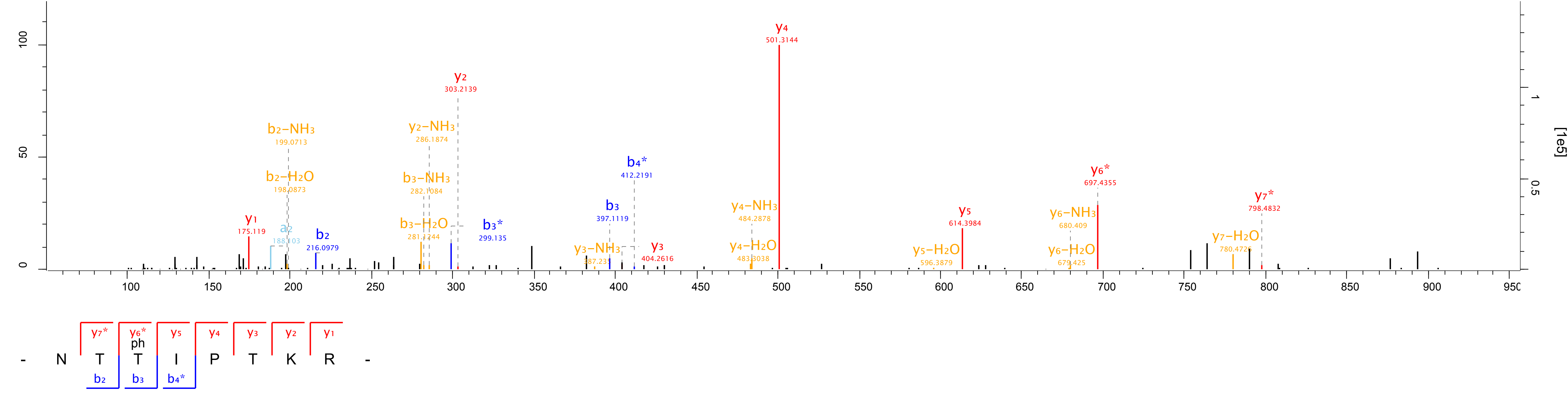

Raw file	Scan	Method	Score	m/z
20101013_Velos3_NaNa_COLLAB_5527_rep_02_fraction03	4033	FTMS; HCD	166.3	726.85

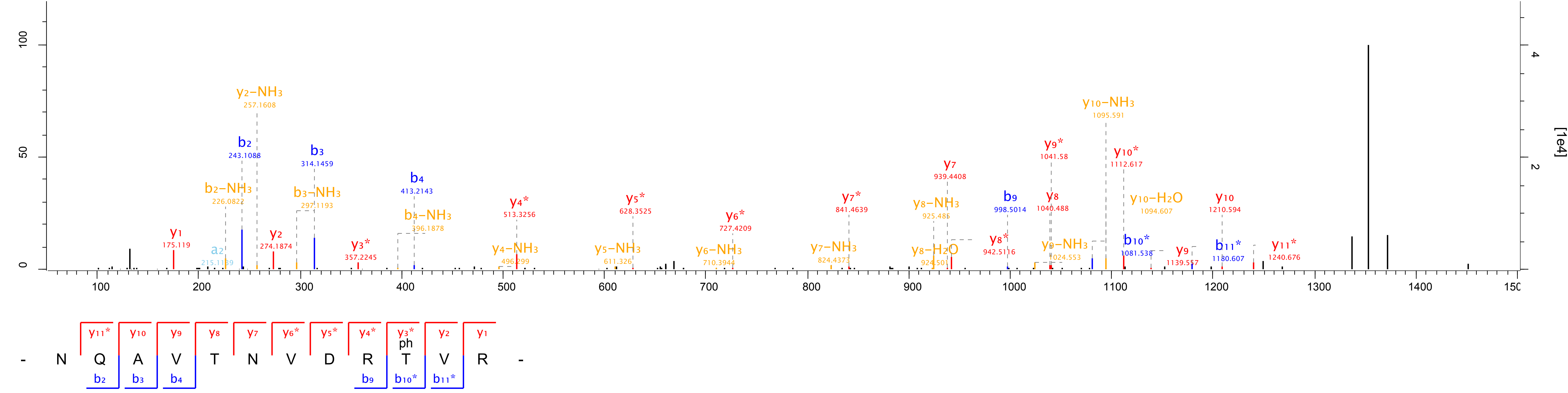

Raw file	Scan	Method	Score	m/z
20101013_Velos3_NaNa_COLLAB_5527_rep_03_fraction01	2478	FTMS; HCD	61.48	548.74

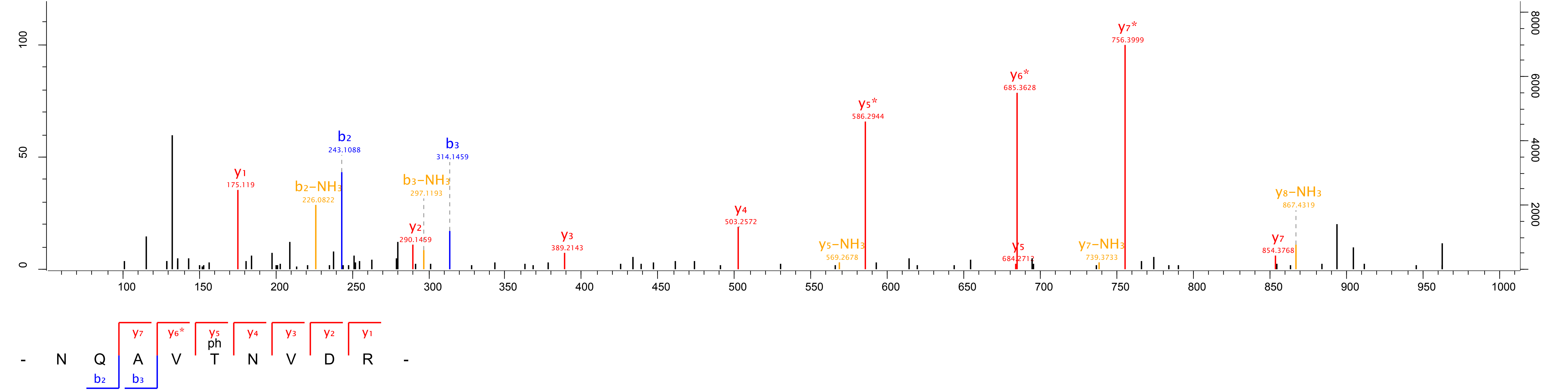

Raw file	Scan	Method	Score	m/z
20101013_Velos3_NaNa_COLLAB_5527_rep_01_flowthru_03	5814	FTMS; HCD	80.63	591.79

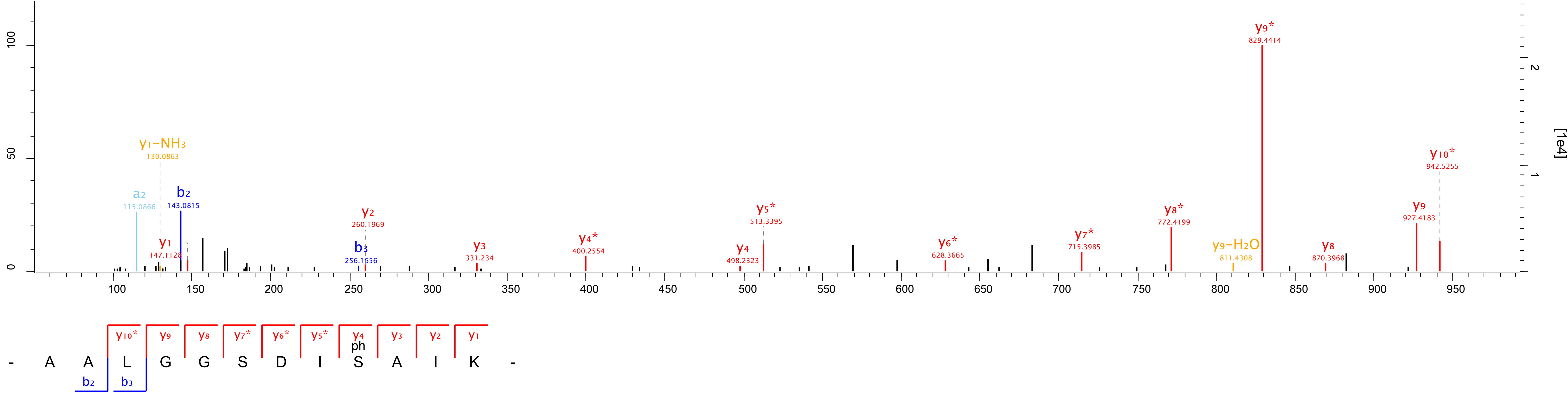

Raw file	Scan	Method	Score	m/z
20101013_Velos3_NaNa_COLLAB_5527_rep_03_fraction01	4077	FTMS; HCD	56.04	513.26

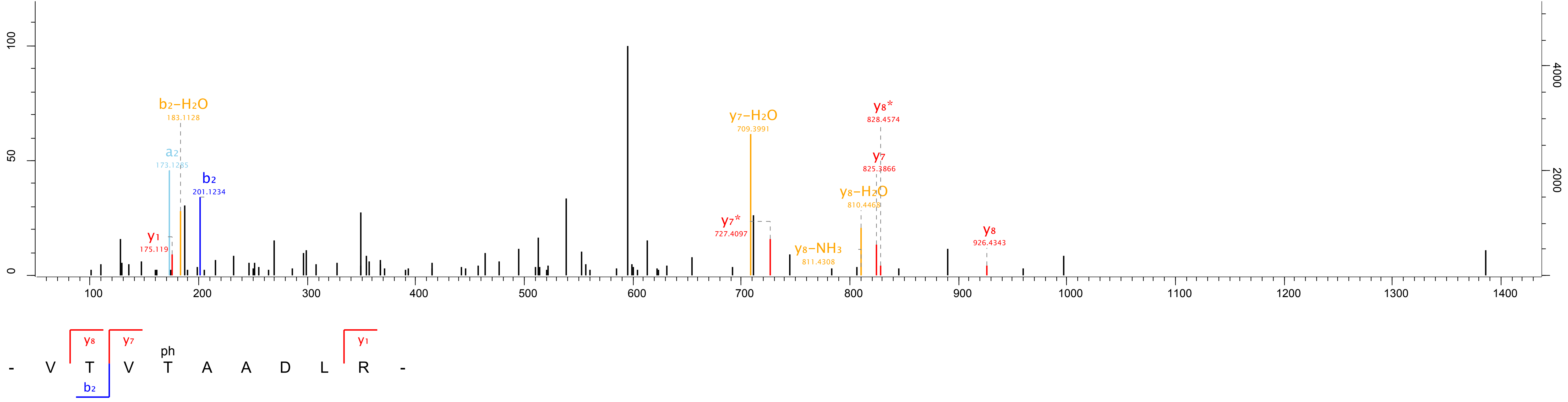

Raw file	Scan	Method	Score	m/z
20101013_Velos3_NaNa_COLLAB_5527_rep_03_fraction03	4751	FTMS; HCD	138.55	594.28

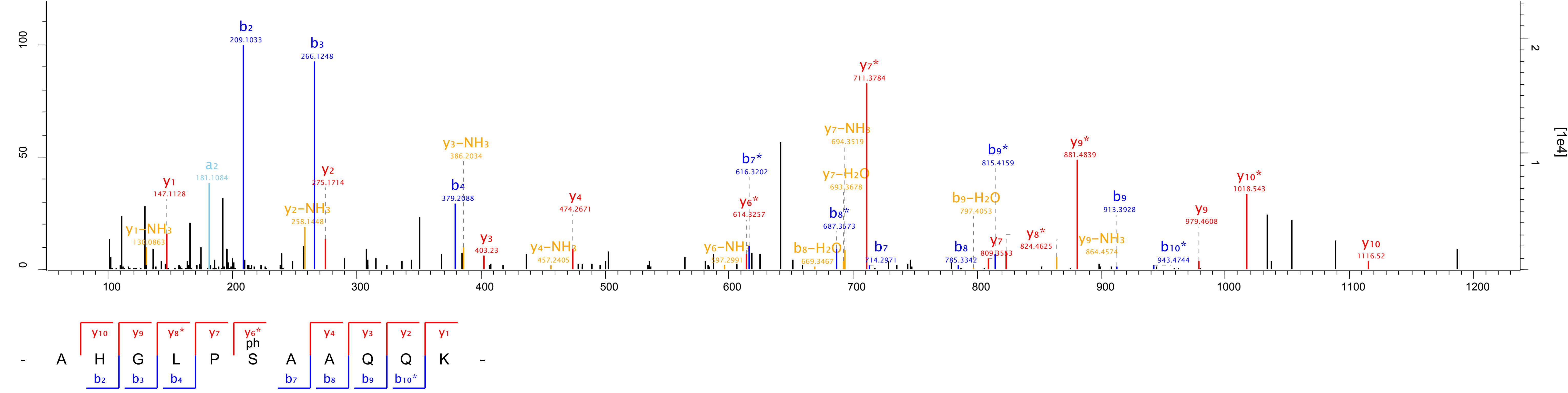

Raw file	Scan	Method	Score	m/z
20101013_Velos3_NaNa_COLLAB_salvage_5527_02	12707	FTMS; HCD	102.5	691.83

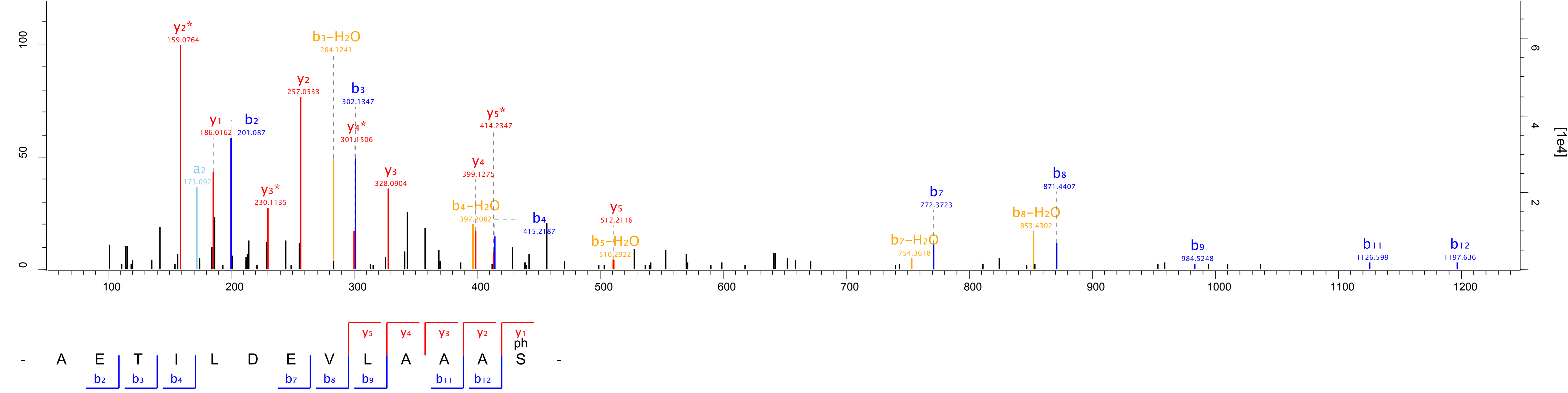

Raw file	Scan	Method	Score	m/z
20101013_Velos3_NaNa_COLLAB_5527_rep_01_flowthru_01	8349	FTMS; HCD	57.17	584.29

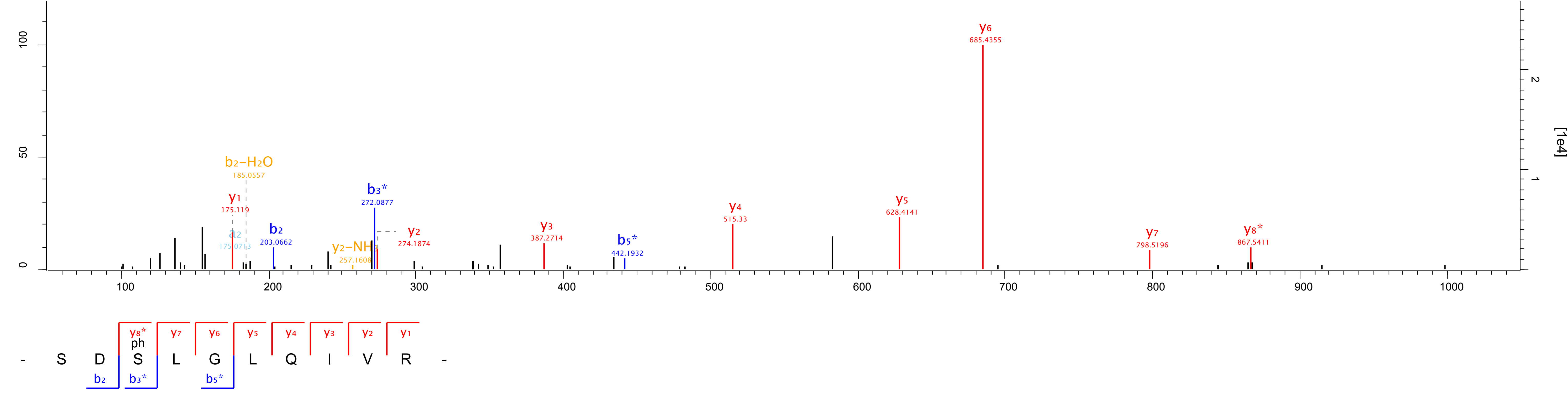

Raw file	Scan	Method	Score	m/z
20101013_Velos3_NaNa_COLLAB_5527_rep_01_flowthru_01	5297	FTMS; HCD	79.65	745.81

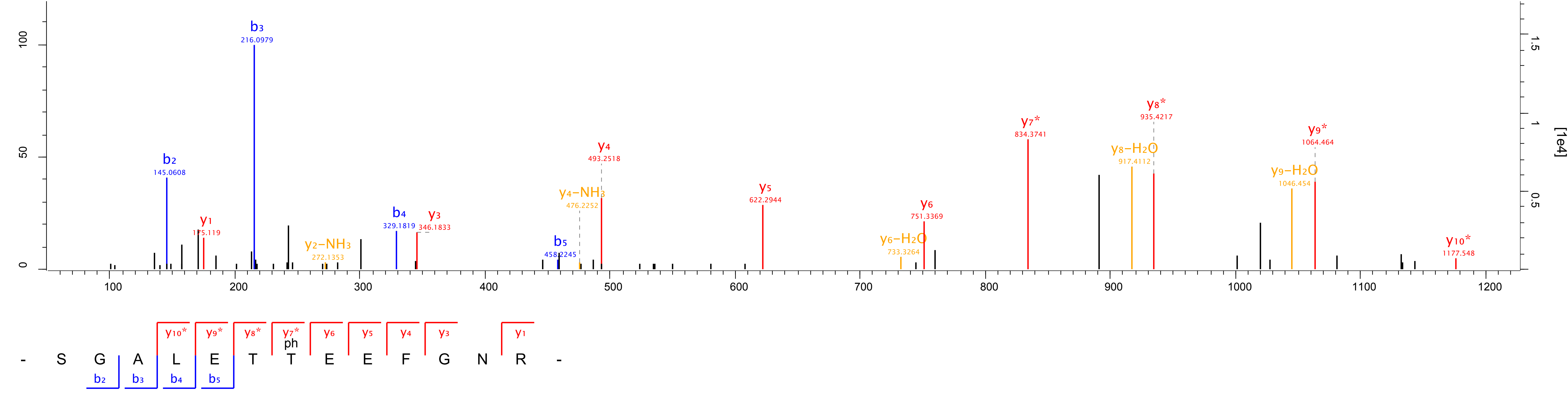

Raw file	Scan	Method	Score	m/z
20101013_Velos3_NaNa_COLLAB_5527_rep_03_fraction01	3505	FTMS; HCD	94.49	723.83

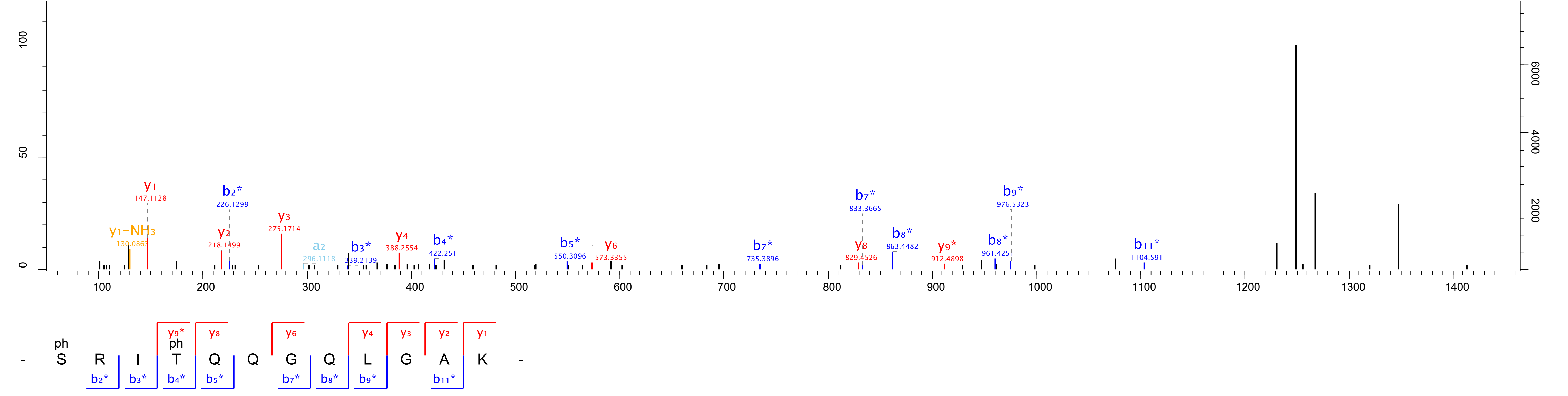

Raw file	Scan	Method	Score	m/z
20101013_Velos3_NaNa_COLLAB_5527_rep_01_fraction02	7044	FTMS; HCD	54.1	509.27

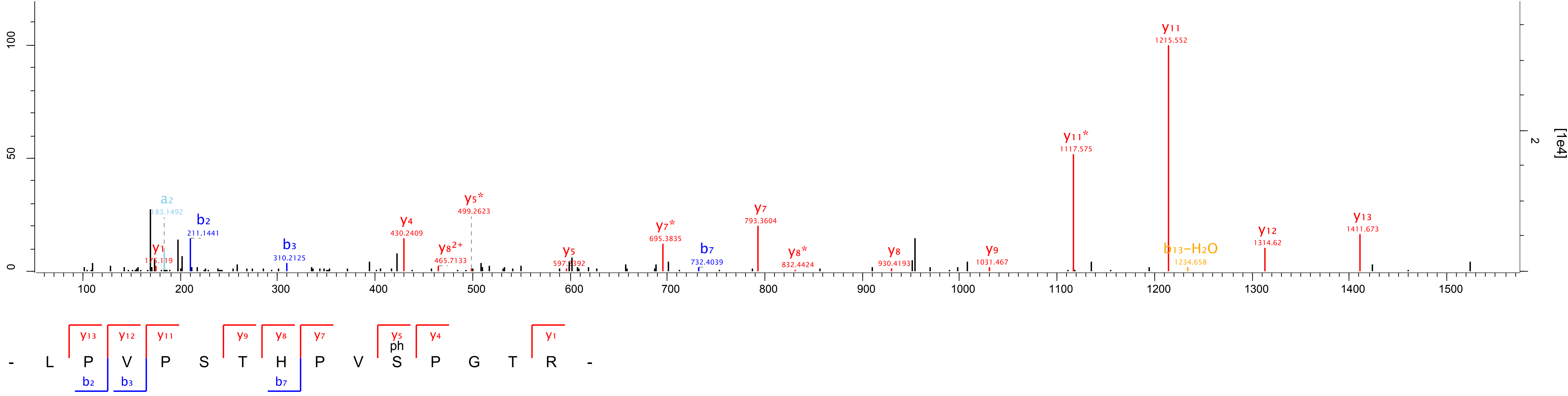

Raw file	Scan	Method	Score	m/z
20101013_Velos3_NaNa_COLLAB_salvage_5527_02	4941	FTMS; HCD	132.17	490.75

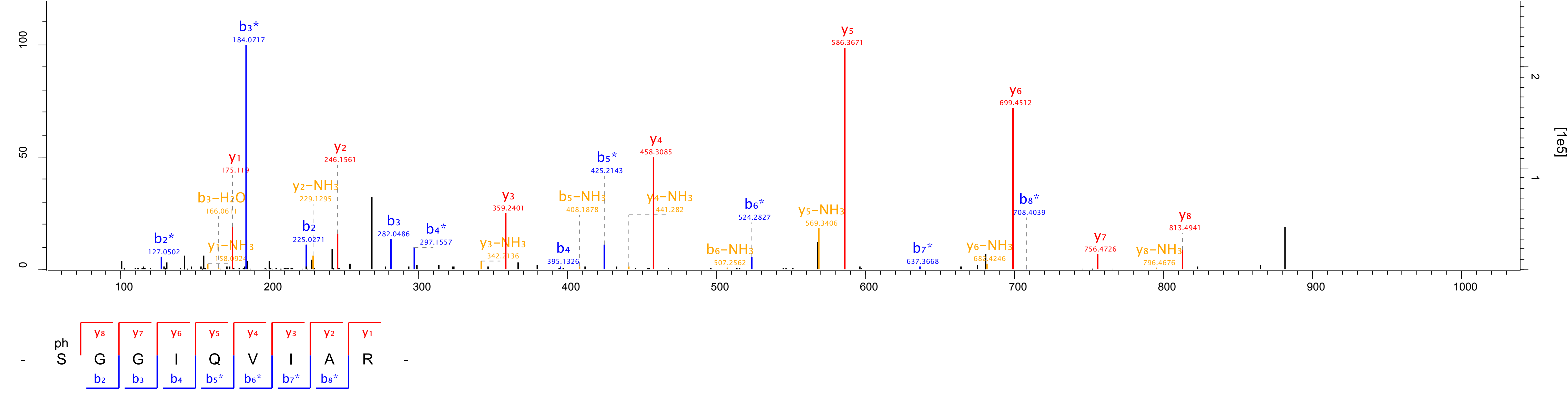

Raw file	Scan	Method	Score	m/z
20101013_Velos3_NaNa_COLLAB_5527_rep_01_flowthru_01	9102	FTMS; HCD	47.68	566.94

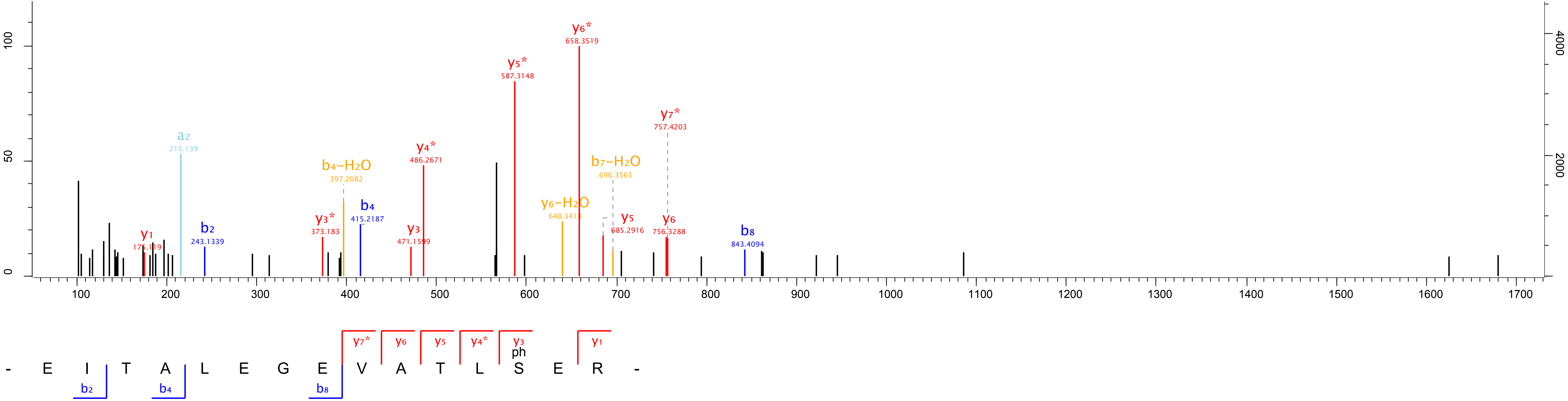

Raw file	Scan	Method	Score	m/z
20101013_Velos3_NaNa_COLLAB_salvage_5527_02	3027	FTMS; HCD	88.28	593.26

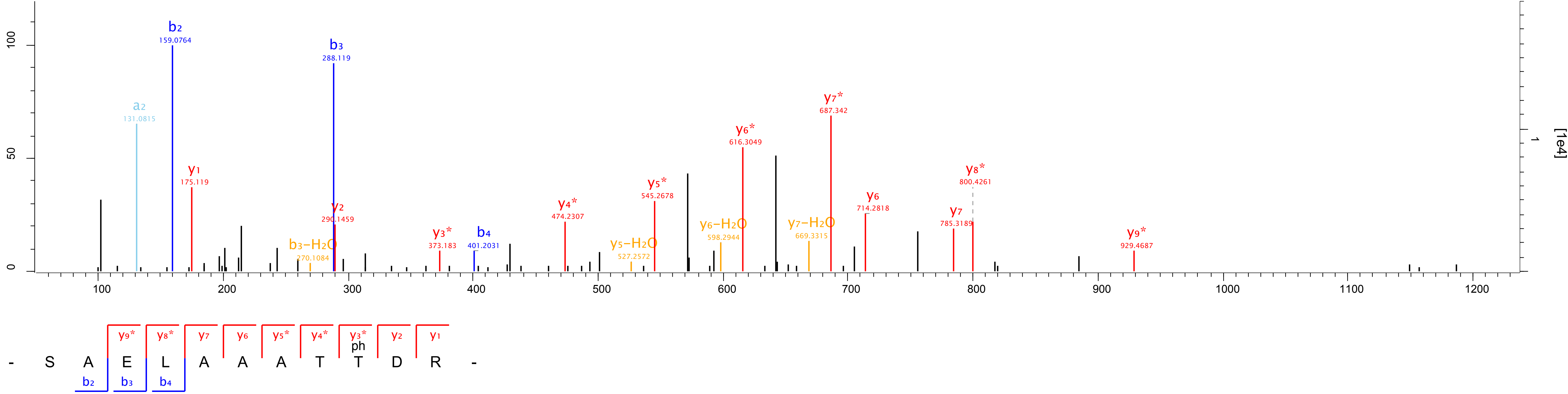

Raw file	Scan	Method	Score	m/z
20101013_Velos3_NaNa_COLLAB_5527_rep_01_flowthru_01	11898	FTMS; HCD	56.08	894.13

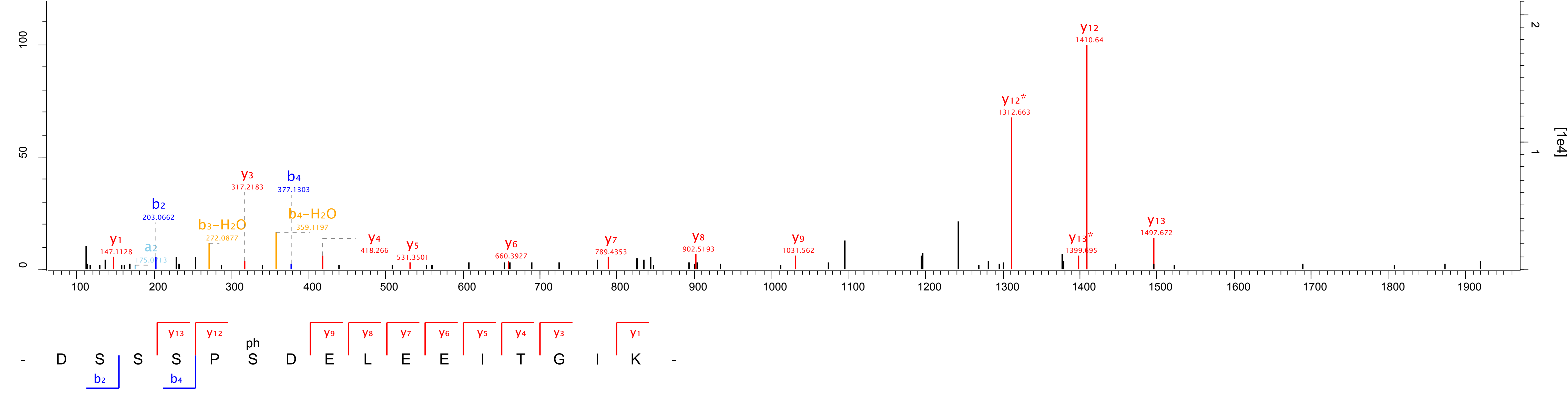

Raw file	Scan	Method	Score	m/z
20101013_Velos3_NaNa_COLLAB_5527_rep_03_fraction01	9899	FTMS; HCD	57.29	677.84

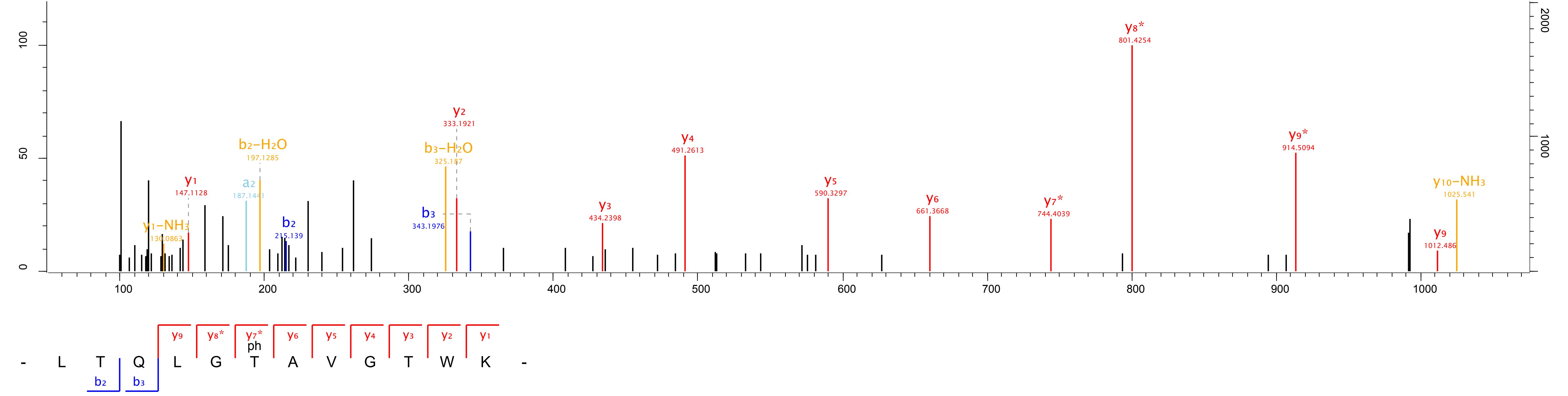

Raw file	Scan	Method	Score	m/z
20101013_Velos3_NaNa_COLLAB_5527_rep_01_flowthru_01	8276	FTMS; HCD	64.55	639.93

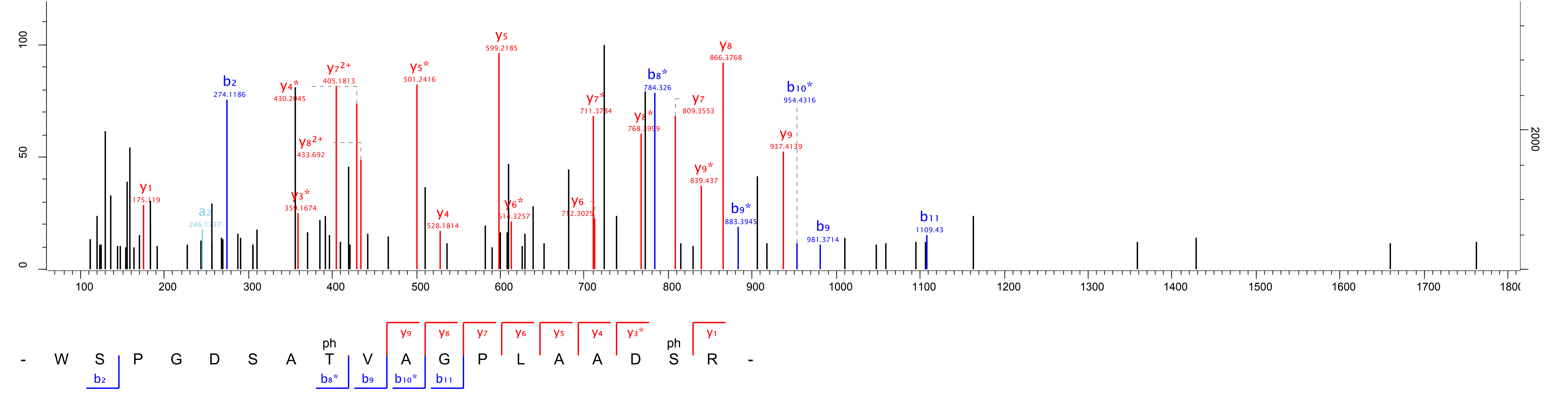

Raw file	Scan	Method	Score	m/z
20101013_Velos3_NaNa_COLLAB_5527_rep_01_flowthru_03	6198	FTMS; HCD	127.18	699.83

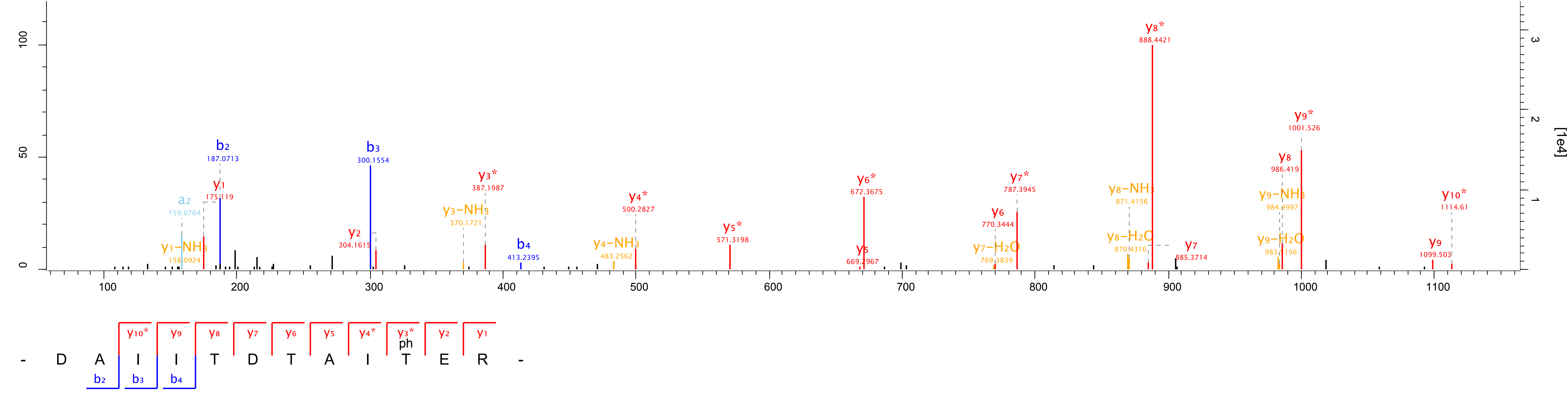

Raw file	Scan	Method	Score	m/z
20101013_Velos3_NaNa_COLLAB_5527_rep_03_flowthru_01	4632	FTMS; HCD	91.08	988.41

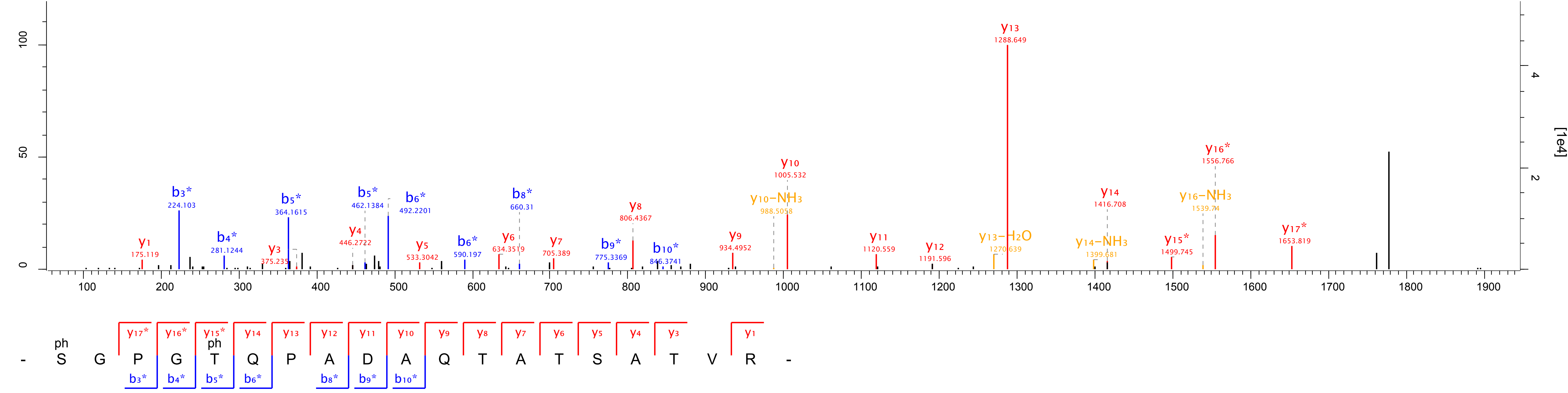

Raw file	Scan	Method	Score	m/z
20101013_Velos3_NaNa_COLLAB_5527_rep_03_flowthru_01	7472	FTMS; HCD	83.18	589.75

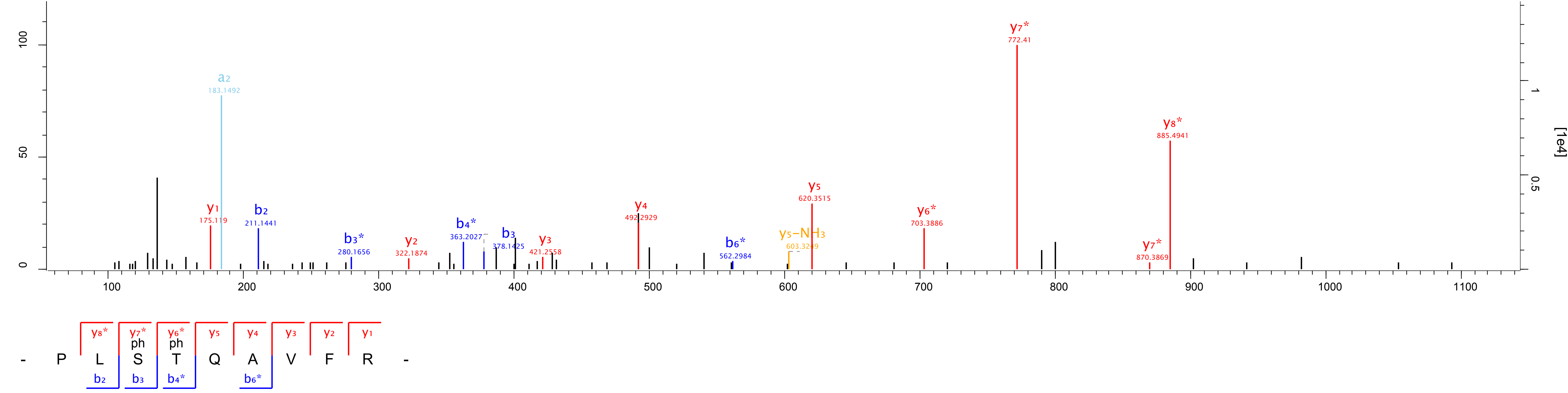

Raw file	Scan	Method	Score	m/z
20101013_Velos3_NaNa_COLLAB_5527_rep_01_fraction04	9875	FTMS; HCD	53.03	623.3

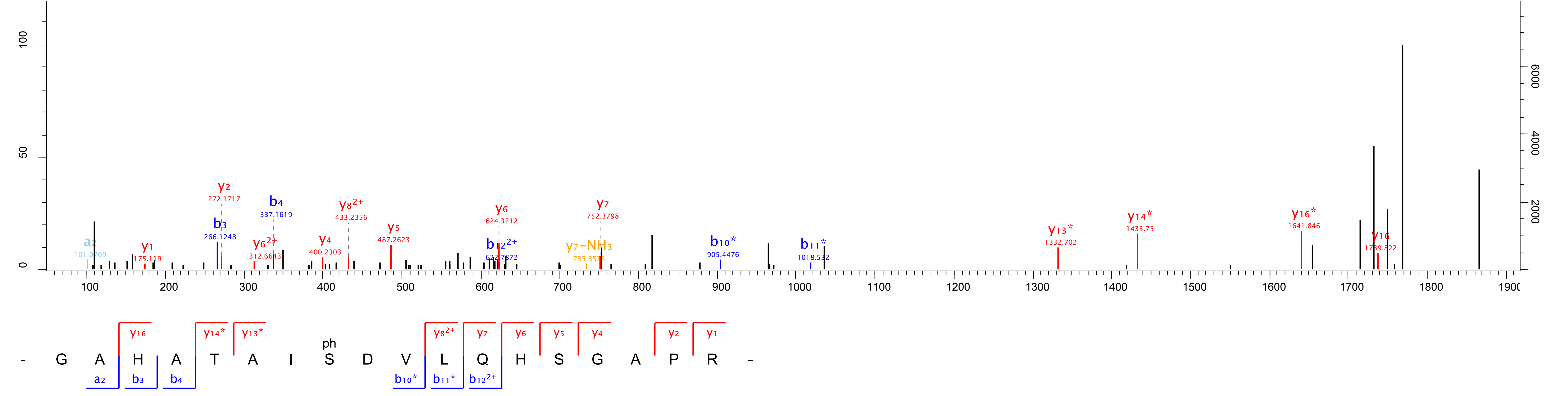

Raw file	Scan	Method	Score	m/z
20101013_Velos3_NaNa_COLLAB_5527_rep_01_flowthru_01	3975	FTMS; HCD	83.09	606.27

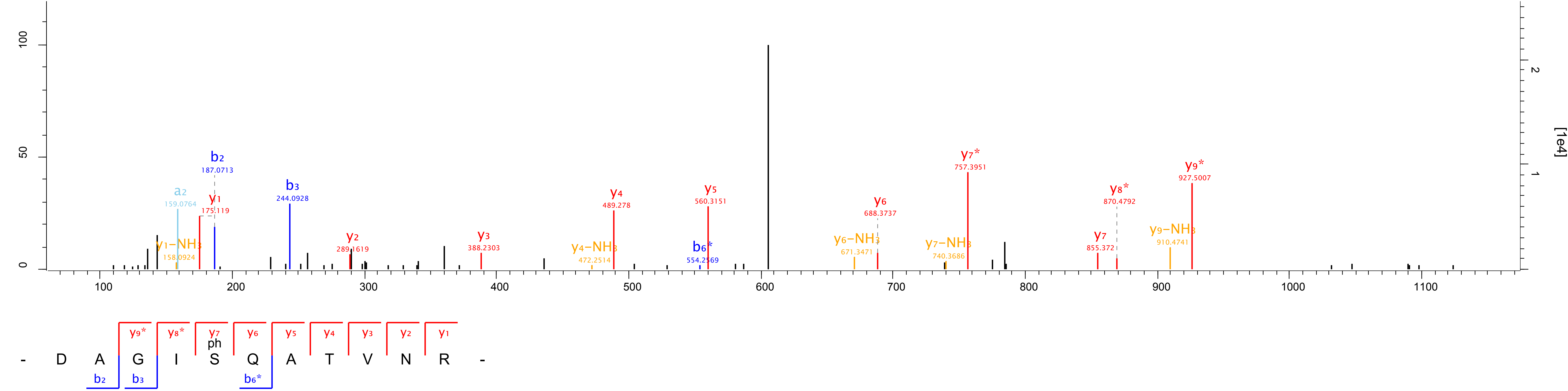

Raw file	Scan	Method	Score	m/z
20101013_Velos3_NaNa_COLLAB_5527_rep_01_flowthru_03	4014	FTMS; HCD	83.54	576.26

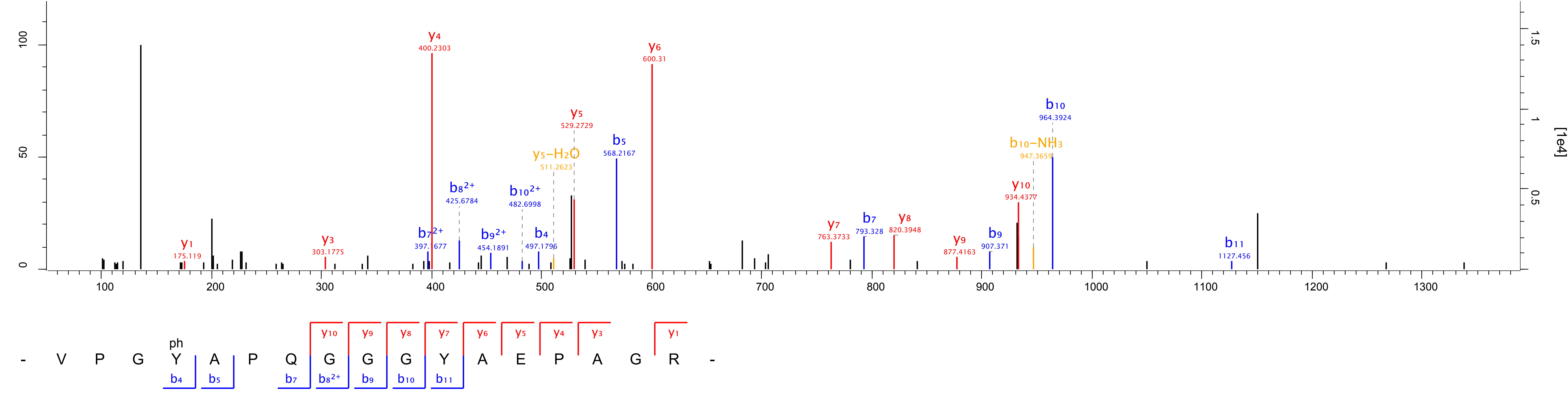

Raw file	Scan	Method	Score	m/z
20101013_Velos3_NaNa_COLLAB_salvage_5527_02	3091	FTMS; HCD	139.94	641.74

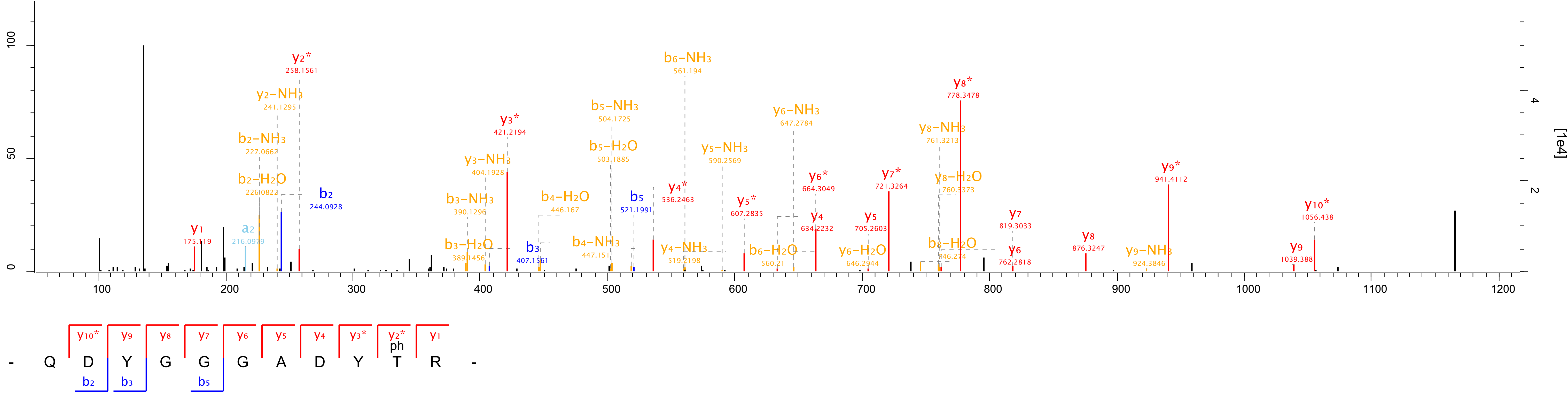

Raw file	Scan	Method	Score	m/z
20101013_Velos3_NaNa_COLLAB_5527_rep_03_flowthru_01	2950	FTMS; HCD	58.17	641.74

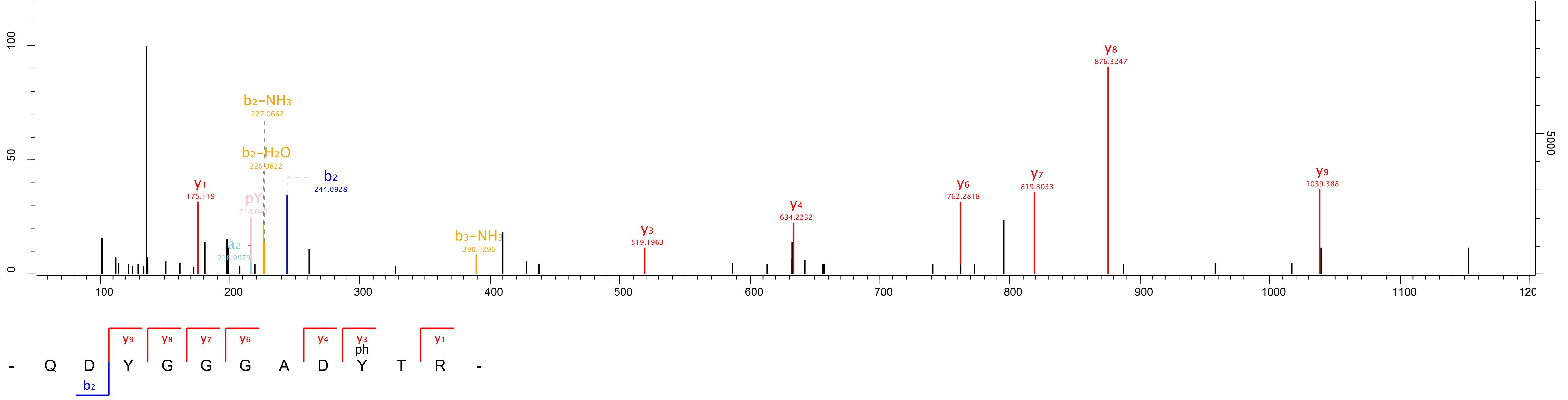

Raw file	Scan	Method	Score	m/z
20101013_Velos3_NaNa_COLLAB_5527_rep_01_flowthru_03	5379	FTMS; HCD	107.75	825.34

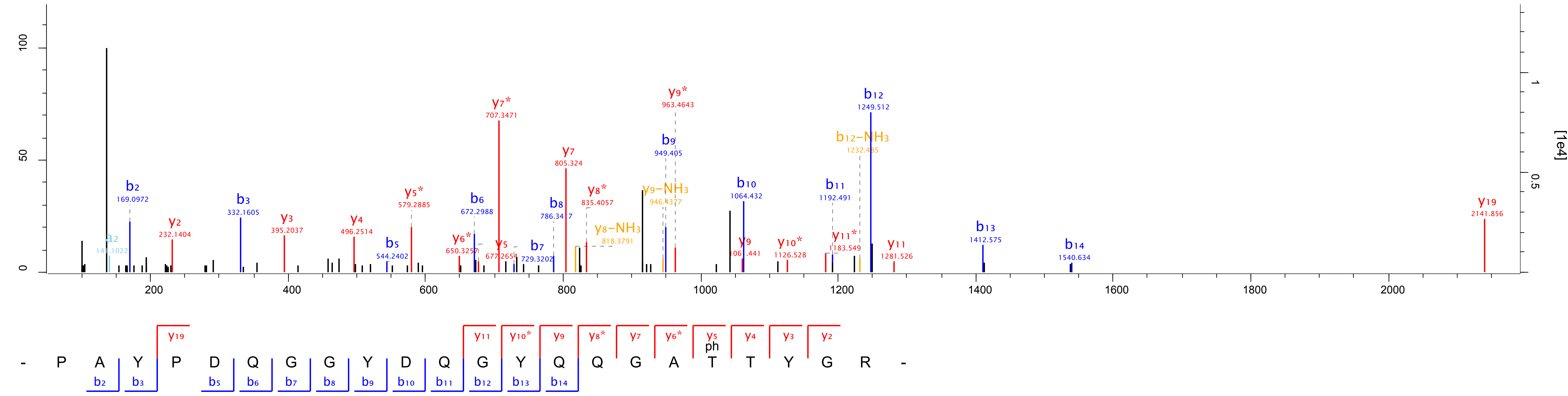

Raw file	Scan	Method	Score	m/z
20101013_Velos3_NaNa_COLLAB_5527_rep_03_fraction02	8272	FTMS; HCD	64.59	909.71

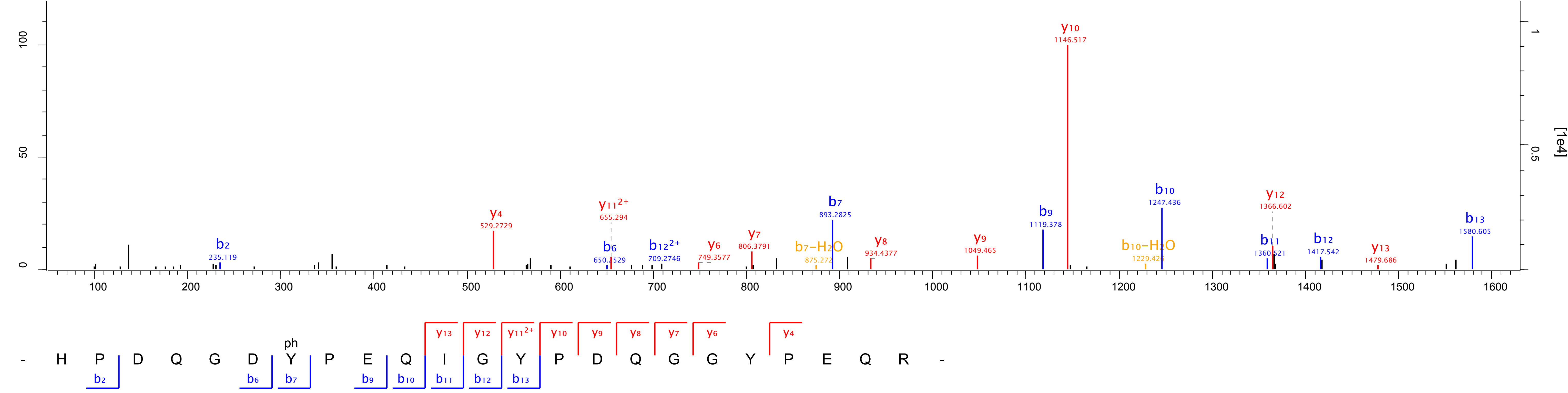

Raw file	Scan	Method	Score	m/z
20101013_Velos3_NaNa_COLLAB_5527_rep_03_fraction03	9011	FTMS; HCD	124.2	1000.43

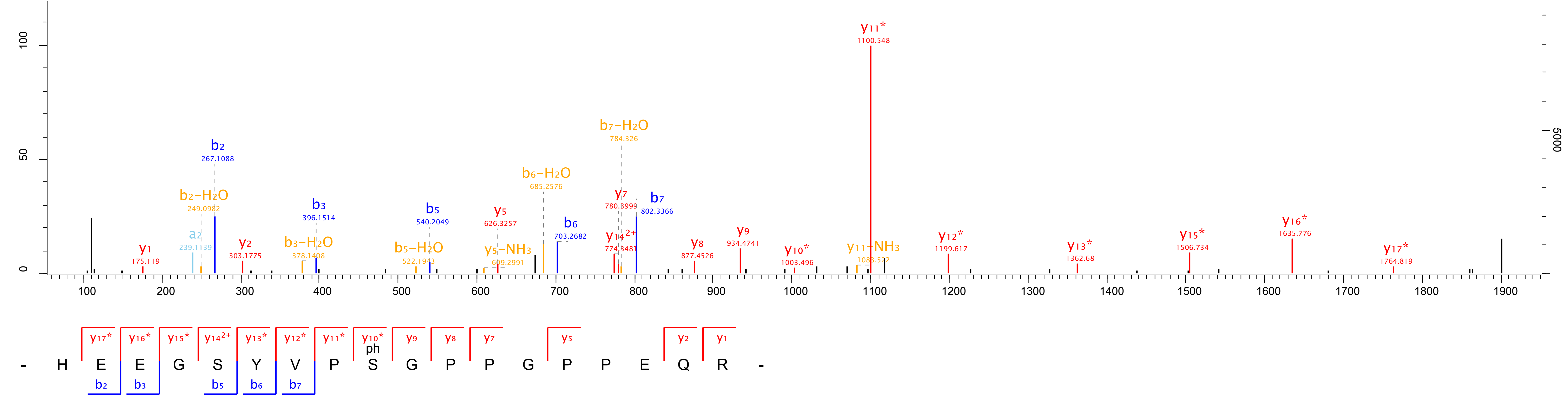

Raw file	Scan	Method	Score	m/z
20101013_Velos3_NaNa_COLLAB_5527_rep_03_fraction02	8428	FTMS; HCD	83.4	686.65

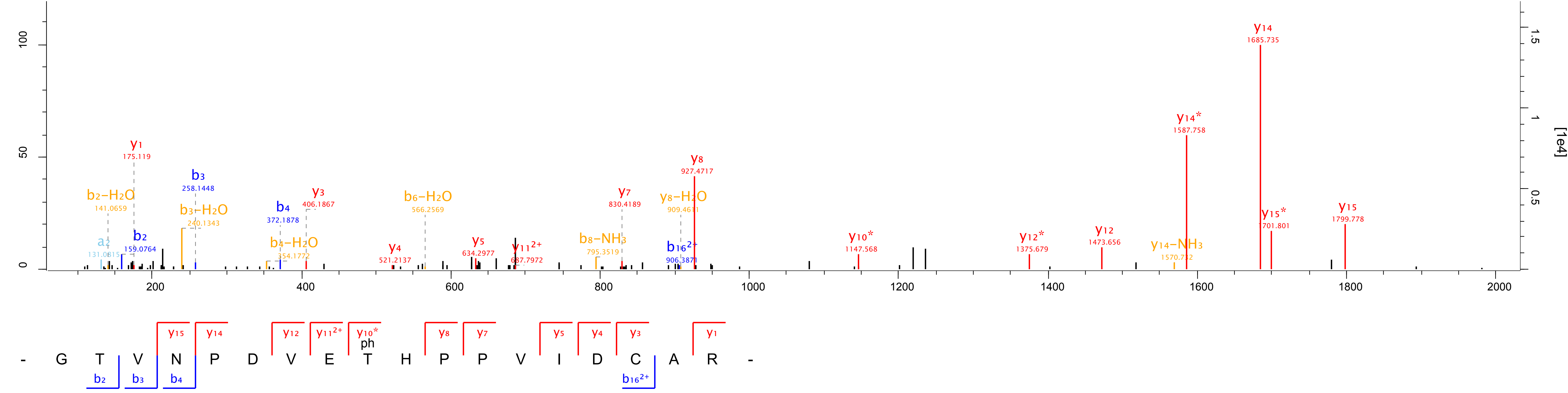

Raw file	Scan	Method	Score	m/z
20101013_Velos3_NaNa_COLLAB_5527_rep_01_fraction03	4925	FTMS; HCD	127.57	598.24

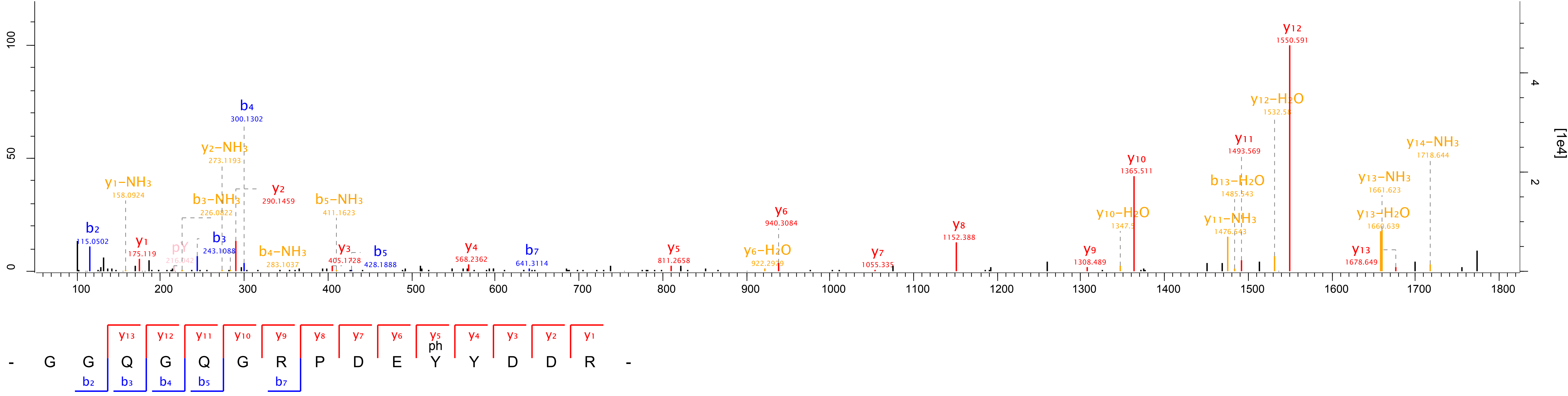

Raw file	Scan	Method	Score	m/z
20101013_Velos3_NaNa_COLLAB_salvage_5527_03	2008	FTMS; HCD	42.24	610.24

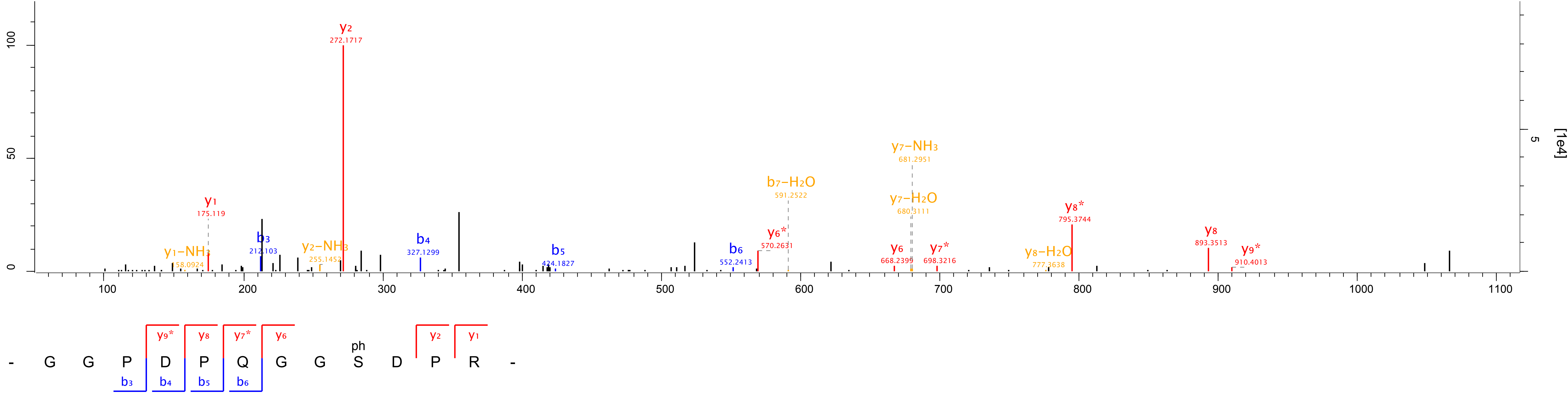

Raw file	Scan	Method	Score	m/z
20101013_Velos3_NaNa_COLLAB_salvage_5527_03	2319	FTMS; HCD	118.19	494.56

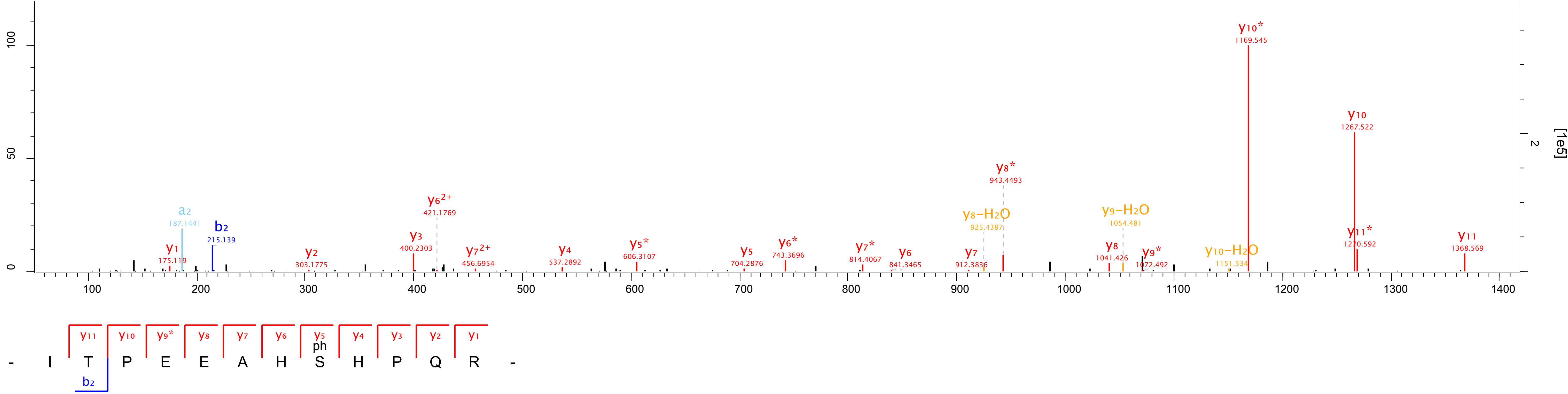

Raw file	Scan	Method	Score	m/z
20101013_Velos3_NaNa_COLLAB_5527_rep_03_fraction03	9171	FTMS; HCD	60.06	510.58

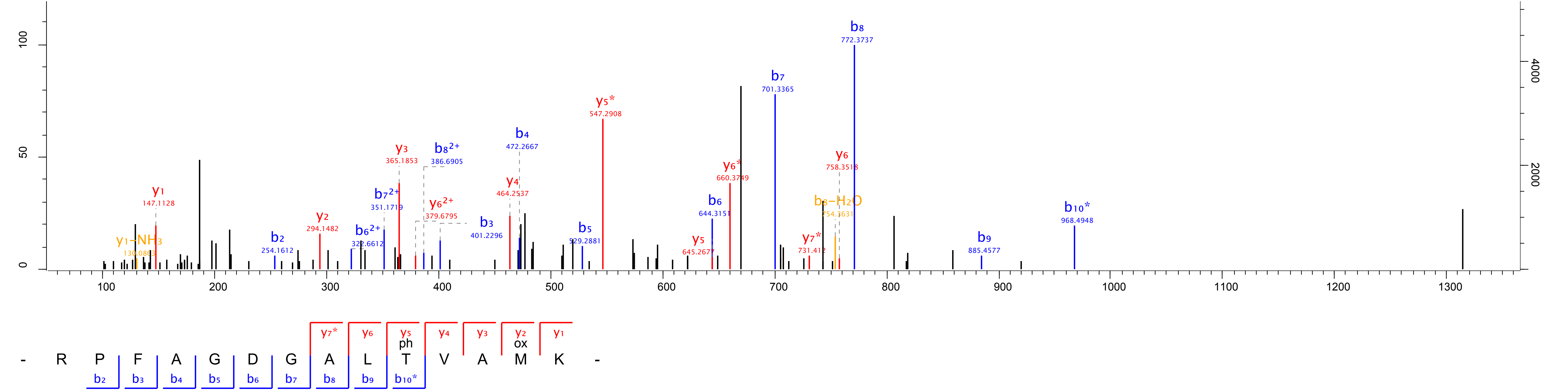

Raw file	Scan	Method	Score	m/z
20101013_Velos3_NaNa_COLLAB_5527_rep_03_flowthru_01	4880	FTMS; HCD	57.17	474.7

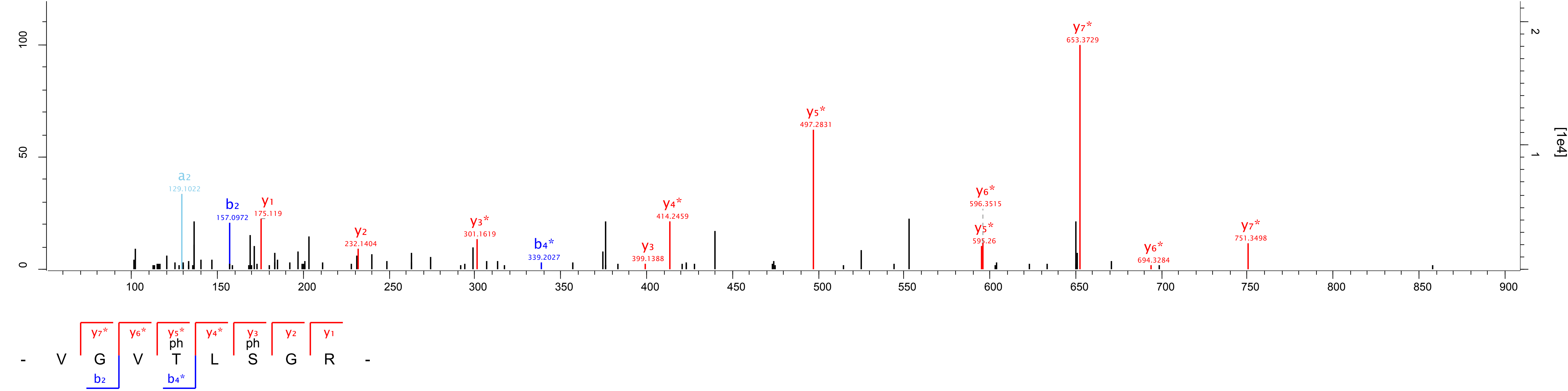

Raw file	Scan	Method	Score	m/z
20101013_Velos3_NaNa_COLLAB_5527_rep_01_fraction02	13724	FTMS; HCD	154.69	731.04

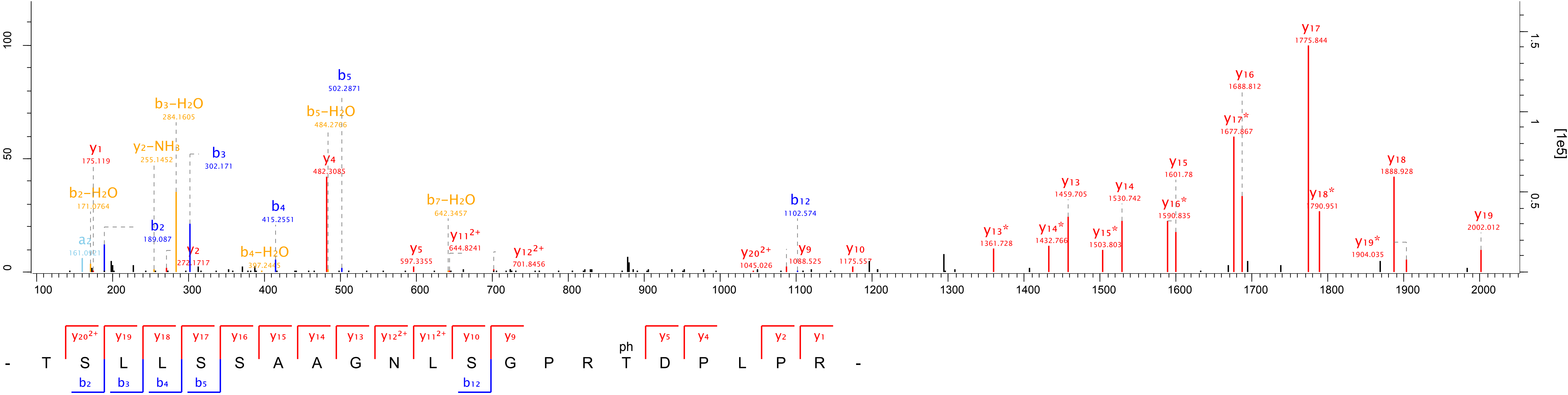

Raw file	Scan	Method	Score	m/z
20101013_Velos3_NaNa_COLLAB_5527_rep_03_fraction01	8058	FTMS; HCD	71.18	483.74

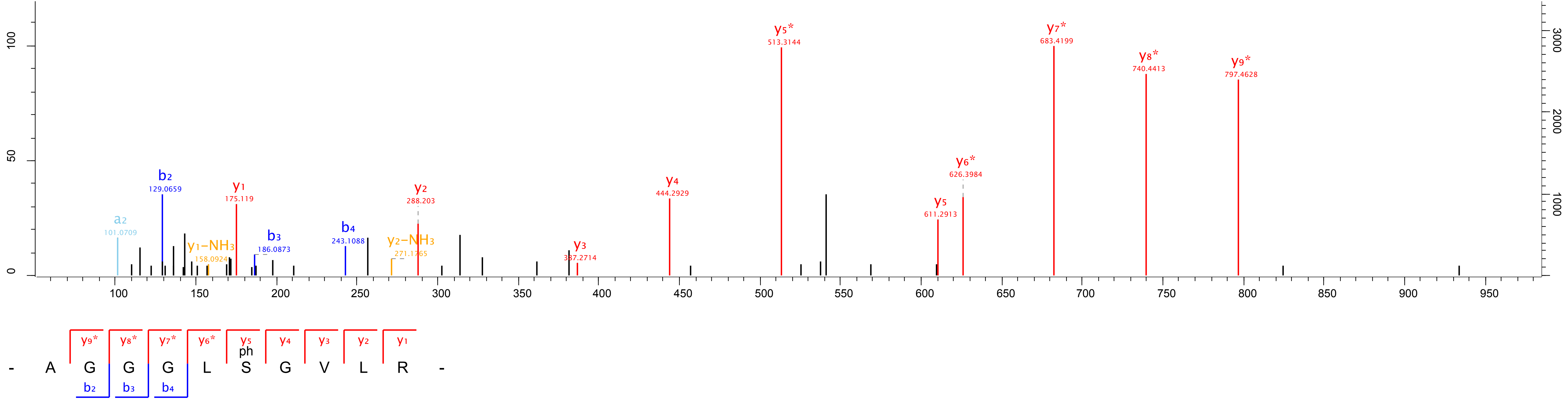

Raw file	Scan	Method	Score	m/z
20101013_Velos3_NaNa_COLLAB_5527_rep_01_flowthru_01	9373	FTMS; HCD	93.37	957.43

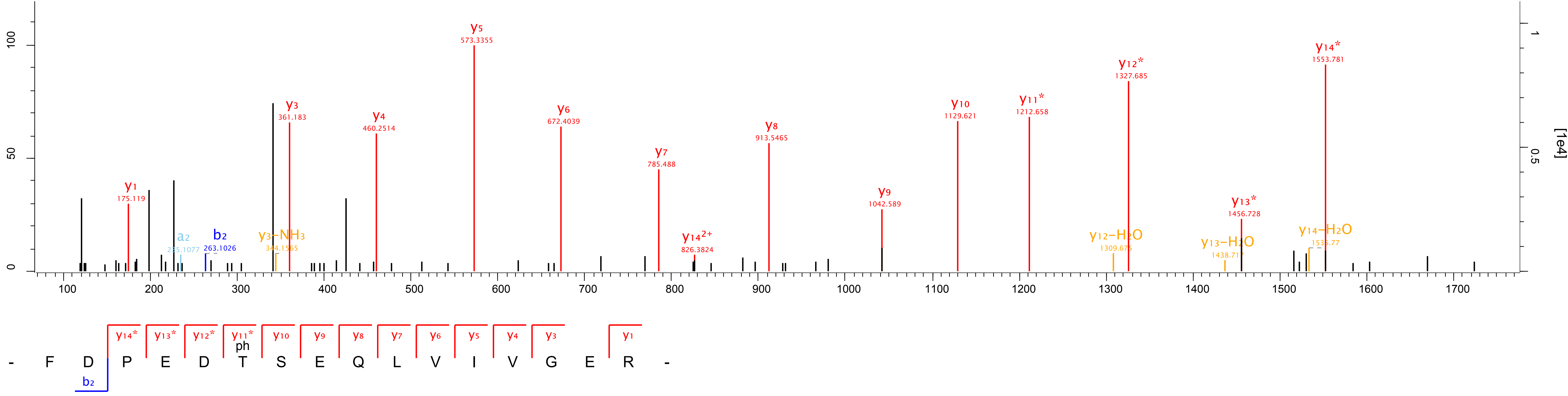

Raw file	Scan	Method	Score	m/z
20101013_Velos3_NaNa_COLLAB_5527_rep_02_fraction02	5623	FTMS; HCD	135.72	502.58

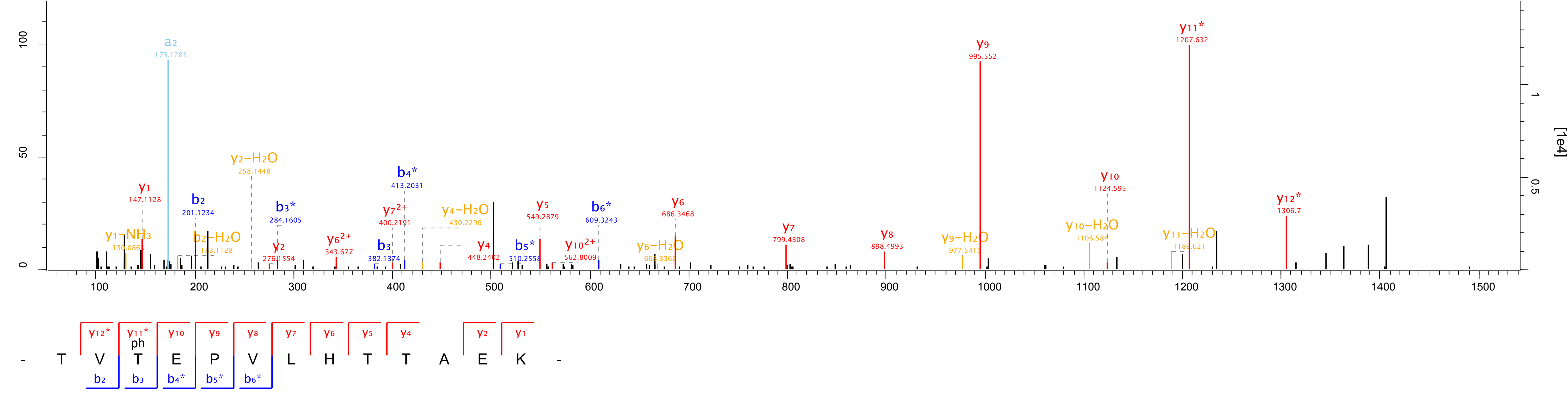

Raw file	Scan	Method	Score	m/z
20101013_Velos3_NaNa_COLLAB_5527_rep_01_flowthru_01	5082	FTMS; HCD	66.99	662.78

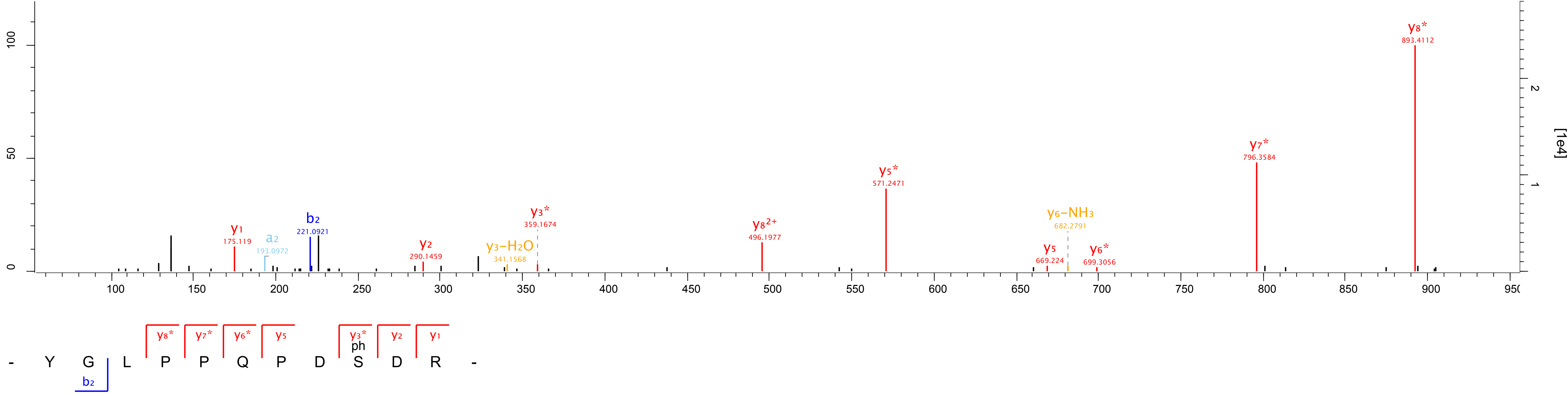

Raw file	Scan	Method	Score	m/z
20101013_Velos3_NaNa_COLLAB_salvage_5527_02	4873	FTMS; HCD	64.27	545.23

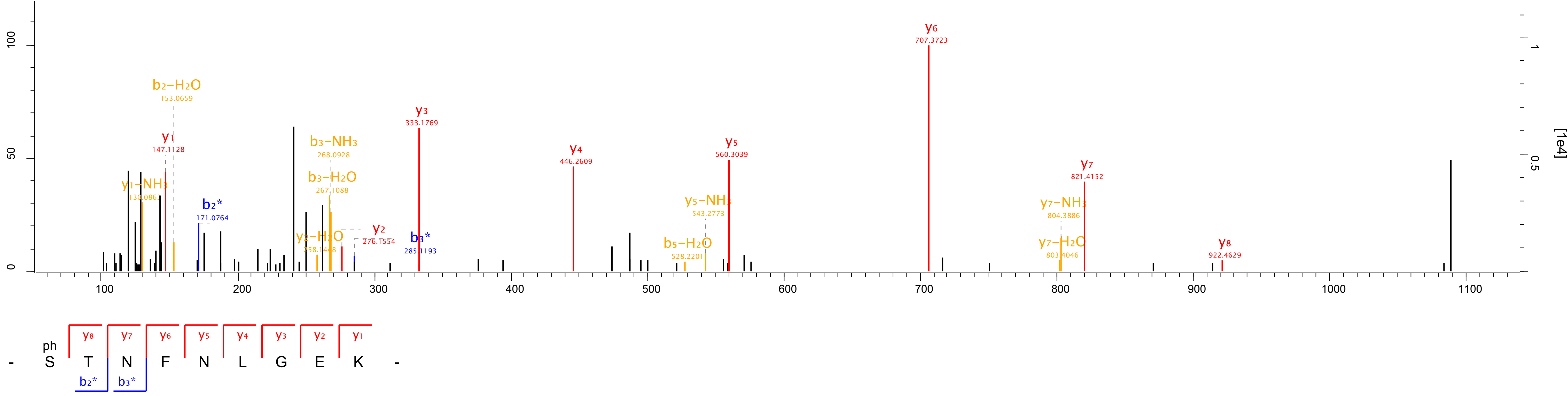

Raw file	Scan	Method	Score	m/z
20101013_Velos3_NaNa_COLLAB_5527_rep_01_flowthru_01	5873	FTMS; HCD	84.36	622.76

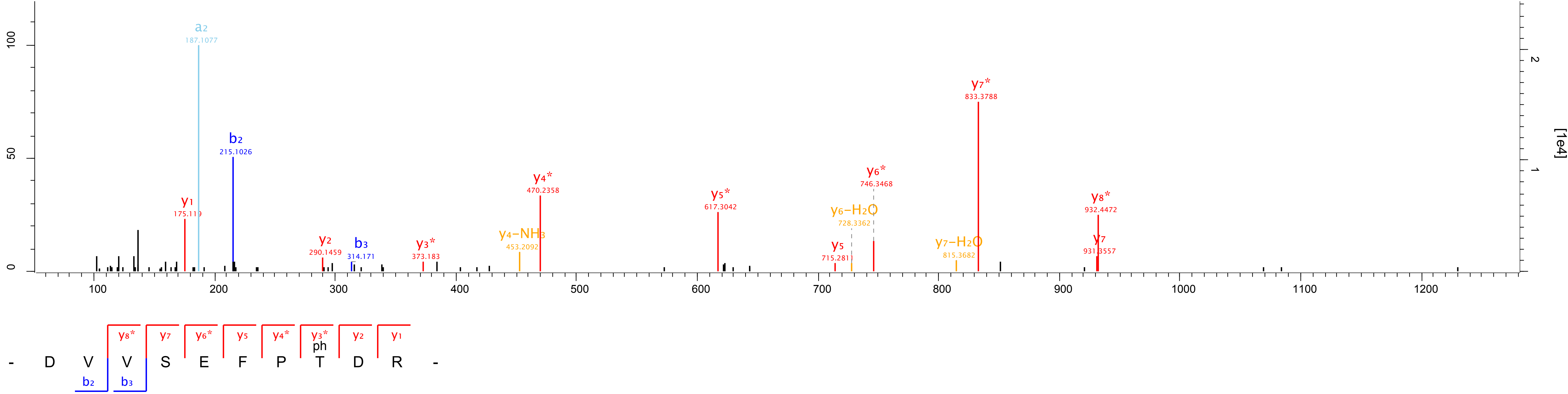

Raw file	Scan	Method	Score	m/z
20101013_Velos3_NaNa_COLLAB_salvage_5527_02	12344	FTMS; HCD	121.5	719.34

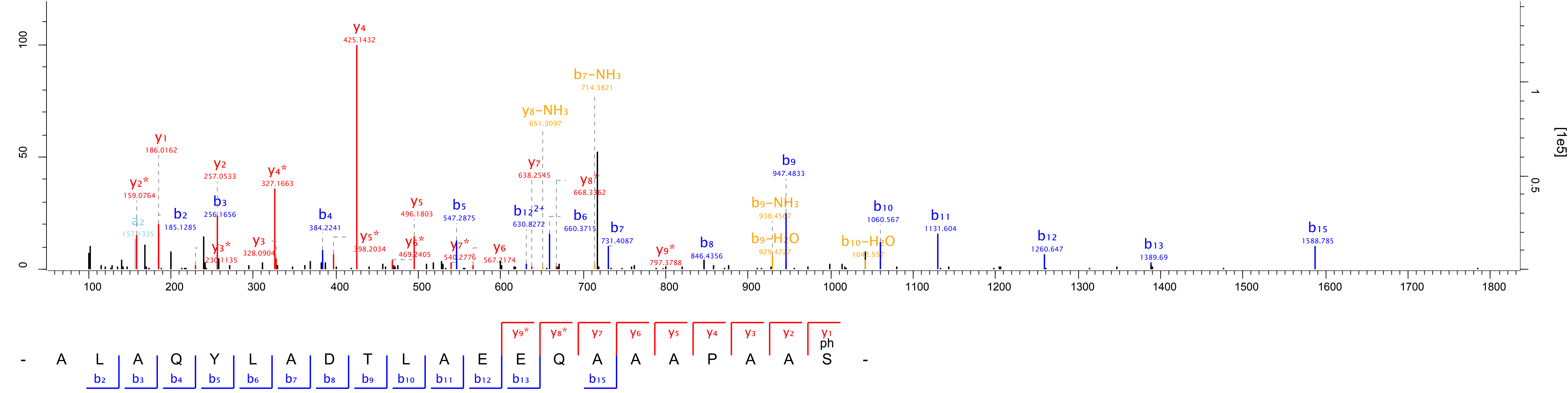

Raw file	Scan	Method	Score	m/z
20101013_Velos3_NaNa_COLLAB_5527_rep_03_fraction01	2780	FTMS; HCD	73.89	466.19

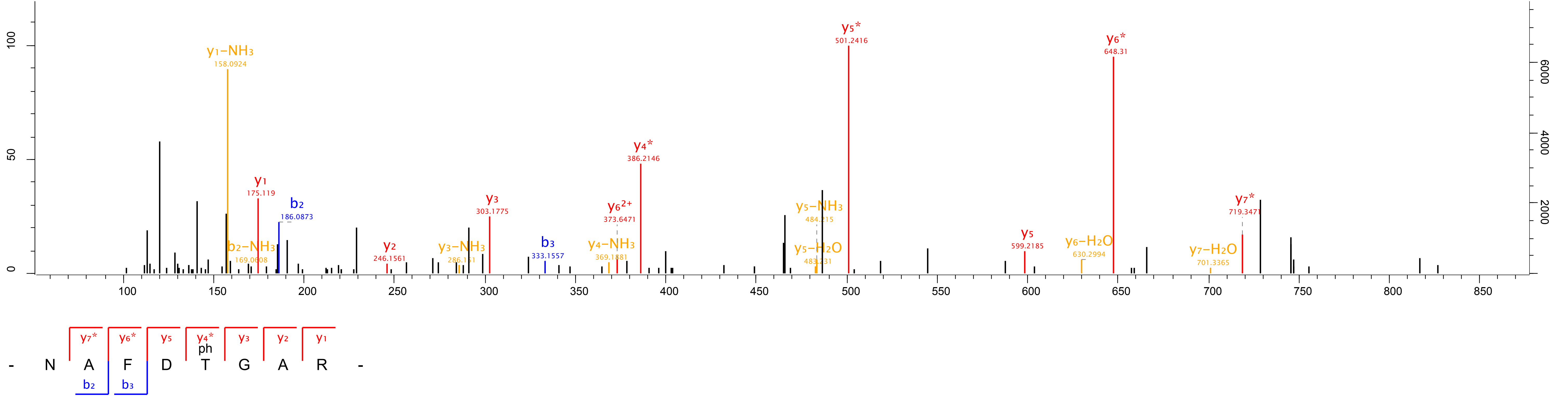

Raw file	Scan	Method	Score	m/z
20101013_Velos3_NaNa_COLLAB_5527_rep_01_fraction05	3475	FTMS; HCD	63.69	514.89

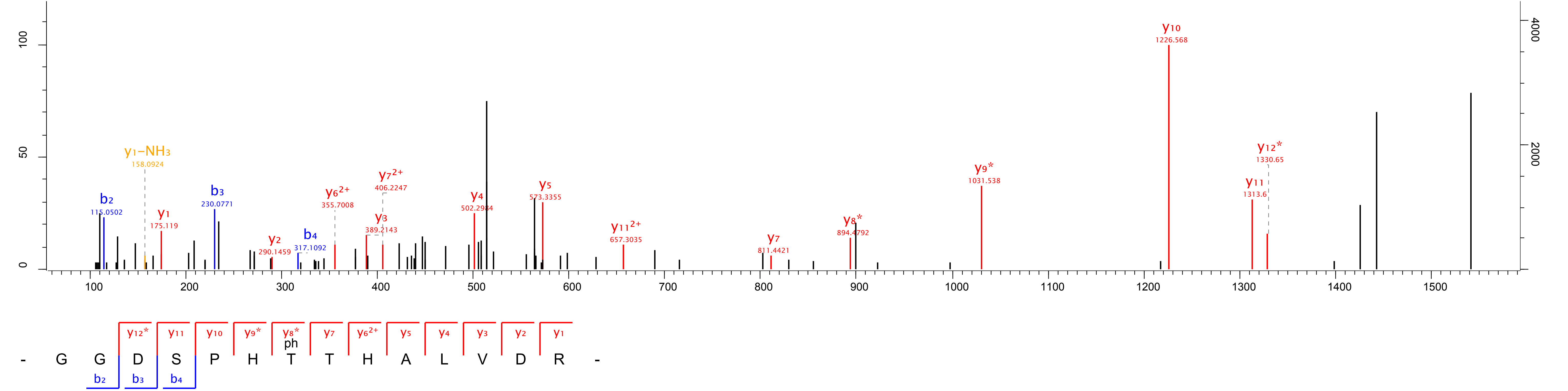

Raw file	Scan	Method	Score	m/z
20101013_Velos3_NaNa_COLLAB_salvage_5527_02	5582	FTMS; HCD	62.94	834.36

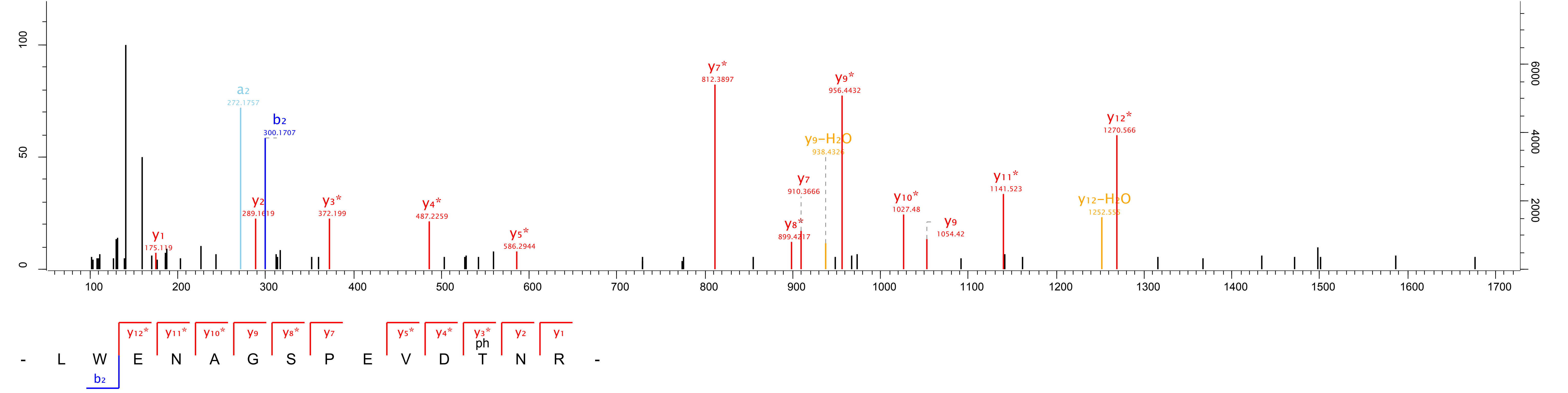

Raw file	Scan	Method	Score	m/z
20101013_Velos3_NaNa_COLLAB_5527_rep_01_flowthru_03	3863	FTMS; HCD	48.9	676.3

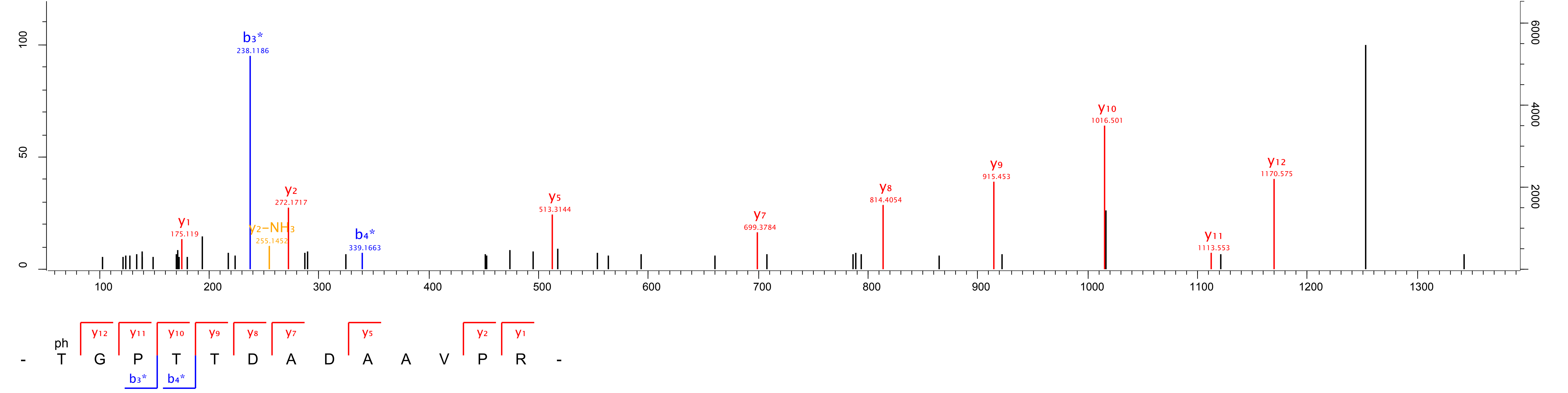

Raw file	Scan	Method	Score	m/z
20101013_Velos3_NaNa_COLLAB_5527_rep_03_fraction01	9631	FTMS; HCD	74.92	480.7

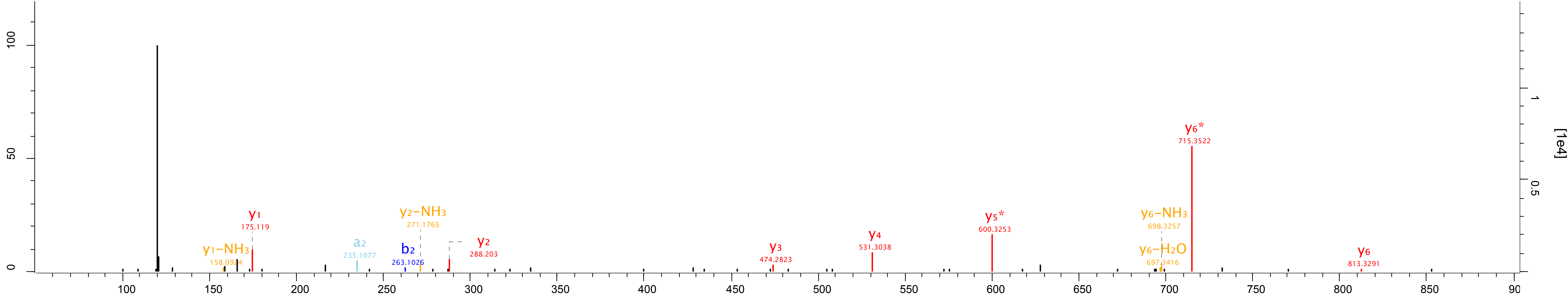

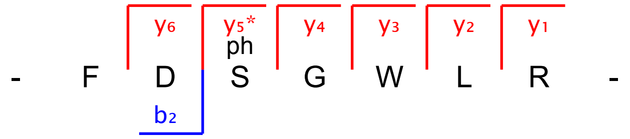

Raw file	Scan	Method	Score	m/z
20101013_Velos3_NaNa_COLLAB_5527_rep_03_flowthru_01	2766	FTMS; HCD	61.44	734.82

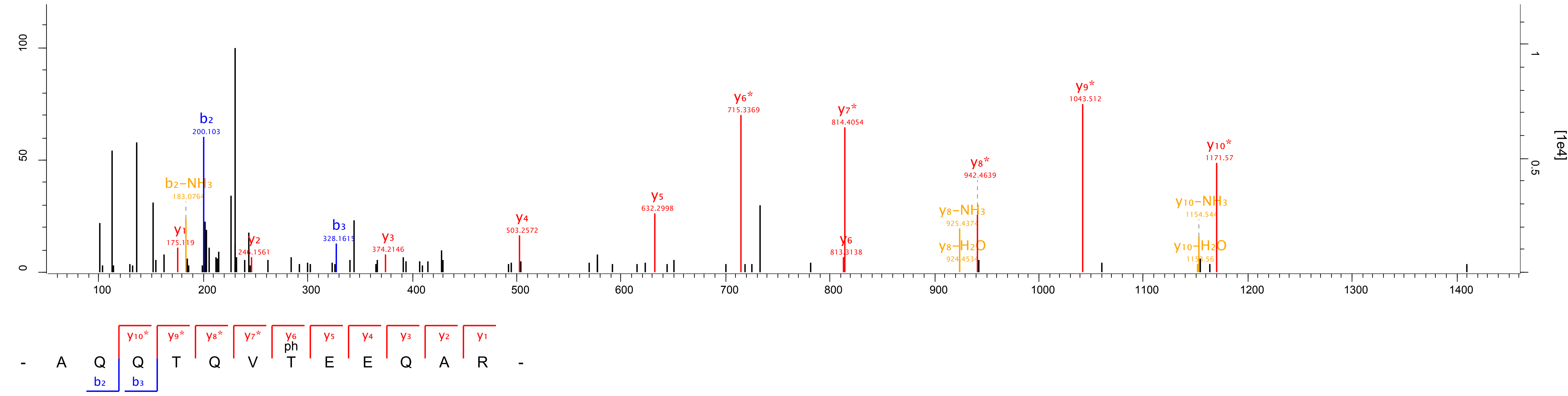

Raw file	Scan	Method	Score	m/z
20101013_Velos3_NaNa_COLLAB_5527_rep_02_fraction05	6160	FTMS; HCD	86.54	543.25

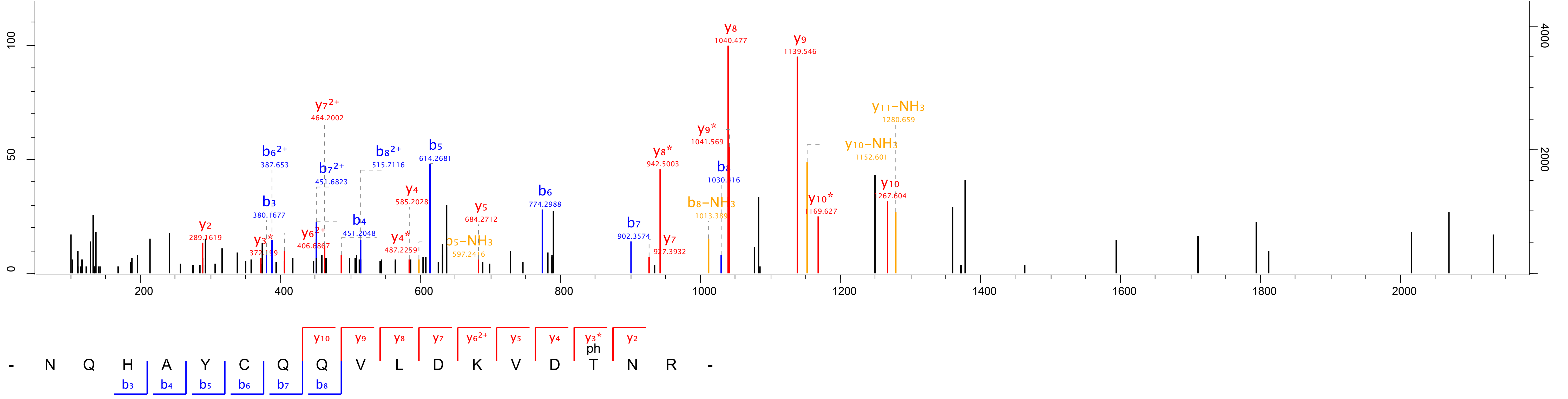

Raw file	Scan	Method	Score	m/z
20101013_Velos3_NaNa_COLLAB_salvage_5527_02	9486	FTMS; HCD	83.69	694.31

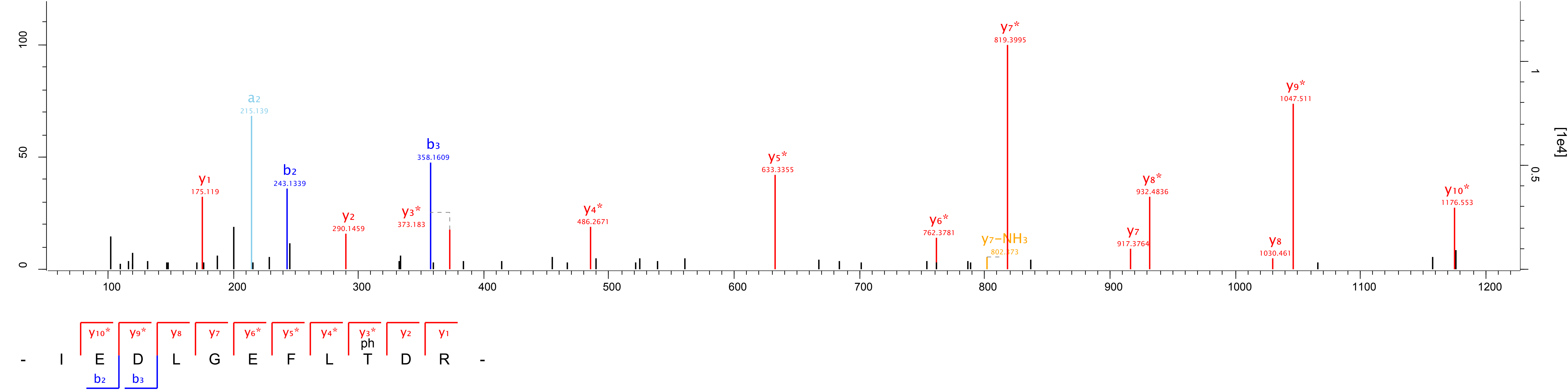

Raw file	Scan	Method	Score	m/z
20101013_Velos3_NaNa_COLLAB_5527_rep_01_fraction02	6909	FTMS; HCD	109.68	638.96

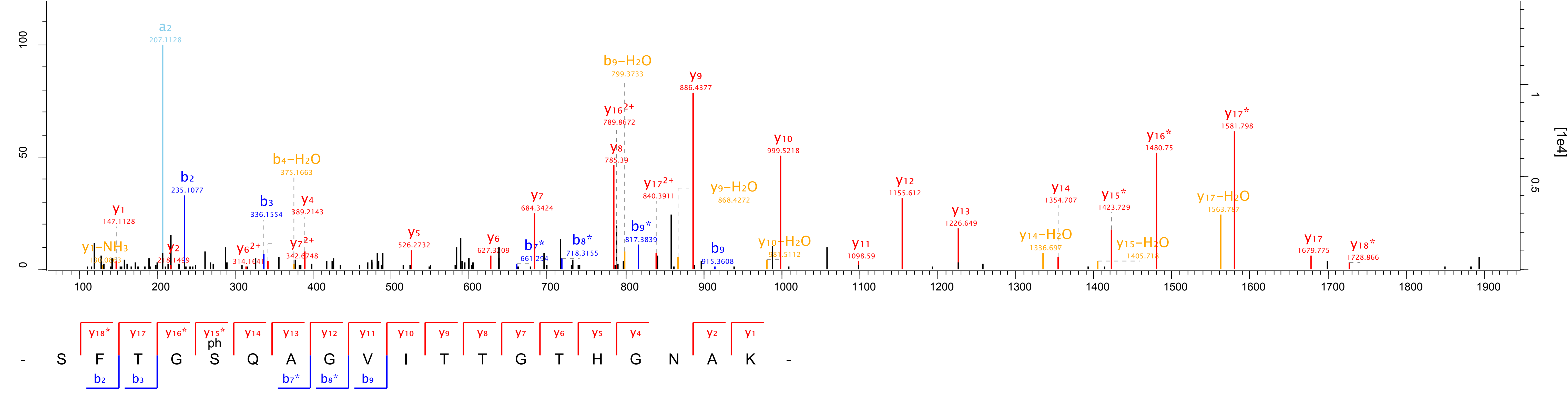

Raw file	Scan	Method	Score	m/z
20101013_Velos3_NaNa_COLLAB_salvage_5527_01	2445	FTMS; HCD	111.64	476.54

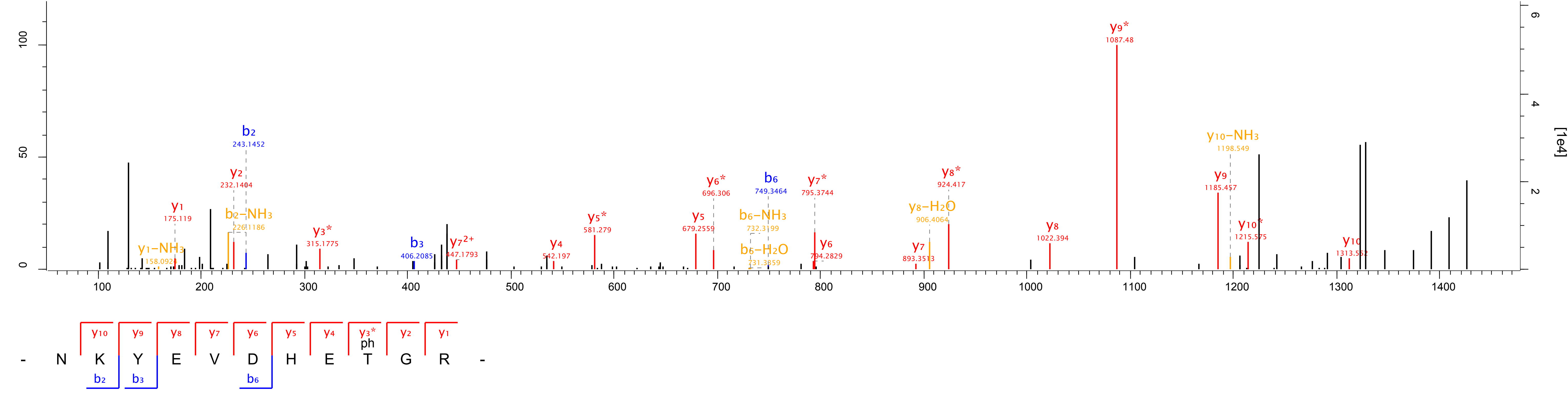

Raw file	Scan	Method	Score	m/z
20101013_Velos3_NaNa_COLLAB_5527_rep_03_fraction05	5742	FTMS; HCD	76.85	353.16

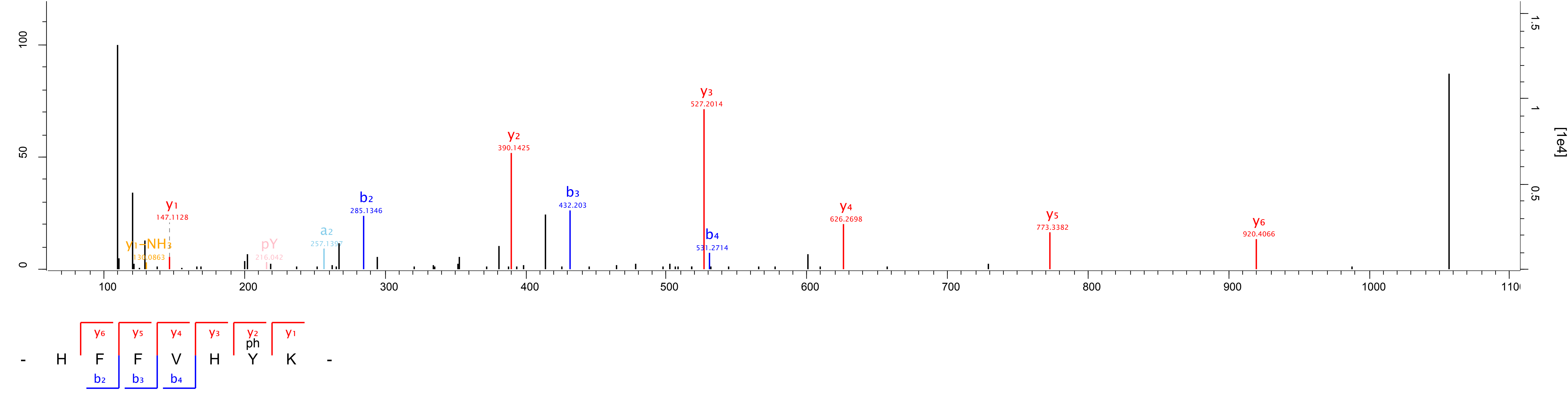

Raw file	Scan	Method	Score	m/z
20101013_Velos3_NaNa_COLLAB_5527_rep_02_flowthru_01	3683	FTMS; HCD	54.28	739.81

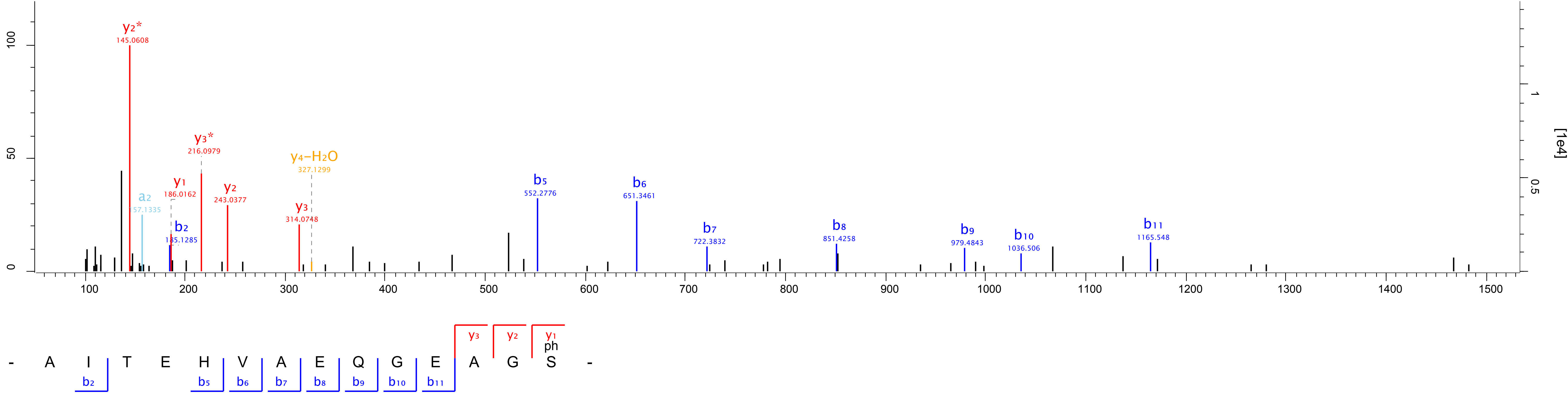

Raw file	Scan	Method	Score	m/z
20101013_Velos3_NaNa_COLLAB_5527_rep_01_flowthru_03	7093	FTMS; HCD	73.93	738.88

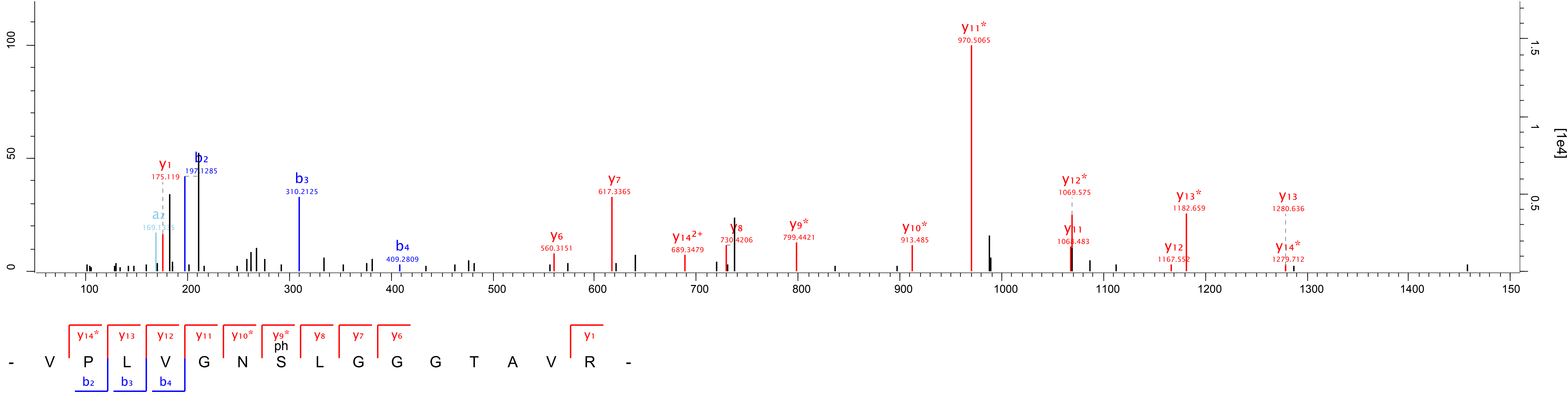

Raw file	Scan	Method	Score	m/z
20101013_Velos3_NaNa_COLLAB_salvage_5527_02	9164	FTMS; HCD	62.47	554.26

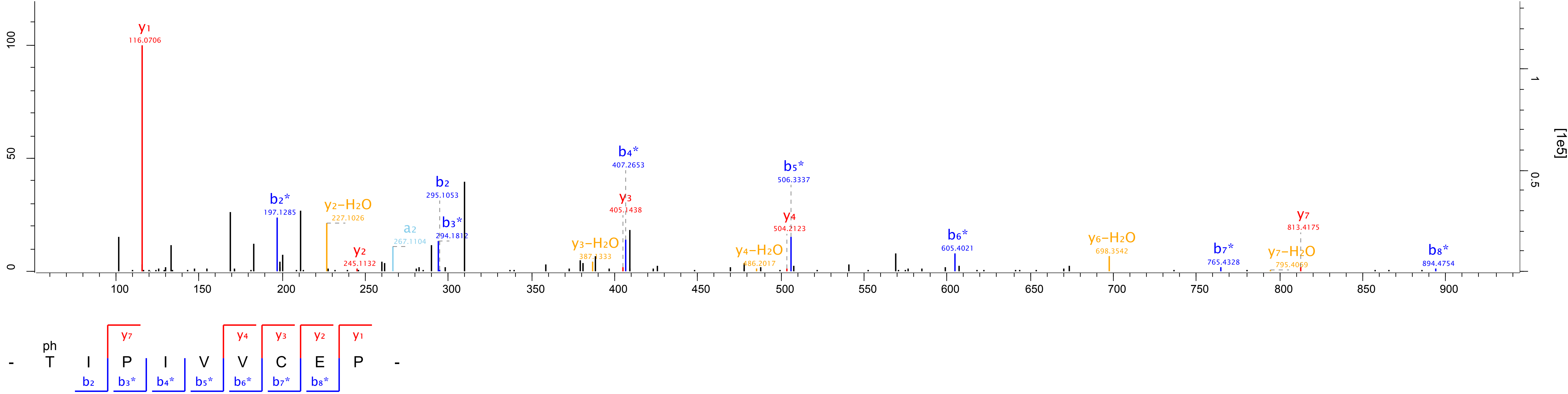

Raw file	Scan	Method	Score	m/z
20101013_Velos3_NaNa_COLLAB_salvage_5527_02	5796	FTMS; HCD	203.26	784.85

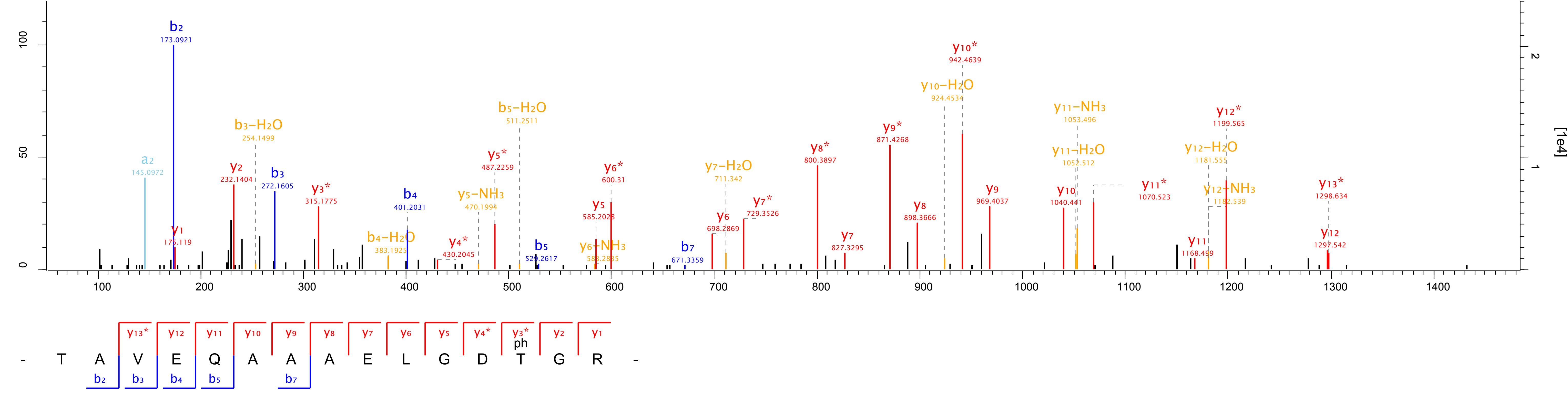

Raw file	Scan	Method	Score	m/z
20101013_Velos3_NaNa_COLLAB_salvage_5527_02	1984	FTMS; HCD	77.66	406.7

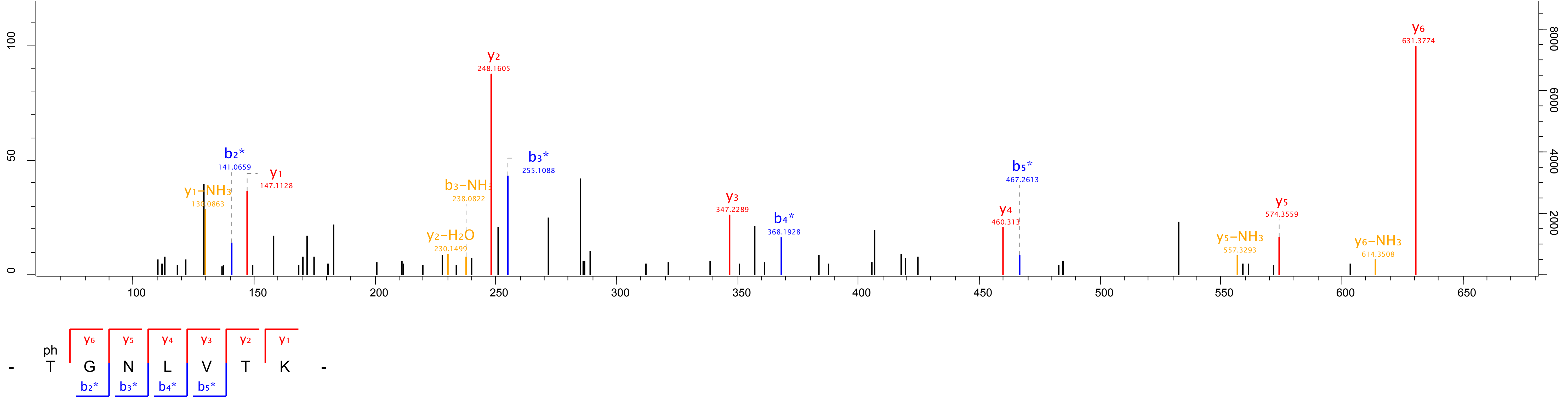

Raw file	Scan	Method	Score	m/z
20101013_Velos3_NaNa_COLLAB_5527_rep_03_flowthru_02	6292	FTMS; HCD	144.56	997.94

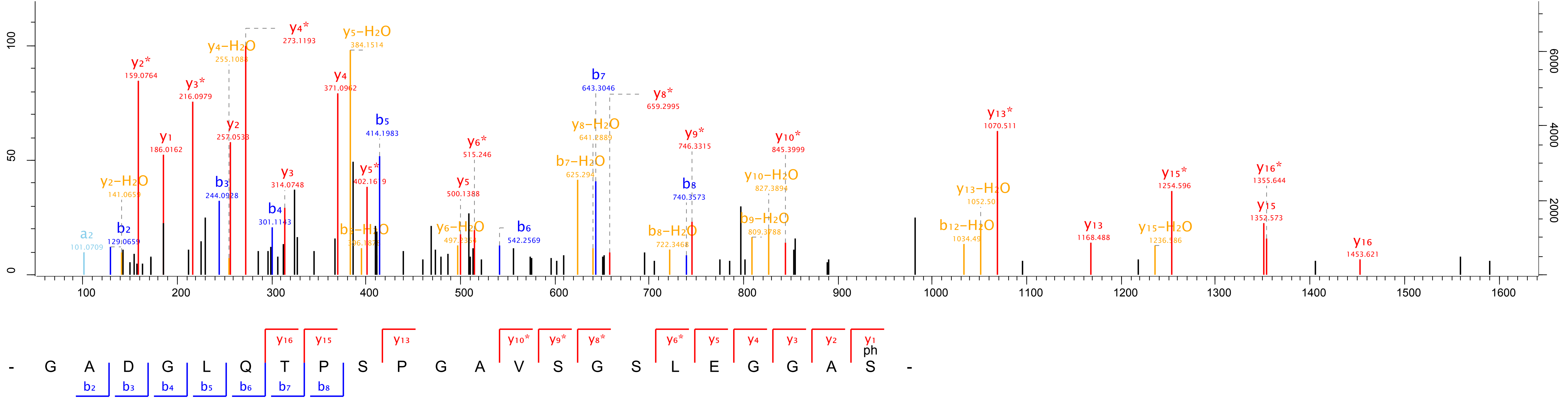

Raw file	Scan	Method	Score	m/z
20101013_Velos3_NaNa_COLLAB_5527_rep_02_fraction01	7670	FTMS; HCD	225.81	786.05

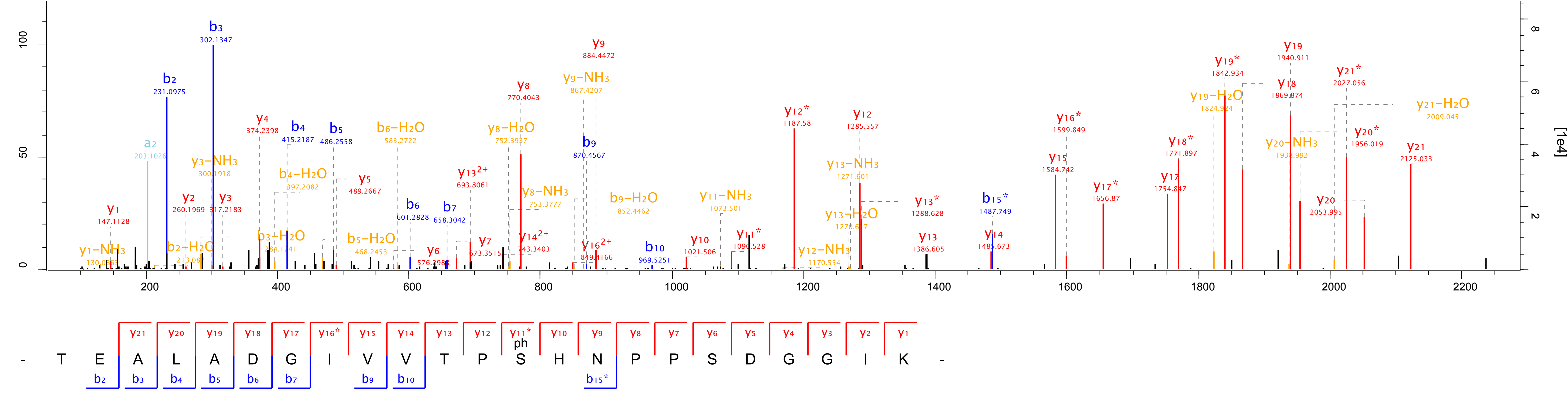

Raw file	Scan	Method	Score	m/z
20101013_Velos3_NaNa_COLLAB_5527_rep_01_fraction05	3936	FTMS; HCD	83.86	645.54

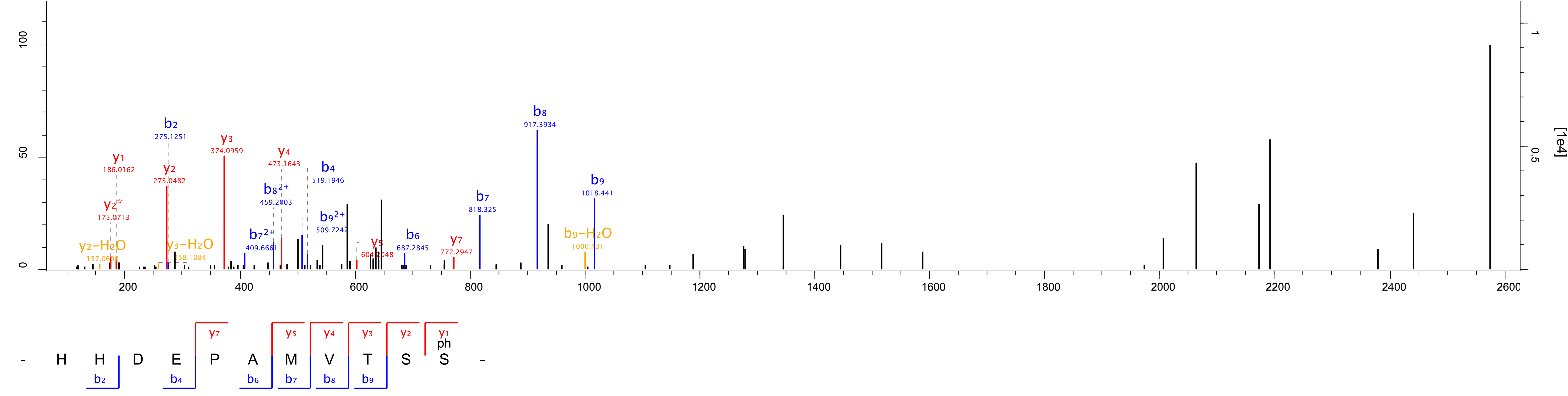

Raw file	Scan	Method	Score	m/z
20101013_Velos3_NaNa_COLLAB_5527_rep_03_flowthru_03	7131	FTMS; HCD	50.83	762.37

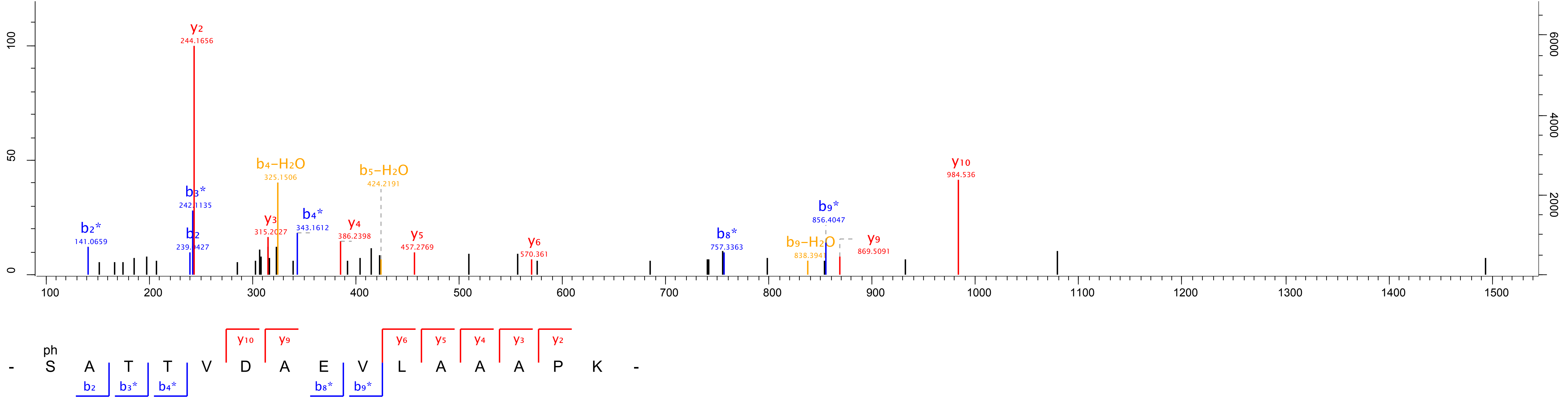

Supplement: Supplementary file 3 [file Image1.PDF]
