# Supplementary material for: Phosphoproteomics analysis of a clinical Mycobacterium tuberculosis Beijing isolate: expanding the mycobacterial phosphoproteome catalog
Source: Front Microbiol. 2015 Feb 10;6:6. doi: 10.3389/fmicb.2015.00006 (PMC4322841; doi:10.3389/fmicb.2015.00006)

Raw file	Scan	Method	Score	m/z
20101013_Velos3_NaNa_COLLAB_5527_rep_02_flowthru_01	9386	FTMS; HCD	60.16	605.31

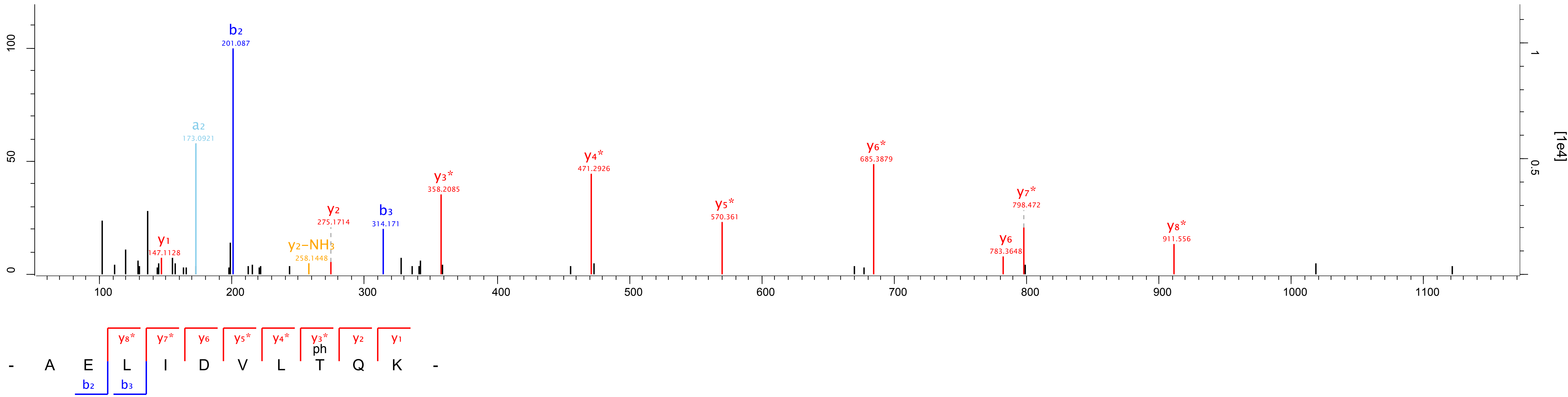

Raw file	Scan	Method	Score	m/z
20101013_Velos3_NaNa_COLLAB_5527_rep_03_flowthru_01	11959	FTMS; HCD	85.74	886.87

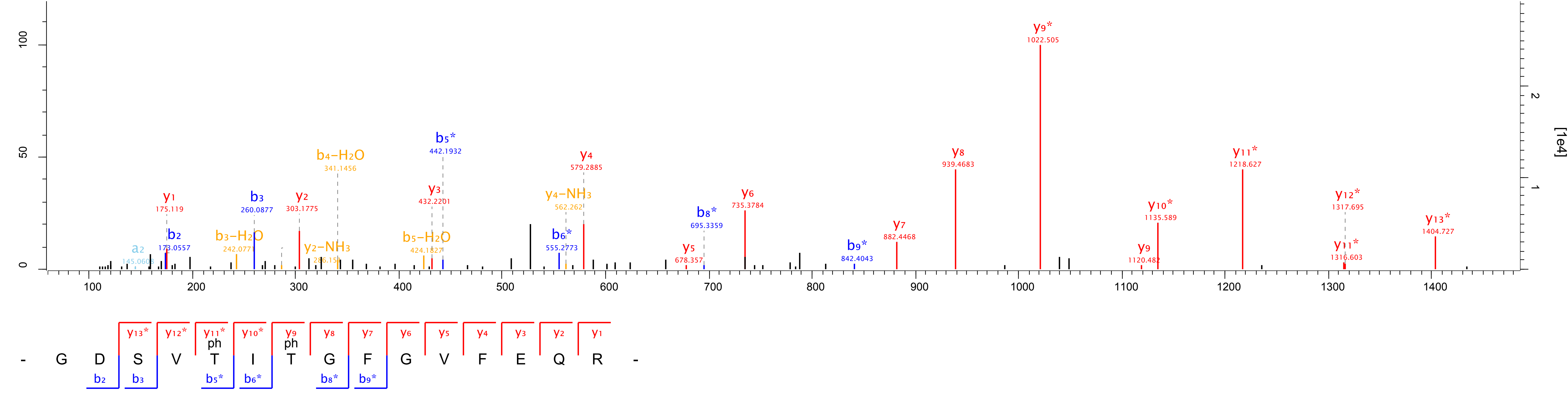

Raw file	Scan	Method	Score	m/z
20101013_Velos3_NaNa_COLLAB_5527_rep_03_flowthru_01	11959	FTMS; HCD	85.74	886.87

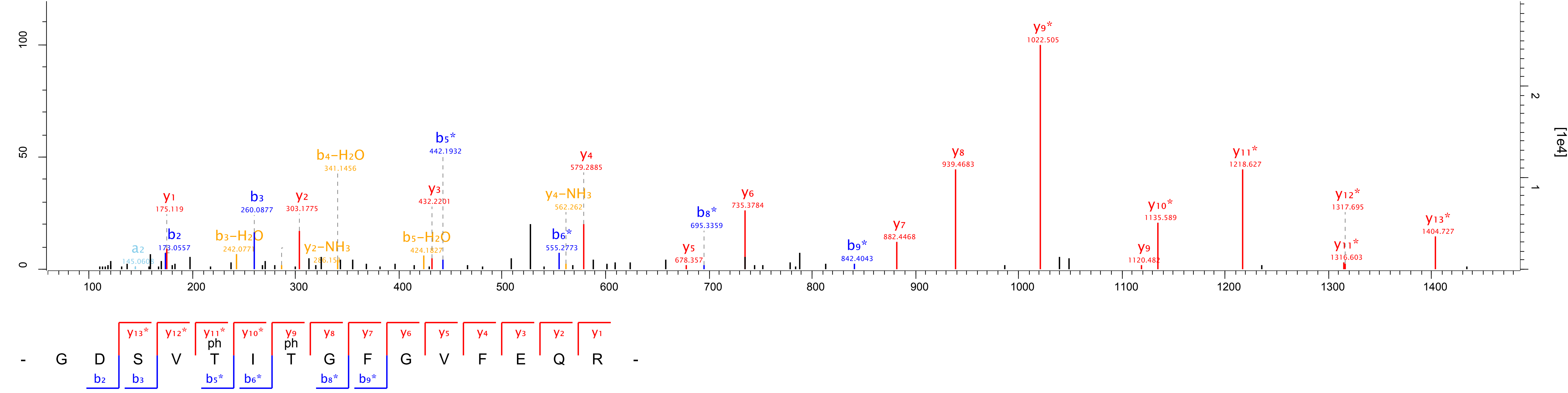

Raw file	Scan	Method	Score	m/z
20101013_Velos3_NaNa_COLLAB_5527_rep_03_fraction02	7444	FTMS; HCD	94.09	651.29

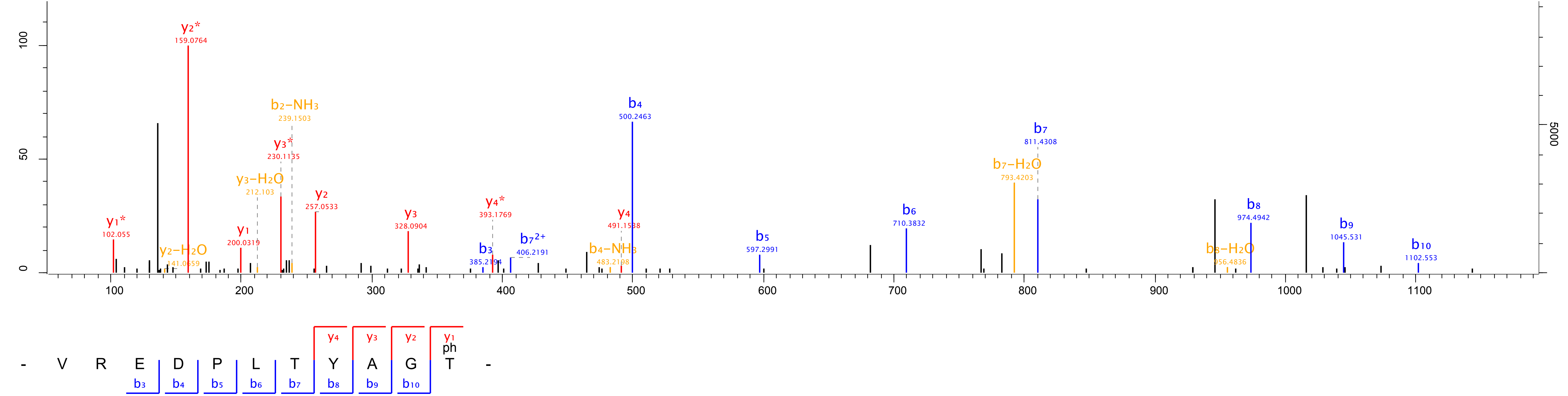

Raw file	Scan	Method	Score	m/z
20101013_Velos3_NaNa_COLLAB_salvage_5527_02	4642	FTMS; HCD	120.57	593.27

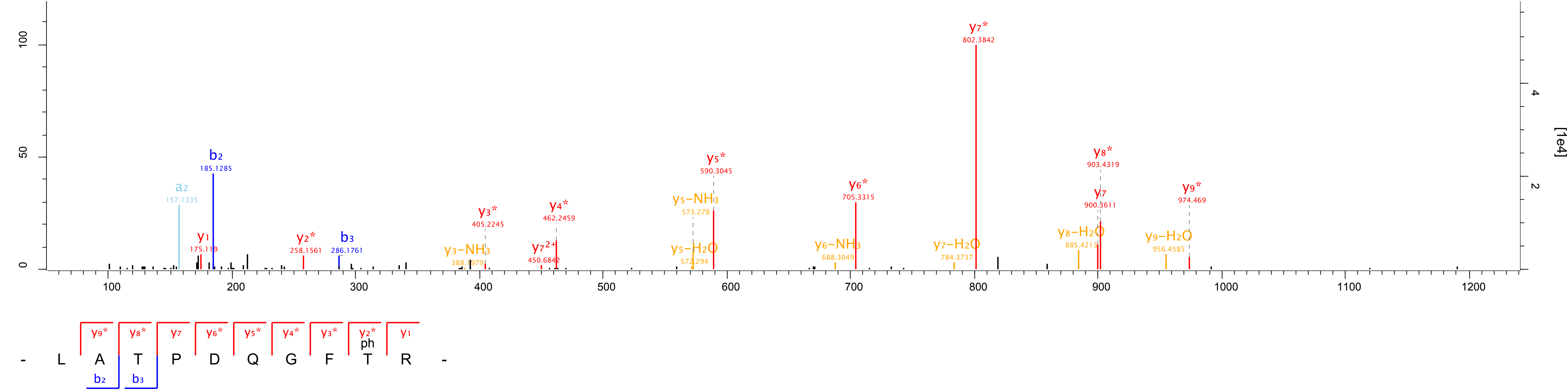

Raw file	Scan	Method	Score	m/z
20101013_Velos3_NaNa_COLLAB_5527_rep_03_flowthru_01	3523	FTMS; HCD	73.28	669.78

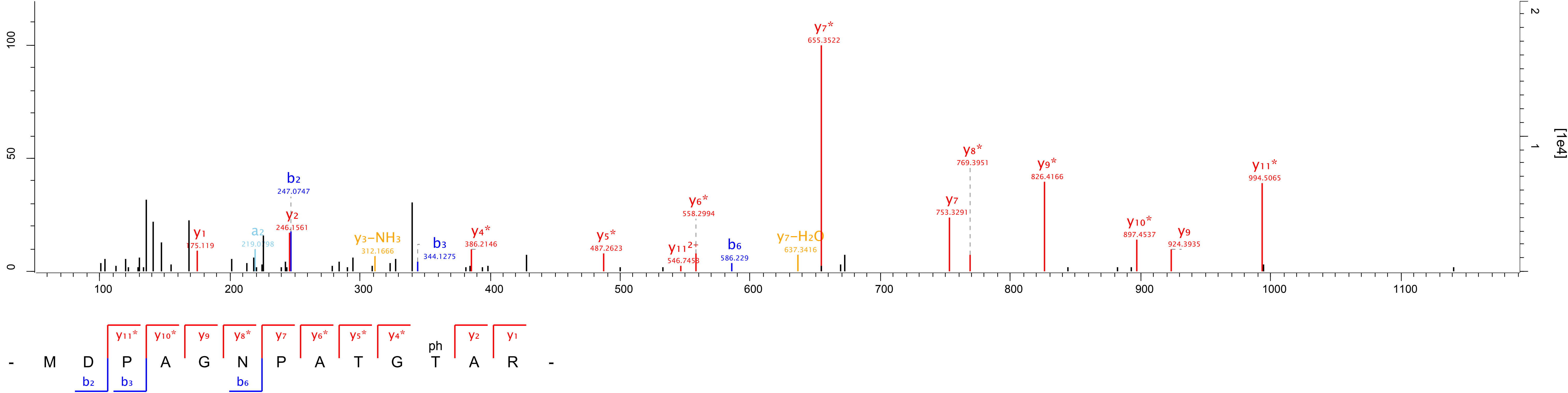

Raw file	Scan	Method	Score	m/z
20101013_Velos3_NaNa_COLLAB_5527_rep_01_flowthru_03	6936	FTMS; HCD	52.81	868.38

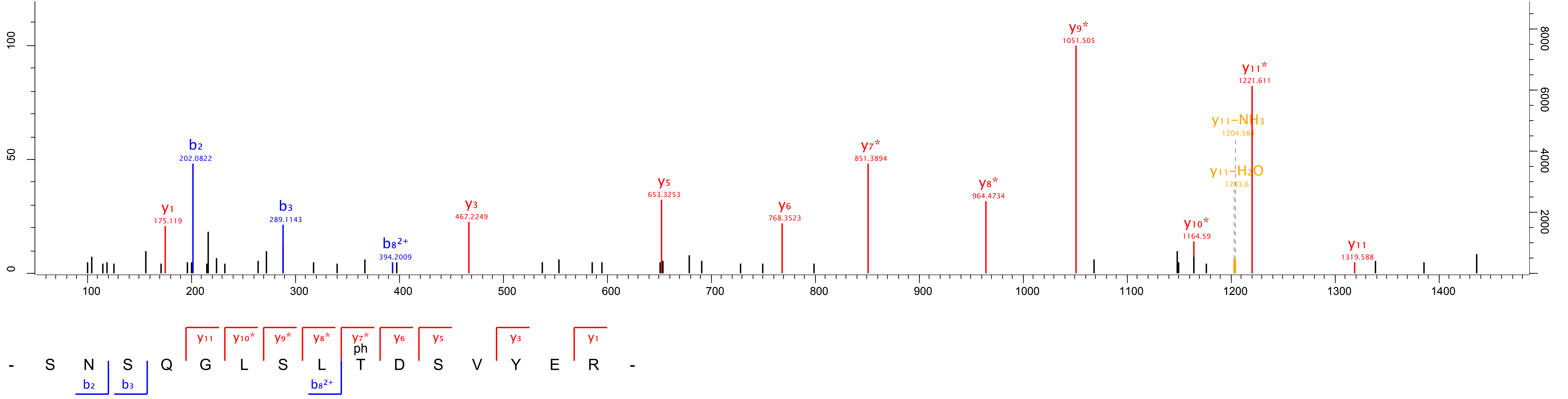

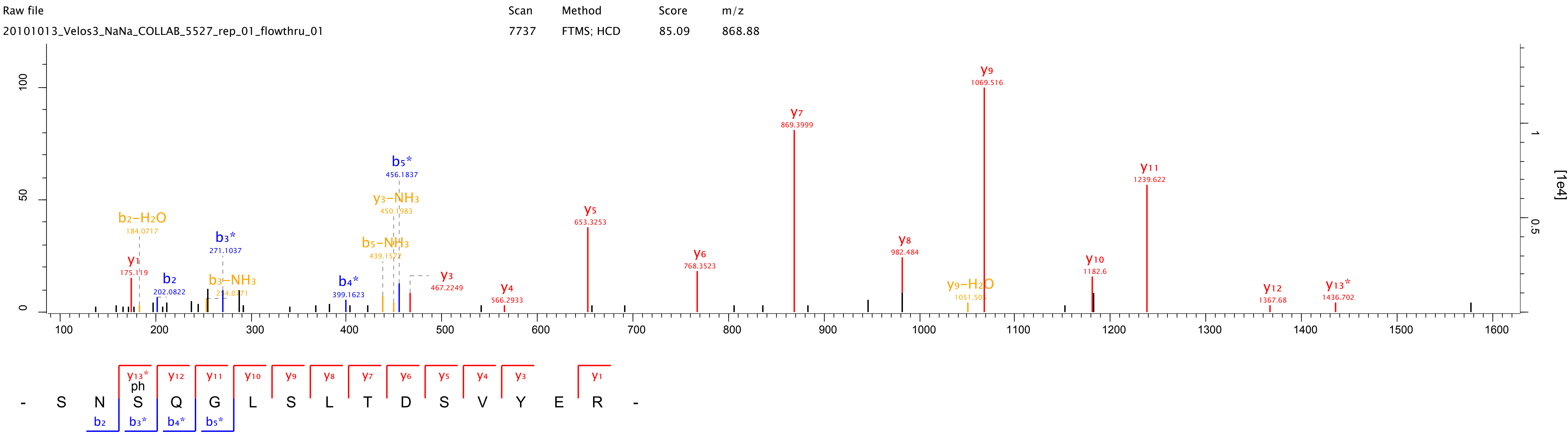
Raw file
20101013_Velos3_NaNa_COLLAB_5527_rep_01_flowthru_01
Scan
7737
Method
FTMS; HCD
Score
85.09
m/z
868.88
[1e4]
y9 1069.516
y7 869.3999
y11 1239.622
b5* 456.1837
y3−NH3 450.1983
b2−H2O 184.0717
b3* 271.1037
b5−NH3 439.1572
y5 653.3253
y8 982.484
y6 768.3523
y1 175.119
y10 1182.6
b2 202.0822
y3 467.2249
b4* 399.1623
b3−NH3 254.0771
y9−H2O 1051.505
y4 566.2933
y12 1367.68
y13* 1436.702
- S N S Q G L S L T D S V Y E R -
ph
y13* y12 y11 y10 y9 y8 y7 y6 y5 y4 y3 y1
b2 b3* b4* b5*

Raw file	Scan	Method	Score	m/z
20101013_Velos3_NaNa_COLLAB_5527_rep_01_flowthru_01	3020	FTMS; HCD	75.76	593.26

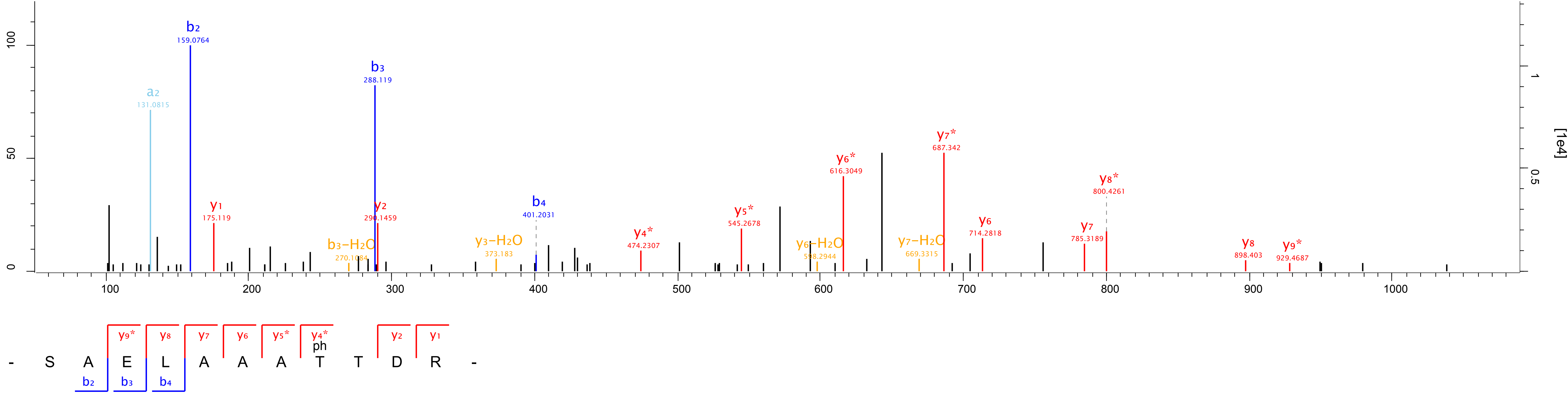

Raw file	Scan	Method	Score	m/z
20101013_Velos3_NaNa_COLLAB_5527_rep_03_fraction02	12245	FTMS; HCD	47.92	553.26

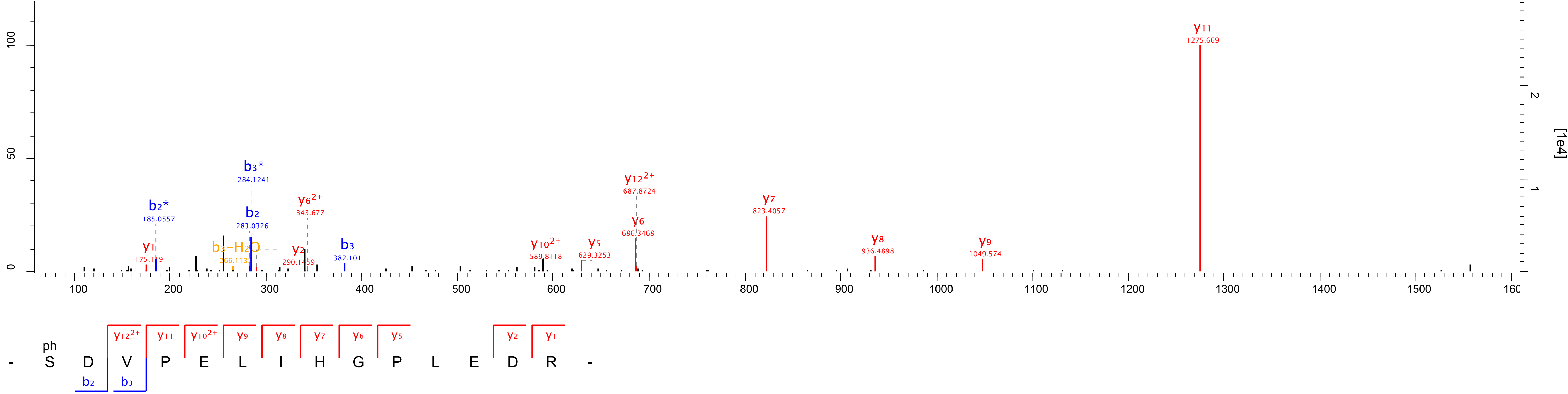

Raw file	Scan	Method	Score	m/z
20101013_Velos3_NaNa_COLLAB_5527_rep_02_flowthru_01	4504	FTMS; HCD	78.67	755.81

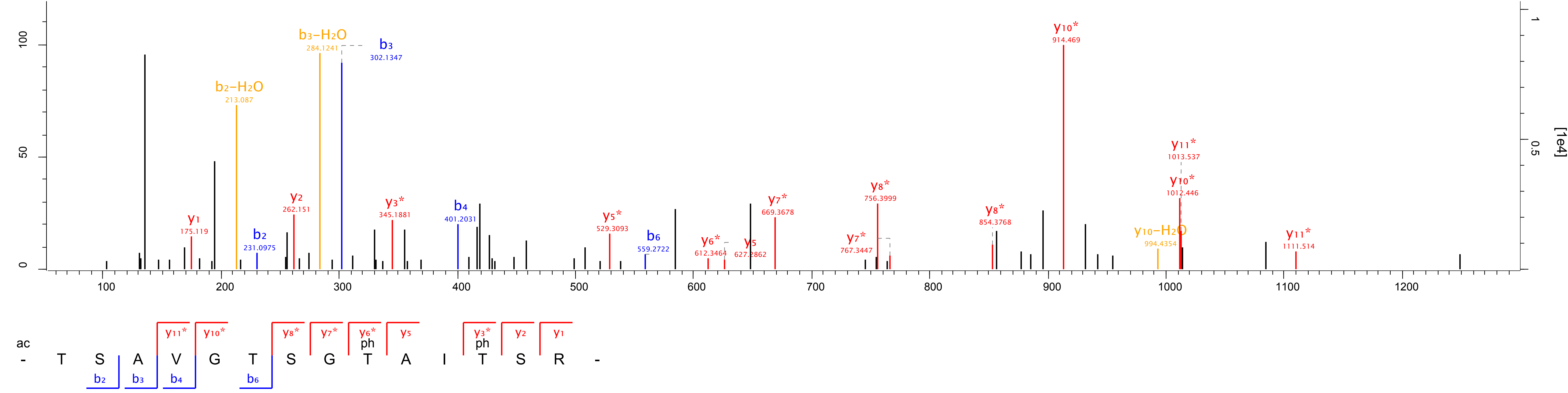

Raw file	Scan	Method	Score	m/z
20101013_Velos3_NaNa_COLLAB_salvage_5527_02	7626	FTMS; HCD	83.18	555.76

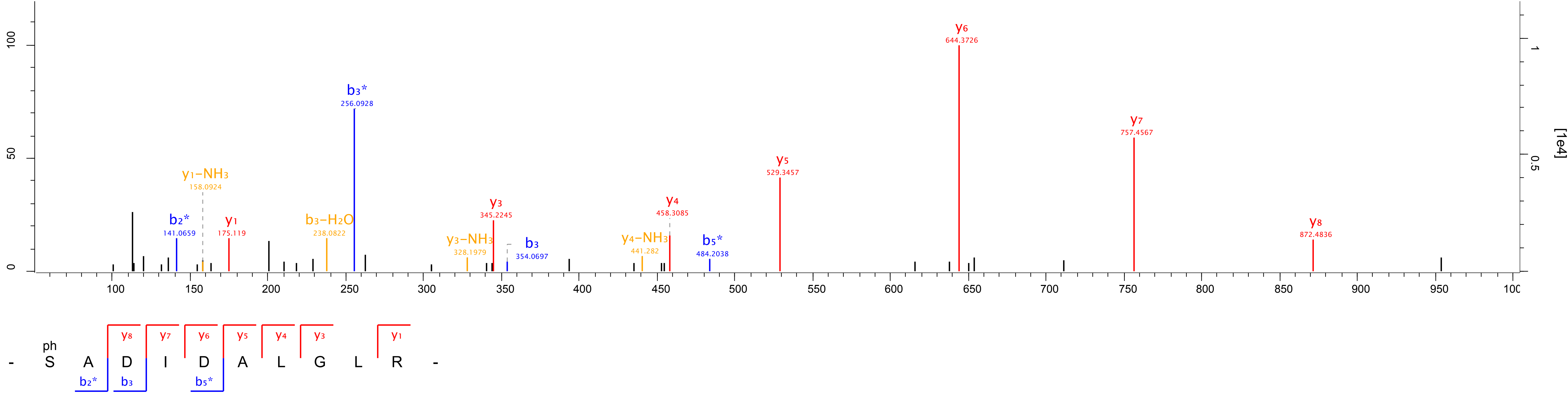

Raw file	Scan	Method	Score	m/z
20101013_Velos3_NaNa_COLLAB_5527_rep_03_fraction01	7921	FTMS; HCD	64.1	601.27

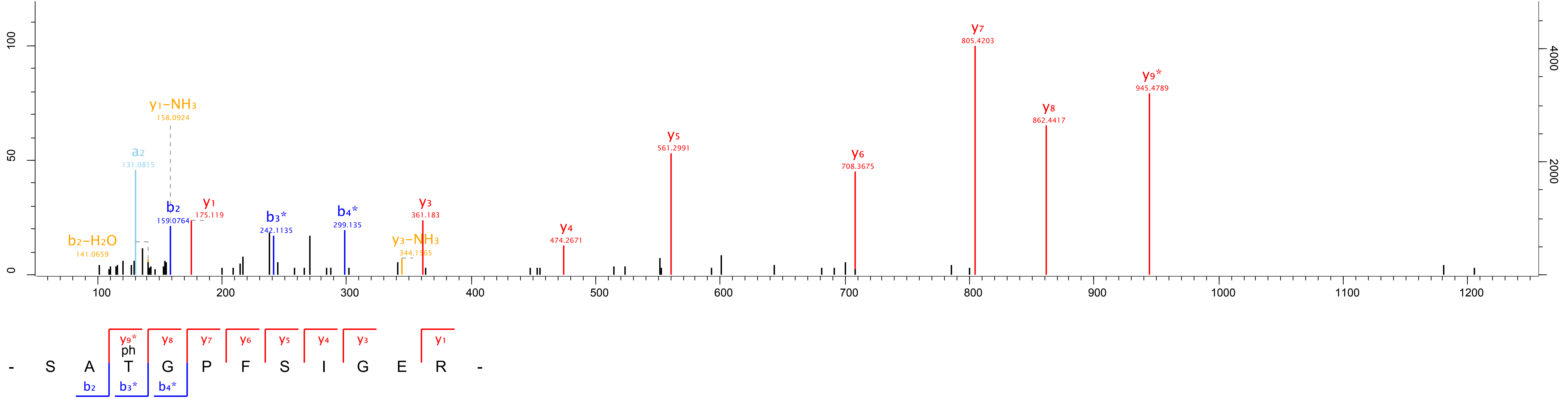

Raw file	Scan	Method	Score	m/z
20101013_Velos3_NaNa_COLLAB_5527_rep_03_flowthru_01	3723	FTMS; HCD	95.93	943.86

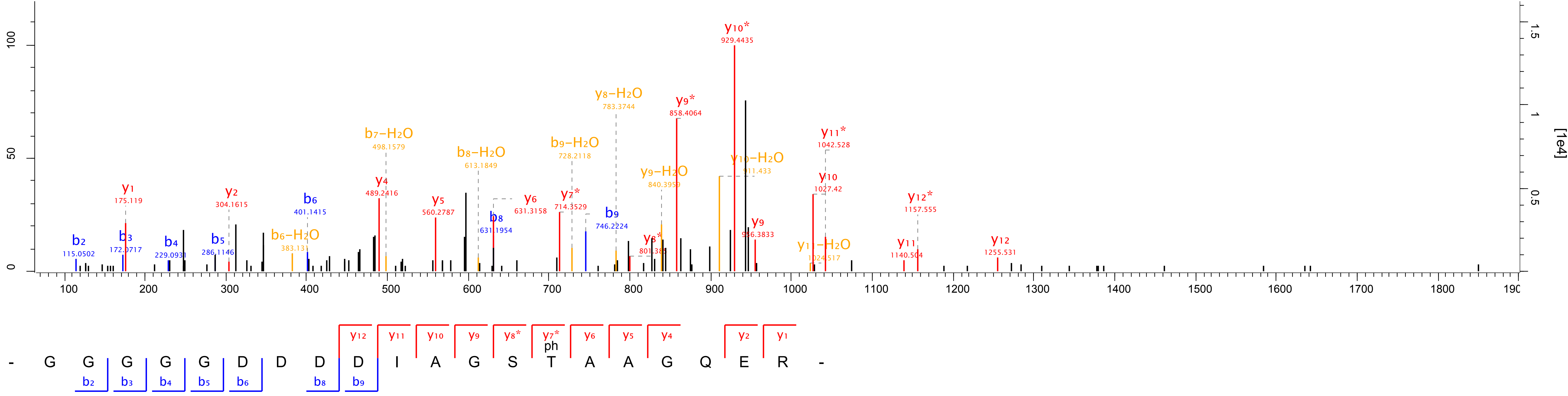

Raw file	Scan	Method	Score	m/z
20101013_Velos3_NaNa_COLLAB_5527_rep_02_flowthru_01	3506	FTMS; HCD	103.02	943.87

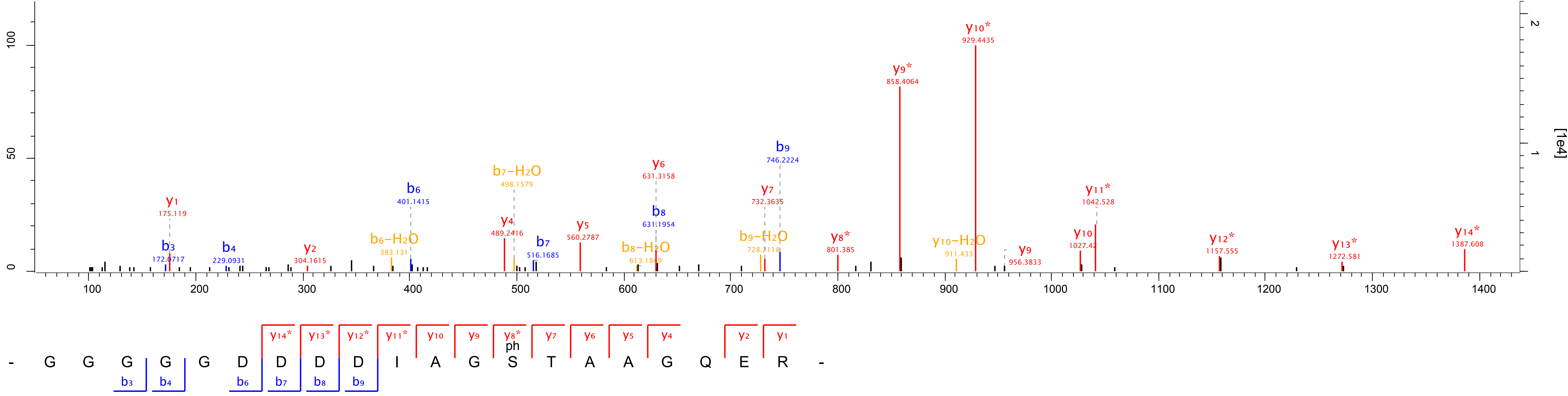

Raw file	Scan	Method	Score	m/z
20101013_Velos3_NaNa_COLLAB_5527_rep_03_flowthru_01	9312	FTMS; HCD	66.06	544.22

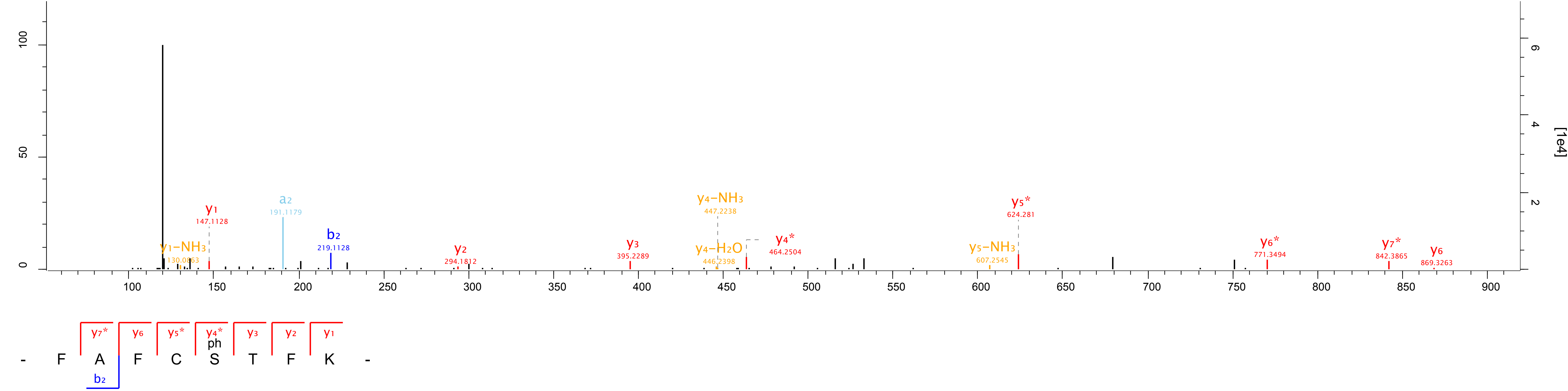

Raw file	Scan	Method	Score	m/z
20101013_Velos3_NaNa_COLLAB_5527_rep_03_fraction01	6973	FTMS; HCD	51.73	615.76

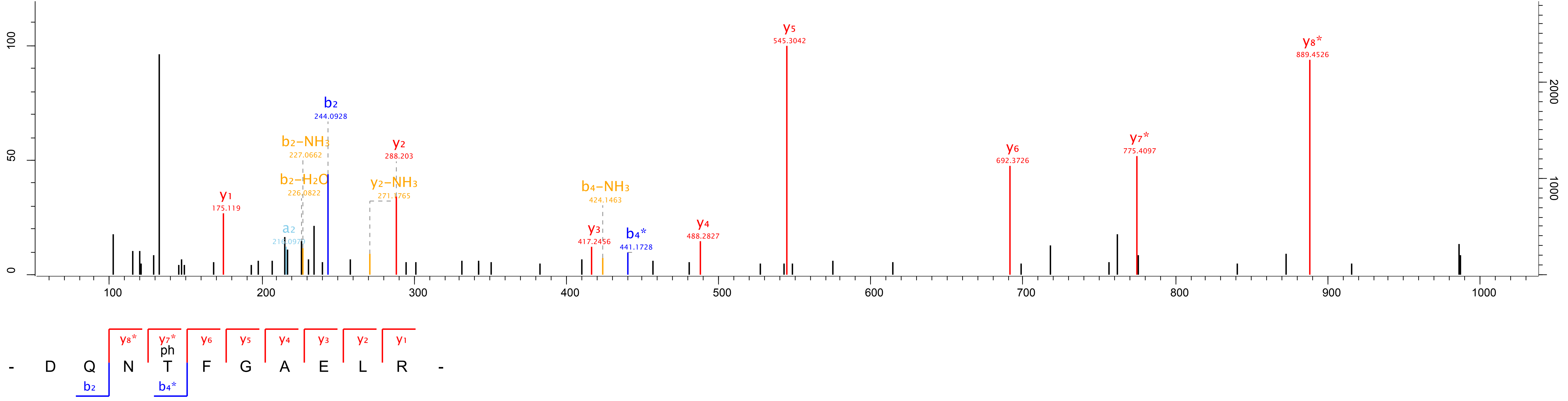

Raw file	Scan	Method	Score	m/z
20101013_Velos3_NaNa_COLLAB_salvage_5527_02	5243	FTMS; HCD	42.08	588.29

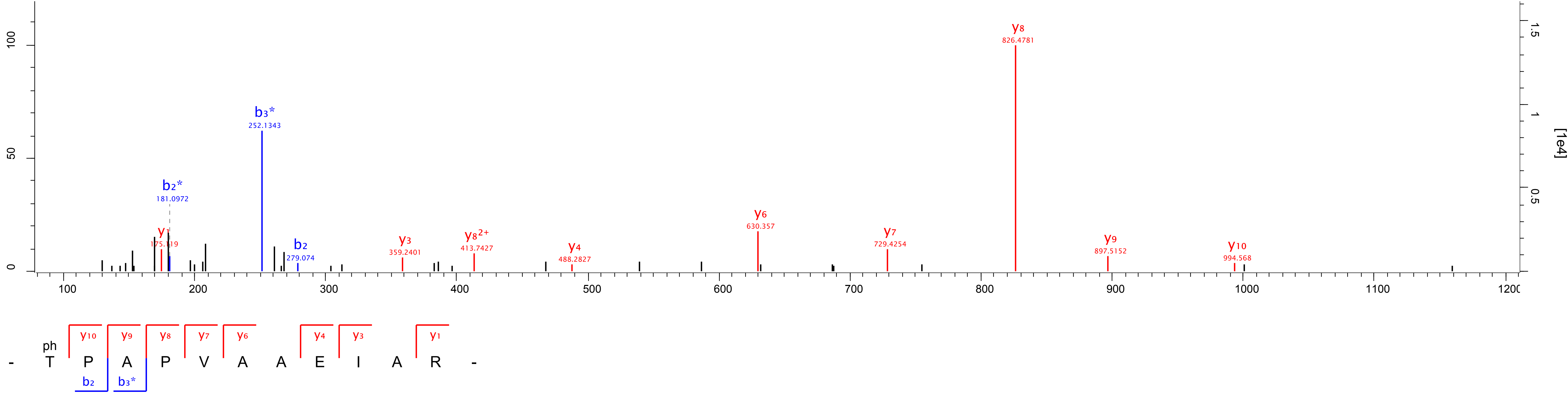

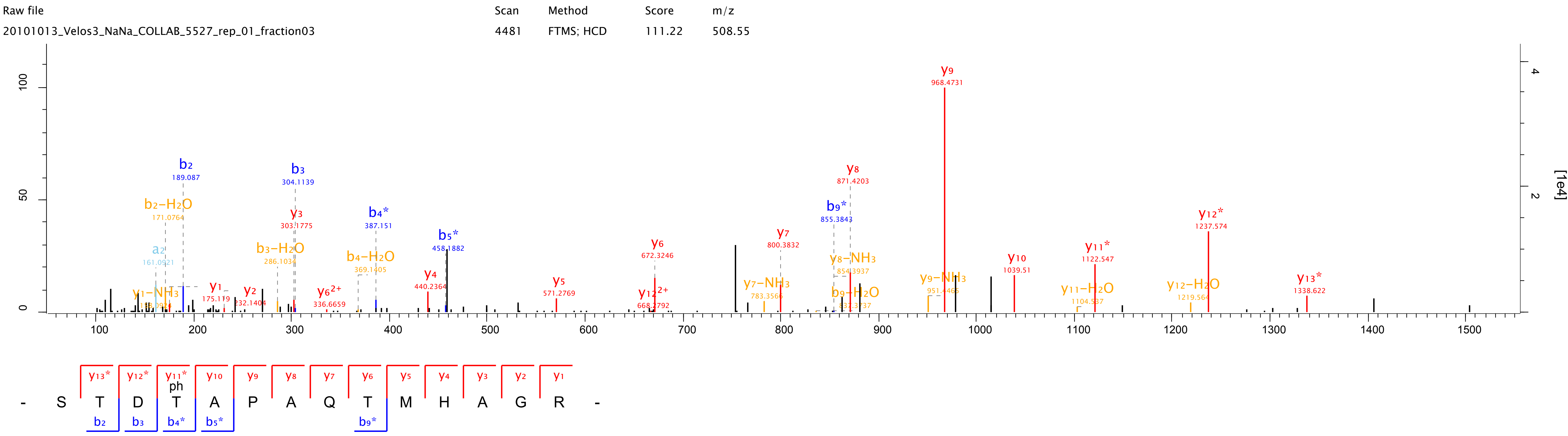
Raw file
20101013_Velos3_NaNa_COLLAB_5527_rep_01_fraction03
Scan
4481
Method
FTMS; HCD
Score
111.22
m/z
508.55
[1e4]
y9 968.4731
y8 871.4203
b9* 855.3843
y12* 1237.574
b2 189.087
b3 304.1139
b2-H2O 171.0764
y3 303.1775
b4* 387.151
b5* 458.1882
a2 161.0921
b3-H2O 286.1034
b4-H2O 369.1405
y6 672.3246
y7 800.3832
y8-NH3 854.3937
y10 1039.51
y11* 1122.547
y4 440.2364
y5 571.2769
y7-NH3 783.3566
y9-NH3 951.4465
b9-H2O 837.3737
y11-H2O 1104.537
y12-H2O 1219.564
y13* 1338.622
y1 175.119
y2 232.1404
y6 2+ 336.6659
y12 2+ 668.2792
y1-NH3
- S T D T A P A Q T M H A G R -
ph
y13* y12* y11* y10 y9 y8 y7 y6 y5 y4 y3 y2 y1
b2 b3 b4* b5* b9*

Raw file	Scan	Method	Score	m/z
20101013_Velos3_NaNa_COLLAB_5527_rep_02_fraction03	7474	FTMS; HCD	47.97	450.25

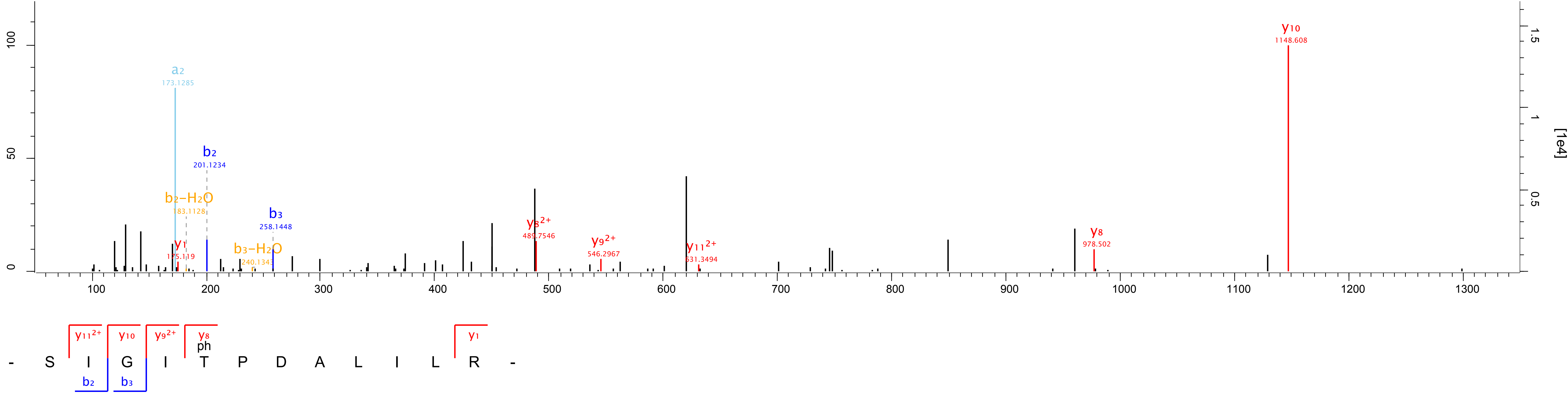

Raw file	Scan	Method	Score	m/z
20101013_Velos3_NaNa_COLLAB_5527_rep_01_flowthru_02	4179	FTMS; HCD	119.39	551.23

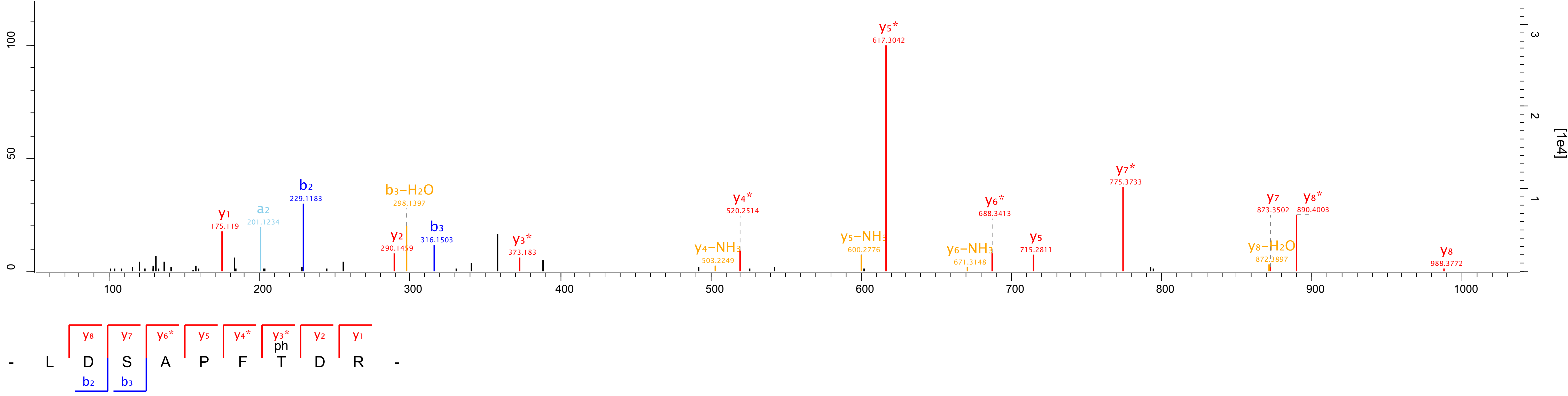

Raw file	Scan	Method	Score	m/z
20101013_Velos3_NaNa_COLLAB_5527_rep_01_flowthru_02	9208	FTMS; HCD	109.83	743.33

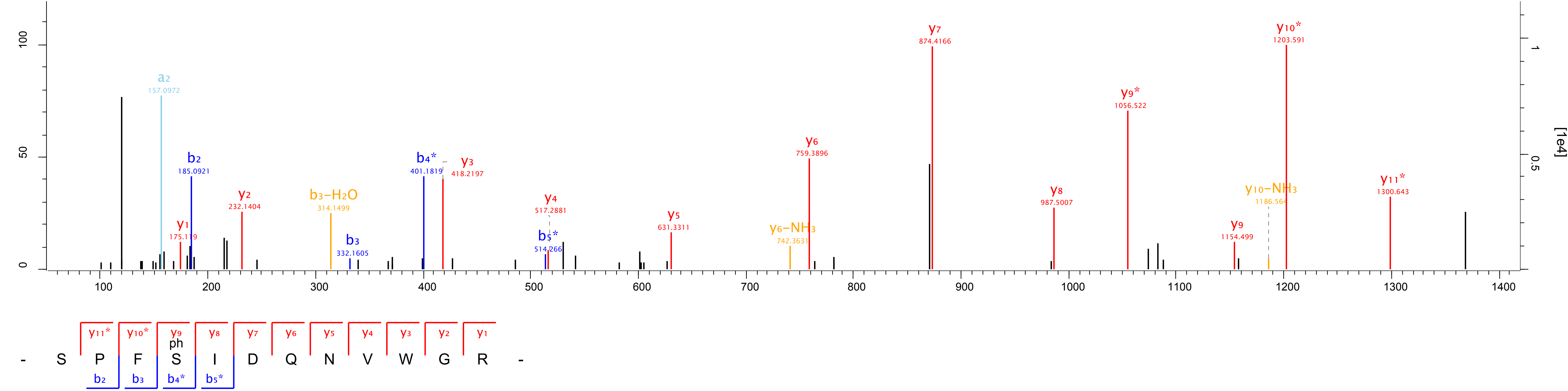

Raw file	Scan	Method	Score	m/z
20101013_Velos3_NaNa_COLLAB_5527_rep_03_fraction01	1933	FTMS; HCD	69.72	457.7

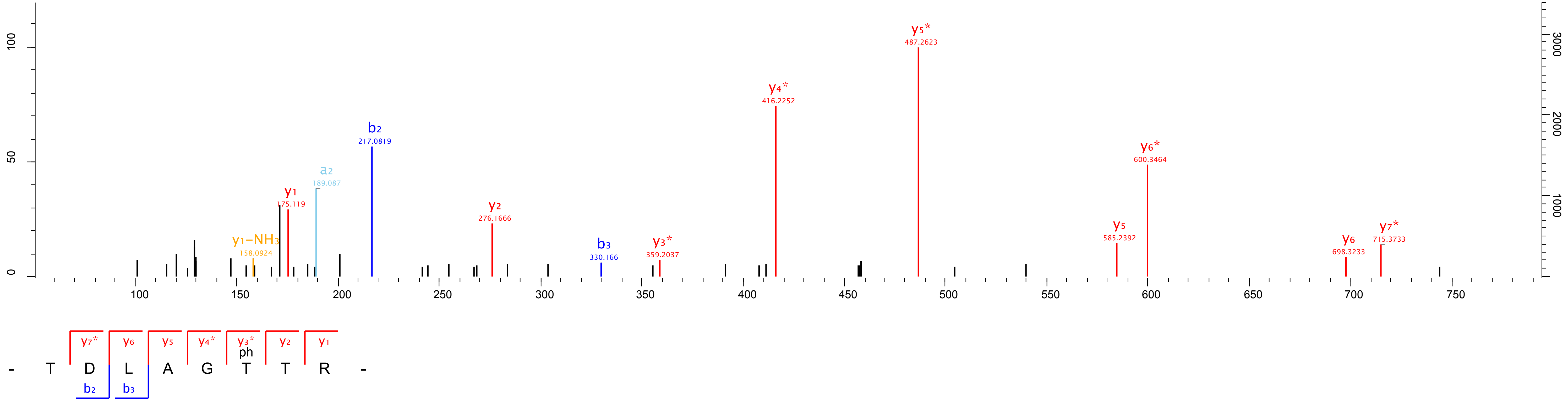

Raw file	Scan	Method	Score	m/z
20101013_Velos3_NaNa_COLLAB_5527_rep_03_flowthru_01	13170	FTMS; HCD	83.06	1010.95

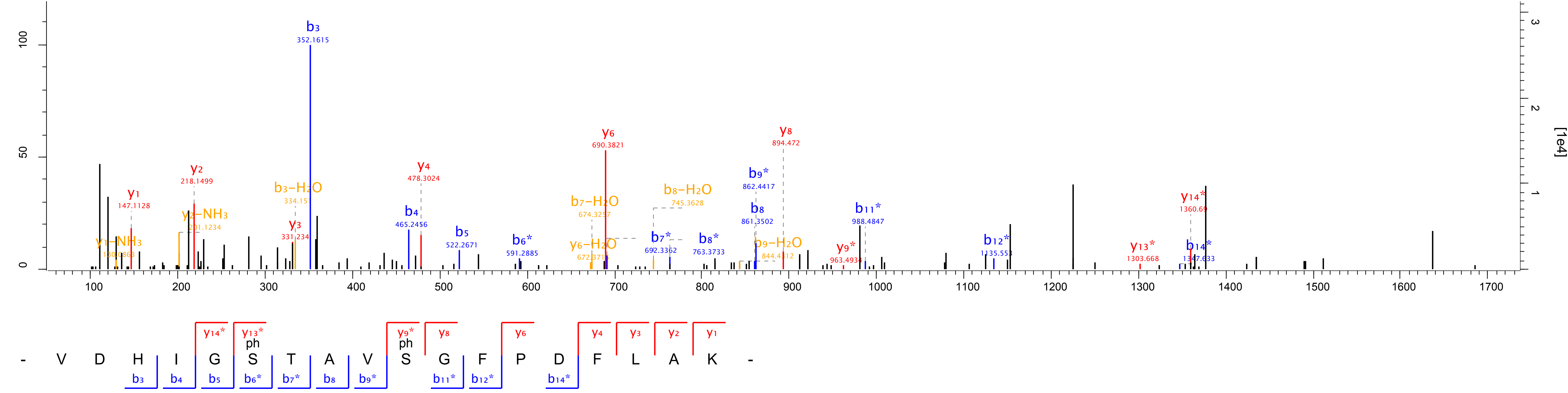

Raw file	Scan	Method	Score	m/z
20101013_Velos3_NaNa_COLLAB_salvage_5527_02	7007	FTMS; HCD	42.31	722.85

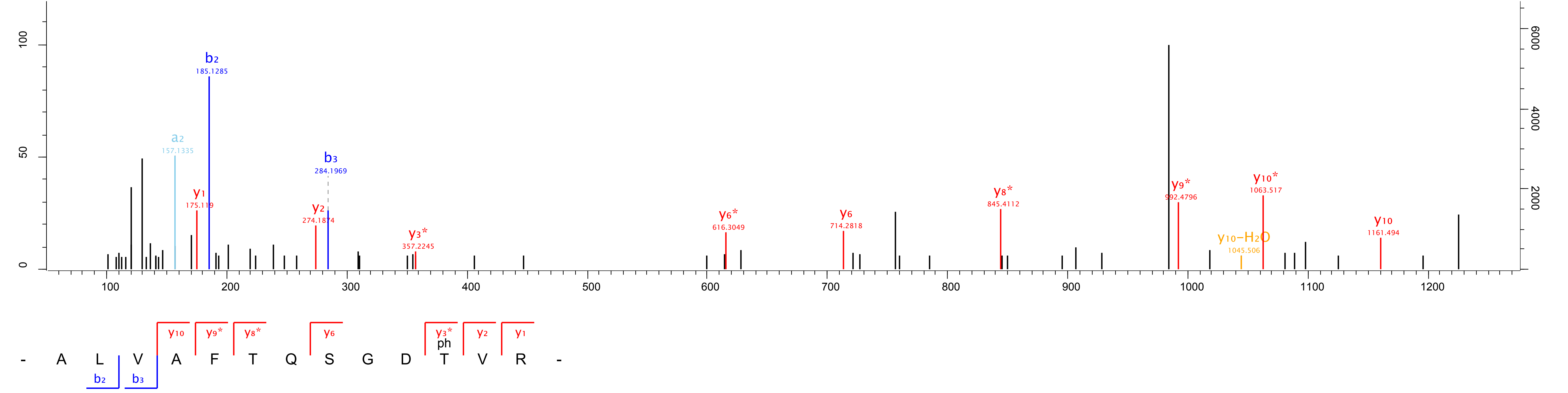

Raw file	Scan	Method	Score	m/z
20101013_Velos3_NaNa_COLLAB_5527_rep_01_flowthru_03	5860	FTMS; HCD	56.41	571.77

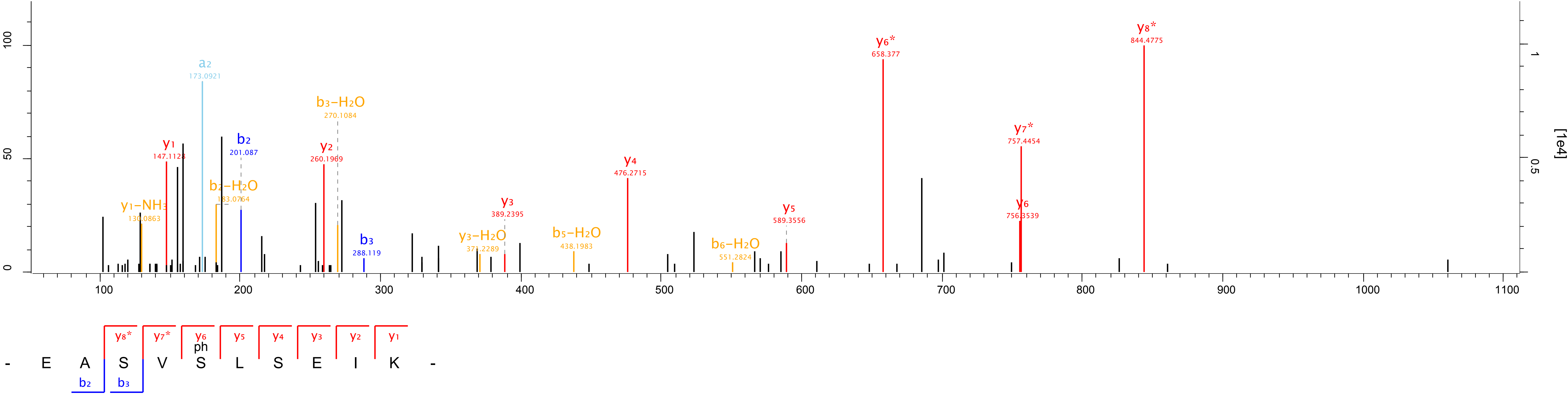

Raw file	Scan	Method	Score	m/z
20101013_Velos3_NaNa_COLLAB_salvage_5527_02	3584	FTMS; HCD	126.84	838.35

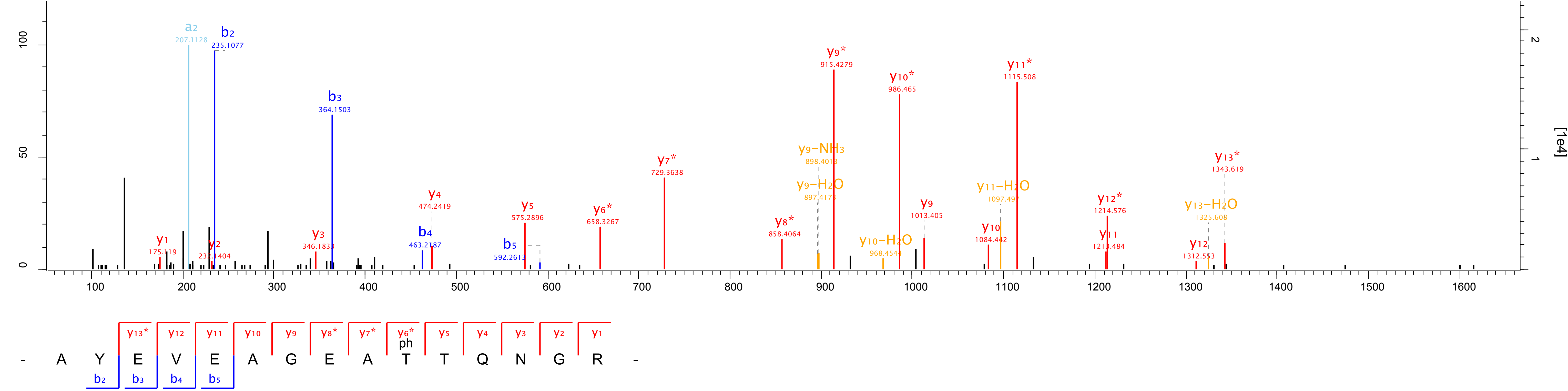

Raw file	Scan	Method	Score	m/z
20101013_Velos3_NaNa_COLLAB_5527_rep_03_fraction03	3903	FTMS; HCD	125.5	697.82

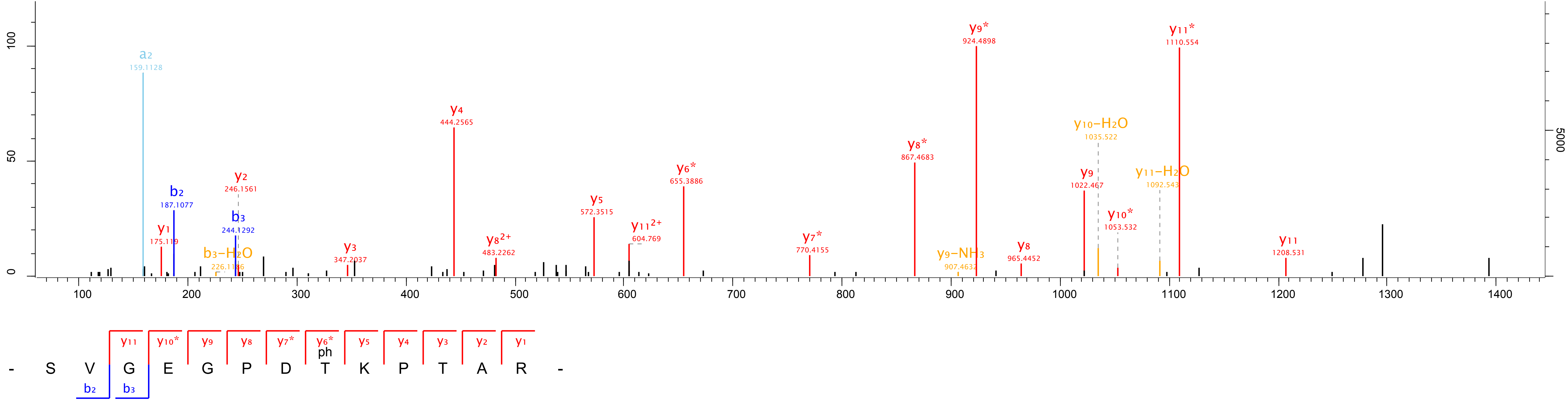

Raw file	Scan	Method	Score	m/z
20101013_Velos3_NaNa_COLLAB_salvage_5527_02	2740	FTMS; HCD	64.37	485.22

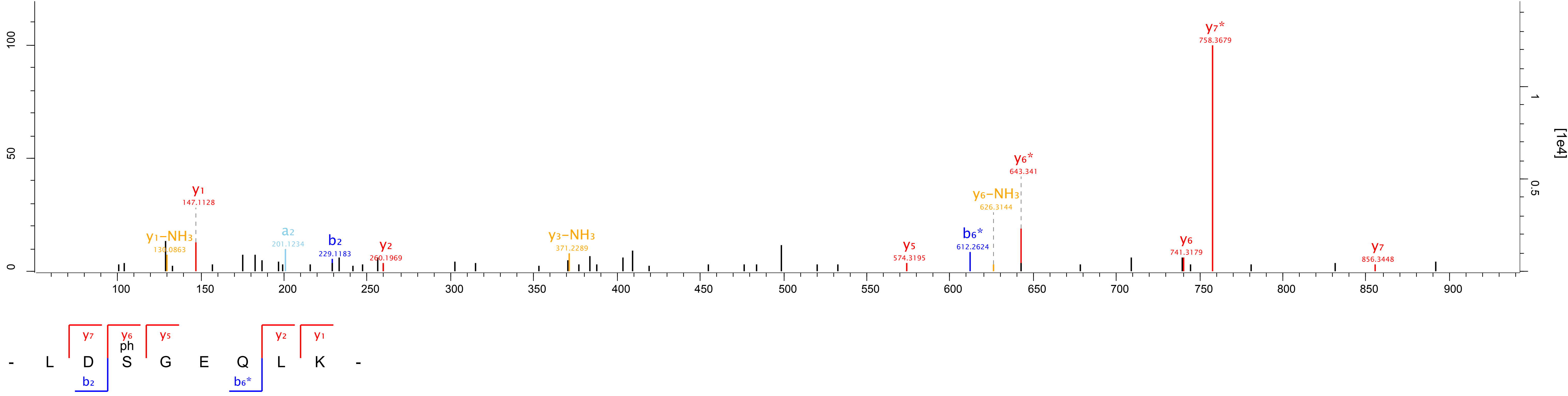

Raw file	Scan	Method	Score	m/z
20101013_Velos3_NaNa_COLLAB_5527_rep_03_flowthru_03	5762	FTMS; HCD	40.49	779.35

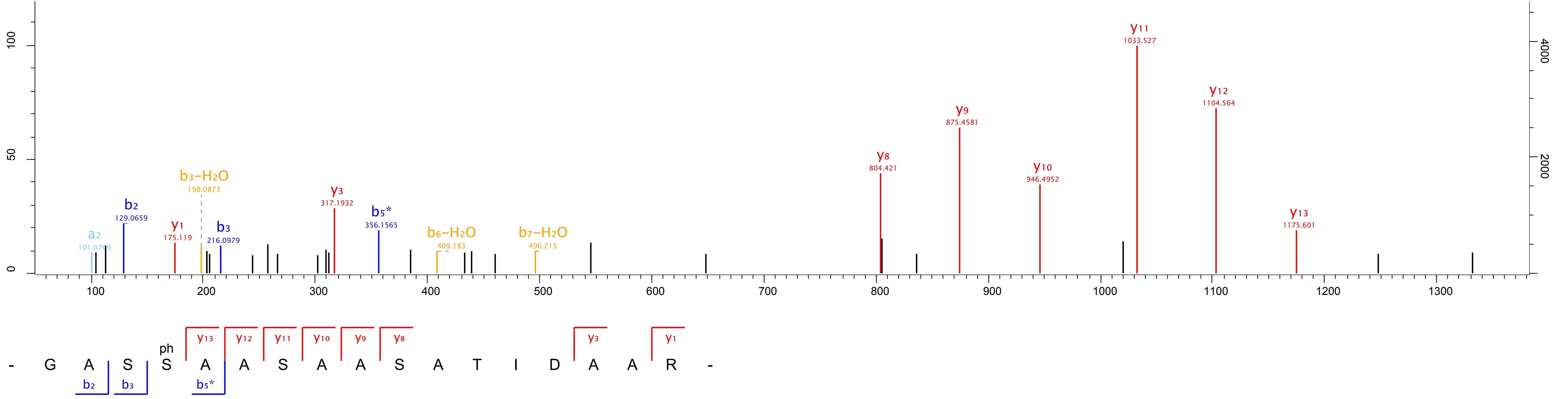

Raw file	Scan	Method	Score	m/z
20101013_Velos3_NaNa_COLLAB_5527_rep_03_fraction01	7078	FTMS; HCD	99.34	648.3

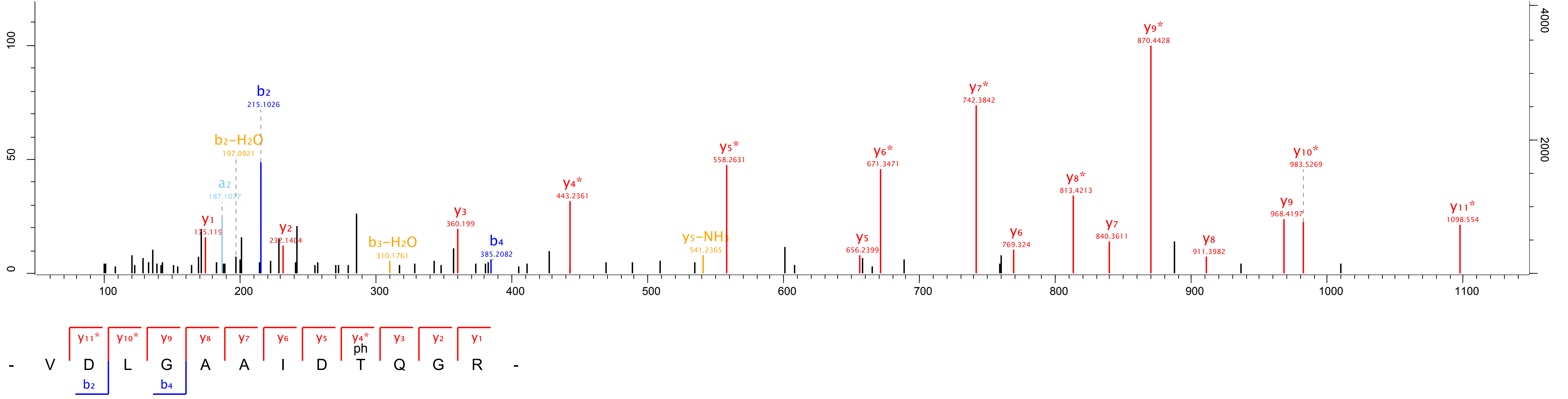

Raw file	Scan	Method	Score	m/z
20101013_Velos3_NaNa_COLLAB_5527_rep_01_flowthru_01	4772	FTMS; HCD	89.43	562.75

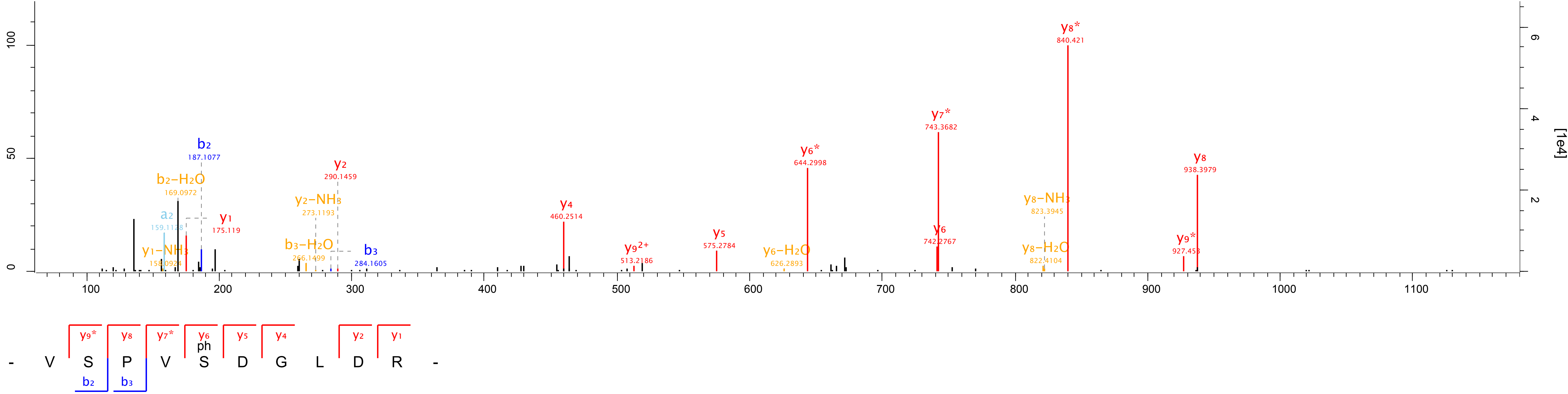

Raw file	Scan	Method	Score	m/z
20101013_Velos3_NaNa_COLLAB_salvage_5527_02	4731	FTMS; HCD	53.75	439.71

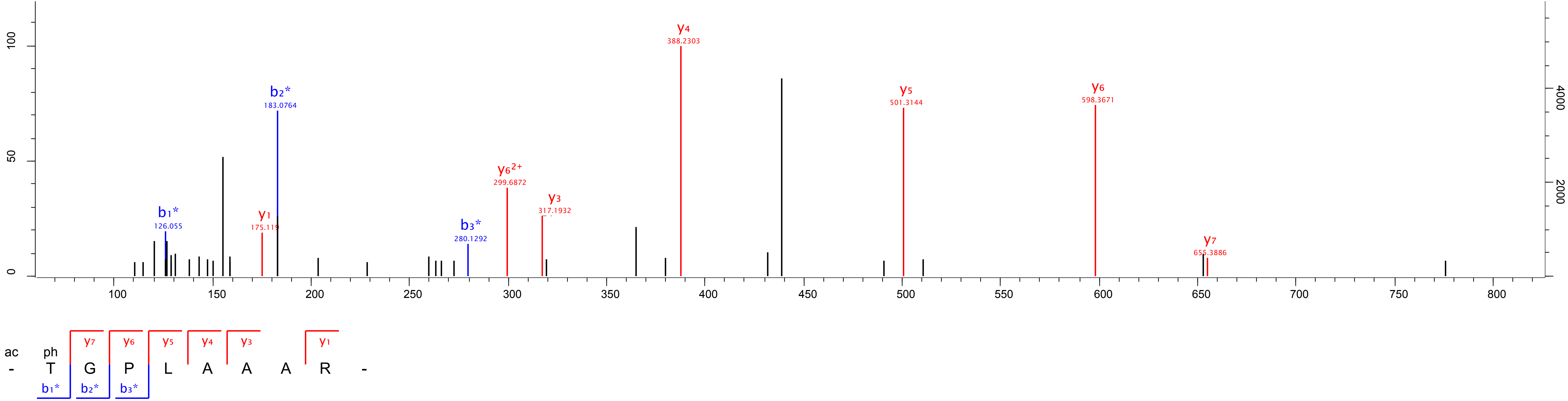

Raw file	Scan	Method	Score	m/z
20101013_Velos3_NaNa_COLLAB_5527_rep_03_fraction04	3702	FTMS; HCD	100.44	702.97

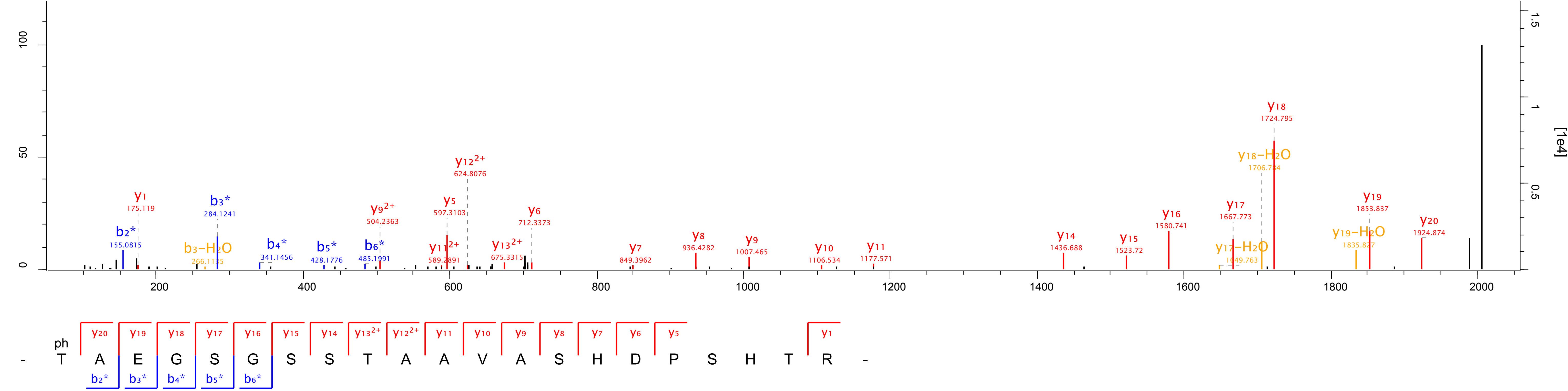

Raw file	Scan	Method	Score	m/z
20101013_Velos3_NaNa_COLLAB_5527_rep_03_fraction02	3750	FTMS; HCD	52.91	590.6

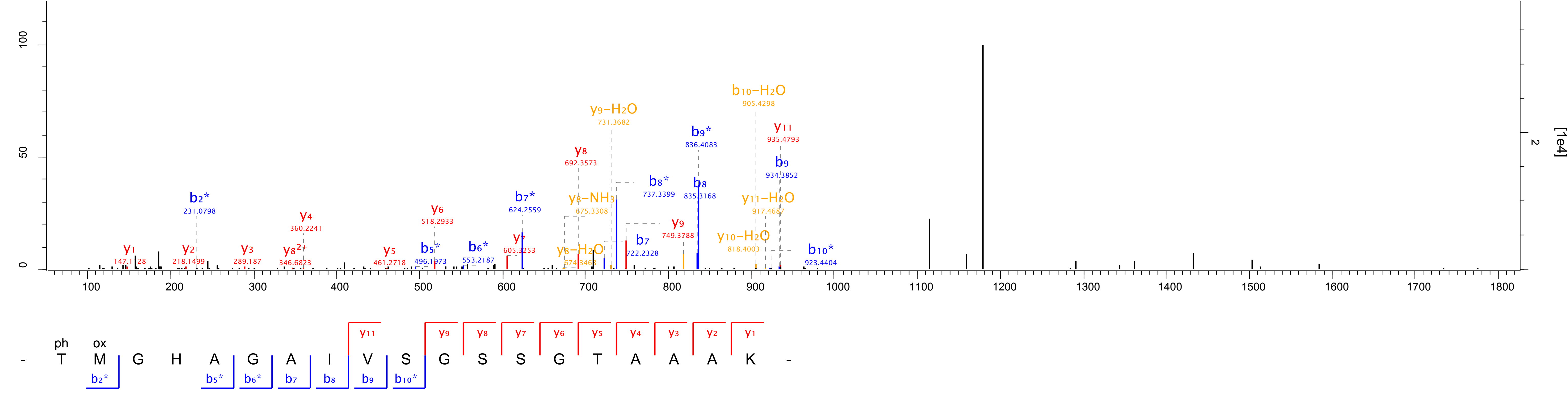

Raw file	Scan	Method	Score	m/z
20101013_Velos3_NaNa_COLLAB_5527_rep_03_flowthru_01	7451	FTMS; HCD	71.03	653.56

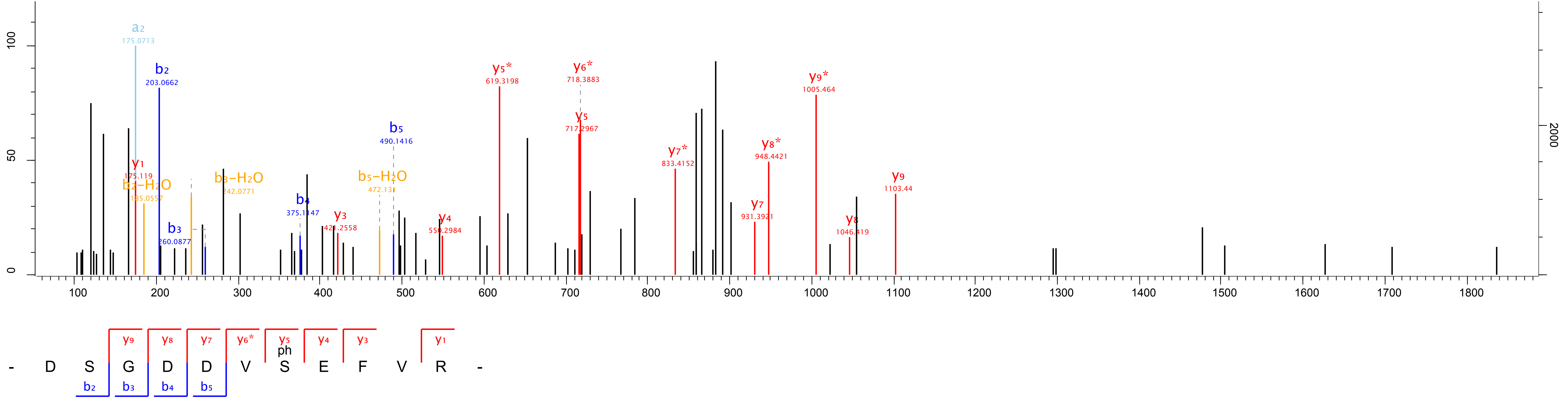

Raw file	Scan	Method	Score	m/z
20101013_Velos3_NaNa_COLLAB_5527_rep_01_flowthru_03	2228	FTMS; HCD	140.16	579.74

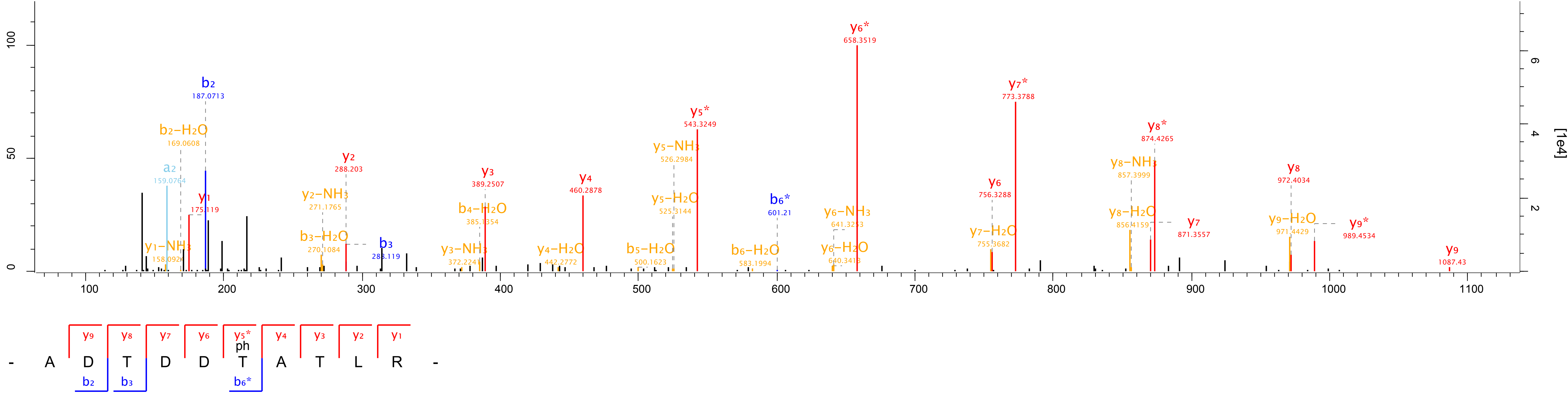

Raw file	Scan	Method	Score	m/z
20101013_Velos3_NaNa_COLLAB_salvage_5527_02	12366	FTMS; HCD	149.35	755.89

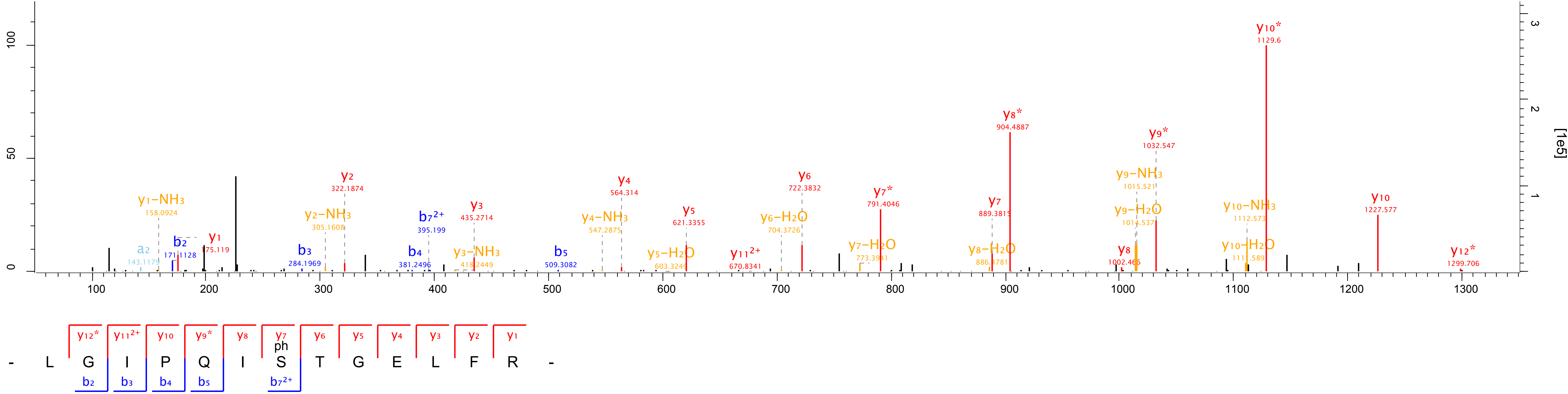

Raw file	Scan	Method	Score	m/z
20101013_Velos3_NaNa_COLLAB_5527_rep_01_flowthru_01	5348	FTMS; HCD	80.96	663.78

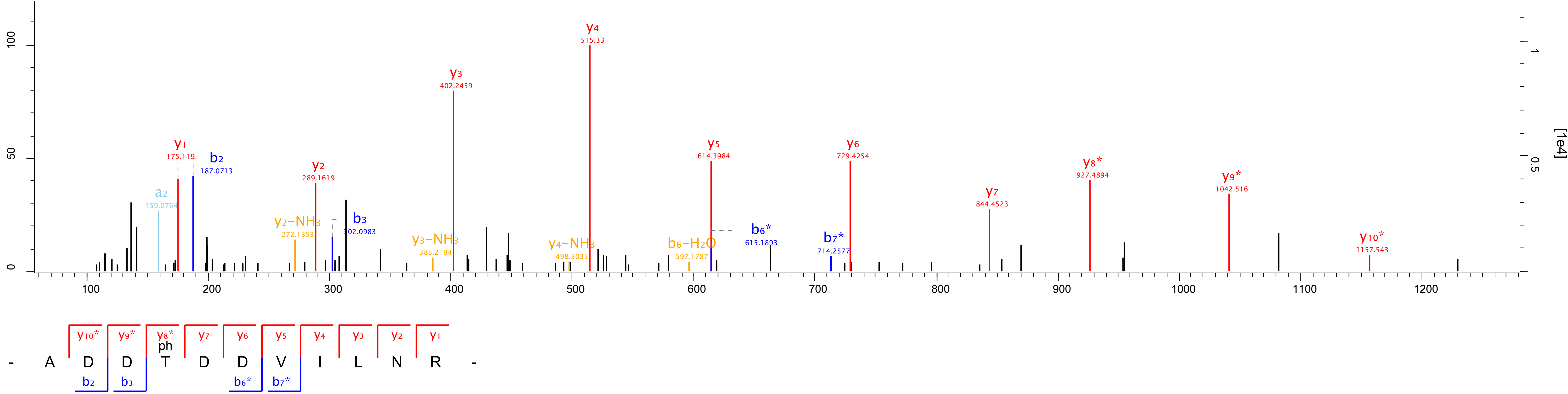

Raw file	Scan	Method	Score	m/z
20101013_Velos3_NaNa_COLLAB_salvage_5527_02	2375	FTMS; HCD	52.86	679.81

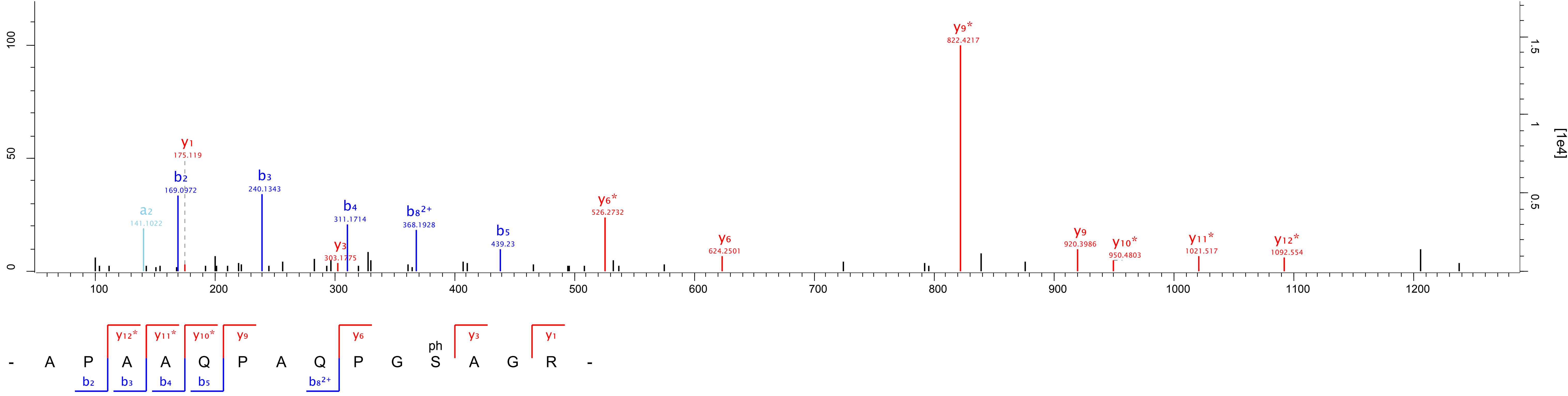

Raw file	Scan	Method	Score	m/z
20101013_Velos3_NaNa_COLLAB_5527_rep_01_flowthru_02	9250	FTMS; HCD	48.62	549.94

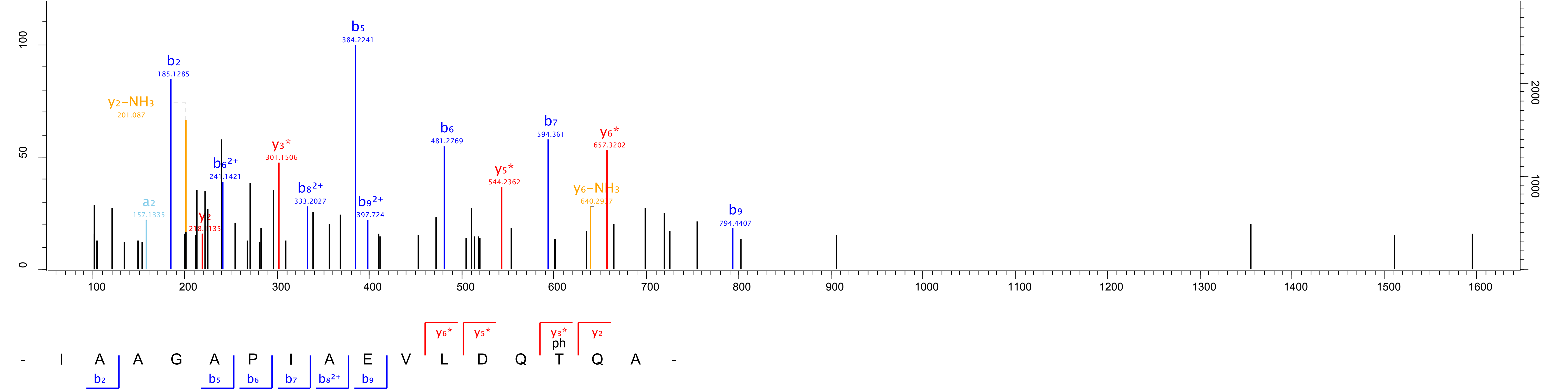

Raw file	Scan	Method	Score	m/z
20101013_Velos3_NaNa_COLLAB_salvage_5527_02	7218	FTMS; HCD	131.72	755.85

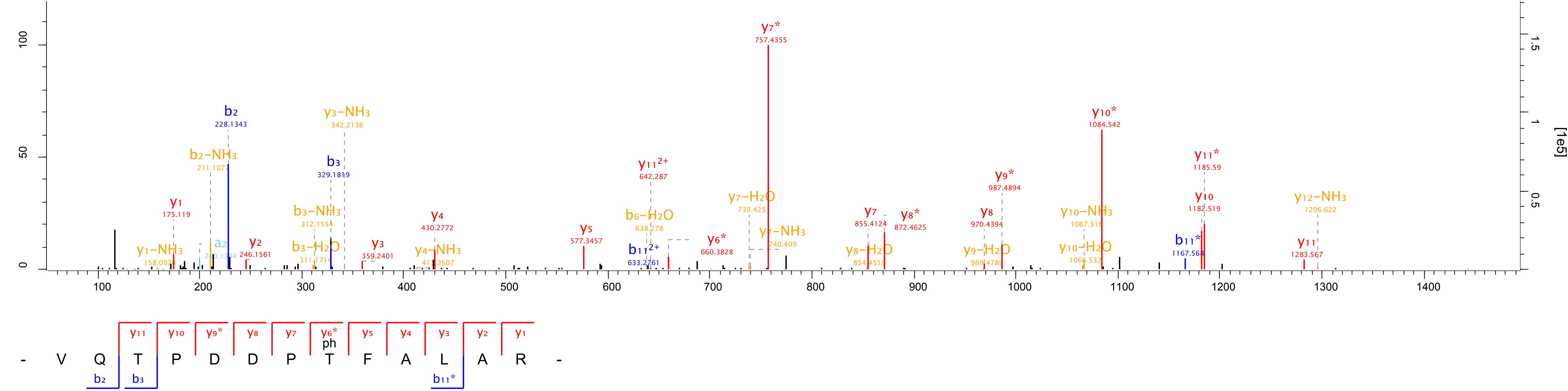

Raw file	Scan	Method	Score	m/z
20101013_Velos3_NaNa_COLLAB_5527_rep_02_fraction03	3709	FTMS; HCD	90.72	557.27

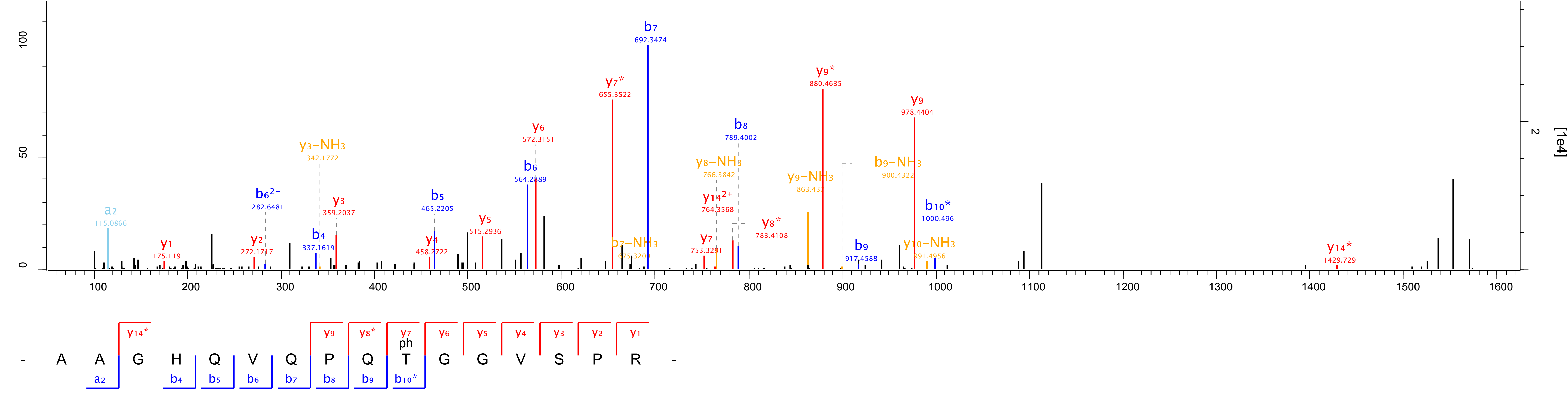

Raw file	Scan	Method	Score	m/z
20101013_Velos3_NaNa_COLLAB_5527_rep_03_fraction02	13194	FTMS; HCD	188.96	525.57

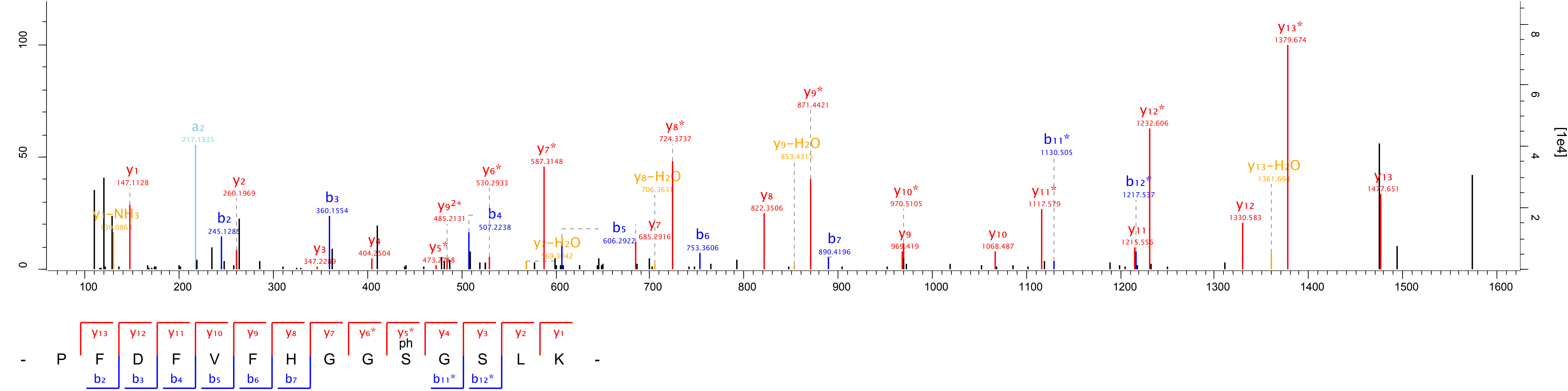

Raw file	Scan	Method	Score	m/z
20101013_Velos3_NaNa_COLLAB_salvage_5527_02	7728	FTMS; HCD	151.03	718.83

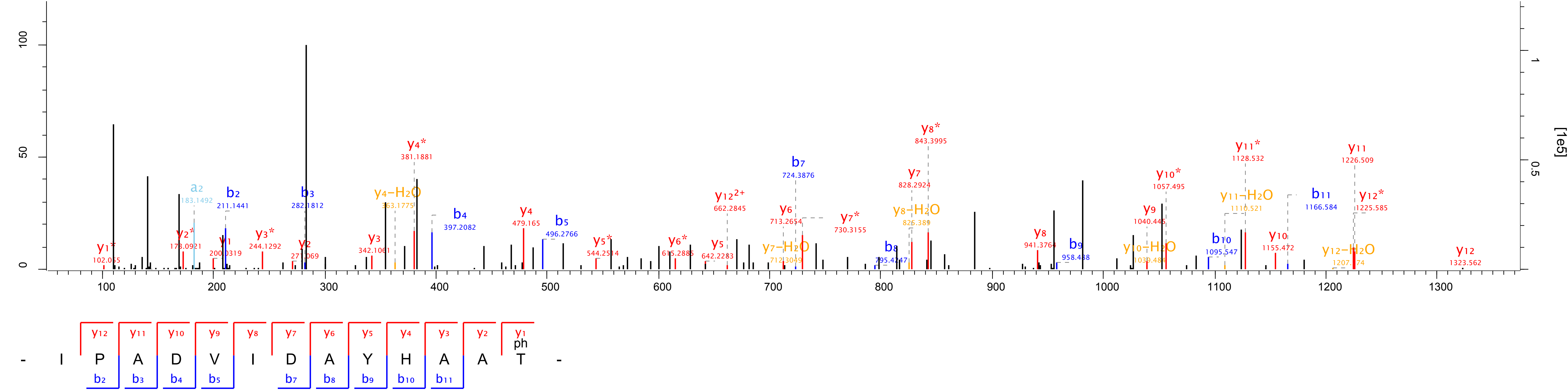

Raw file	Scan	Method	Score	m/z
20101013_Velos3_NaNa_COLLAB_5527_rep_03_fraction03	4682	FTMS; HCD	95.62	452.74

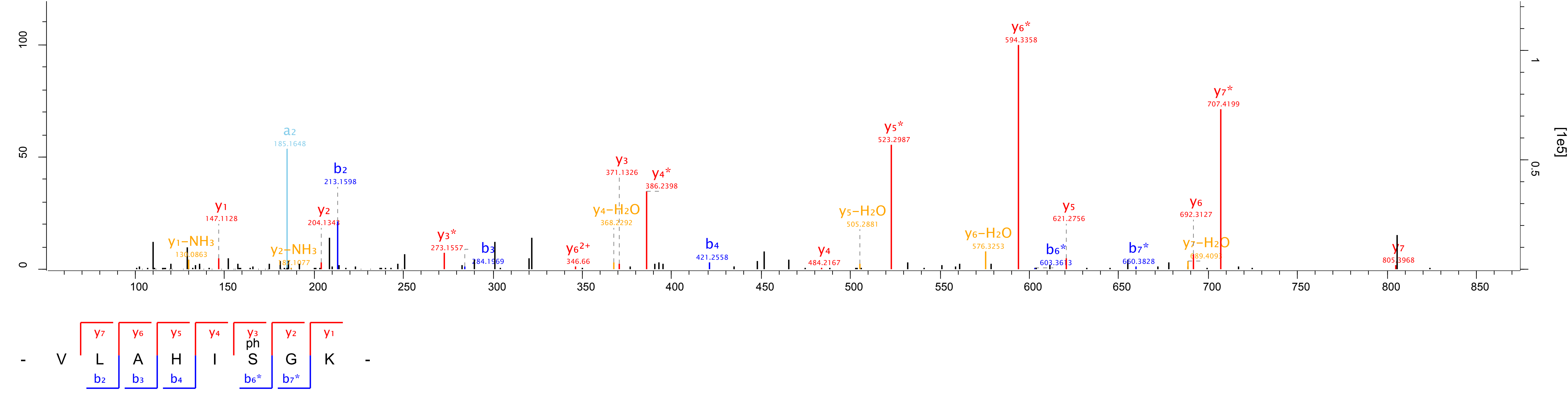

Raw file	Scan	Method	Score	m/z
20101013_Velos3_NaNa_COLLAB_5527_rep_01_fraction04	7486	FTMS; HCD	94.63	677.29

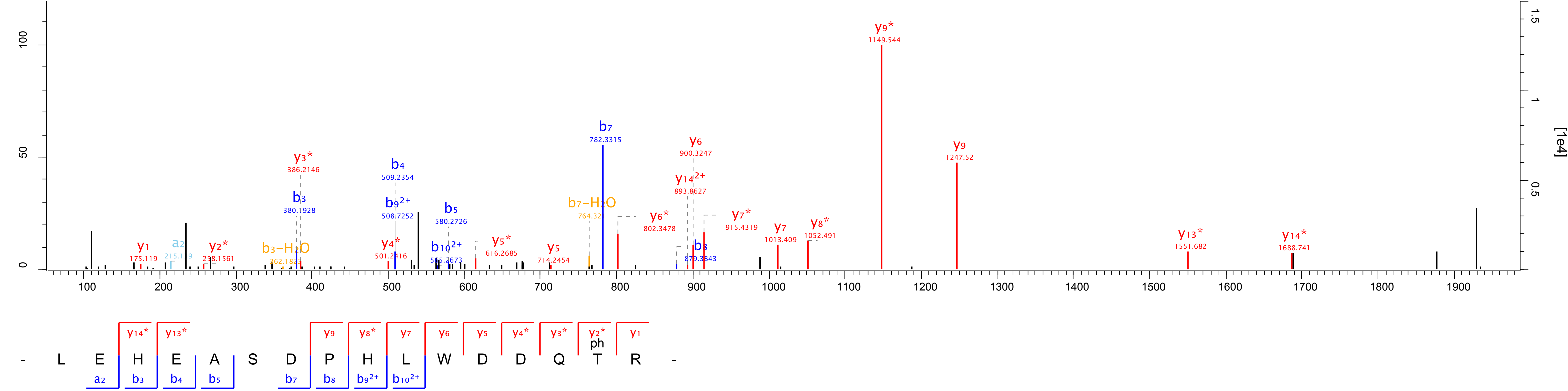

Raw file	Scan	Method	Score	m/z
20101013_Velos3_NaNa_COLLAB_5527_rep_01_flowthru_03	7065	FTMS; HCD	86.94	629.78

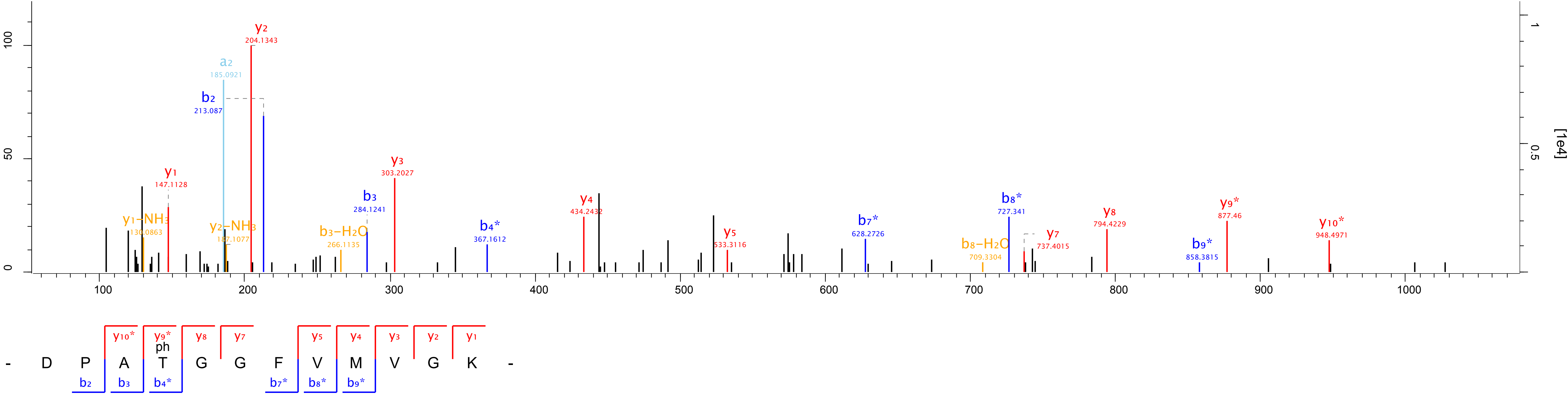

Raw file	Scan	Method	Score	m/z
20101013_Velos3_NaNa_COLLAB_5527_rep_03_flowthru_01	4212	FTMS; HCD	59.12	571.73

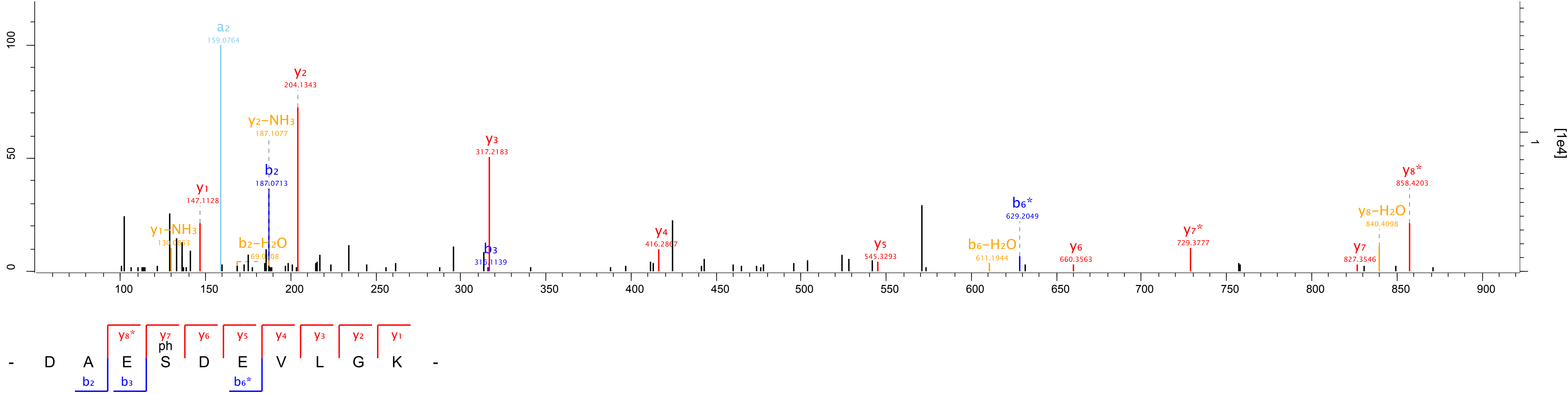

Raw file	Scan	Method	Score	m/z
20101013_Velos3_NaNa_COLLAB_5527_rep_02_fraction02	11837	FTMS; HCD	90.38	613.63

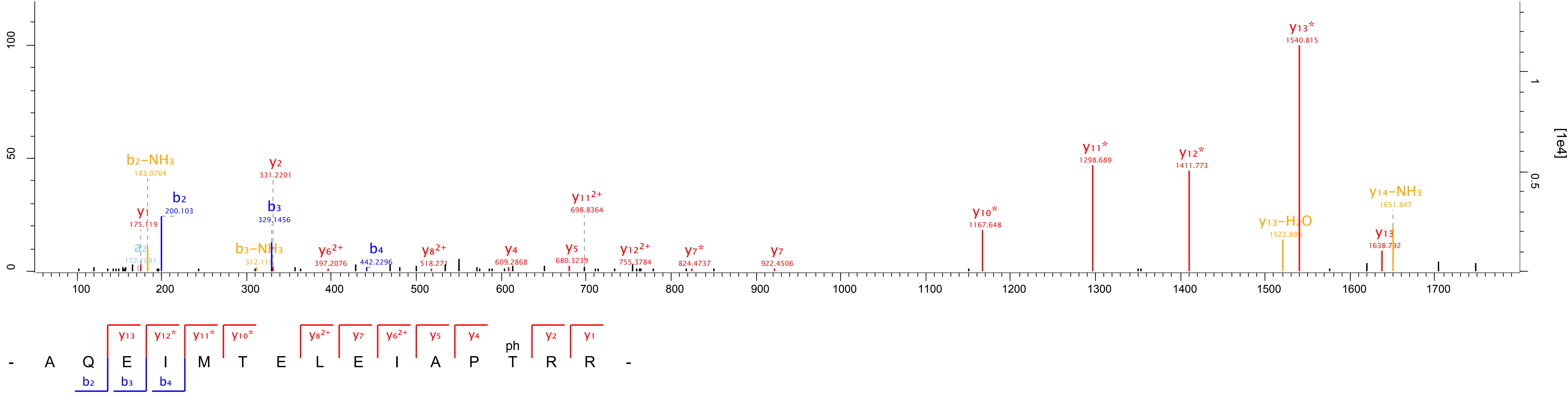

Raw file	Scan	Method	Score	m/z
20101013_Velos3_NaNa_COLLAB_5527_rep_02_fraction02	10491	FTMS; HCD	149.53	668.63

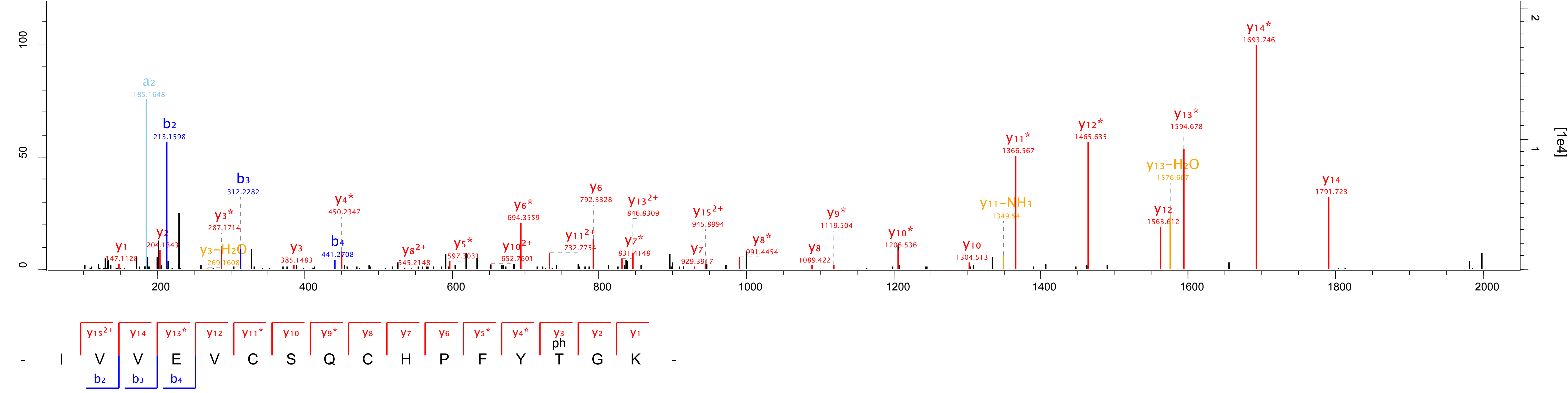

Raw file	Scan	Method	Score	m/z
20101013_Velos3_NaNa_COLLAB_salvage_5527_03	3127	FTMS; HCD	94.09	405.87

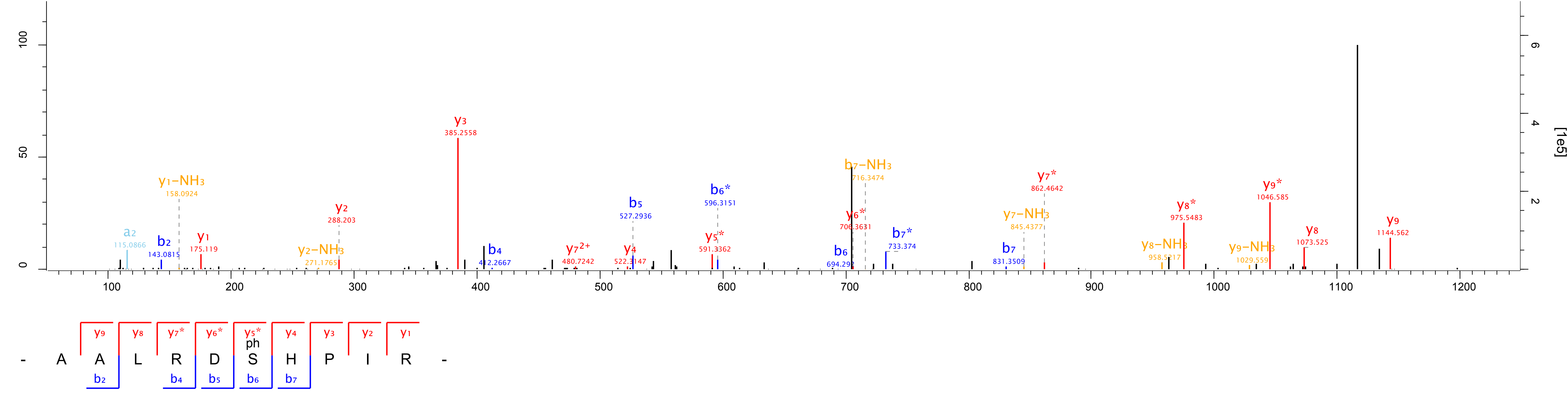

Raw file	Scan	Method	Score	m/z
20101013_Velos3_NaNa_COLLAB_salvage_5527_02	3025	FTMS; HCD	110.55	664.23

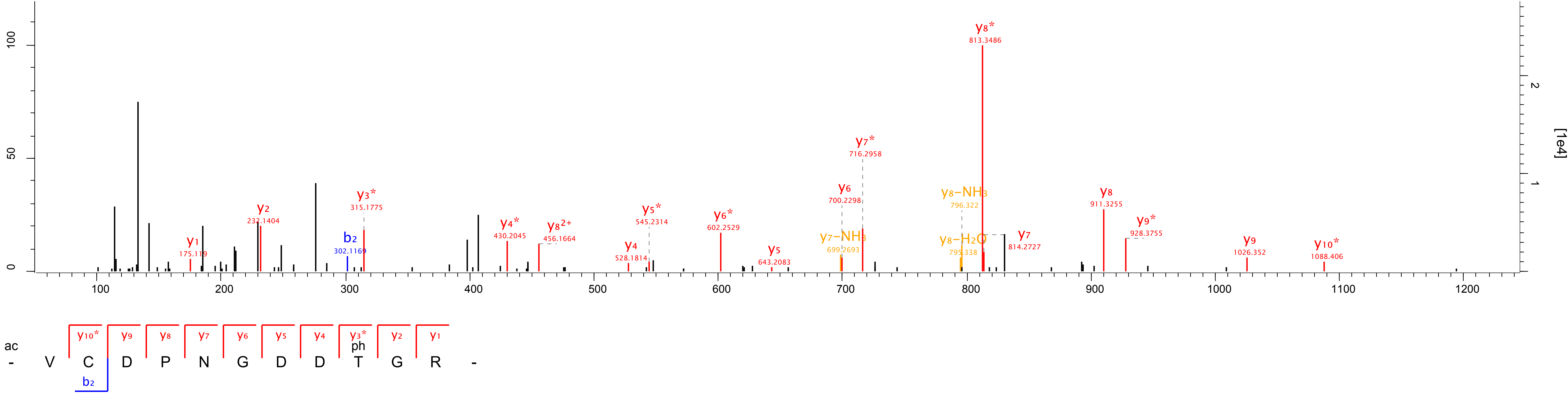

Raw file	Scan	Method	Score	m/z
20101013_Velos3_NaNa_COLLAB_5527_rep_03_fraction01	2859	FTMS; HCD	73.88	546.72

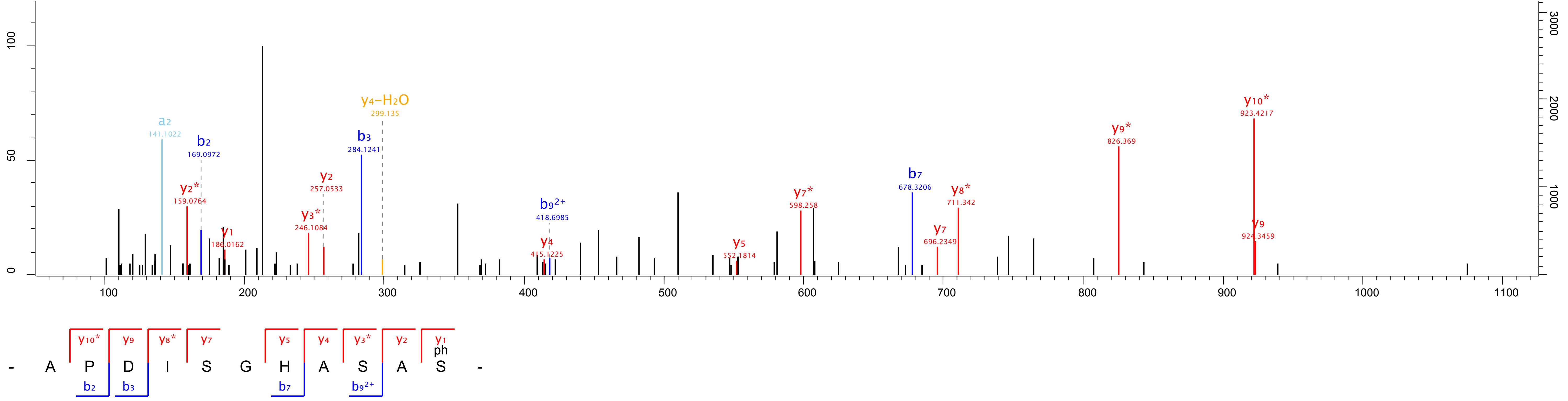

Raw file	Scan	Method	Score	m/z
20101013_Velos3_NaNa_COLLAB_5527_rep_01_flowthru_01	5284	FTMS; HCD	123.29	769.84

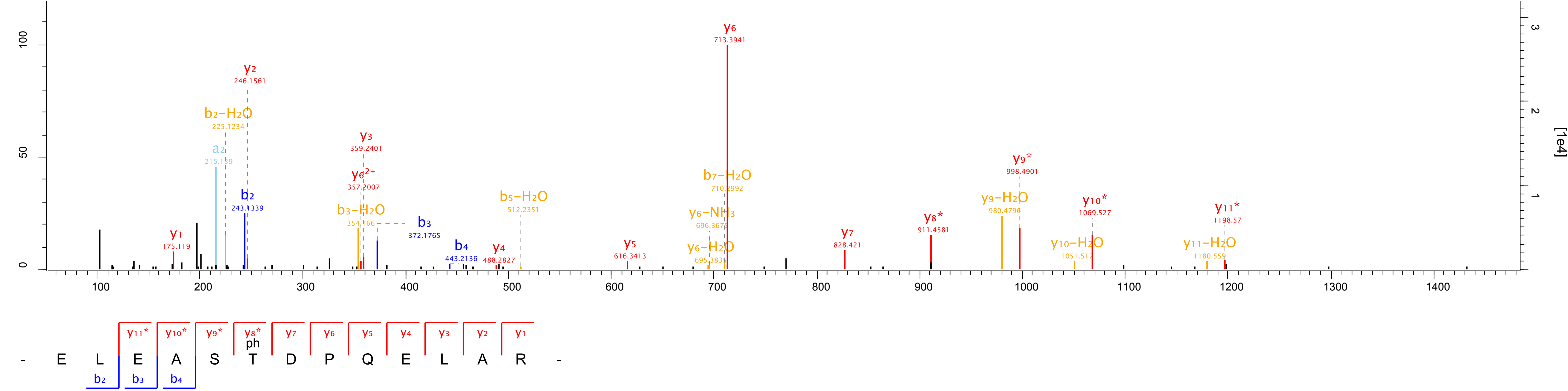

Raw file	Scan	Method	Score	m/z
20101013_Velos3_NaNa_COLLAB_5527_rep_01_fraction03	12889	FTMS; HCD	82.41	723.35

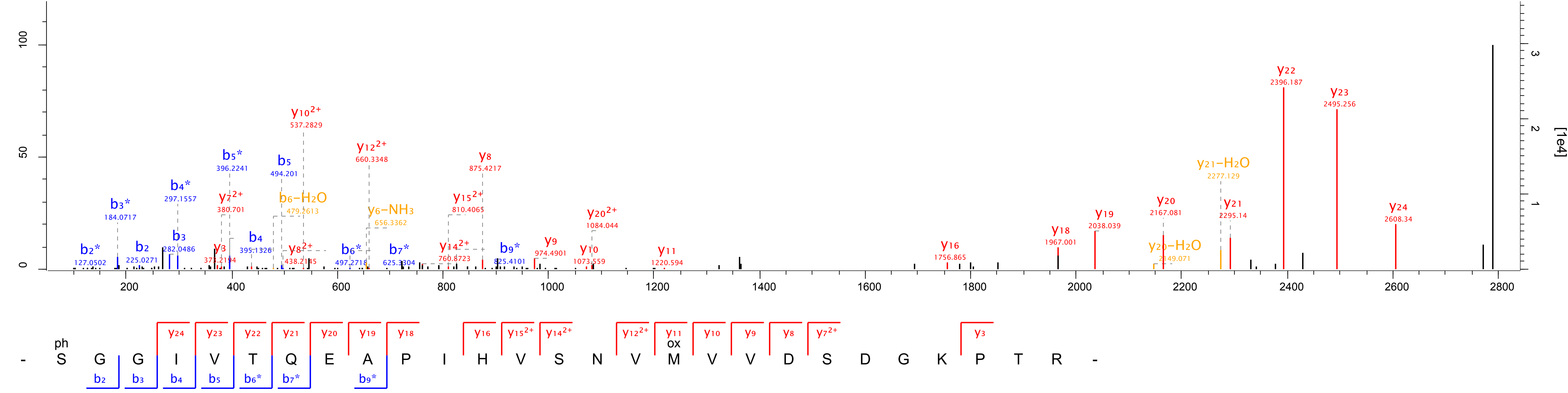

Raw file	Scan	Method	Score	m/z
20101013_Velos3_NaNa_COLLAB_5527_rep_03_flowthru_01	5665	FTMS; HCD	89.31	677.3

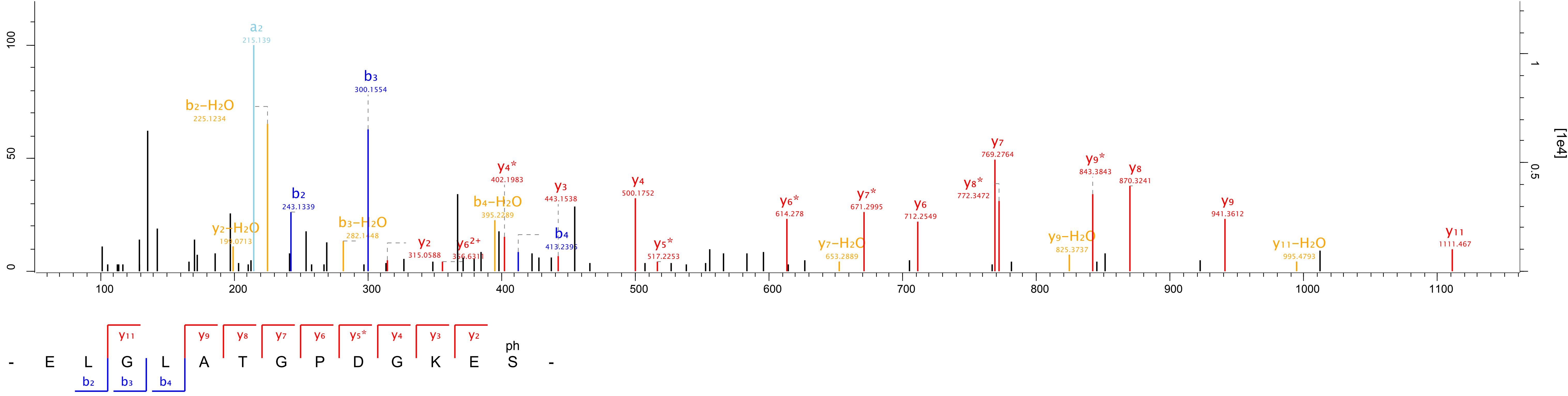

Raw file	Scan	Method	Score	m/z
20101013_Velos3_NaNa_COLLAB_5527_rep_02_flowthru_01	12773	FTMS; HCD	43.16	740.69

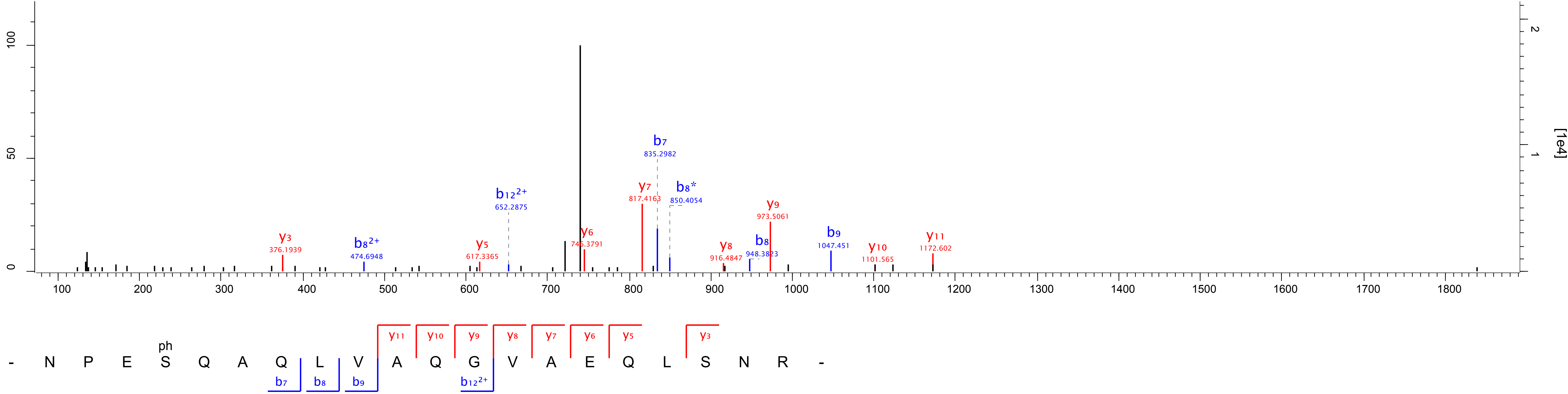

Raw file	Scan	Method	Score	m/z
20101013_Velos3_NaNa_COLLAB_salvage_5527_02	4683	FTMS; HCD	80.08	703.96

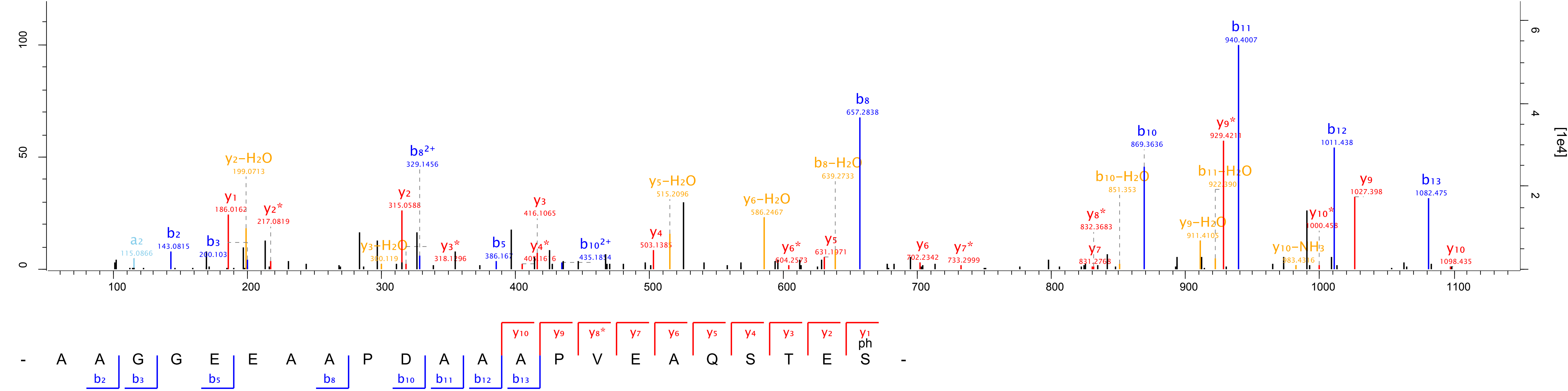

Raw file	Scan	Method	Score	m/z
20101013_Velos3_NaNa_COLLAB_5527_rep_03_fraction04	9873	FTMS; HCD	62.86	549.6

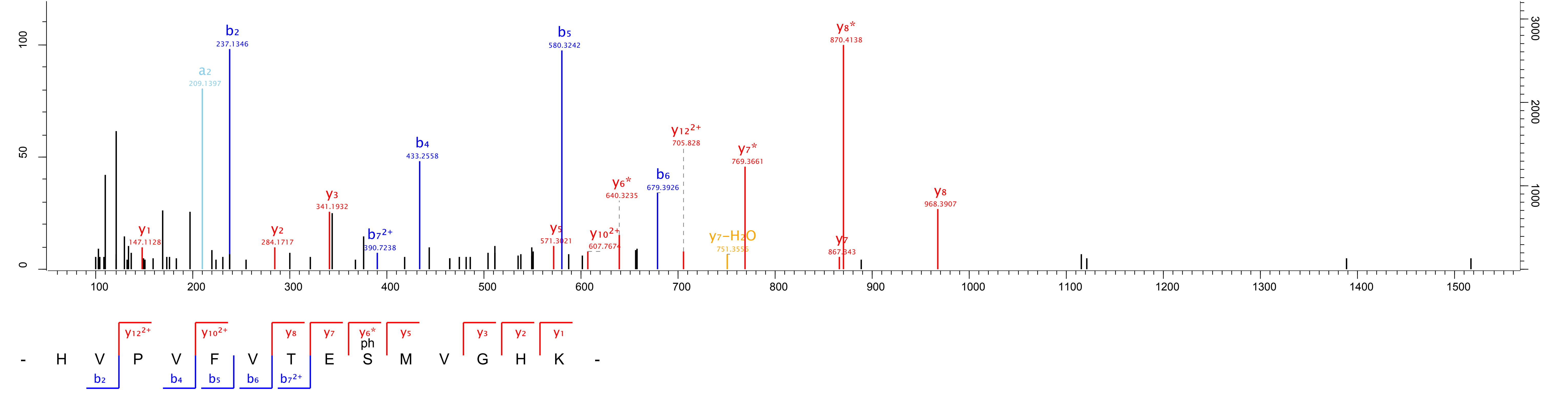

Raw file	Scan	Method	Score	m/z
20101013_Velos3_NaNa_COLLAB_salvage_5527_02	4167	FTMS; HCD	87.15	464.72

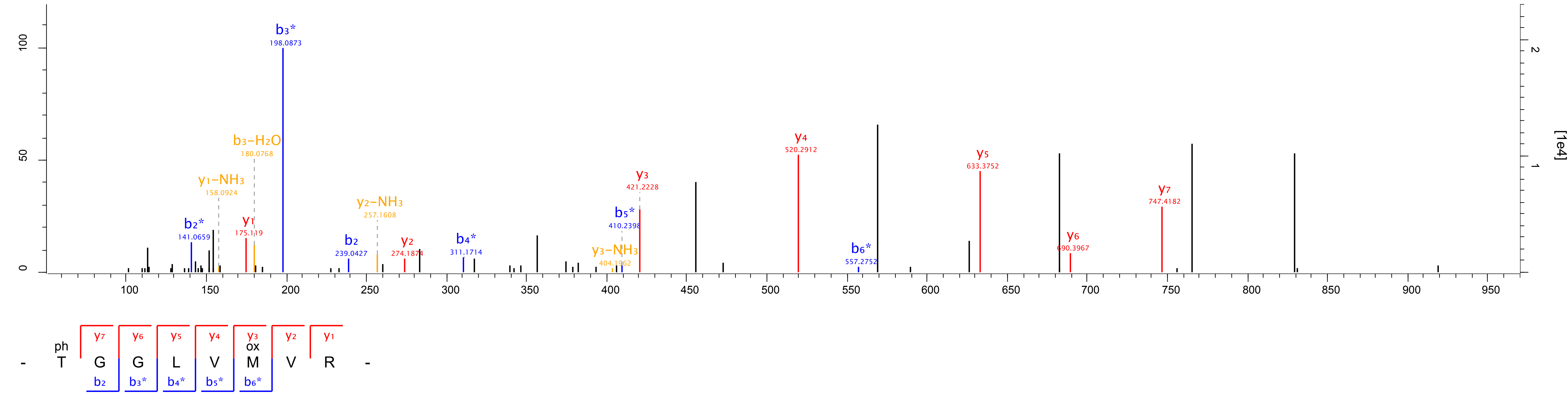

Raw file	Scan	Method	Score	m/z
20101013_Velos3_NaNa_COLLAB_salvage_5527_01	6965	FTMS; HCD	84.51	545.93

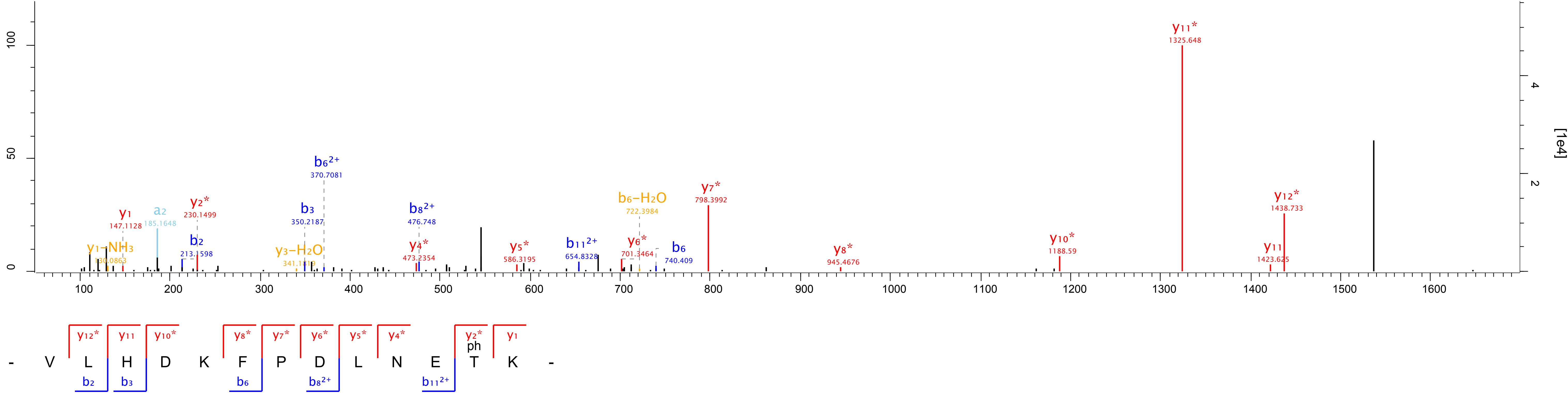

Raw file	Scan	Method	Score	m/z
20101013_Velos3_NaNa_COLLAB_5527_rep_03_fraction01	4118	FTMS; HCD	115.78	484.73

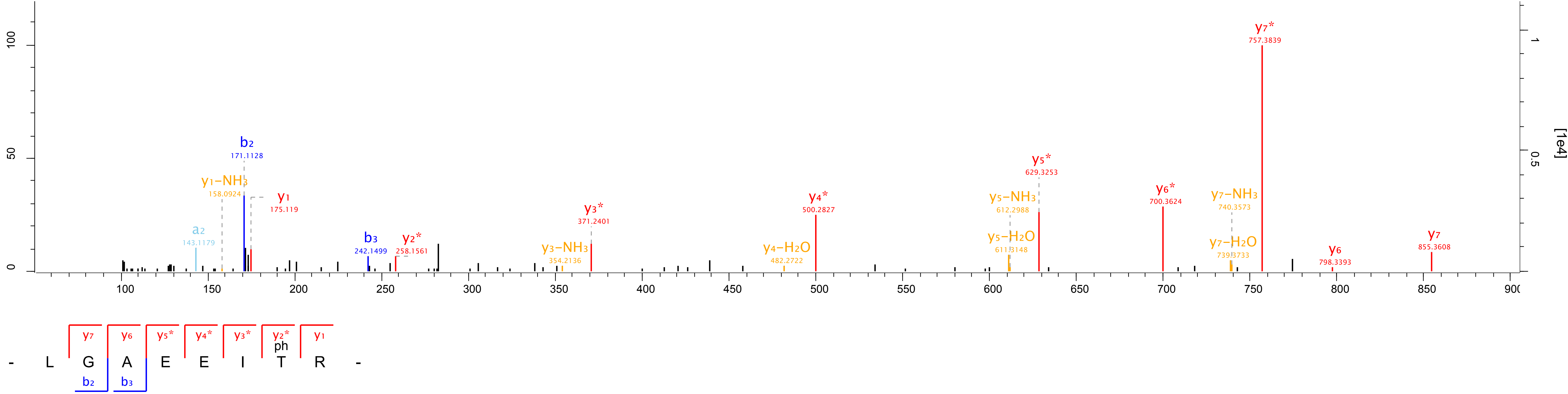

Raw file	Scan	Method	Score	m/z
20101013_Velos3_NaNa_COLLAB_5527_rep_01_flowthru_03	11138	FTMS; HCD	94.28	772.39

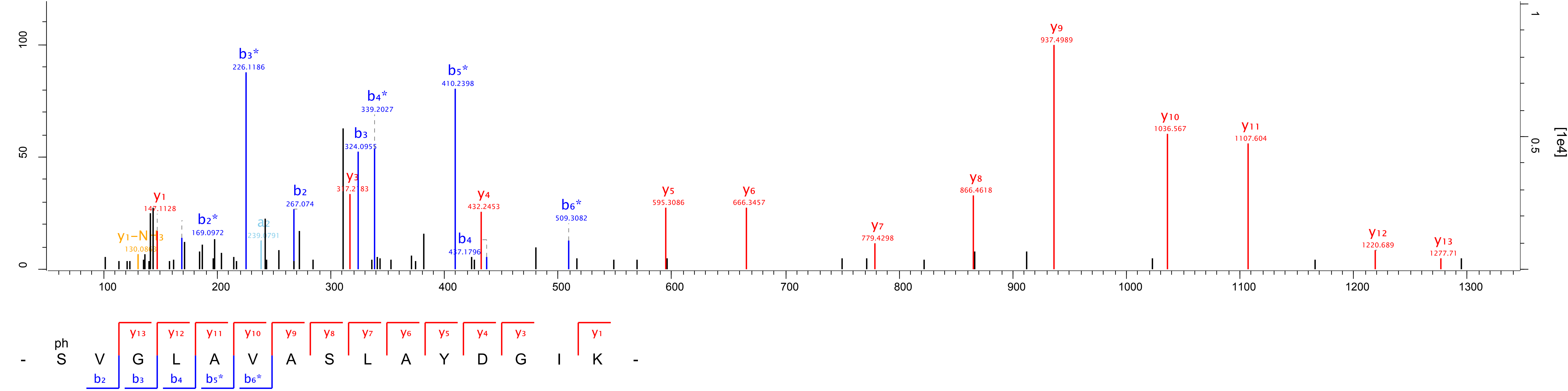

Raw file	Scan	Method	Score	m/z
20101013_Velos3_NaNa_COLLAB_5527_rep_03_fraction01	1582	FTMS; HCD	114.02	446.17

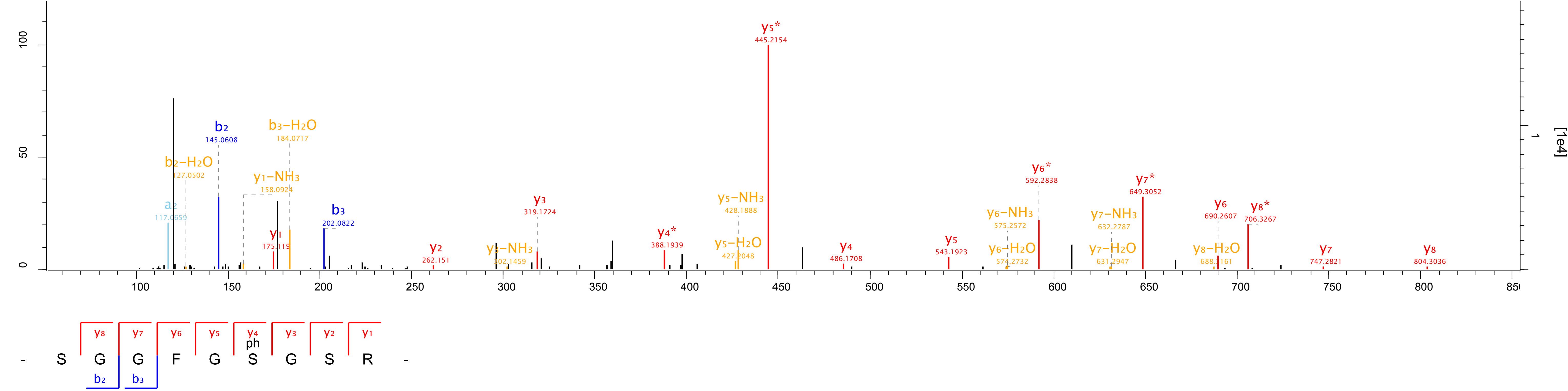

Raw file	Scan	Method	Score	m/z
20101013_Velos3_NaNa_COLLAB_5527_rep_02_fraction02	13029	FTMS; HCD	91.66	837.07

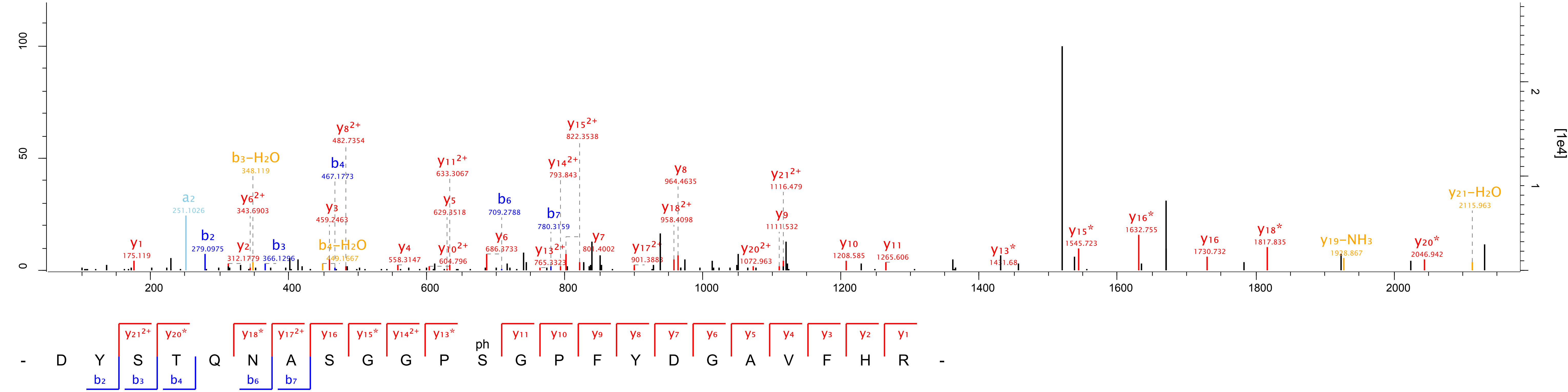

Raw file	Scan	Method	Score	m/z
20101013_Velos3_NaNa_COLLAB_salvage_5527_02	6957	FTMS; HCD	60.36	644.26

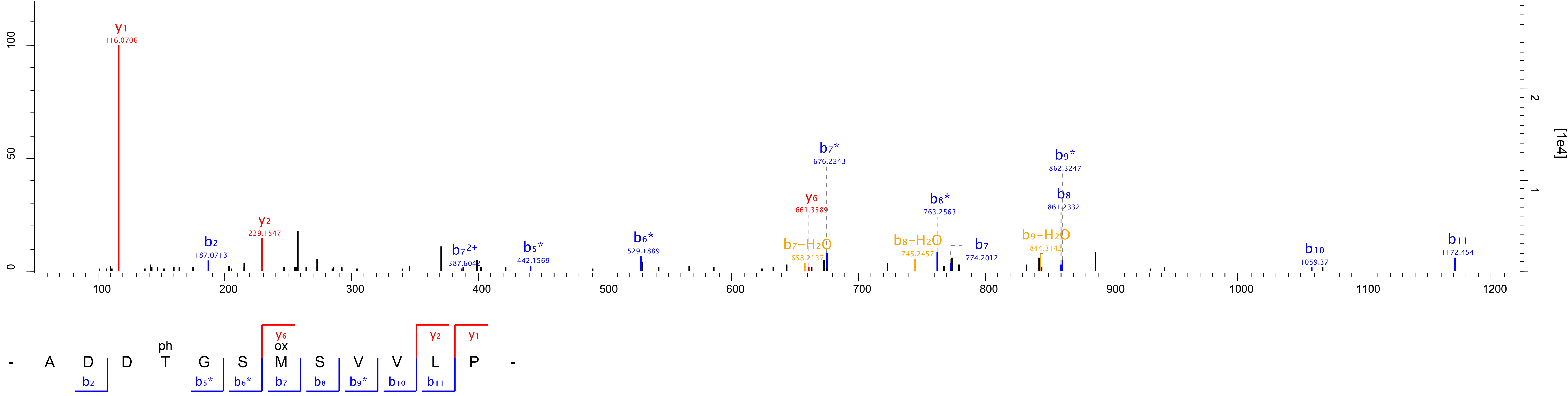

Raw file	Scan	Method	Score	m/z
20101013_Velos3_NaNa_COLLAB_5527_rep_01_flowthru_01	7237	FTMS; HCD	91.24	1030.42

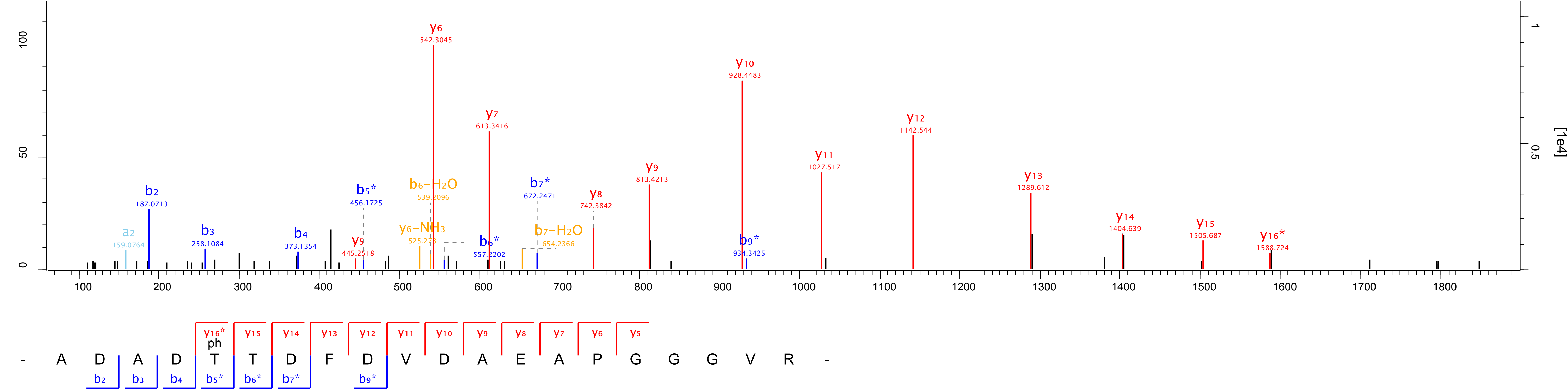

Raw file	Scan	Method	Score	m/z
20101013_Velos3_NaNa_COLLAB_5527_rep_02_fraction03	3911	FTMS; HCD	66.25	661.27

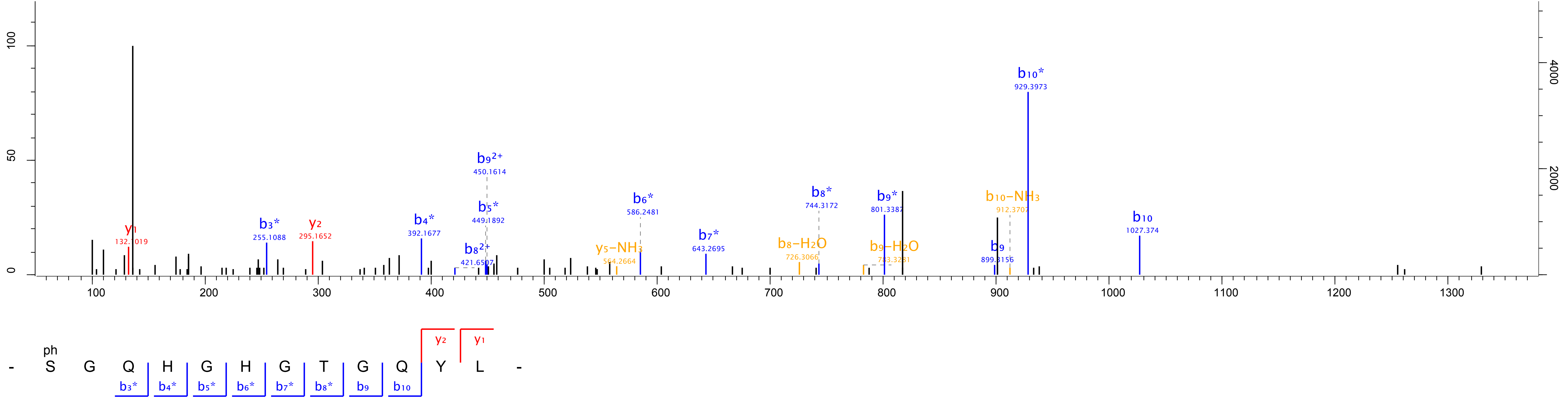

Raw file	Scan	Method	Score	m/z
20101013_Velos3_NaNa_COLLAB_salvage_5527_02	8807	FTMS; HCD	65.23	761.9

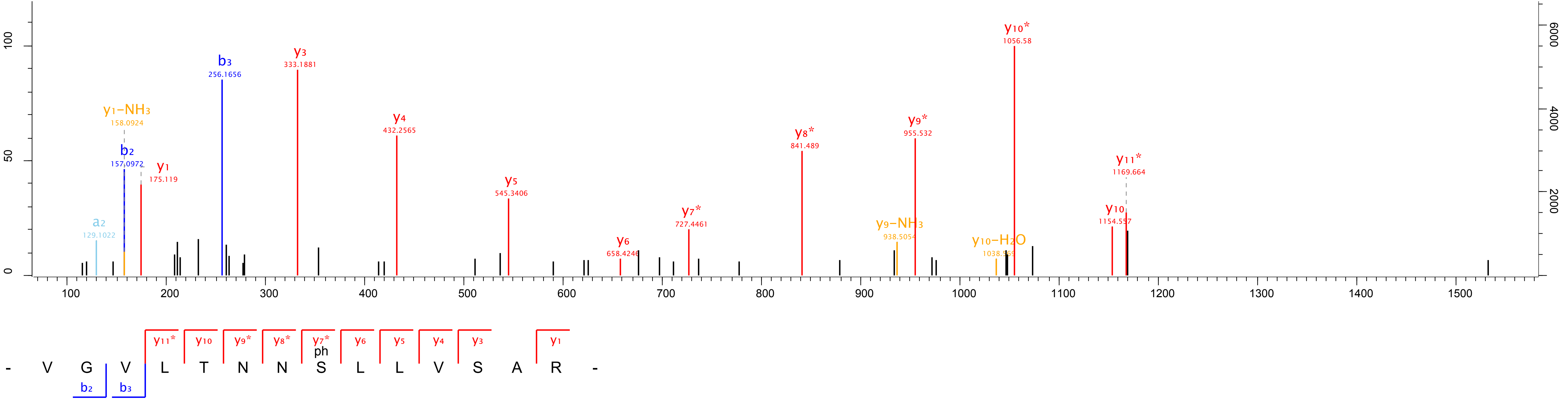

Raw file	Scan	Method	Score	m/z
20101013_Velos3_NaNa_COLLAB_5527_rep_03_flowthru_01	5290	FTMS; HCD	60.49	747.29

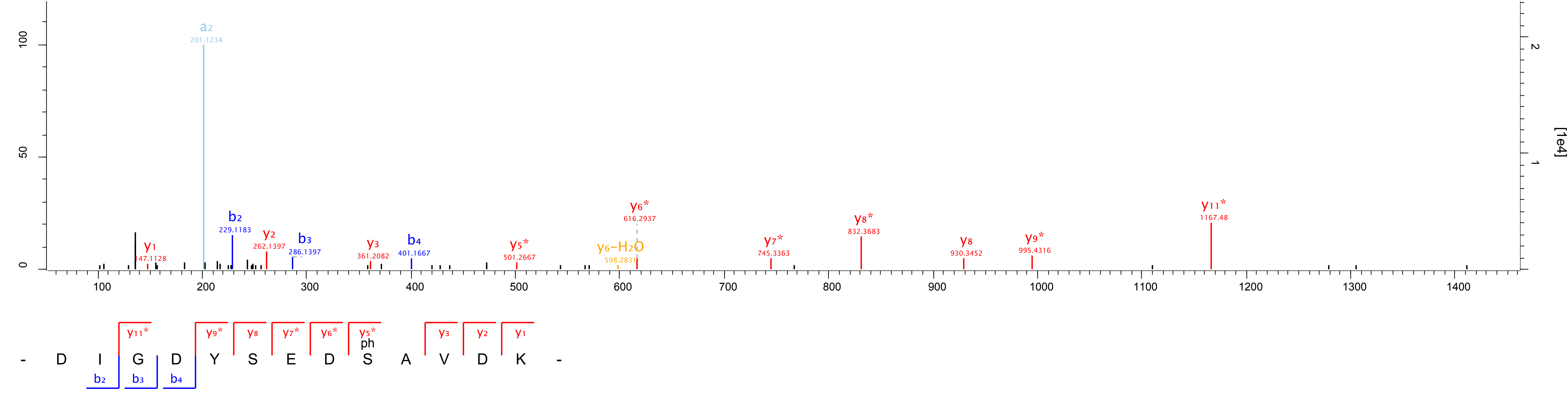

Raw file	Scan	Method	Score	m/z
20101013_Velos3_NaNa_COLLAB_salvage_5527_02	8426	FTMS; HCD	124.62	707.81

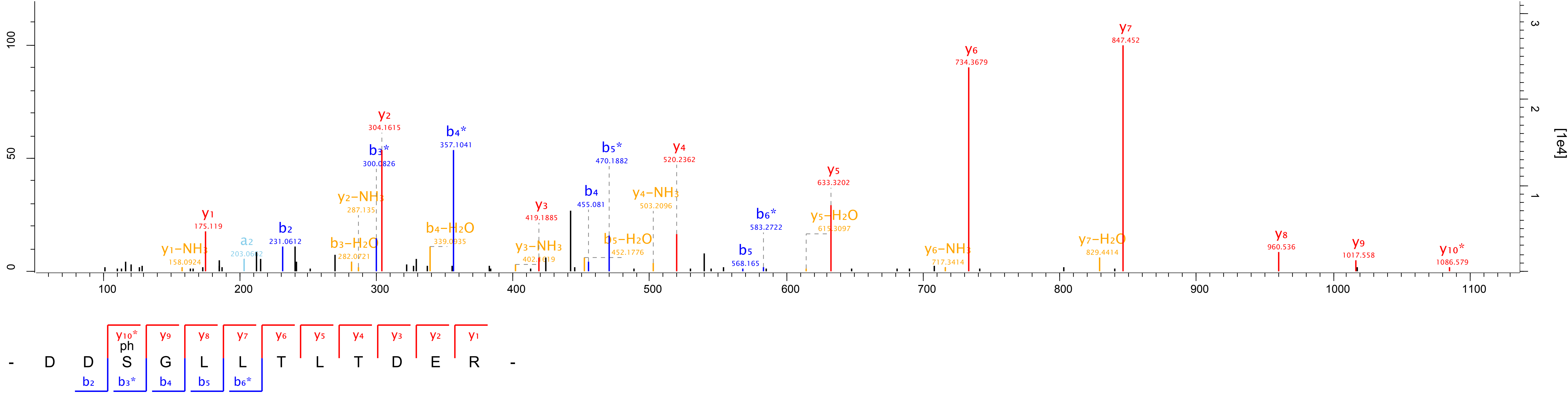

Raw file	Scan	Method	Score	m/z
20101013_Velos3_NaNa_COLLAB_5527_rep_03_flowthru_01	6462	FTMS; HCD	103.13	732.8

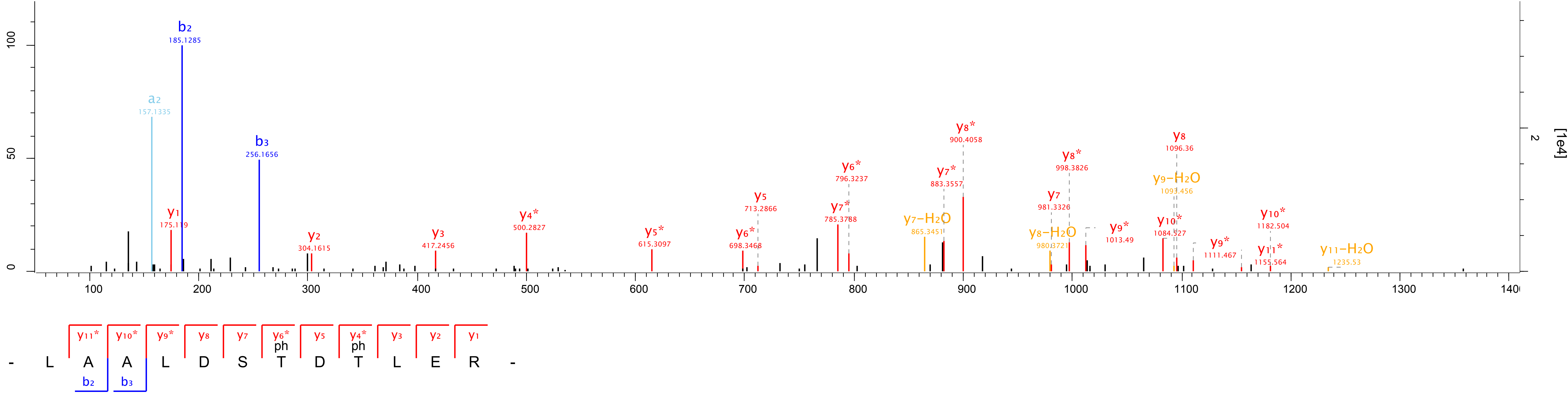

Raw file	Scan	Method	Score	m/z
20101013_Velos3_NaNa_COLLAB_5527_rep_01_flowthru_01	5001	FTMS; HCD	80.36	757.34

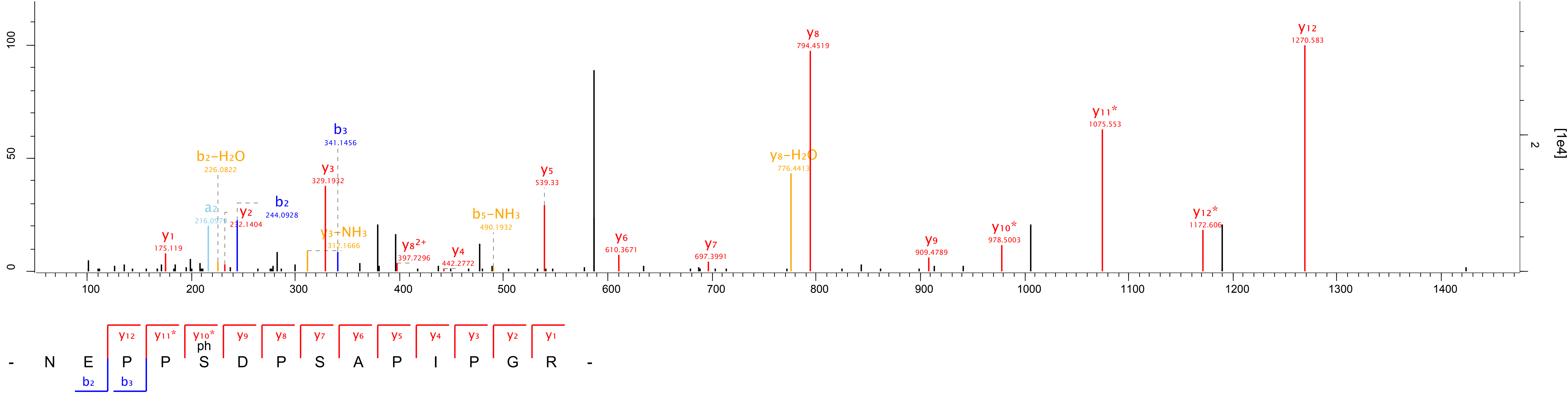

Raw file	Scan	Method	Score	m/z
20101013_Velos3_NaNa_COLLAB_5527_rep_03_fraction01	10300	FTMS; HCD	87.08	671.29

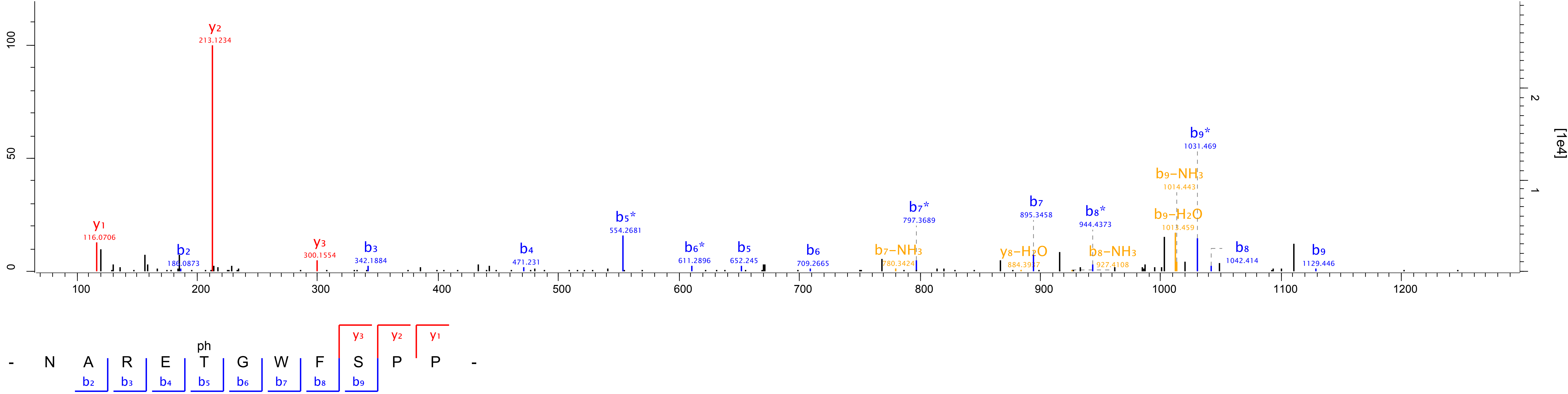

Raw file	Scan	Method	Score	m/z
20101013_Velos3_NaNa_COLLAB_salvage_5527_02	5900	FTMS; HCD	65.83	727.63

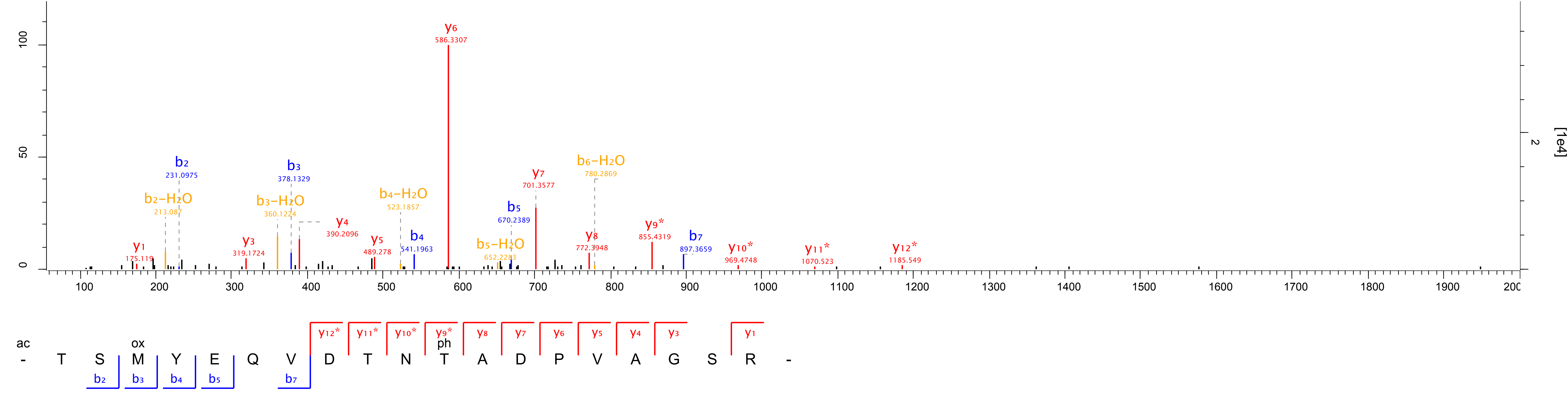

Raw file	Scan	Method	Score	m/z
20101013_Velos3_NaNa_COLLAB_salvage_5527_01	3701	FTMS; HCD	58.7	528.26

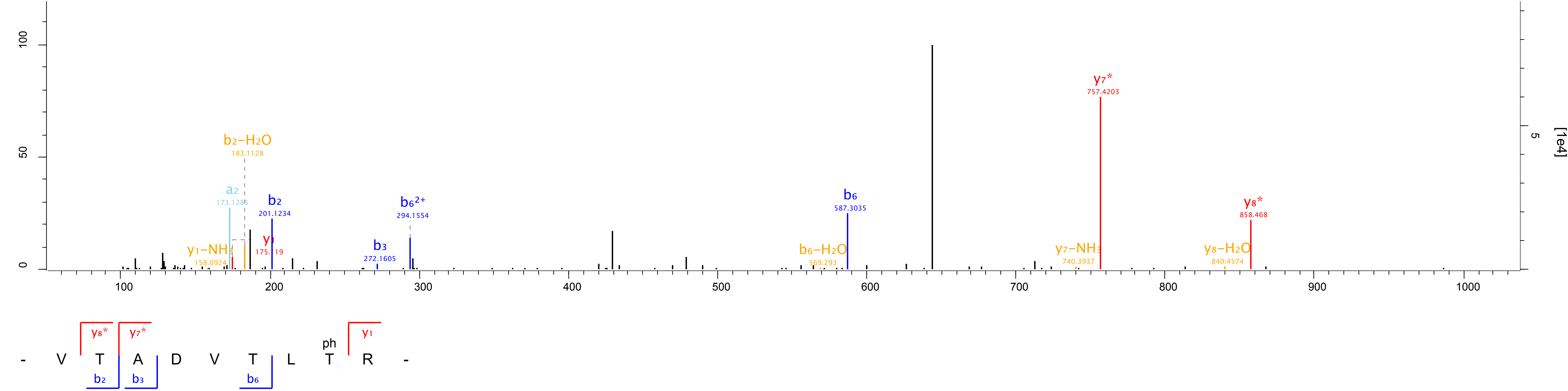

Raw file	Scan	Method	Score	m/z
20101013_Velos3_NaNa_COLLAB_5527_rep_01_flowthru_01	7331	FTMS; HCD	70.12	634.96

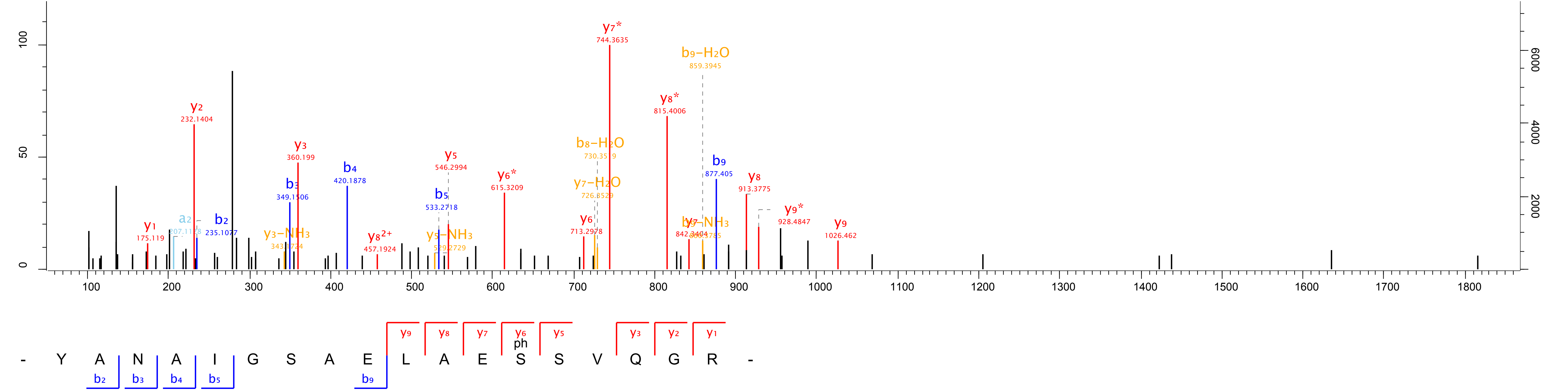

Raw file	Scan	Method	Score	m/z
20101013_Velos3_NaNa_COLLAB_5527_rep_01_flowthru_01	5195	FTMS; HCD	95.21	878.88

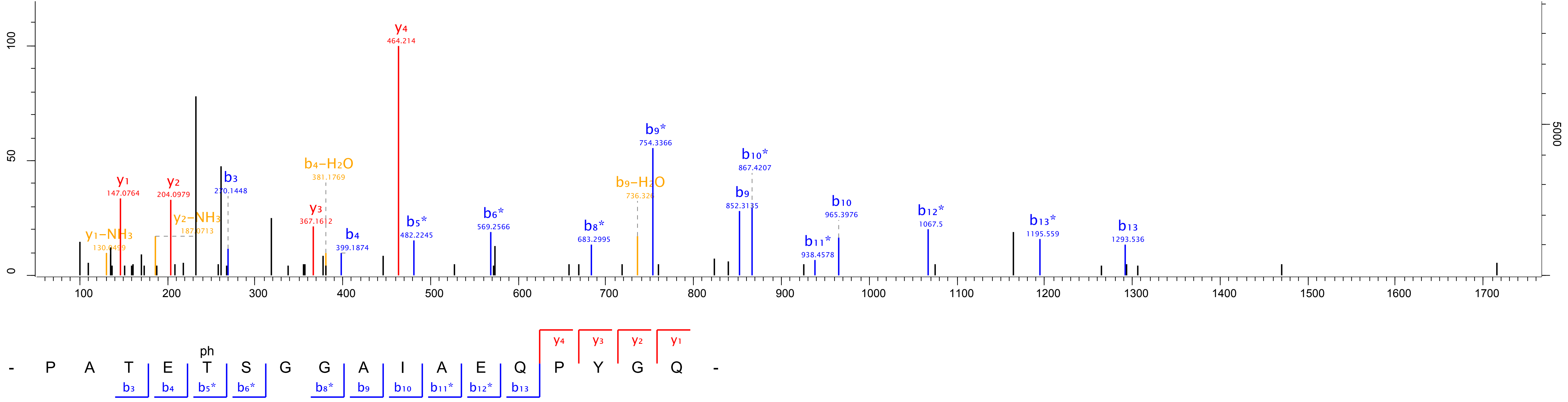

Raw file	Scan	Method	Score	m/z
20101013_Velos3_NaNa_COLLAB_salvage_5527_02	3673	FTMS; HCD	80.31	622.77

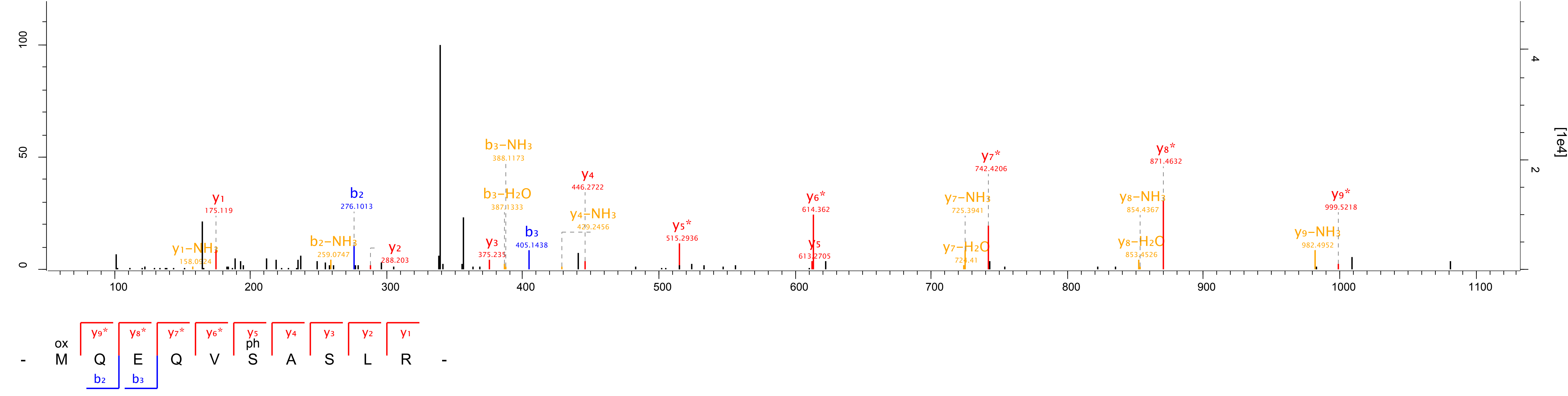

Raw file	Scan	Method	Score	m/z
20101013_Velos3_NaNa_COLLAB_5527_rep_03_flowthru_03	3574	FTMS; HCD	70.78	689.82

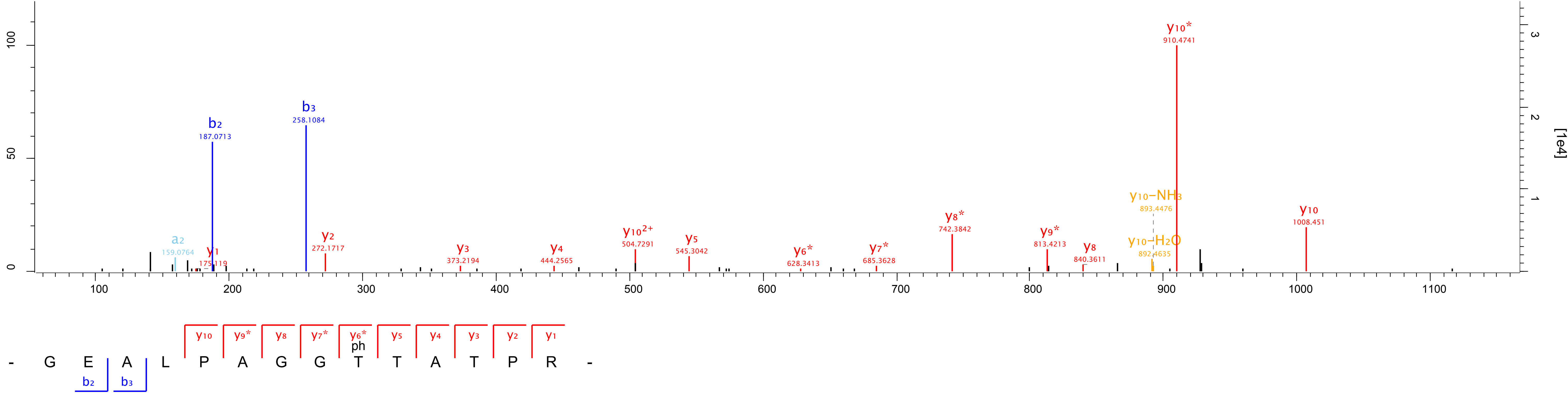

Raw file	Scan	Method	Score	m/z
20101013_Velos3_NaNa_COLLAB_salvage_5527_02	4136	FTMS; HCD	114.46	910.9

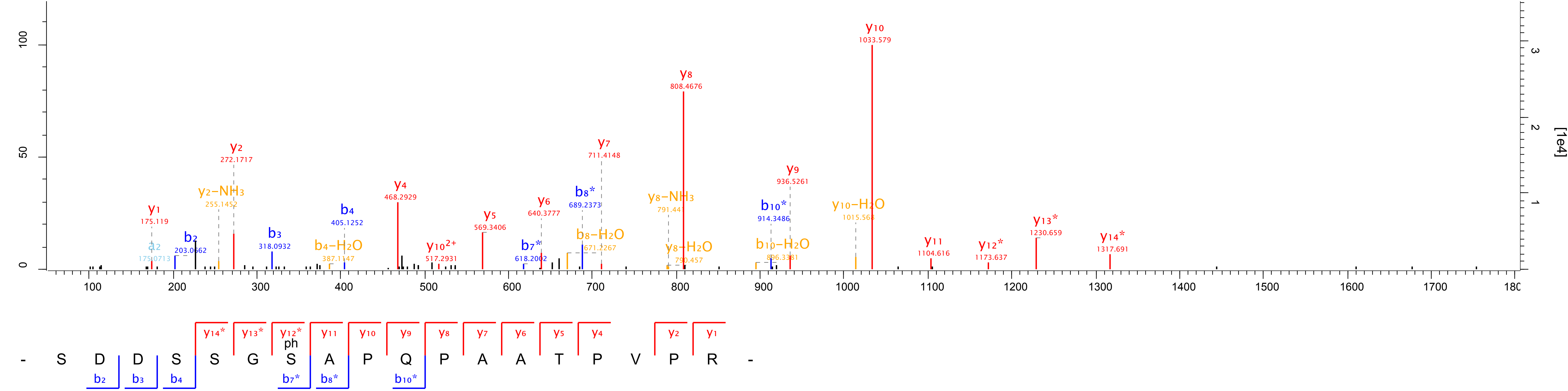

Raw file	Scan	Method	Score	m/z
20101013_Velos3_NaNa_COLLAB_salvage_5527_02	8928	FTMS; HCD	52.18	929.87

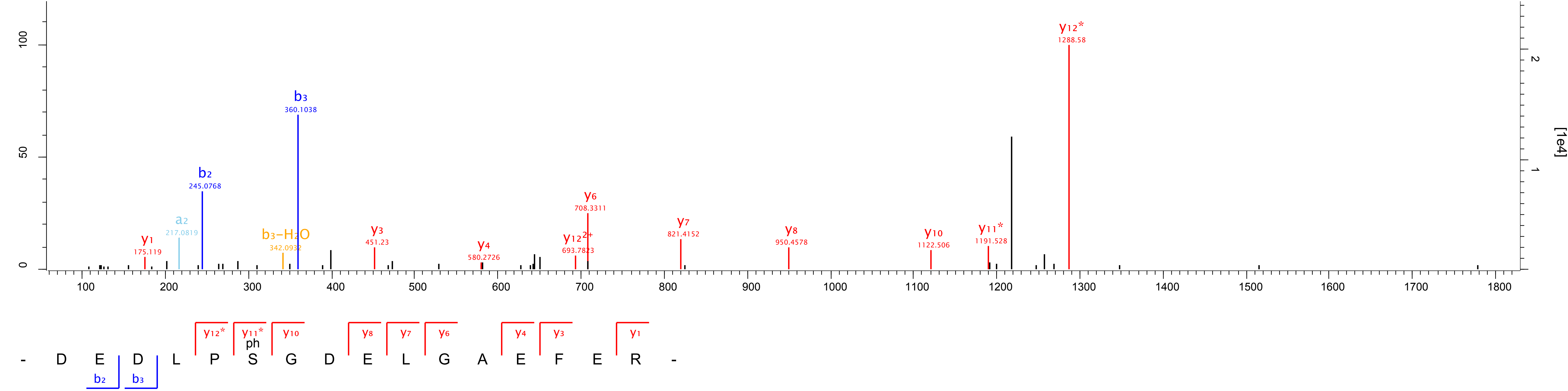

Raw file	Scan	Method	Score	m/z
20101013_Velos3_NaNa_COLLAB_5527_rep_03_fraction01	1385	FTMS; HCD	112.42	606.26

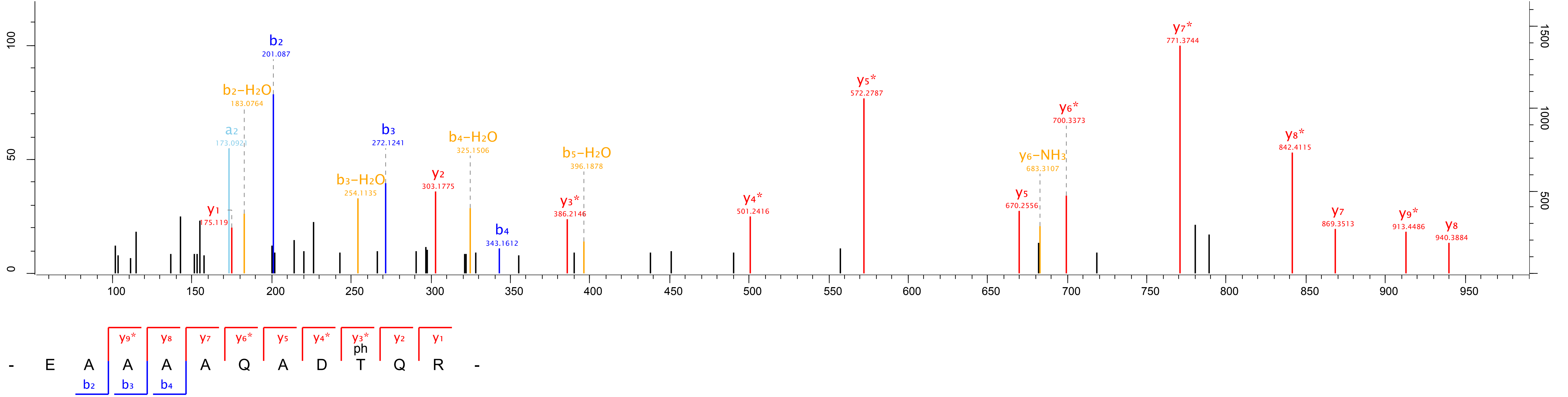

Raw file	Scan	Method	Score	m/z
20101013_Velos3_NaNa_COLLAB_salvage_5527_03	4969	FTMS; HCD	71.15	532.77

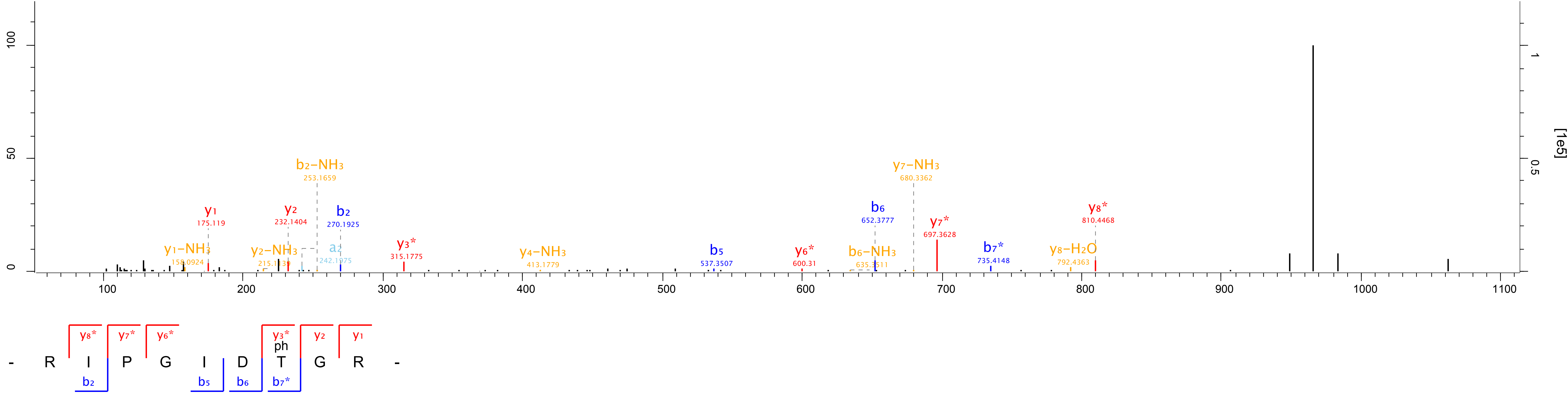

Raw file	Scan	Method	Score	m/z
20101013_Velos3_NaNa_COLLAB_5527_rep_01_flowthru_01	9226	FTMS; HCD	76.18	863.37

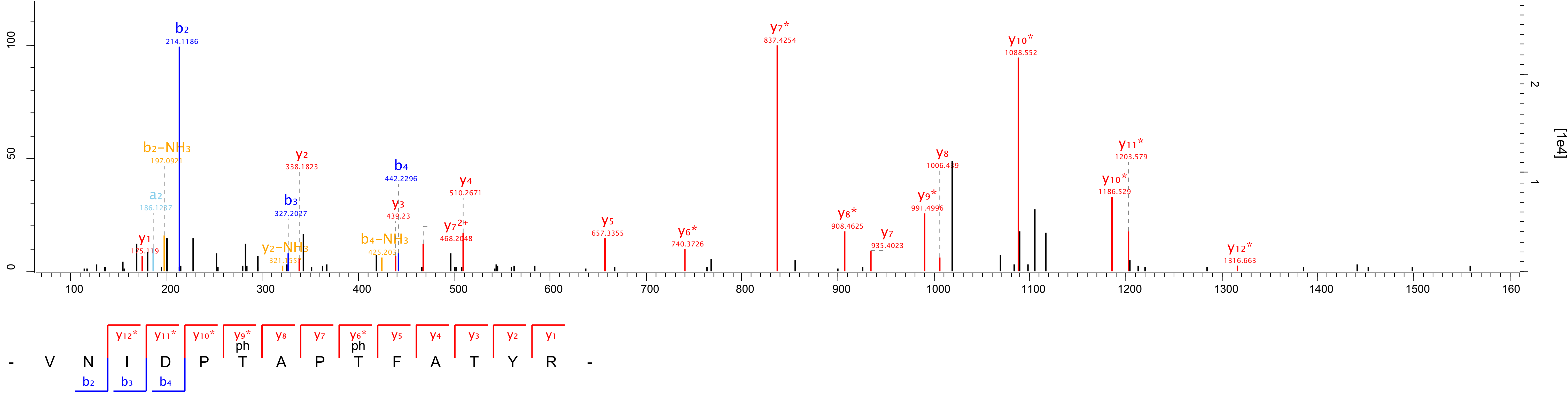

Raw file	Scan	Method	Score	m/z
20101013_Velos3_NaNa_COLLAB_5527_rep_02_flowthru_01	9261	FTMS; HCD	56.08	863.87

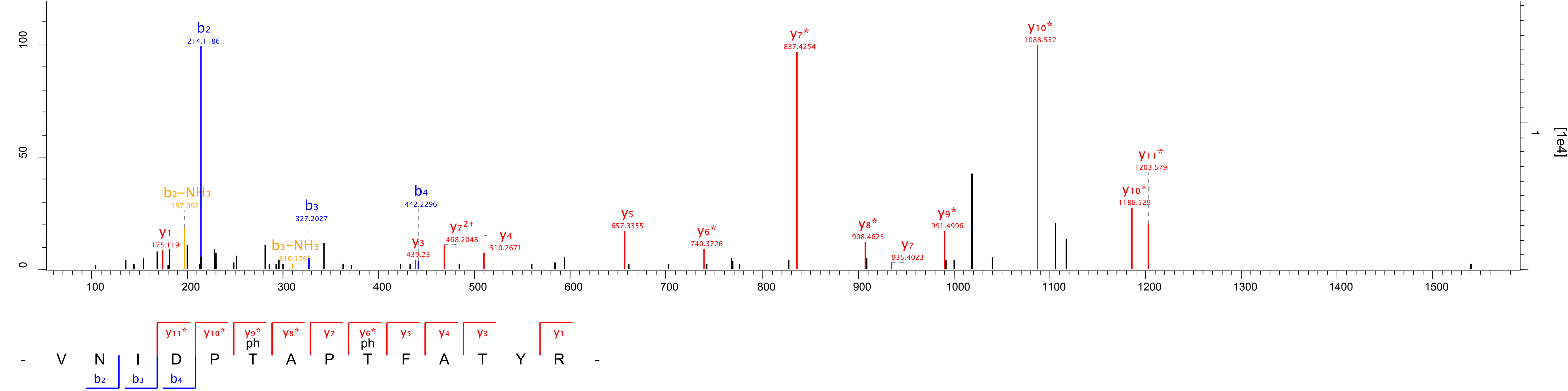

Raw file	Scan	Method	Score	m/z
20101013_Velos3_NaNa_COLLAB_5527_rep_02_flowthru_01	6188	FTMS; HCD	90.35	773.33

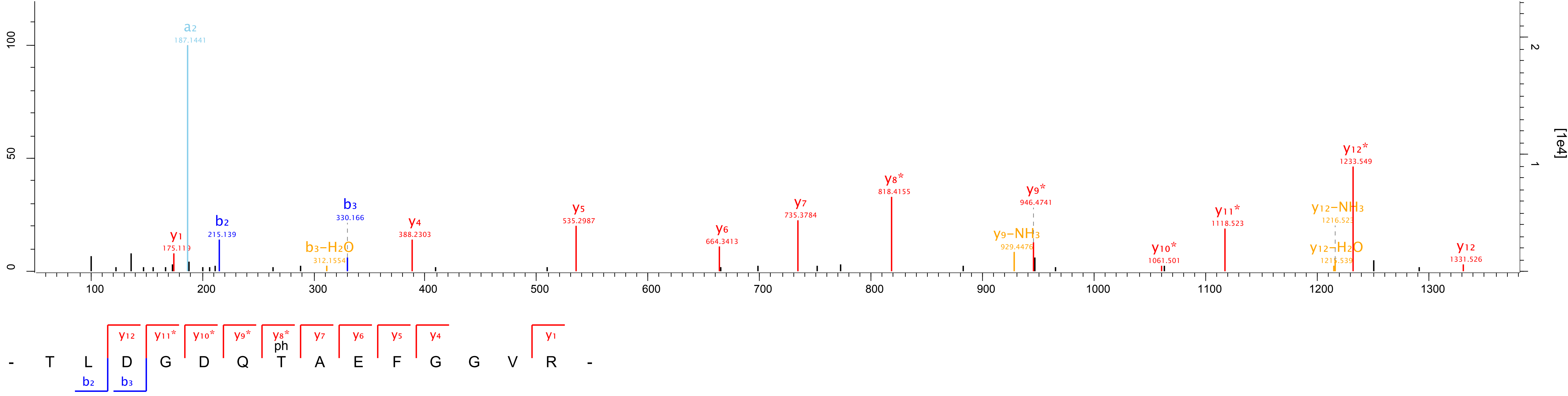

Raw file	Scan	Method	Score	m/z
20101013_Velos3_NaNa_COLLAB_salvage_5527_02	5605	FTMS; HCD	83.53	607.27

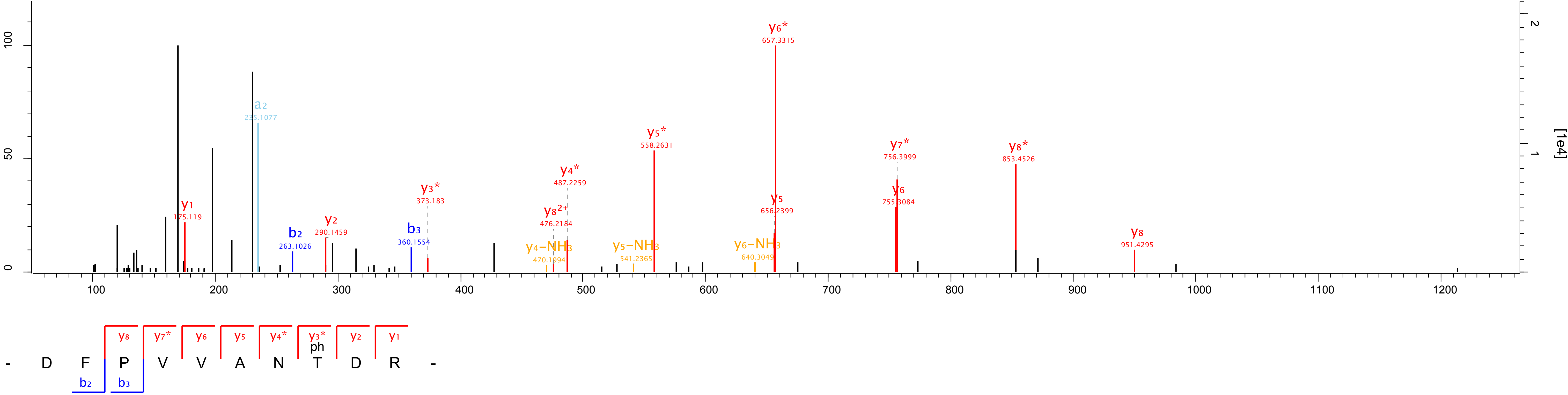

Raw file	Scan	Method	Score	m/z
20101013_Velos3_NaNa_COLLAB_5527_rep_03_flowthru_02	6107	FTMS; HCD	219.21	987.39

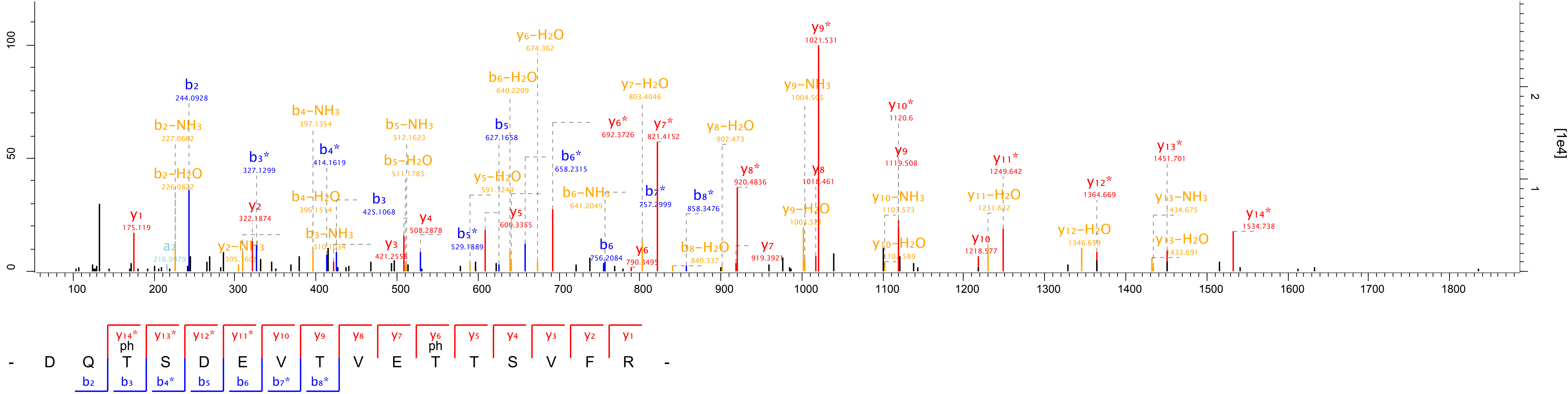

Raw file	Scan	Method	Score	m/z
20101013_Velos3_NaNa_COLLAB_salvage_5527_02	7461	FTMS; HCD	81.66	754.33

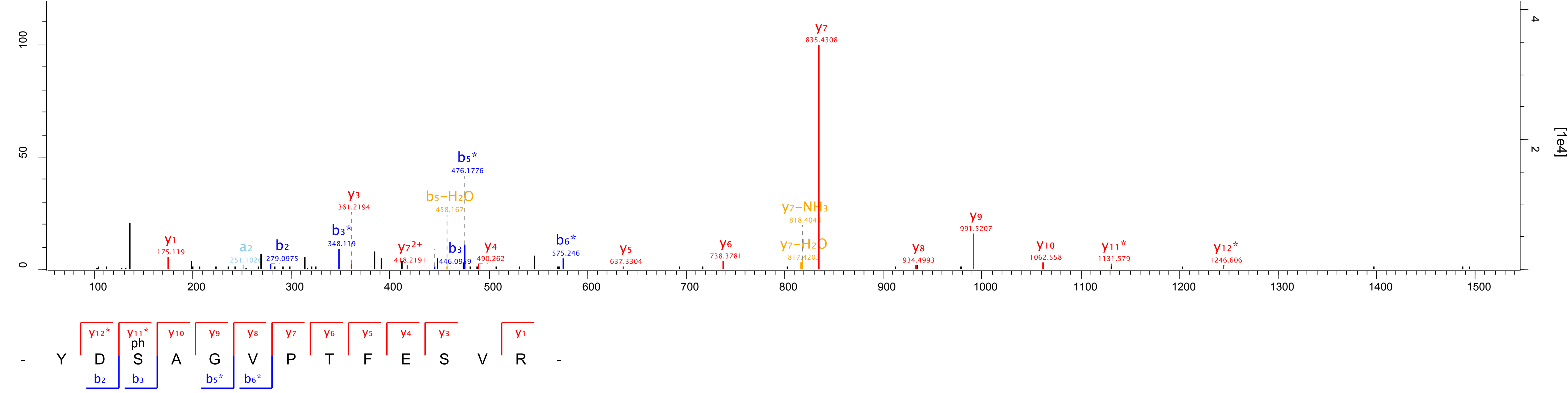

Raw file	Scan	Method	Score	m/z
20101013_Velos3_NaNa_COLLAB_5527_rep_01_fraction03	8898	FTMS; HCD	53.3	584.26

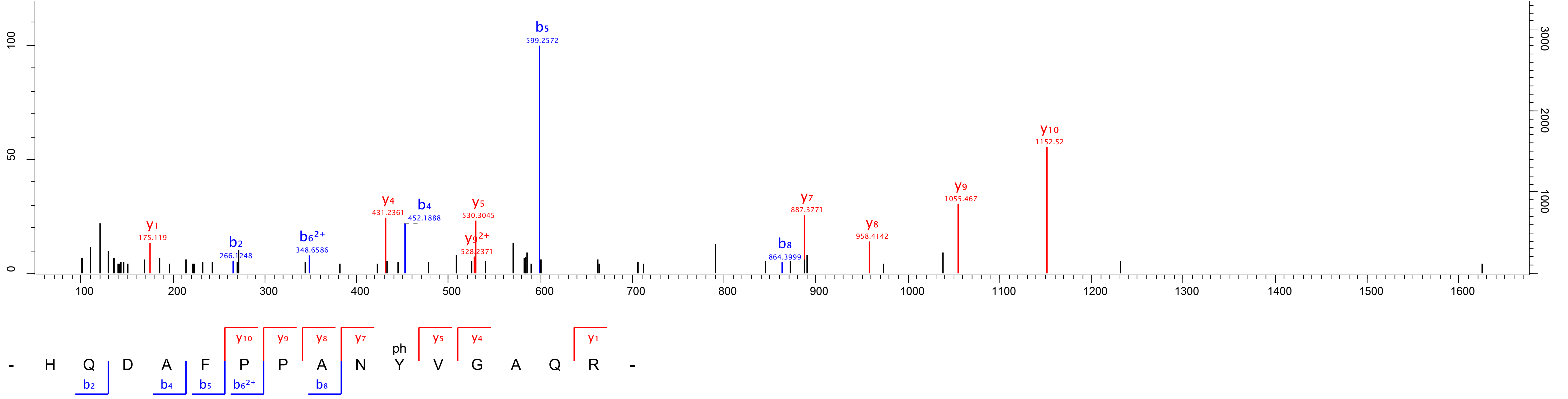

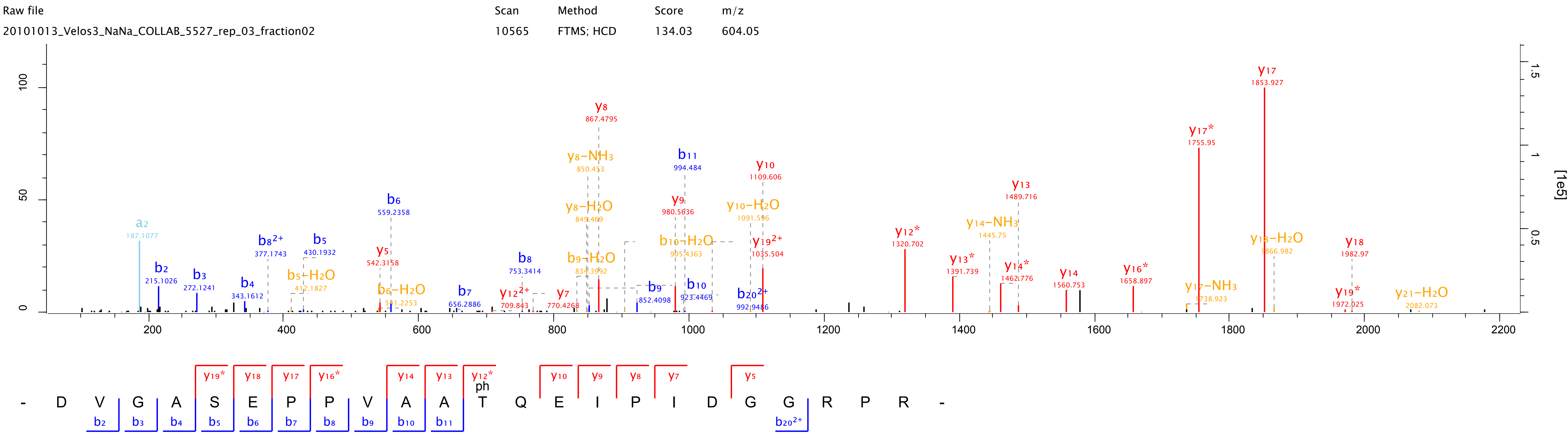

Raw file
20101013_Velos3_NaNa_COLLAB_5527_rep_03_fraction02
Scan
10565
Method
FTMS; HCD
Score
134.03
m/z
604.05
a2 187.1077
b2 215.1026
b3 272.1241
b4 343.1612
b8 2+ 377.1743
b5 430.1932
b5-H2O 412.1827
y5 542.3158
b6 559.2358
b6-H2O 541.2253
b7 656.2886
y12 2+ 709.843
b8 753.3414
y7 770.4268
y8 867.4795
y8-NH3 850.453
y8-H2O 849.469
b9-H2O 834.3992
b9 852.4098
y9 980.5636
b10-H2O 905.4363
b10 923.4469
b11 994.484
y10 1109.606
y10-H2O 1091.596
y19 2+ 1035.504
b20 2+ 992.9486
y12* 1320.702
y13* 1391.739
y14-NH3 1445.75
y13 1489.716
y14* 1462.776
y14 1560.753
y16* 1658.897
y17* 1755.95
y17-NH3 1738.923
y17 1853.927
y18-H2O 1866.982
y18 1982.97
y19* 1972.025
y21-H2O 2082.073
[1e5]
- D V G A S E P P V A A T Q E I P I D G G R P R -
ph
y19* y18 y17 y16* y14 y13 y12* y10 y9 y8 y7 y5
b2 b3 b4 b5 b6 b7 b8 b9 b10 b11 b20 2+

Raw file	Scan	Method	Score	m/z
20101013_Velos3_NaNa_COLLAB_5527_rep_03_fraction05	5060	FTMS; HCD	97.06	527.01

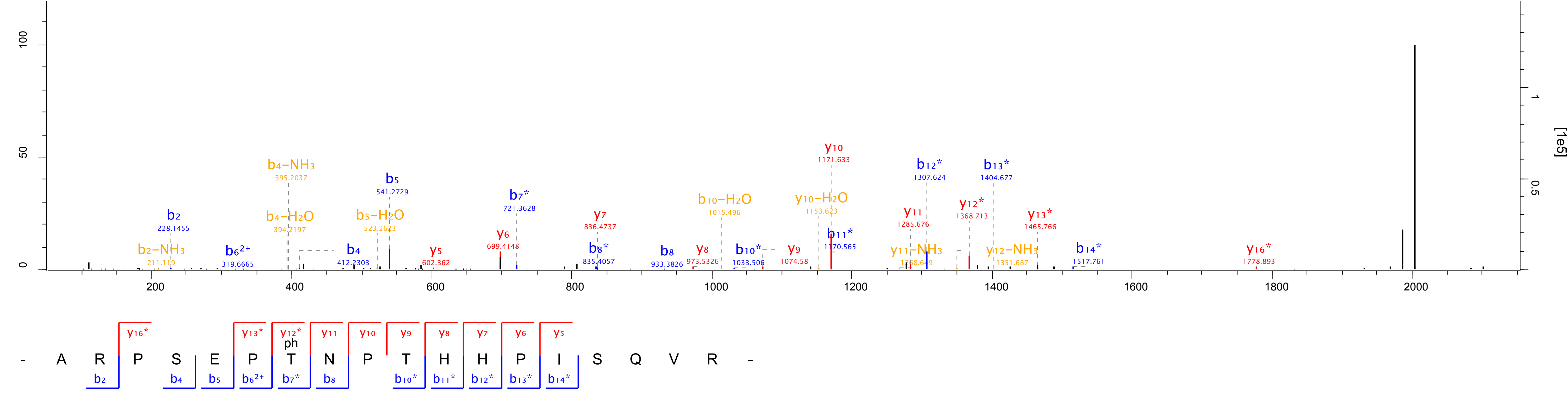

Raw file	Scan	Method	Score	m/z
20101013_Velos3_NaNa_COLLAB_salvage_5527_02	8443	FTMS; HCD	42	839.86

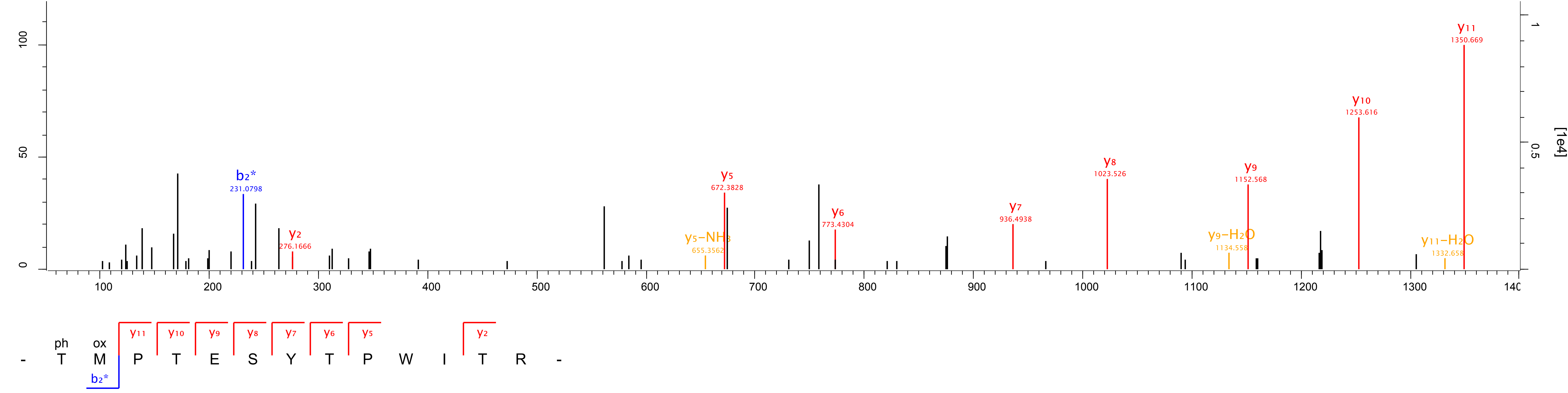

Raw file	Scan	Method	Score	m/z
20101013_Velos3_NaNa_COLLAB_5527_rep_01_fraction02	8412	FTMS; HCD	134.47	730.65

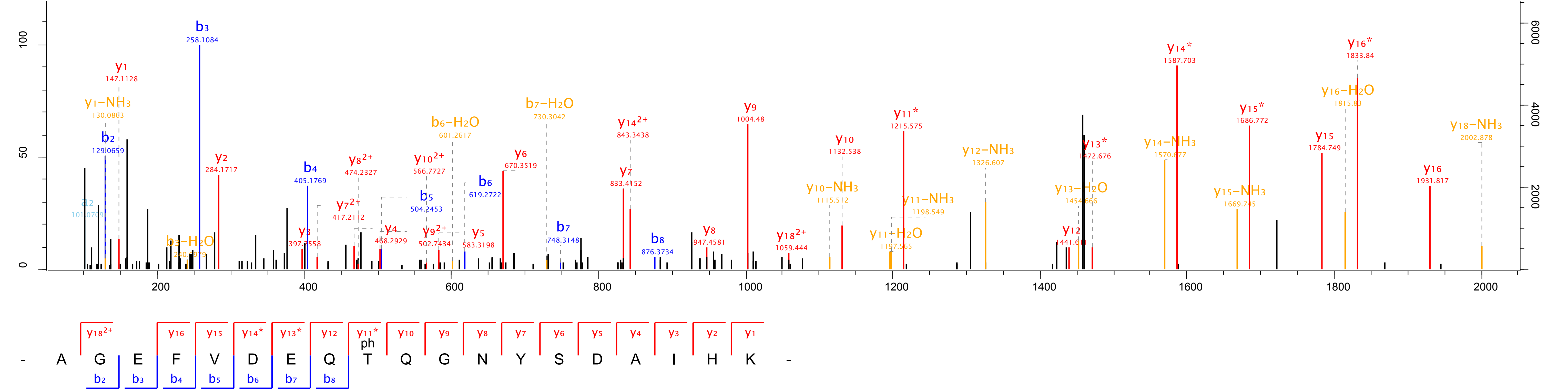

Raw file	Scan	Method	Score	m/z
20101013_Velos3_NaNa_COLLAB_5527_rep_02_fraction02	8092	FTMS; HCD	58.95	730.64

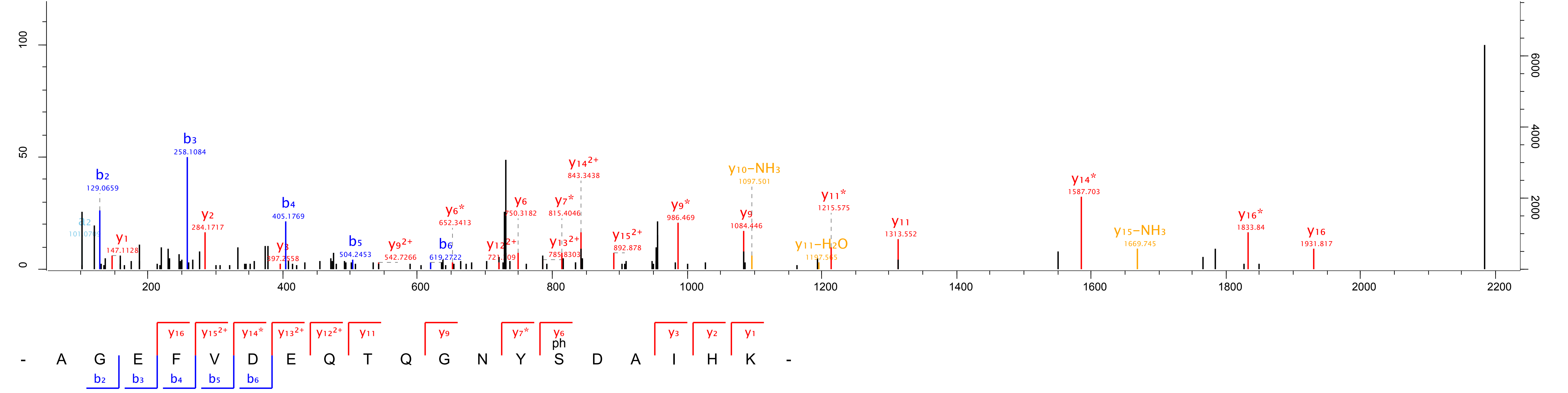

Raw file	Scan	Method	Score	m/z
20101013_Velos3_NaNa_COLLAB_5527_rep_01_fraction03	15690	FTMS; HCD	44.95	886.16

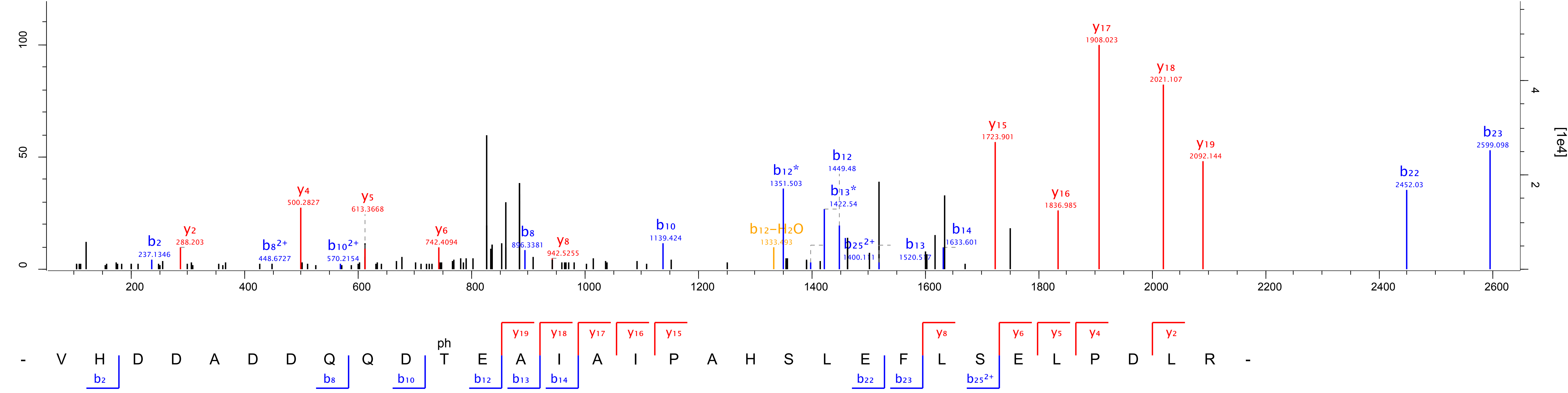

Raw file	Scan	Method	Score	m/z
20101013_Velos3_NaNa_COLLAB_5527_rep_01_flowthru_03	8995	FTMS; HCD	64.1	800.39

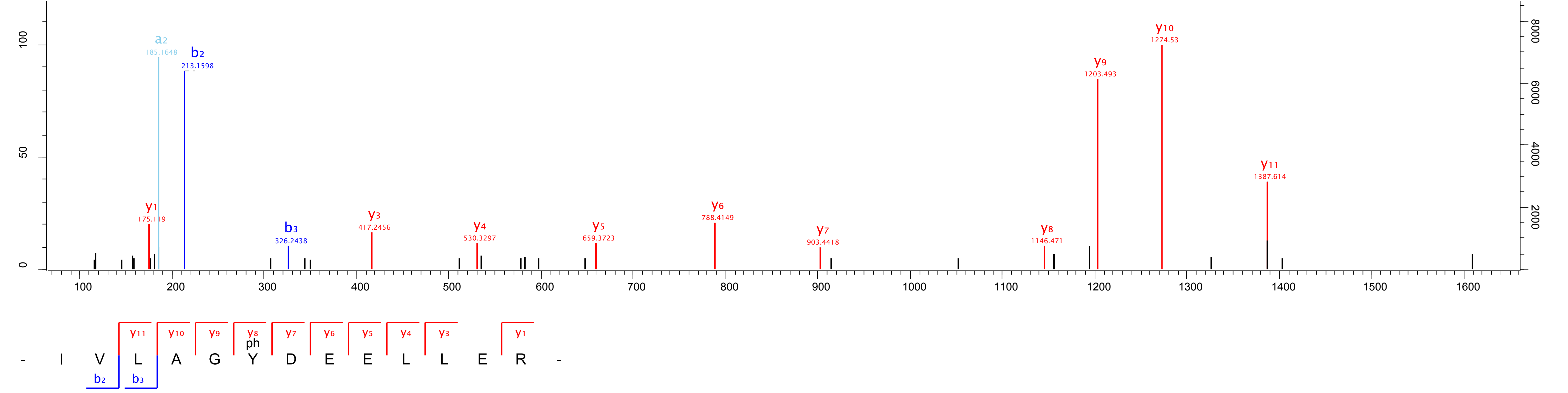

Raw file	Scan	Method	Score	m/z
20101013_Velos3_NaNa_COLLAB_5527_rep_03_fraction01	7770	FTMS; HCD	83.86	472.73

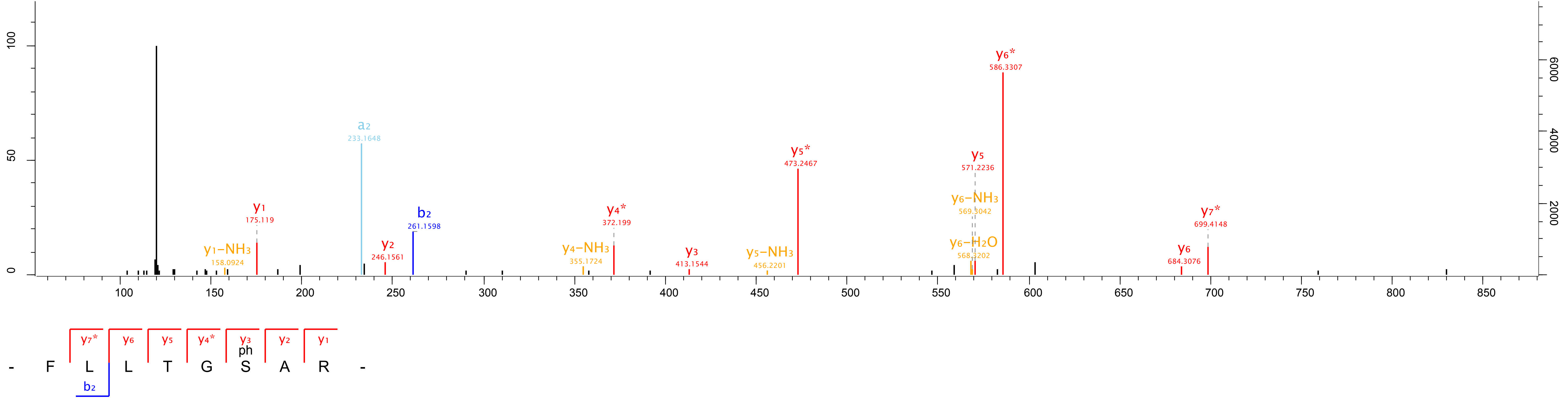

Raw file	Scan	Method	Score	m/z
20101013_Velos3_NaNa_COLLAB_5527_rep_03_fraction03	12314	FTMS; HCD	78.81	450.56

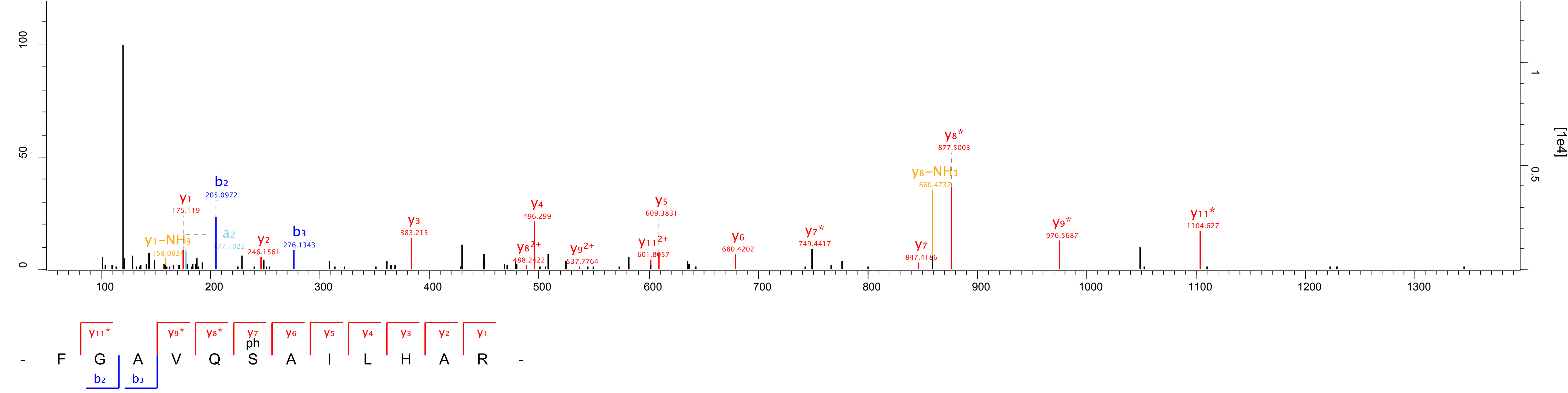

Raw file	Scan	Method	Score	m/z
20101013_Velos3_NaNa_COLLAB_salvage_5527_02	3210	FTMS; HCD	82.53	636.27

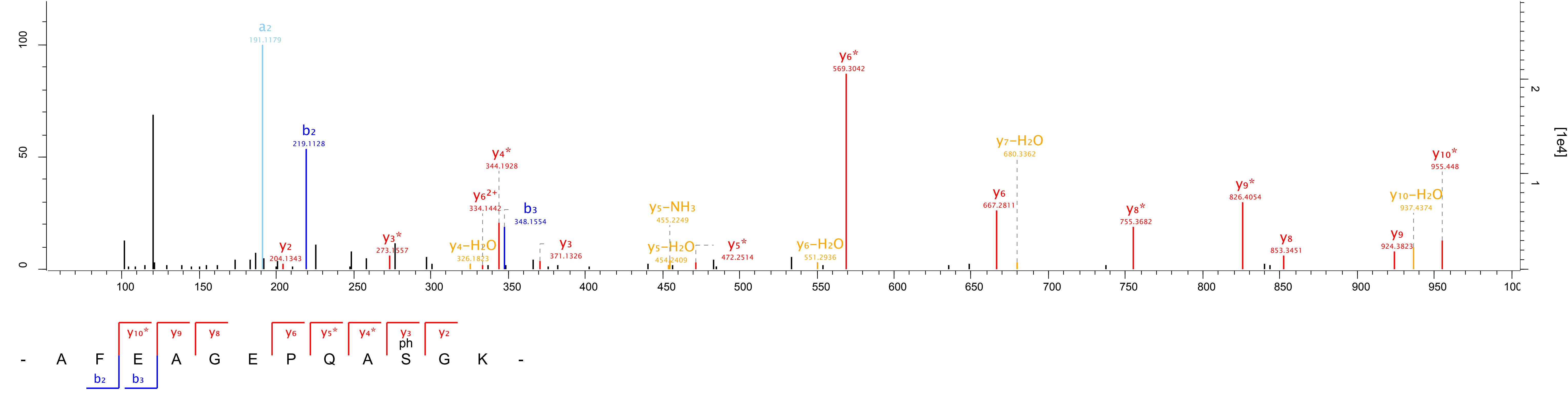

Raw file	Scan	Method	Score	m/z
20101013_Velos3_NaNa_COLLAB_5527_rep_03_fraction01	4373	FTMS; HCD	69.03	591.25

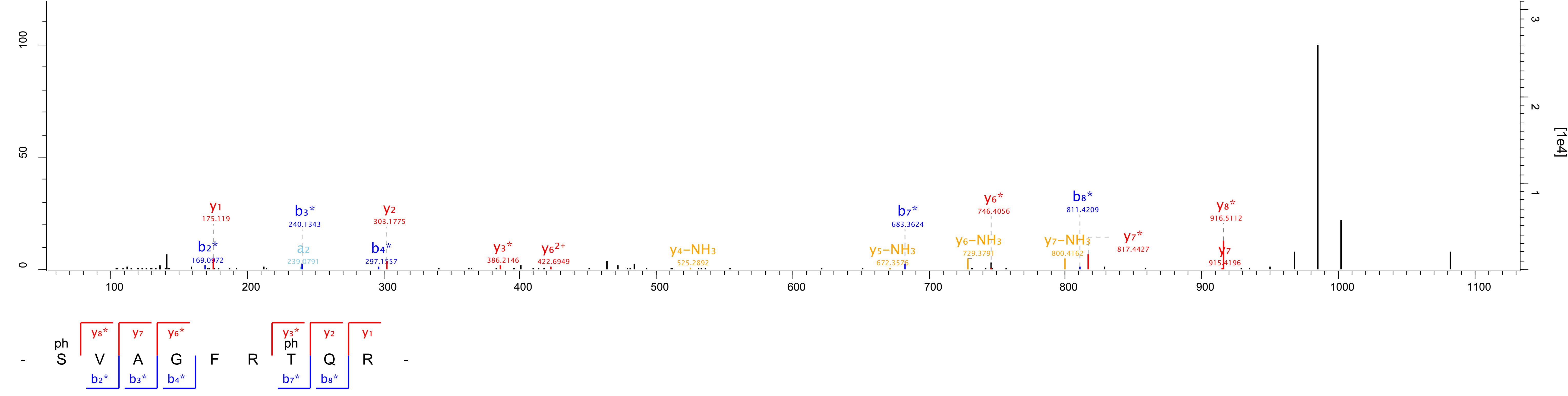

Raw file	Scan	Method	Score	m/z
20101013_Velos3_NaNa_COLLAB_salvage_5527_02	3237	FTMS; HCD	44.83	420.52

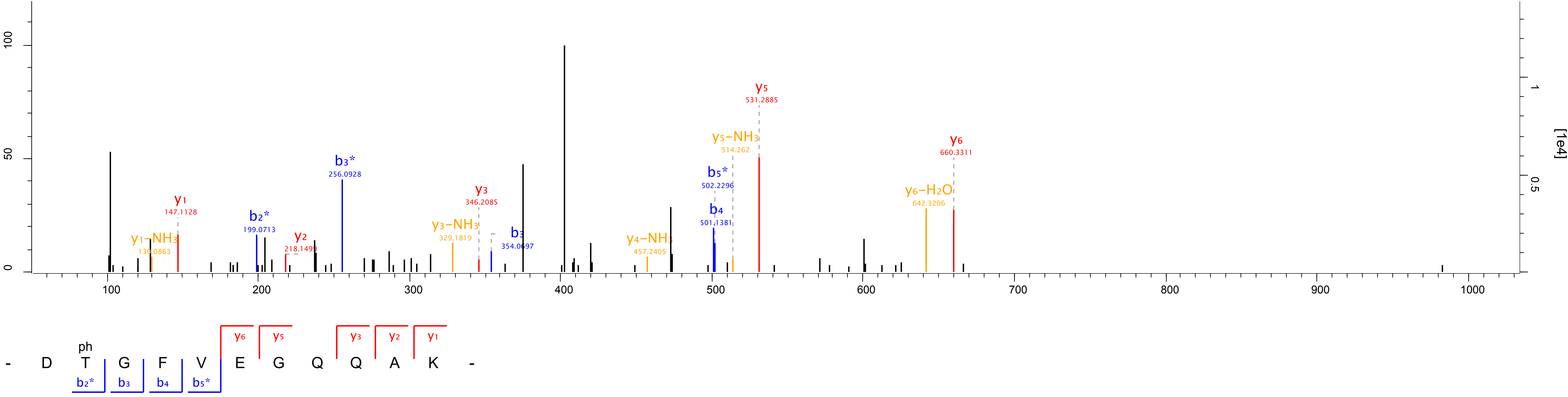

Raw file	Scan	Method	Score	m/z
20101013_Velos3_NaNa_COLLAB_5527_rep_03_flowthru_01	7959	FTMS; HCD	52.66	591.26

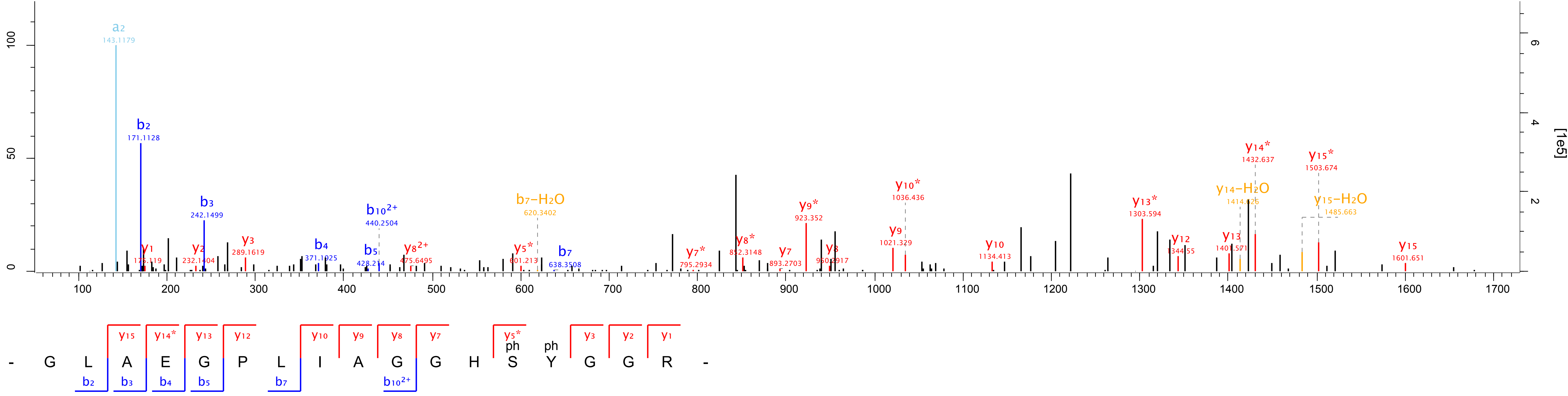

Raw file	Scan	Method	Score	m/z
20101013_Velos3_NaNa_COLLAB_5527_rep_03_fraction02	7648	FTMS; HCD	153.25	641.94

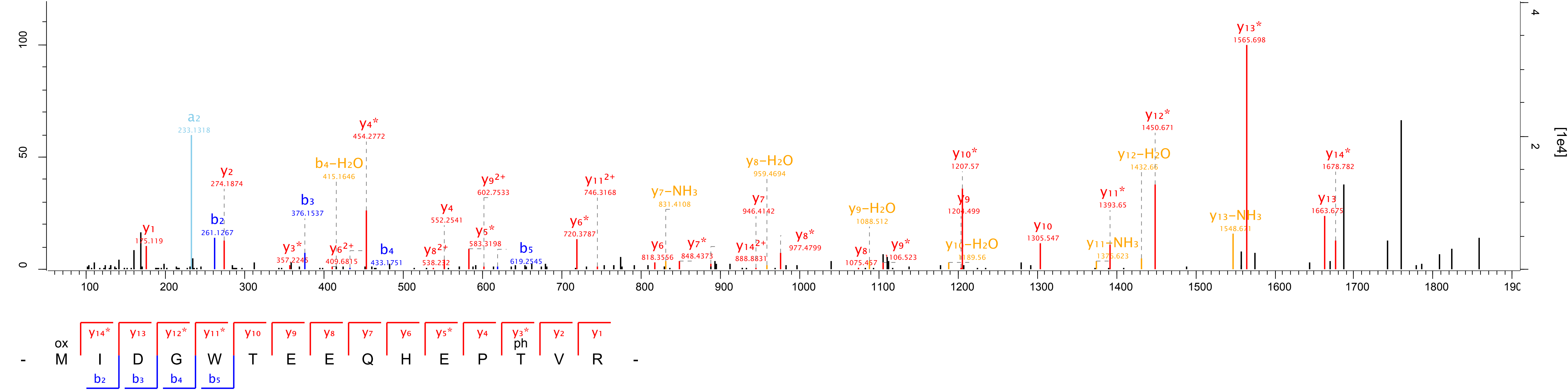

Raw file	Scan	Method	Score	m/z
20101013_Velos3_NaNa_COLLAB_salvage_5527_02	4759	FTMS; HCD	90.71	680.3

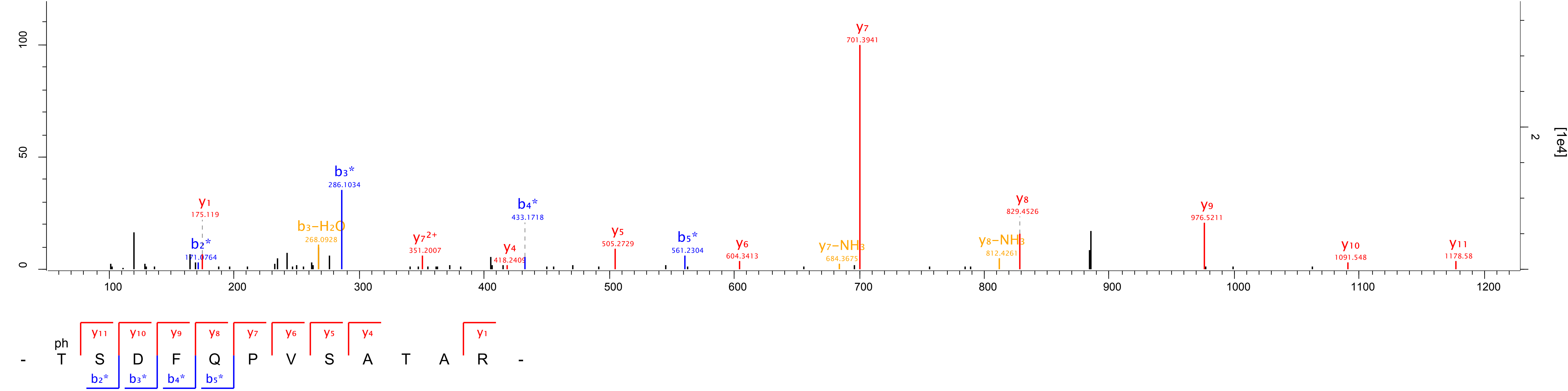

Raw file	Scan	Method	Score	m/z
20101013_Velos3_NaNa_COLLAB_5527_rep_03_flowthru_03	6813	FTMS; HCD	102.98	986.86

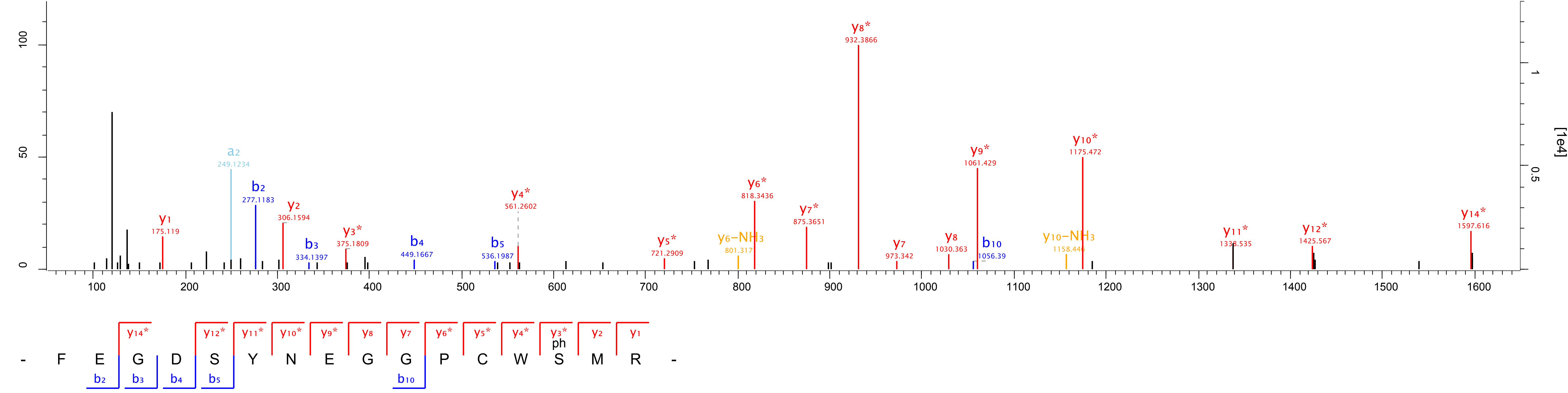

Raw file	Scan	Method	Score	m/z
20101013_Velos3_NaNa_COLLAB_5527_rep_03_flowthru_01	9784	FTMS; HCD	71.15	982.9

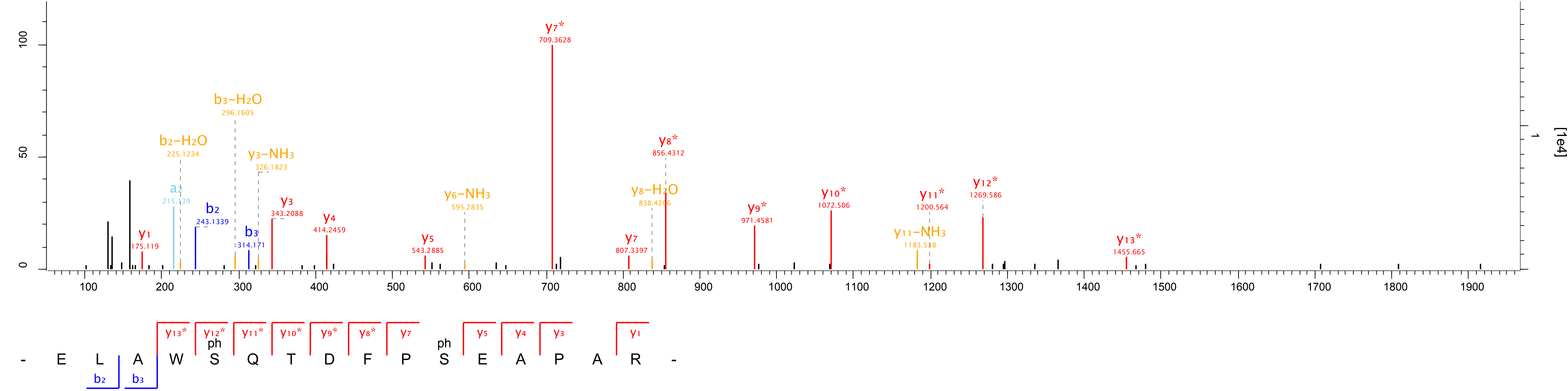

Raw file	Scan	Method	Score	m/z
20101013_Velos3_NaNa_COLLAB_5527_rep_03_flowthru_01	9784	FTMS; HCD	71.15	982.9

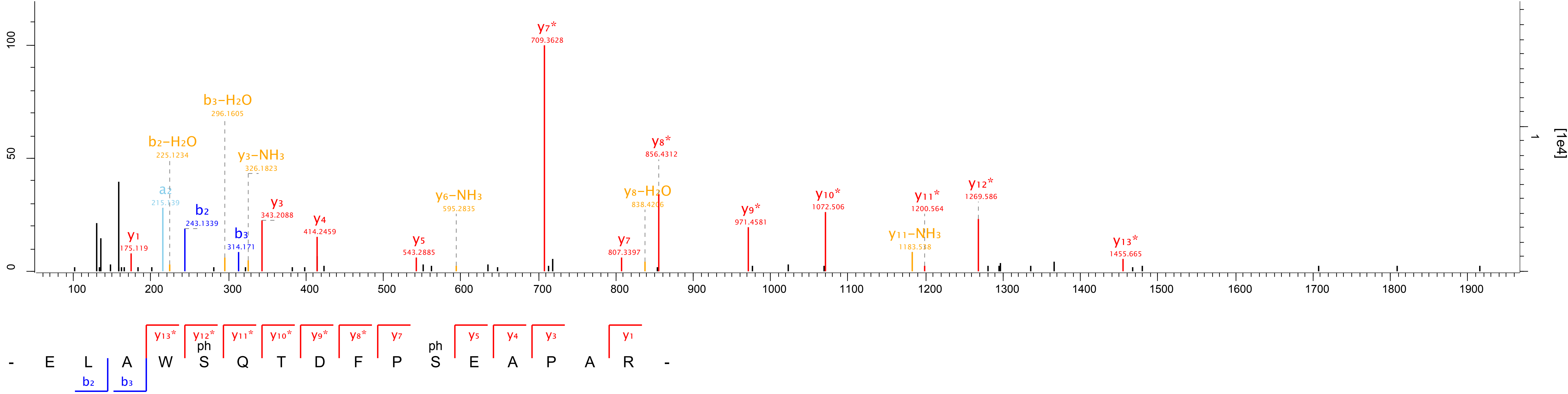

Raw file	Scan	Method	Score	m/z
20101013_Velos3_NaNa_COLLAB_5527_rep_02_flowthru_01	9892	FTMS; HCD	84.51	504.91

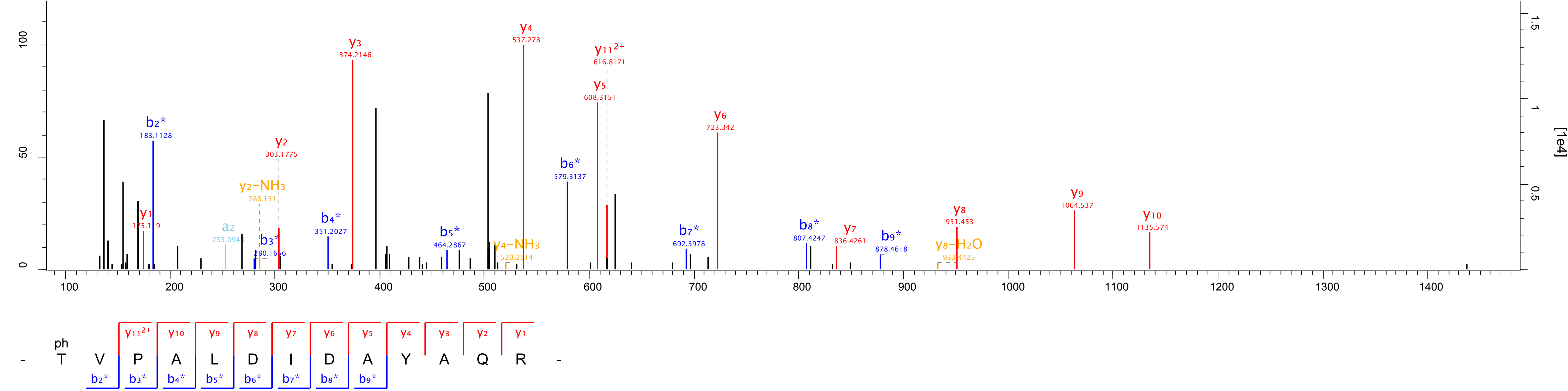

Raw file	Scan	Method	Score	m/z
20101013_Velos3_NaNa_COLLAB_salvage_5527_02	2634	FTMS; HCD	120.86	503.73

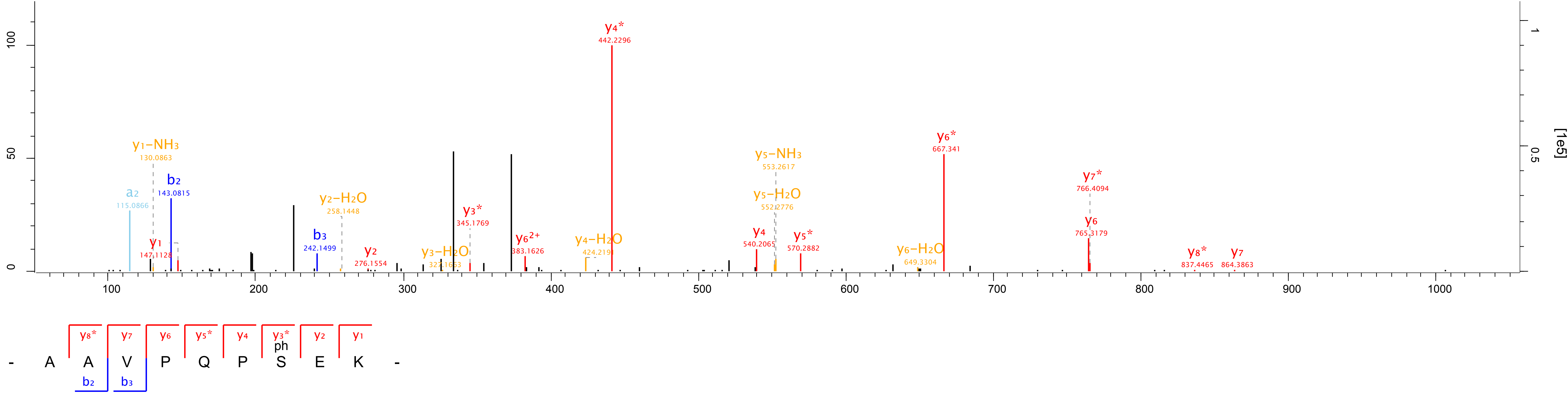

Raw file	Scan	Method	Score	m/z
20101013_Velos3_NaNa_COLLAB_5527_rep_03_fraction01	2215	FTMS; HCD	54.9	565.23

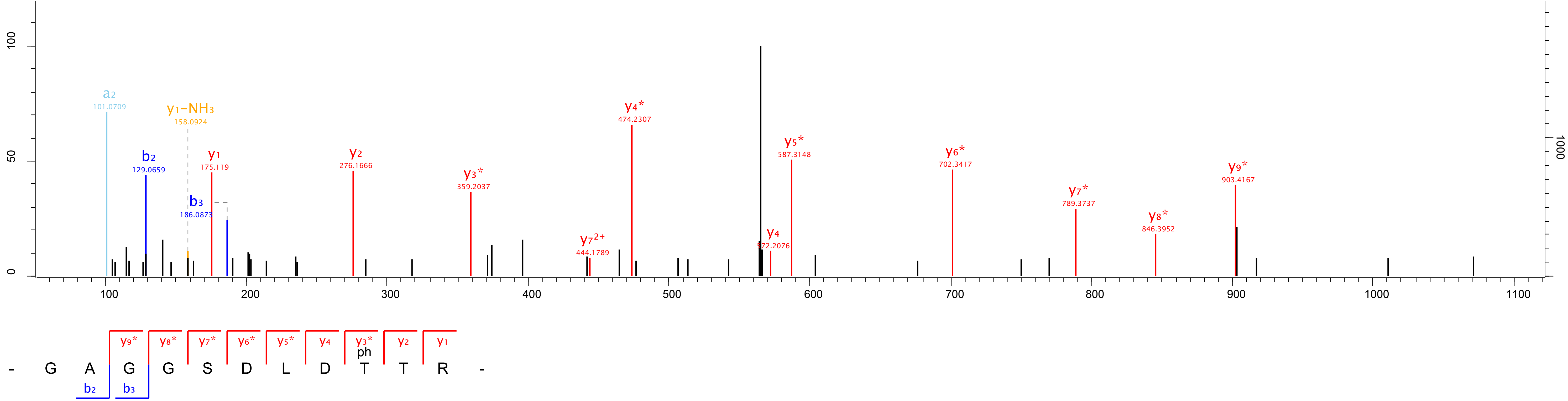

Raw file	Scan	Method	Score	m/z
20101013_Velos3_NaNa_COLLAB_5527_rep_03_fraction01	2784	FTMS; HCD	52.58	528.25

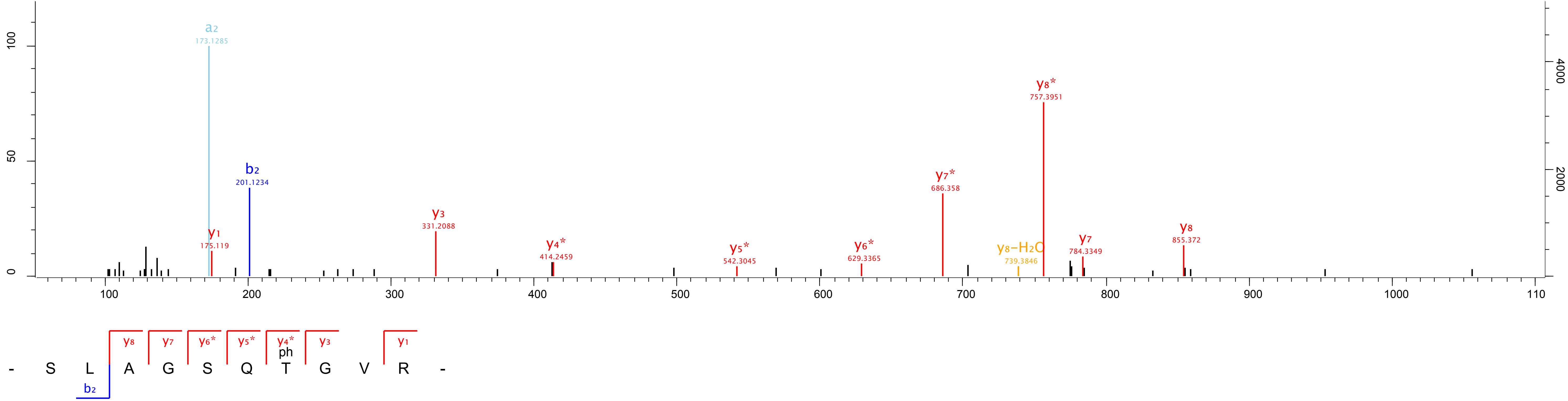

Raw file	Scan	Method	Score	m/z
20101013_Velos3_NaNa_COLLAB_salvage_5527_02	5642	FTMS; HCD	45.88	526.57

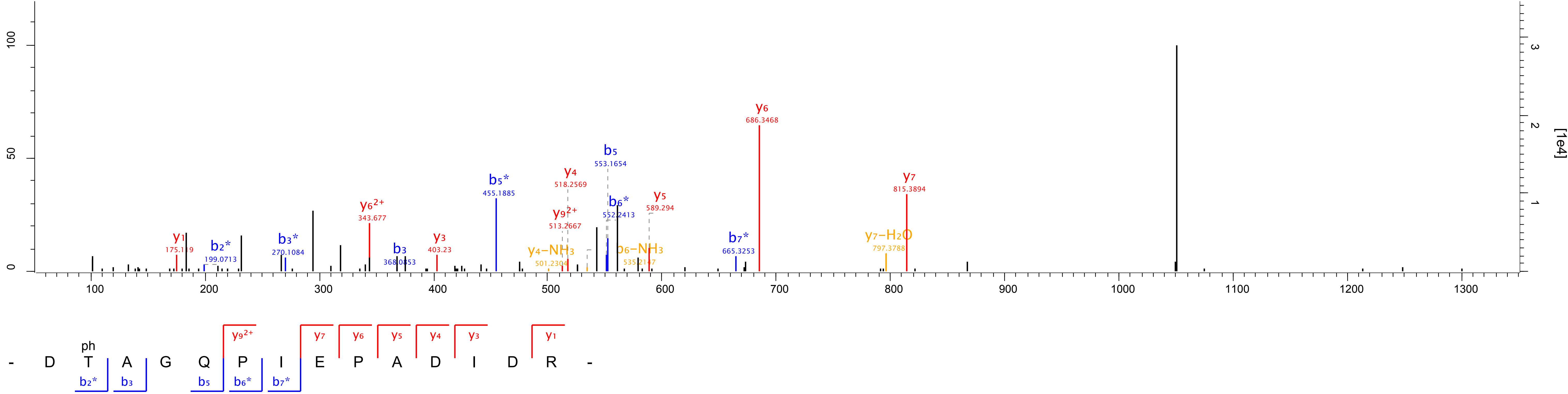

Raw file	Scan	Method	Score	m/z
20101013_Velos3_NaNa_COLLAB_salvage_5527_02	4446	FTMS; HCD	105.9	805.86

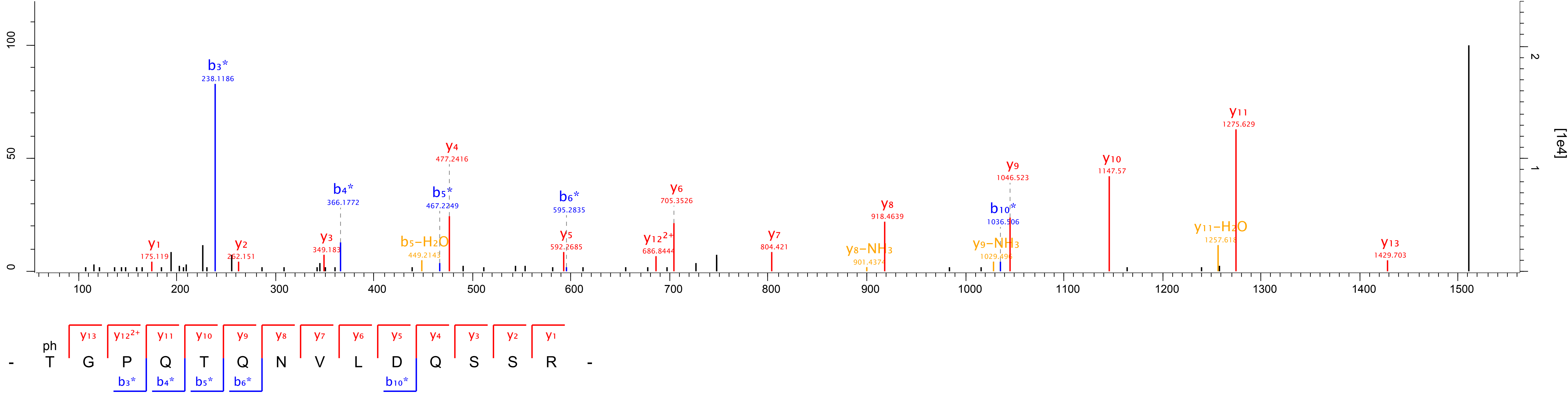

Raw file	Scan	Method	Score	m/z
20101013_Velos3_NaNa_COLLAB_5527_rep_01_flowthru_02	2399	FTMS; HCD	57.53	495.68

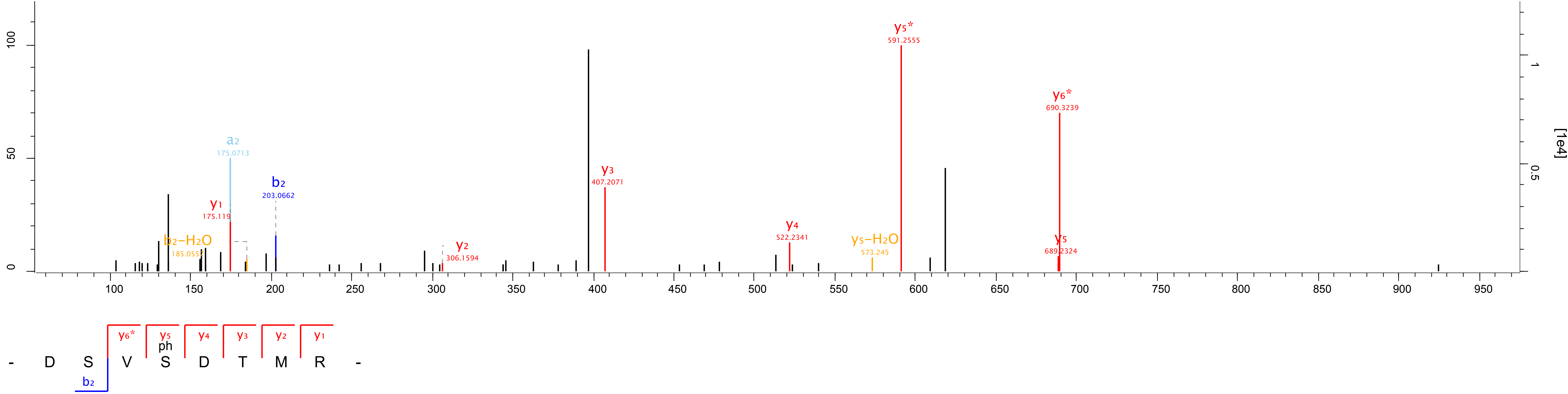

Raw file	Scan	Method	Score	m/z
20101013_Velos3_NaNa_COLLAB_5527_rep_01_flowthru_03	6652	FTMS; HCD	58.17	598.78

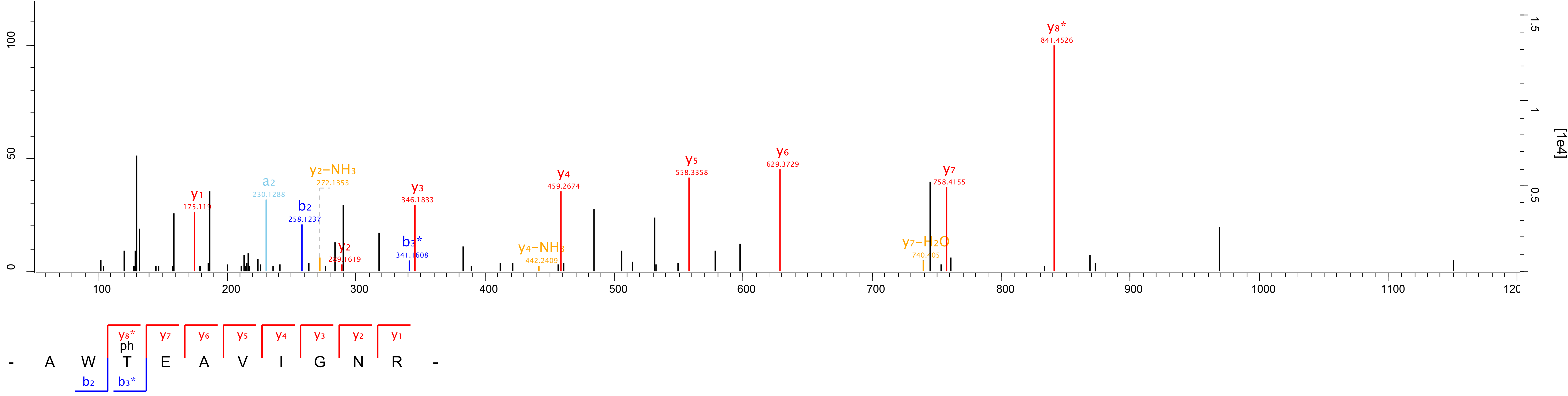

Raw file	Scan	Method	Score	m/z
20101013_Velos3_NaNa_COLLAB_5527_rep_03_fraction02	5055	FTMS; HCD	44.54	456.24

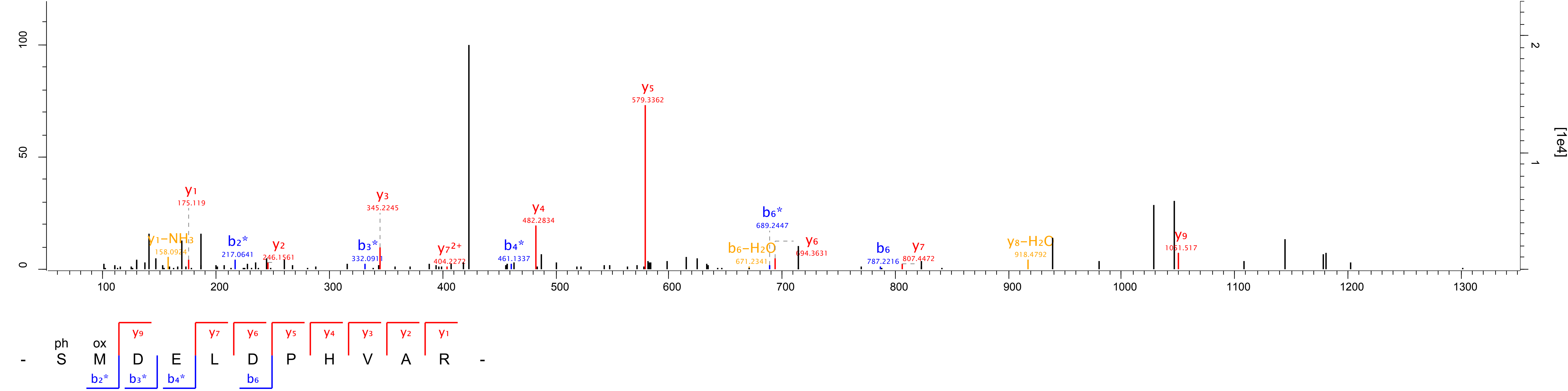

Raw file	Scan	Method	Score	m/z
20101013_Velos3_NaNa_COLLAB_5527_rep_01_fraction04	3902	FTMS; HCD	80.24	654.98

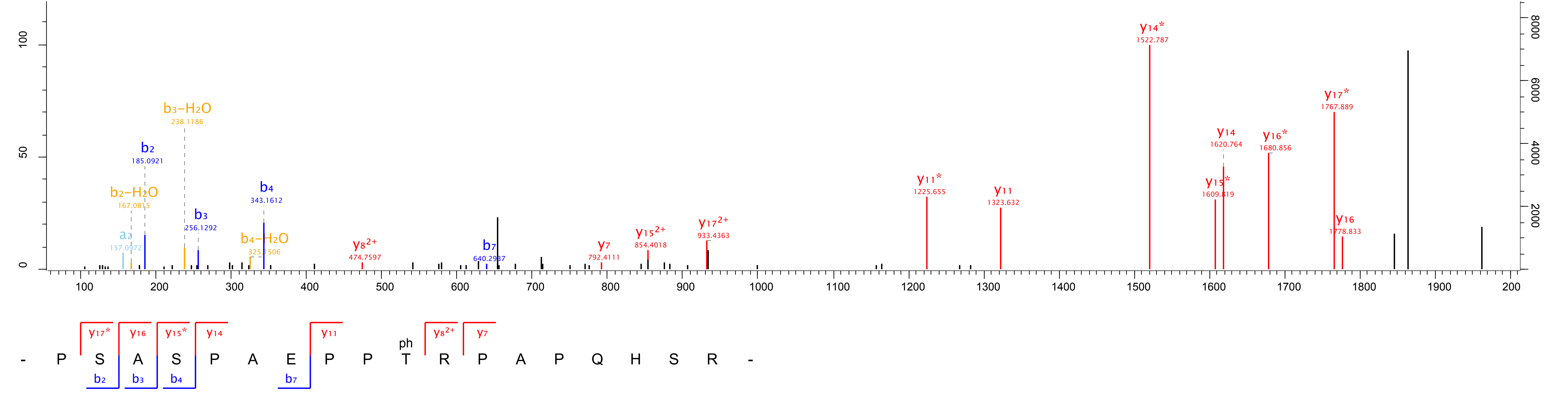

Raw file	Scan	Method	Score	m/z
20101013_Velos3_NaNa_COLLAB_salvage_5527_02	3092	FTMS; HCD	106.26	555.25

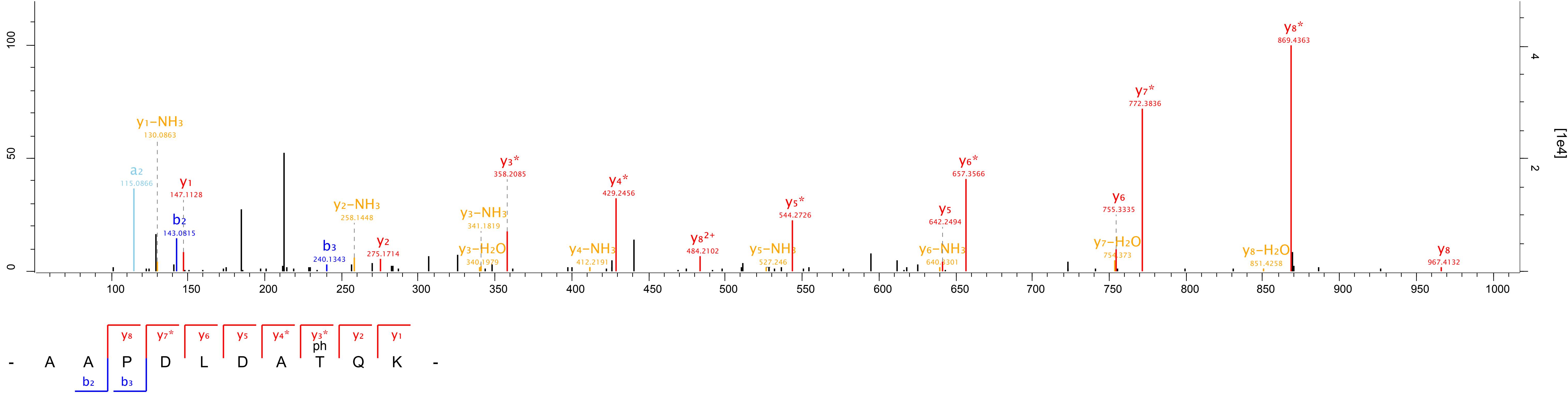

Raw file	Scan	Method	Score	m/z
20101013_Velos3_NaNa_COLLAB_5527_rep_03_fraction03	9236	FTMS; HCD	41.55	655.05

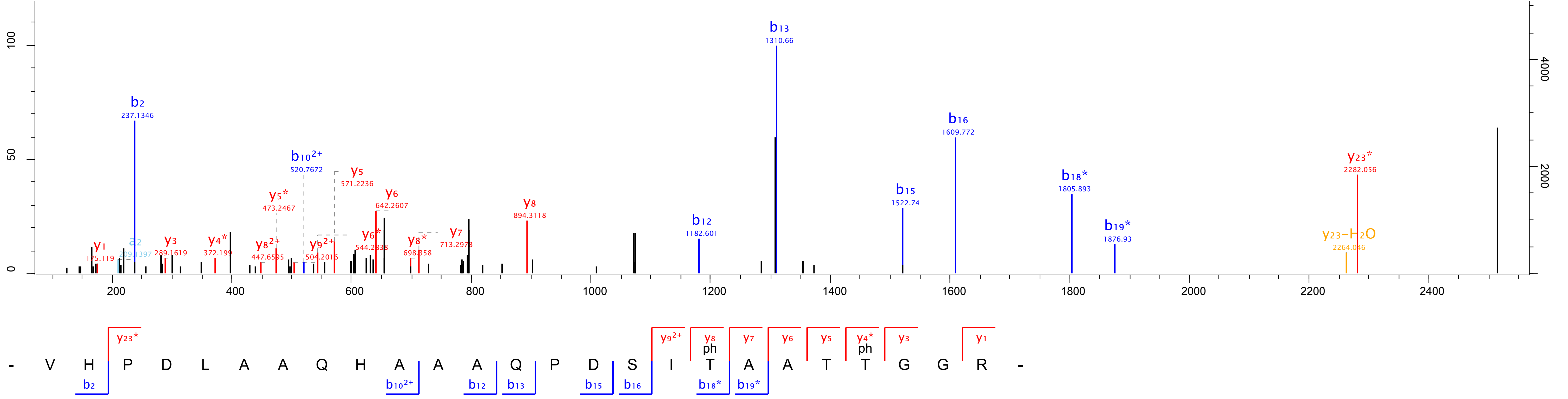

Raw file	Scan	Method	Score	m/z
20101013_Velos3_NaNa_COLLAB_5527_rep_03_flowthru_01	3811	FTMS; HCD	74.85	580.25

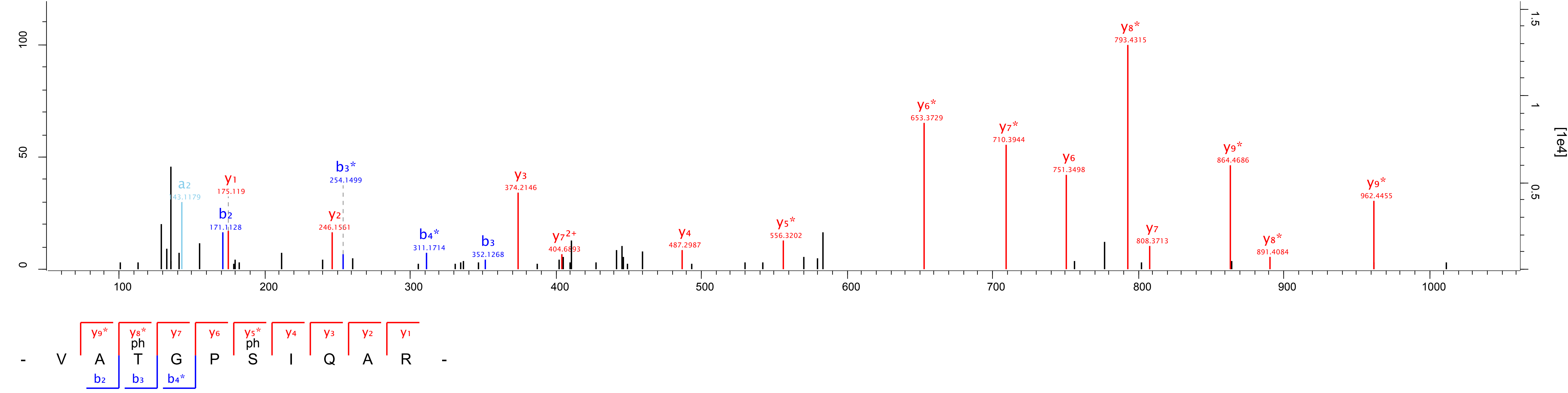

Raw file	Scan	Method	Score	m/z
20101013_Velos3_NaNa_COLLAB_5527_rep_03_fraction01	8960	FTMS; HCD	68.54	921.1

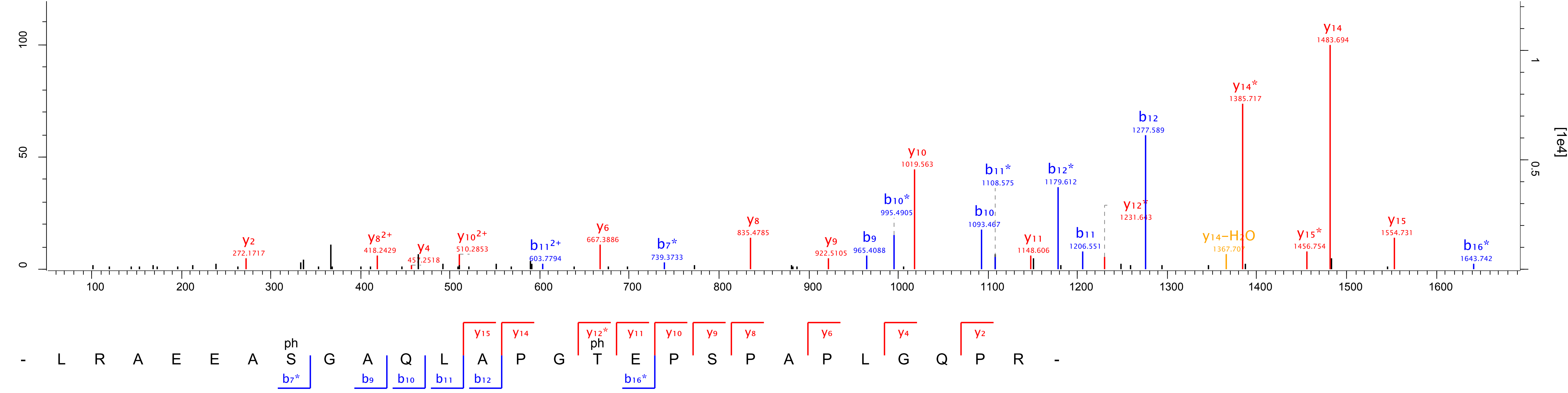

Raw file	Scan	Method	Score	m/z
20101013_Velos3_NaNa_COLLAB_salvage_5527_02	3652	FTMS; HCD	88.71	611.8

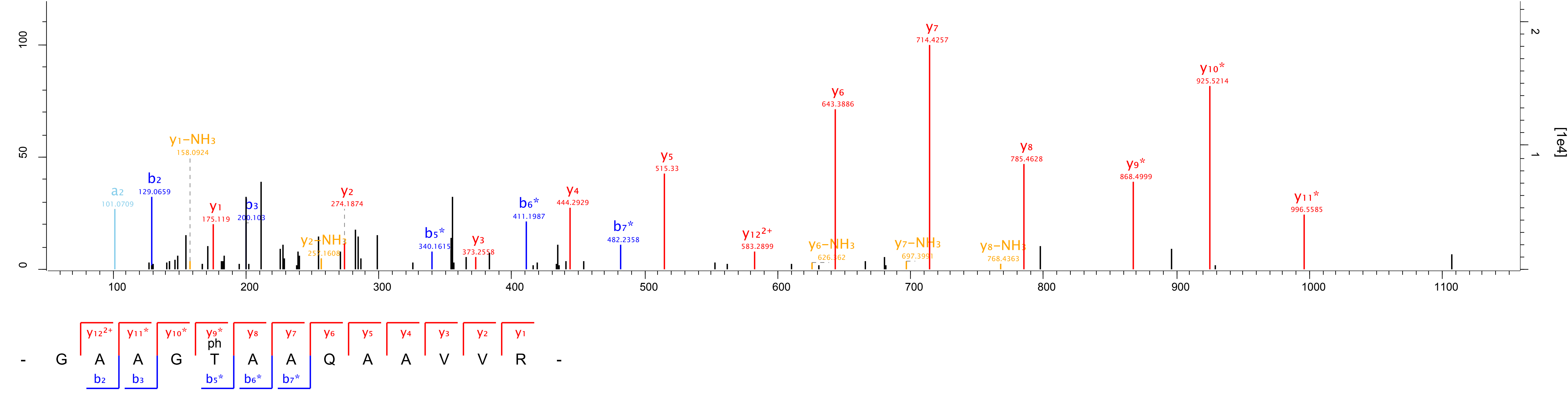

Raw file	Scan	Method	Score	m/z
20101013_Velos3_NaNa_COLLAB_5527_rep_01_fraction03	8811	FTMS; HCD	113.98	551.93

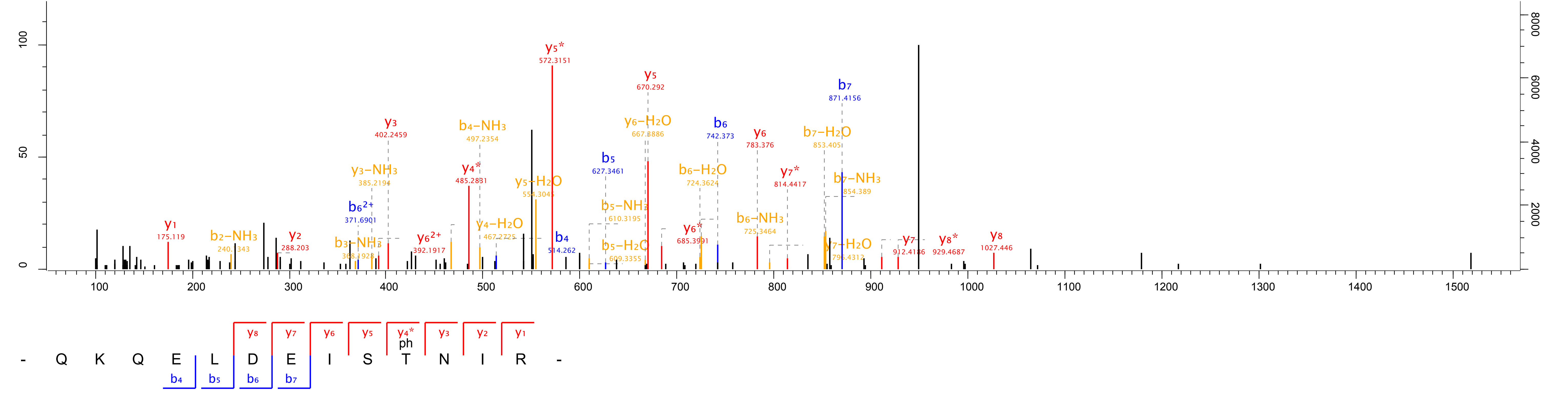

Raw file	Scan	Method	Score	m/z
20101013_Velos3_NaNa_COLLAB_5527_rep_01_flowthru_01	8882	FTMS; HCD	107.03	837.36

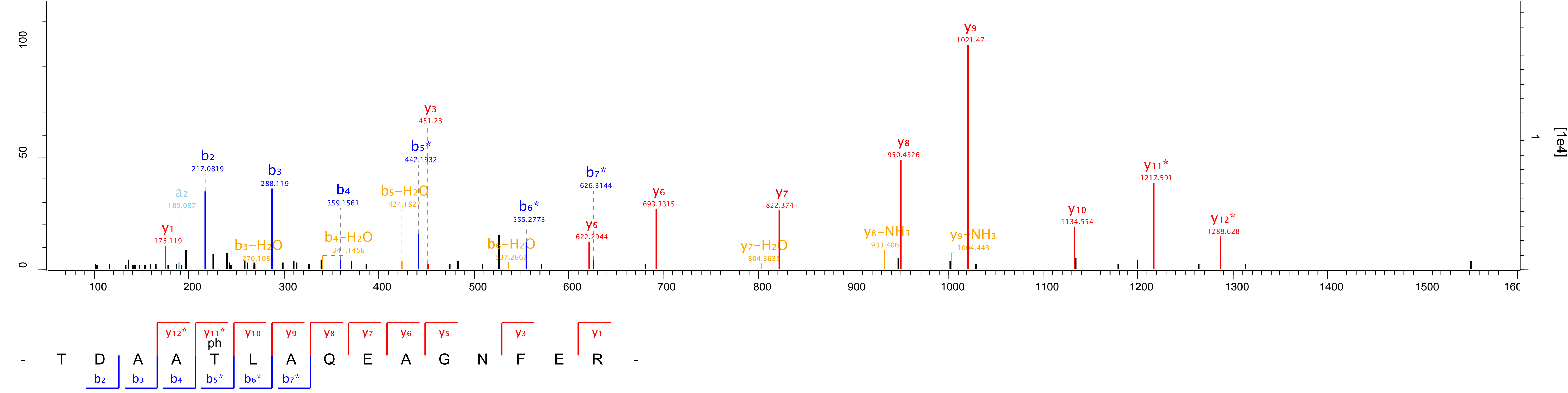

Raw file	Scan	Method	Score	m/z
20101013_Velos3_NaNa_COLLAB_5527_rep_03_fraction01	2429	FTMS; HCD	80.04	494.72

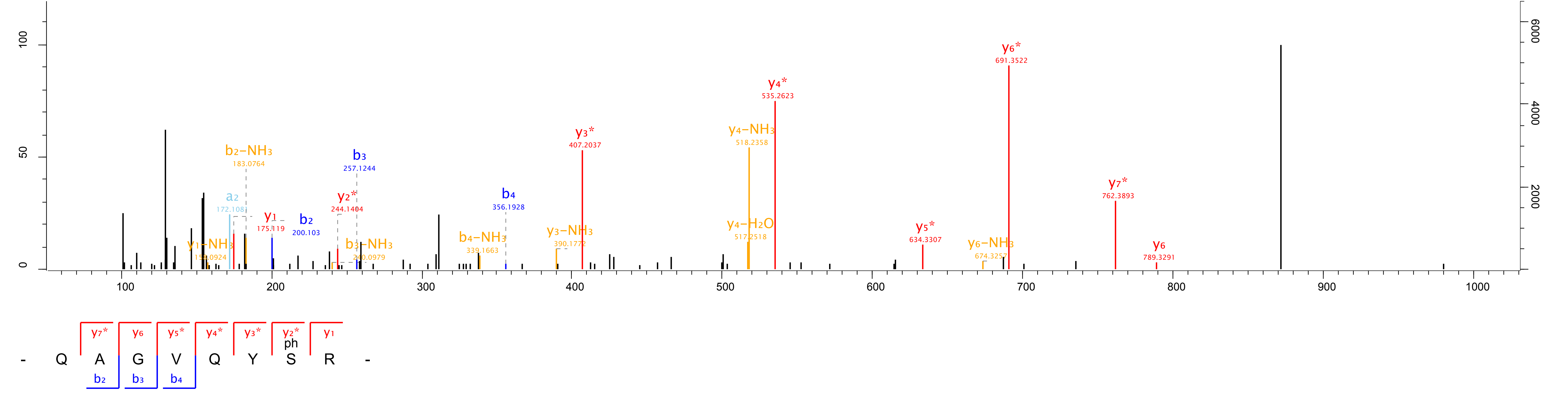

Raw file	Scan	Method	Score	m/z
20101013_Velos3_NaNa_COLLAB_5527_rep_01_flowthru_01	8745	FTMS; HCD	72.43	874.85

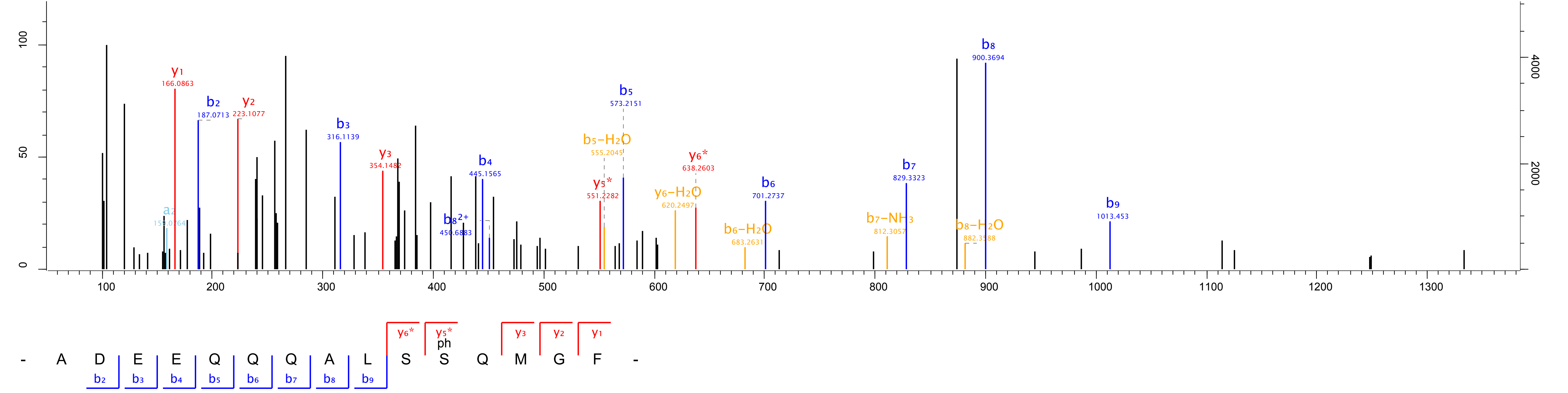

Raw file	Scan	Method	Score	m/z
20101013_Velos3_NaNa_COLLAB_salvage_5527_02	9292	FTMS; HCD	87.68	848.39

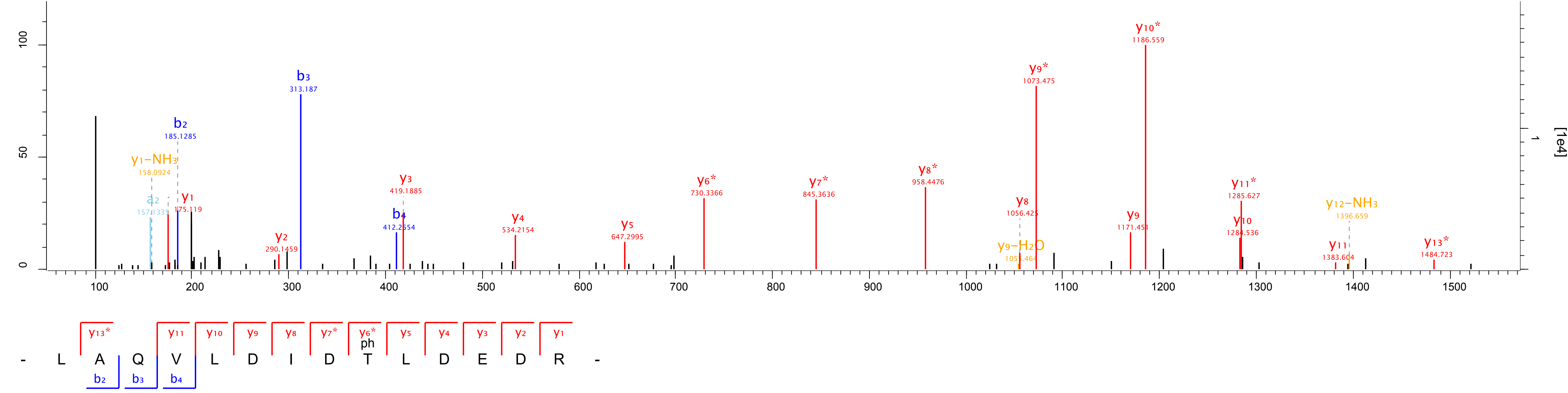

Raw file	Scan	Method	Score	m/z
20101013_Velos3_NaNa_COLLAB_5527_rep_03_fraction01	11927	FTMS; HCD	70.81	988.17

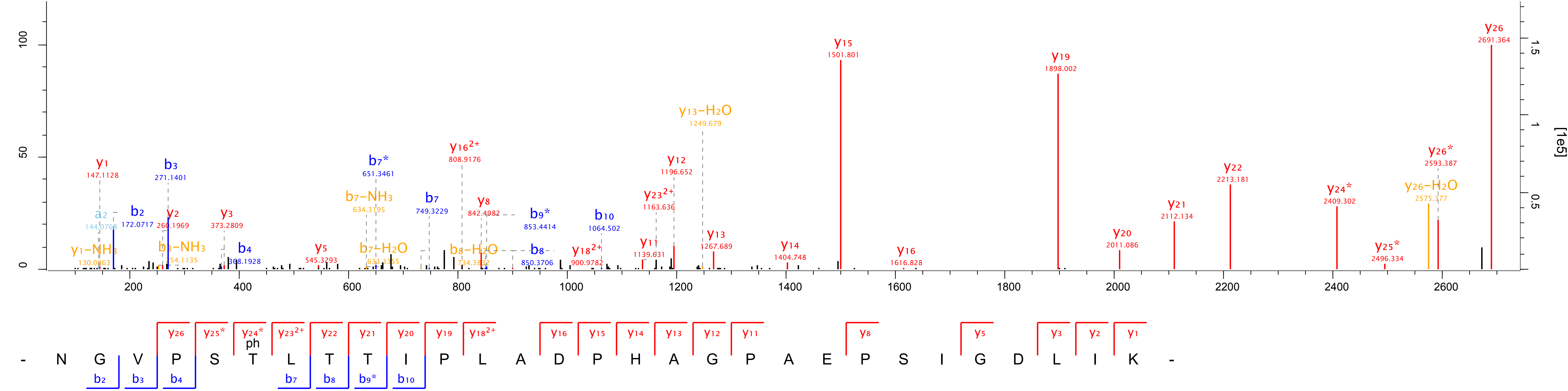

Raw file	Scan	Method	Score	m/z
20101013_Velos3_NaNa_COLLAB_5527_rep_03_fraction01	2645	FTMS; HCD	56.2	542.24

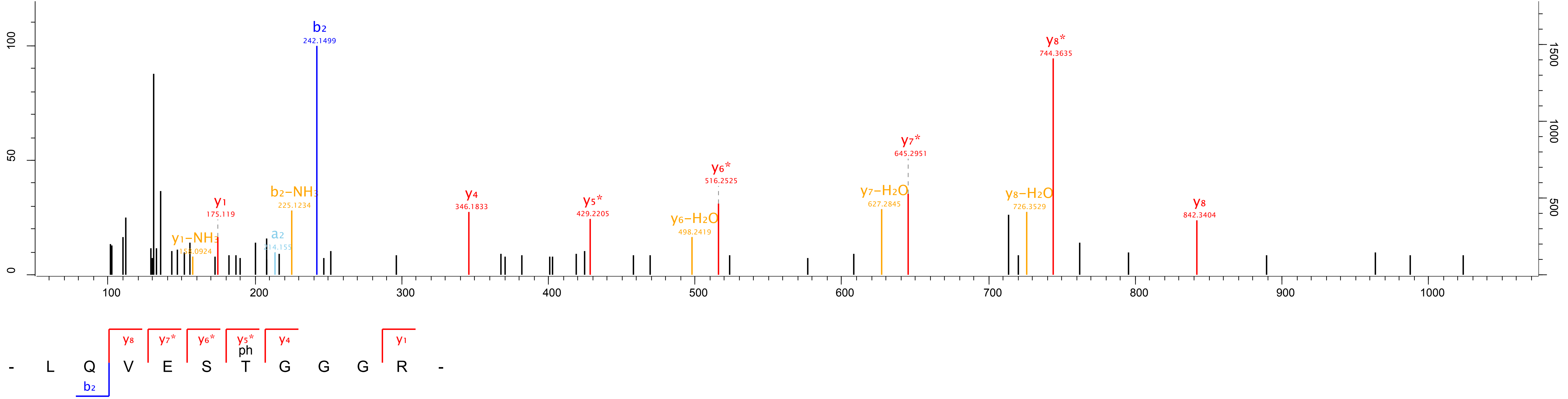

Raw file	Scan	Method	Score	m/z
20101013_Velos3_NaNa_COLLAB_5527_rep_02_flowthru_01	10560	FTMS; HCD	165.66	805.87

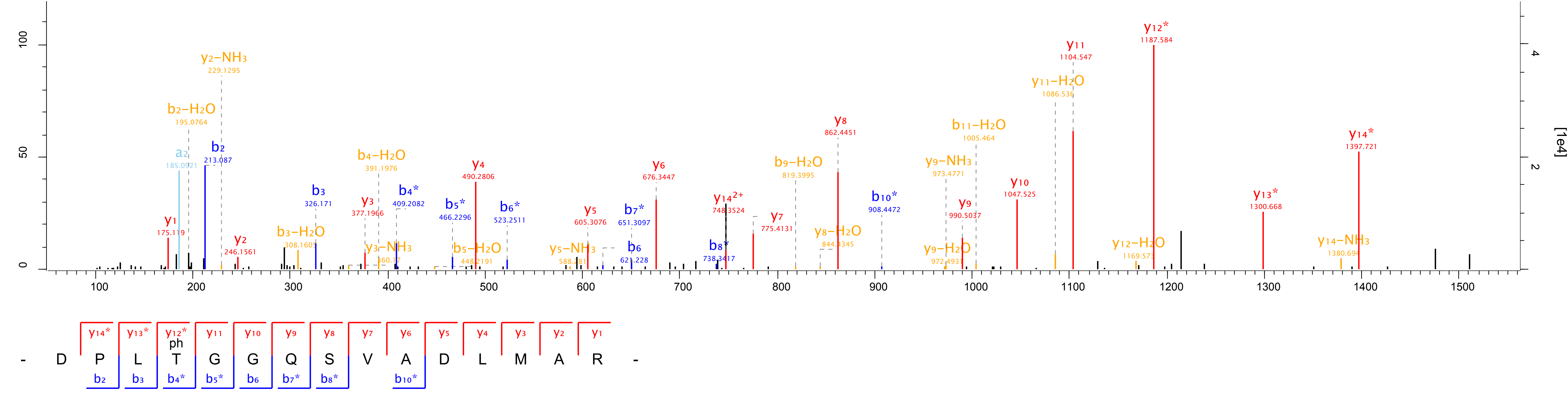

Raw file	Scan	Method	Score	m/z
20101013_Velos3_NaNa_COLLAB_5527_rep_01_flowthru_01	7419	FTMS; HCD	77.08	891.37

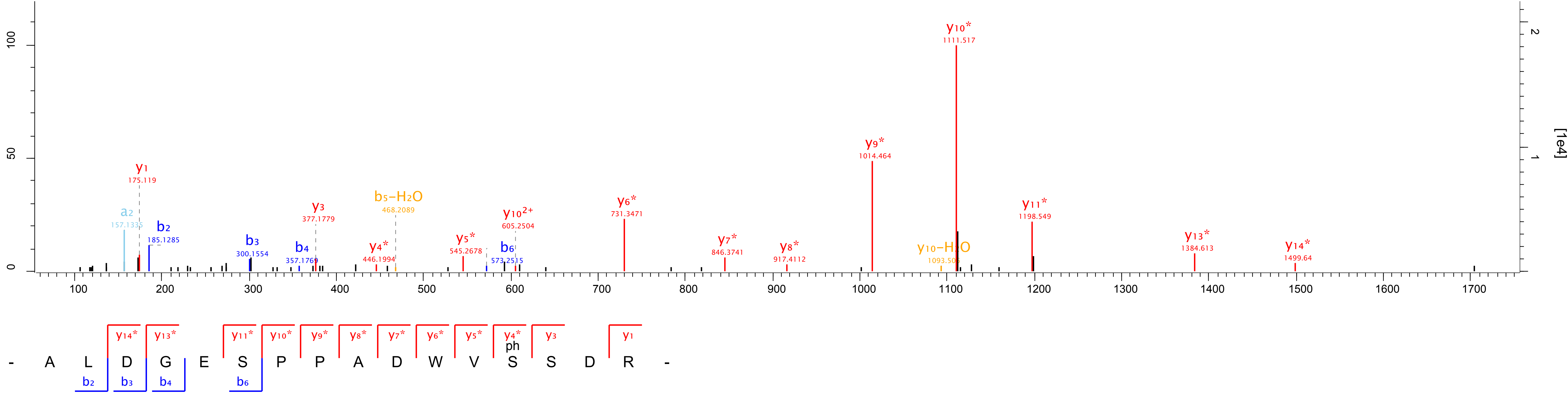

Raw file	Scan	Method	Score	m/z
20101013_Velos3_NaNa_COLLAB_5527_rep_01_fraction04	10795	FTMS; HCD	79.34	657.66

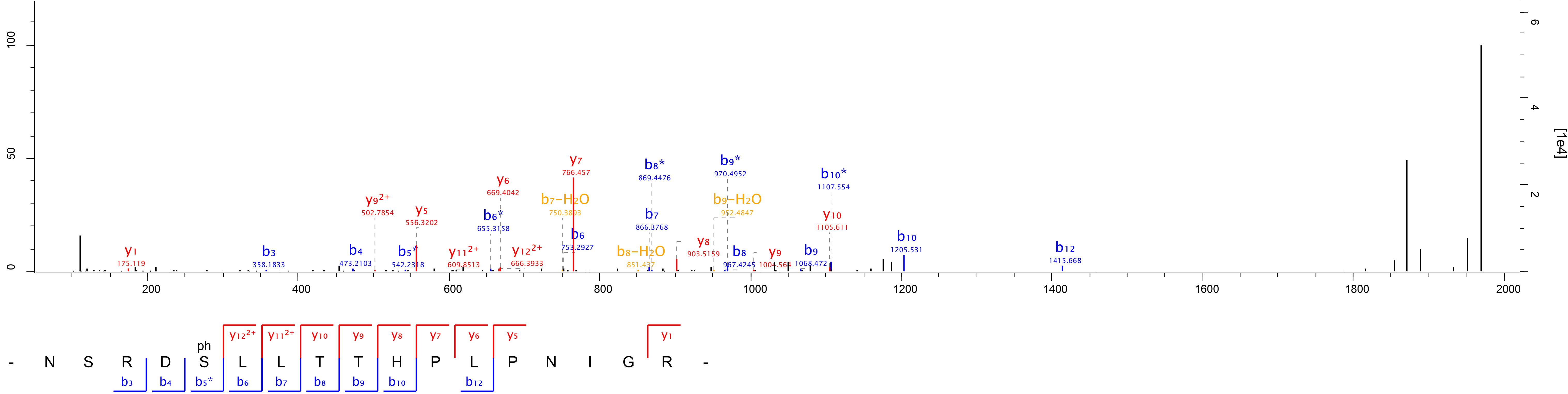

Raw file	Scan	Method	Score	m/z
20101013_Velos3_NaNa_COLLAB_salvage_5527_02	5710	FTMS; HCD	85.49	728.32

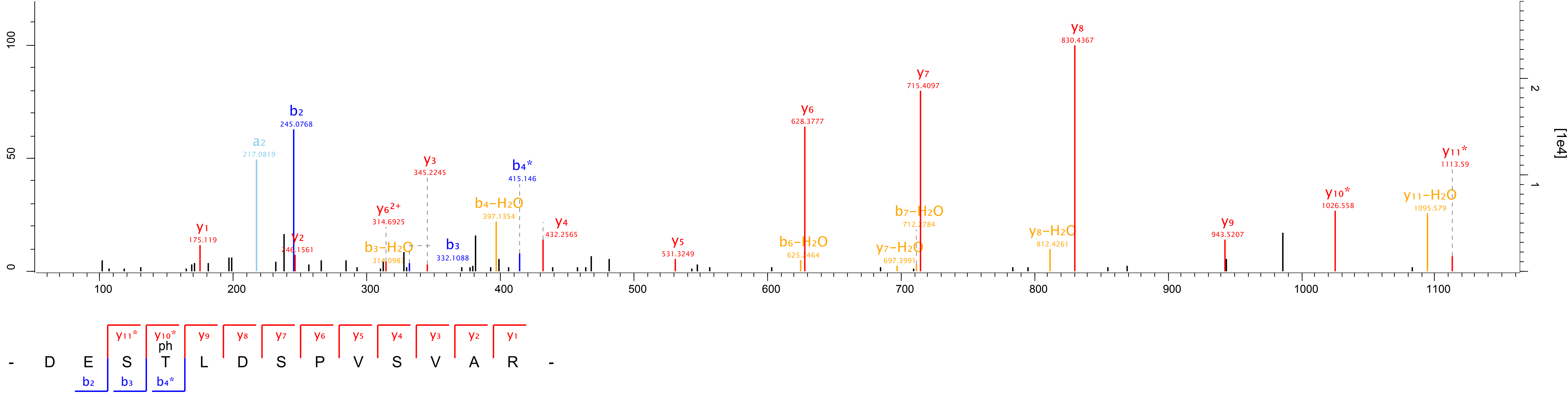

Supplement: Supplementary file 4 [file Image2.PDF]
